# ACE2-Fc and DPP4-Fc decoy receptors against SARS-CoV-2 and MERS-CoV variants: a quick therapeutic option for current and future coronaviruses outbreaks

**DOI:** 10.1093/abt/tbad030

**Published:** 2023-12-12

**Authors:** Mohamed A Alfaleh, Reem M Alsulaiman, Sarah A Almahboub, Leena Nezamuldeen, Ayat Zawawi, Najwa D Aljehani, Muhammad Yasir, Rwaa H Abdulal, Rami Alkhaldi, Assala Helal, Sawsan S Alamri, Jana Malki, Rowa Y Alhabbab, Turki S Abujamel, Nabil A Alhakamy, Aisha Alnami, Abdullah Algaissi, Mazen Hassanain, Anwar M Hashem

**Affiliations:** Department of Pharmaceutics, Faculty of Pharmacy, King Abdulaziz University, Jeddah 21859, Saudi Arabia; Vaccines and Immunotherapy Unit, King Fahd Medical Research Center, King Abdulaziz University, Jeddah 21859, Saudi Arabia; Vaccines and Immunotherapy Unit, King Fahd Medical Research Center, King Abdulaziz University, Jeddah 21859, Saudi Arabia; Vaccines and Immunotherapy Unit, King Fahd Medical Research Center, King Abdulaziz University, Jeddah 21859, Saudi Arabia; Vaccines and Immunotherapy Unit, King Fahd Medical Research Center, King Abdulaziz University, Jeddah 21859, Saudi Arabia; Vaccines and Immunotherapy Unit, King Fahd Medical Research Center, King Abdulaziz University, Jeddah 21859, Saudi Arabia; Department of Medical Laboratory Sciences, Faculty of Applied Medical Sciences, King Abdulaziz University, Jeddah 21859, Saudi Arabia; Vaccines and Immunotherapy Unit, King Fahd Medical Research Center, King Abdulaziz University, Jeddah 21859, Saudi Arabia; Vaccines and Immunotherapy Unit, King Fahd Medical Research Center, King Abdulaziz University, Jeddah 21859, Saudi Arabia; Vaccines and Immunotherapy Unit, King Fahd Medical Research Center, King Abdulaziz University, Jeddah 21859, Saudi Arabia; Department of Pharmaceutics, Faculty of Pharmacy, King Abdulaziz University, Jeddah 21859, Saudi Arabia; Vaccines and Immunotherapy Unit, King Fahd Medical Research Center, King Abdulaziz University, Jeddah 21859, Saudi Arabia; Vaccines and Immunotherapy Unit, King Fahd Medical Research Center, King Abdulaziz University, Jeddah 21859, Saudi Arabia; Department of Pharmacology and Toxicology, Faculty of Pharmacy, King Abdulaziz University, Jeddah 21859, Saudi Arabia; Vaccines and Immunotherapy Unit, King Fahd Medical Research Center, King Abdulaziz University, Jeddah 21859, Saudi Arabia; Vaccines and Immunotherapy Unit, King Fahd Medical Research Center, King Abdulaziz University, Jeddah 21859, Saudi Arabia; Vaccines and Immunotherapy Unit, King Fahd Medical Research Center, King Abdulaziz University, Jeddah 21859, Saudi Arabia; Department of Medical Laboratory Sciences, Faculty of Applied Medical Sciences, King Abdulaziz University, Jeddah 21859, Saudi Arabia; Vaccines and Immunotherapy Unit, King Fahd Medical Research Center, King Abdulaziz University, Jeddah 21859, Saudi Arabia; Department of Medical Laboratory Sciences, Faculty of Applied Medical Sciences, King Abdulaziz University, Jeddah 21859, Saudi Arabia; Department of Pharmaceutics, Faculty of Pharmacy, King Abdulaziz University, Jeddah 21859, Saudi Arabia; Vaccines and Immunotherapy Unit, King Fahd Medical Research Center, King Abdulaziz University, Jeddah 21859, Saudi Arabia; Department of Pharmacology and Toxicology, Faculty of Pharmacy, King Abdulaziz University, Jeddah 21859, Saudi Arabia; Vaccines and Immunotherapy Unit, King Fahd Medical Research Center, King Abdulaziz University, Jeddah 21859, Saudi Arabia; Department of Medical Laboratories Technology, College of Applied Medical Sciences, Jazan University, Jazan, Saudi Arabia; Department of Surgery, Faculty of Medicine, King Saud University, Riyadh 11451, Saudi Arabia; Vaccines and Immunotherapy Unit, King Fahd Medical Research Center, King Abdulaziz University, Jeddah 21859, Saudi Arabia; Department of Medical Microbiology and Parasitology, Faculty of Medicine, King Abdulaziz University, Jeddah 21859, Saudi Arabia

**Keywords:** ACE2, decoy receptors, DPP4, Fc-fusion proteins, MERS-CoV, immunoadhesins, receptor traps, SARS-CoV-2

## Abstract

The severe acute respiratory syndrome coronavirus-2 (SARS-CoV-2) and the Middle East respiratory syndrome coronavirus (MERS-CoV) are highly pathogenic human coronaviruses (CoVs). Anti-CoVs mAbs and vaccines may be effective, but the emergence of neutralization escape variants is inevitable. Angiotensin-converting enzyme 2 and dipeptidyl peptidase 4 enzyme are the getaway receptors for SARS-CoV-2 and MERS-CoV, respectively. Thus, we reformatted these receptors as Fc-fusion decoy receptors. Then, we tested them in parallel with anti-SARS-CoV (ab1-IgG) and anti-MERS-CoV (M336-IgG) mAbs against several variants using pseudovirus neutralization assay. The generated Fc-based decoy receptors exhibited a strong inhibitory effect against all pseudotyped CoVs. Results showed that although mAbs can be effective antiviral drugs, they might rapidly lose their efficacy against highly mutated viruses. We suggest that receptor traps can be engineered as Fc-fusion proteins for highly mutating viruses with known entry receptors, for a faster and effective therapeutic response even against virus harboring antibodies escape mutations.

## Teaser

Statement of Significance: Decoy receptors can be effective against infectious diseases once their gateway receptors are identified. In our report, we showed that ACE2-Fc and DPP4-Fc were able to inhibit pseudotyped SARS-CoV-2 and MERS-CoV variants. Therefore, antibody-like receptor traps can act as potential therapeutics for highly mutated viruses.

## INTRODUCTION

The Middle East respiratory syndrome (MERS-CoV) and the severe acute respiratory syndrome coronavirus-2 (SARS-CoV) are two highly pathogenic and deadly human coronaviruses (CoVs) [1]. Despite intensive vaccination campaigns and rigorous protection measures, SARS-CoV-2 is still spreading, causing millions of infections and thousands of deaths on a daily basis (WHO Coronavirus (COVID-19) Dashboard: https://covid19.who.int updated 12 June 2022). The efficiency of most of the available anti-SARS-CoV-2 monoclonal antibodies (mAbs) and vaccines is challenged by several aspects including the continuous variations in the viral genome and emergence of new variants including the “variants of concern” (VoCs) and “variants of interest” (VoIs) [2–5]. While the highly pathogenic respiratory virus MERS-CoV has affected 27 countries resulting in 858 deaths [6, 7], there are no licensed mAbs or vaccines for MERS-CoV with only a few that have entered clinical studies [8].

The entry of SARS-CoV-2 into the host cells depends on the binding of the receptor-binding domain (RBD) of the spike (S) protein to the host cellular membrane-bound angiotensin-converting enzyme 2 (ACE2) receptors [9, 10]. As a result, human recombinant ACE2 (hACE2) can be utilized as a decoy receptor to block the binding between the cell surface ACE2 receptor and SARS-CoV-2-RBD [11, 12]. Similarly, it has been previously reported that purified recombinant dipeptidyl peptidase 4 enzyme (DPP4) can inhibit MERS-CoV infection *in vitro* and in animal models [13, 14], as it utilizes DPP4 as a gateway receptor [15, 16]. Unlike mAbs and vaccines, variants escaping neutralization by such decoy receptors may attenuate the viruses indirectly by reducing binding to their native receptor.

Fc-fusion proteins (i.e. immunoadhesins), which are antibody-like molecules, offer several benefits as antiviral treatments. Despite the success of immunoadhesin-based drugs, which have been licensed in the USA and Europe for the treatment of a variety of disorders such as systemic lupus erythematosus and multiple sclerosis, no antiviral immunoadhesins have been approved [17–19]. The Fc effector functions allow for the recruitment of a wide variety of immune cells to facilitate the activation of the host antiviral immune response [20–22]. Moreover, they are characterized by improved half-life, binding affinity, long-acting time, serum stability and antiviral efficacy [23–26]. For example, fusing ACE2 with immunoglobin Fc domain (ACE2-Fc) enhanced its affinity, plasma stability, half-life and therapeutic efficacy compared to unfused recombinant receptor [22, 27, 28].

**Figure 1:**
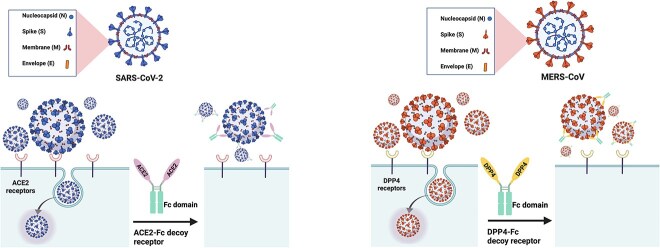
Generation of soluble recombinant human ACE2-Fc and DPP4-Fc protein as decoy receptors to SARS-CoV-2 and MERS-CoV, respectively. Recombinant human ACE2-Fc and DPP4-Fc proteins were generated by fusing the C terminus of the human ACE2 and DPP4 extracellular domains to a human IgG Fc domain as coronaviruses inhibitors.

In this study, we targeted these two highly pathogenic CoVs (SARS-CoV-2 and MERS-CoV) by developing Fc-fusion receptors decoy as a proof-of-concept of fast therapeutic option against important emerging viruses and their variants. Thus, we constructed IgG1-based ^WT^ACE2-Fc and ^Modified^ACE2-Fc [29] to be tested against all VoCs and one VOI (Kappa) of SARS-CoV-2 using pseudovirus neutralization assay (Fig. 1). The ^Modified^ACE2-Fc included in this report was optimized to have no intrinsic enzymatic activity, and to be able to bind and neutralize SARS-CoV-2 more effectively than ^WT^ACE2-Fc [29]. In addition, to enhance the affinity and neutralization potency of the recombinant DPP4 decoy receptor against several pseudotyped MERS-CoV variants, we fused it with IgG1-Fc domain (DPP4-Fc) (Fig. 1). The neutralizing activity of these receptor traps was directly compared with anti-SARS-CoV-2 and anti-MERS-CoV S proteins mAbs, ab1-IgG and M336-IgG, respectively [30, 31]. These Fc-fused receptor traps could be exploited as possible biotherapeutics for the current pandemic or future outbreaks of SARS-CoV-2 and MERS-CoV.

## MATERIALS AND METHODS

### Cloning protocol for mAbs and Fc-fusion proteins

The sequence of ^Wild-type(WT)^ACE2, ^Modified^ACE2, ^Beta^RBD, DPP4, M336 and ab1 were synthesized and codon-optimized for expression in CHO cells by Genscript (China). ^WT^ACE2 sequence includes the extracellular domains of human ACE2 (UniProt ID. Q9BYF1). ^Modified^ACE2 sequence includes the WT extracellular domains with five mutations [K31F/N33D/H34S/E35Q/H345L] as previously described [29]. ^Beta^RBD includes the WT sequence with three mutations K417N/N501Y/E484K found in the RBD of Beta VoC (UniPort ID. P0DTC2). Human DPP4 sequence includes the extracellular domains of human DPP4 (UniPort ID. P27487). These genes were cloned into the mammalian expression vector containing IL2 signal sequence pFUSE-hIgG1-Fc2 (Invivogen, Toulouse, France), using restriction sites EcoRV and BglII. The variable domains (V_H_ and V_L_) of M336 (PDB. 4XAK) and ab1 (PDB. 7MJL) were reformatted as IgG1s. For the V_H_, we used the mammalian expression vector containing IL2 signal sequence pFUSEss-CHIg-hG1 using restriction sites EcoRI and NheI (Invivogen, Toulouse, France). For the V_L_, we used the mammalian expression vector containing IL2 signal sequence pFUSE2ss-CLIg-hκ using restriction sites EcoRI and BsiWI (Invivogen, Toulouse, France). DNA used for transfection was prepared using GenElute HP Plasmid Maxiprep Kit (Sigma-Aldrich, Germany).

### Protein expression and purification

ExpiCHO-S cells (Gibco, Carlsbad, CA, US) were cultured in CD-CHO expression media (Gibco, Grand Island, New York, US) at 37 °C, 7.5% CO_2_ with shaking at 130 rpm. On the day of transfection, cells were prepared at ~6.0 × 10^6^ cells/mL at greater than 98% viability. For soluble expression of Fc-fusion proteins and mAbs, transient transfection was prepared as follows: 0.5 μg total of DNA per million cells per mL culture was mixed with OptiPRO SFM (Gibco, Grand Island, New York, US) and concurrently 1:3 of PEI-Pro (Polyplus-transfection) was mixed with OptiPRO SFM. The PEIpro complex was then added to the DNA complex by gentle pipetting and incubated static at room temperature for 15 min. The DNA complex was then added to CHO culture in a shake-flask and mixed by gentle swirling. The culture was incubated at 37 °C, 7.5% CO_2_ with shaking at 130 rpm for 4 to 6 h, after which the culture was diluted 1:2 (v/v) with CD-OptiCHO supplemented with EfficientFeed A and B (Gibco Carlsbad, CA, US), 0.4% Anti-Clumping Agent (Gibco, Grand Island, New York, US) and 8 mM GlutaMAX (Gibco, Grand Island, New York, US). The culture was incubated at 32 °C, 7.5% CO_2_, 130 rpm for 8 to 10 days then the supernatant was harvested by centrifugation and filtered using a 0.22-μm filter. The expressed products were purified using protein-A affinity chromatography. After loading the culture supernatant, the HiTrap MabSelect SuRe column (Cytiva, Marlborough, MA, US) was washed with PBS buffer and the proteins were eluted using 0.1 M glycine pH 3 and neutralized with 1 M Tris–HCl (pH 9.0). Eluted proteins were desalted into PBS using HiPrep 26/10 column (Cytiva, Marlborough, MA, US) and then filtered using a 0.22-μm filter. The concentration of eluted protein was determined at 280 nm using a Nanodrop 1000 spectrophotometer and the calculated extinction coefficient.

### SDS-PAGE and Western blot

The purity and the molecular mass of the protein of interest were determined by using SDS-PAGE and Western blot. For sodium dodecyl sulfate–polyacrylamide gel electrophoresis (SDS-PAGE) analysis, 8% SDS-PAGE was used with NuPAGE® LDS sample buffer (4×) as a loading buffer and NuPAGE® sample reducing agent (10×) as a denaturing buffer (Invitrogen, Carlsbad, CA, US), or water in the case where sample reducing was not required. The gel was run in 1% 2-Morpholinoethanesulphonic acid (MES) buffer for 35 min at 200 V, followed by staining with SimplyBlue SafeStain (Invitrogen, Carlsbad, CA, US) for 1 h at room temperature (RT). PageRuler Prestained Protein Ladder (Thermo Scientific, Vilnius, Lithuania) was used to estimate the molecular mass of the purified proteins. For Western blot, the purified proteins were separated on 8% SDS-PAGE followed by semi-dry transfer to polyvinylidene difluoride (PVDF) membrane for 40 min using Trans turbo blot (Bio-Rad, USA). After 1 h of blocking with 5% skim milk, the membrane was incubated with ECL Anti-Human IgG, Horseradish Peroxidase (HRP)-linked whole antibody (Cytiva, UK) at 1:2000 dilution overnight at 4°C. After 1 h, ECL substrate (Bio-Rad, US) was added and proteins were detected using iBright FL1500 (Invitrogen, Carlsbad, CA, US).

### ELISA on MERS-CoV and SARS-CoV S proteins

The sensitivity of the Fc-fusion proteins and mAbs toward recombinant soluble SARS-CoV S and MERS-CoV S proteins was measured using enzyme-linked immunosorbent assay (ELISA). In brief, a Nunc maxisorb plate was coated with 100 μL per well of recombinant MERS-CoV-S or SARS-S (SinoBiological) at 1 μg/mL in phosphate buffered saline (PBS) overnight at 4 °C. The wells were then washed three times with PBS–1% Tween 20 (PBS-T) and blocked with 300 μL per well of 5% skim milk, PBS (MPBS) for 1 h at RT. Also, 3 μg/mL of purified mAbs and immunoadhesins were blocked in 5% MPBS and incubated at RT for 1 h. Then, the ELISA plate was decanted and loaded with 200 μL/well of the blocked Fc-fusion proteins and mAbs and incubated at RT for 1 h, followed by 3× washes with PBS-T. Then, 200 μL of ECL Anti-Human IgG, HRP-linked whole antibody (Cytiva, UK) at 1:3000 dilution was added into each well and further incubated at RT for 1 h. Finally, the plate was washed 3× with PBS-T before adding 100 μL of 3,3′,5,5′ tetramethylbenzidine substrate (KPL, Gaithersburg, MD, USA) and incubated for 10 min. The reactions were terminated by adding 100 μL of 2 M H_2_SO_4_ per well. Synergy 2 Multi-Detection Microplate Reader (BioTek, Winooski, VT) was used at 450 nm to measure the absorbance.

### MERS-CoV spike protein alignment

A total of 1397 Middle East Respiratory Syndrome-related coronavirus (MERS-CoV) spike protein sequences were extracted from the National Center for Biotechnology Information (NCBI) viruses database [32]. The dataset was divided into two separate subsets according to the taxonomic classification of *Homo sapiens* and *Camelus dromedarius*. Next, a comparative analysis was performed on the sequences of the RBD in the human dataset, as well as in the dataset of dromedary camels. The Multiple Alignment using Fast Fourier Transform (MAFFT) multiple sequence alignment method [33] was utilized to align the RBD sequences. The variants that were previously identified within the RBD sequences were documented. The relevant accession numbers and references can be found in Appendix 1. In addition, the structure of the RBD sequence was extracted from the PDB database (4KQZ). The sites of the variations on the RBD structure were clarified using Chimera [34].

### Pseudoviruses generation

Generation of vesicular stomatitis virus (VSV)-pseudoviruses expressing SARS-CoV-2 and MERS-CoV S and neutralization assay were performed as previously described [35, 36]. Plasmids expressing codon-optimized truncated S protein (lacking the last 21 residues at the C-terminal) from ancestral Wuhan SARS-CoV-2 strain (GenBank accession nos. MN908947) and MERS-CoV (GenBank accession nos. KF958702) were previously generated [35]. Alpha, Beta, Gamma, Delta, Kappa and Omicron SARS-CoV-2-S VoCs were generated from pcDNA™3.1(+) (Invitrogen, Carlsbad, CA, US) plasmid expressing ancestral Wuhan SARS-CoV-2 strain as a template for site-directed mutagenesis using In-Fusion HD Cloning plus (Takara Bio) to introduce all indicated mutations. All constructs were confirmed by sequencing. For the generation of pseudoviruses, constructs were then transfected into BHK21/WI-2 cells using Lipofectamine 2000 (Invitrogen) and infected with VSVΔG/G*-luciferase 24 h later. One hour after infection and incubated at 37 °C, virus inoculum was removed, cells were washed and cells were incubated in Dulbecco's Modified Eagle Medium (DMEM) (Gibco, Grand Island, New York, US) containing rabbit polyclonal anti-VSV-G antibody at 1:1000 for 24 h at 37 °C and 5% CO_2_. The supernatants containing pseudoviruses were then collected 24 h later and titrated as described previously [35].

### Pseudotyped viral vector neutralization assay

The pseudovirus neutralization assay was performed as previously described [35]. Briefly, serially diluted immunoadhesins and mAbs starting from 100 μg/mL to 0.001 μg/mL in DMEM with 5% Fetal Bovine Serum (FBS) were mixed with an equal volume of media containing VSV pseudoviruses that yield 1 × 10^5^ relative luciferase unit (RLU) and incubated for 1 h at 37 °C in a 5% CO_2_ incubator. Then, 100 μL of the mixture was transferred onto Vero E6 cell monolayers seeded in a 96-well white plate with a clear bottom and incubated at 37 °C and 5% CO_2_ for 24 h. On the next day, cells were washed with PBS and lysed with 1× lysis buffer (20 mM tricin, 100 μM EDTA, 1.07 mM MgCO_3_, 2.67 mM MgSO_4_, 17 mM DTT, 1% Triton X-100, 1% Tween 20, 0.4% Tergitol and 0.1% Antifoam dissolved into dH_2_O). Luciferase activity was measured as a RLU by adding 50 μL luciferase substrate (0.03 mg/mL D-Luciferin dissolved into dH_2_O) and 100 μL luciferase buffer (1 mM adenosine 5'-triphosphate (ATP), 15 mM MgSO_4_, 1 mM Dithiothreitol (DTT) and 15 mM KPO_4_; the volume was completed with sterile dH_2_O) to each well, respectively. Cell-only control (CC) and virus control (VC) were included in each run. Luminescence was measured using BioTek Synergy 2 microplate reader (BioTek). The inhibition of luciferase activity by each dilution of each protein was determined as follows: 100 − [(average RLU from each dilution − average RLU from CC)/(average RLU from VC − average RLU from CC) × 100]. Then, neutralization titers were computed as IC_50_ using a four-parameter logistic (4PL) curve in GraphPad Prism V9 software (GraphPad Co., San Diego, CA, USA) and calculated as 100 − [(mean RLU from each sample − mean RLU from CC)/(mean RLU from VC − mean RLU from CC) × 100].

## RESULTS

### Anti-SARS-CoV-2 and anti-MERS-CoV mAbs and immunoadhesins

**Figure 2:**
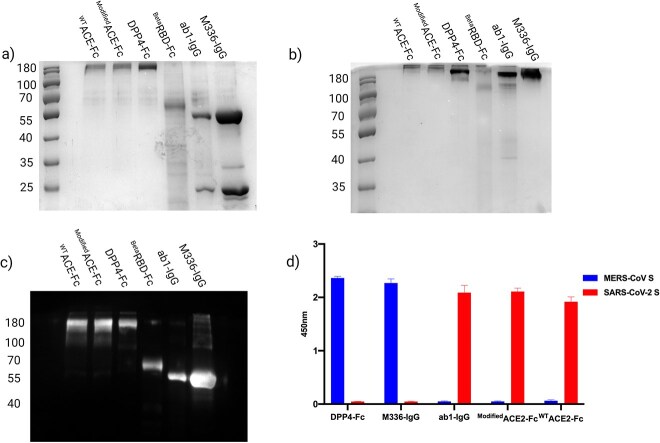
Purified recombinant ^WT^ACE2-Fc, ^Modified^ACE2-Fc, DPP4-Fc, ^Beta^RBD-Fc, IgG ab1-IgG and M336-IgG. (A) The stained SDS-PAGE gel under reducing conditions shows ^WT^ACE2-Fc, ^Modified^ACE2-Fc and DPP4-Fc at ~100 kDa, ^Beta^RBD-Fc at ~ 60 kDa, and IgGs light chain at ~25 kDa and heavy chain at ~50 kDa. (B) Stained gel under nonreducing conditions shows ^WT^ACE2-Fc, ^Modified^ACE2-Fc and DPP4-Fc at ~200 kDa, ^Beta^RBD-Fc at ~120 kDa and IgGs at ~ 150 kDa. (C) Western blot stained with anti-human IgG-HRP polyclonal antibody confirmed that Fc-fusion proteins and IgGs mAbs were efficiently expressed and purified. (D) ELISA for anti-SARS-CoV-2 and anti-MERS-CoV Fc-fusion proteins and IgGs. ELISA plate was coated with 1 μg/mL recombinant SARS-CoV-S and MERS-CoV-S proteins, then probed with 3 μg/mL of the anti-SARS-CoV-2 and anti-MERS-CoV Fc-fusion proteins and IgGs mAbs. Binding was detected using HRP-conjugated anti-human IgG polyclonal antibody. Error bars represent standard deviations.

The expression of M336-IgG, ab1-IgG and ^WT^ACE2-Fc, ^Modified^ACE2-Fc, ^Beta^RBD-Fc and DPP4-Fc proteins was performed in ExpiCHO-S cells using PEI-mediated transfection method under mild hypothermic conditions and purified by protein-A affinity chromatography. The average yields of the pure recombinant proteins were 1 mg from 50 mL transfection. SDS-PAGE and Western blot analysis showed pure protein bands at the calculated molecular weight under reducing (monomeric form) and nonreducing (dimeric form) conditions (Fig. 2A–C). The specificity of the antibodies and immunoadhesins toward recombinant soluble SARS-CoV-S and MERS-CoV-S protein was also confirmed by ELISA (Fig. 2D).

### *In vitro* neutralization of SARS-CoV-2 VoCs

**Figure 3:**
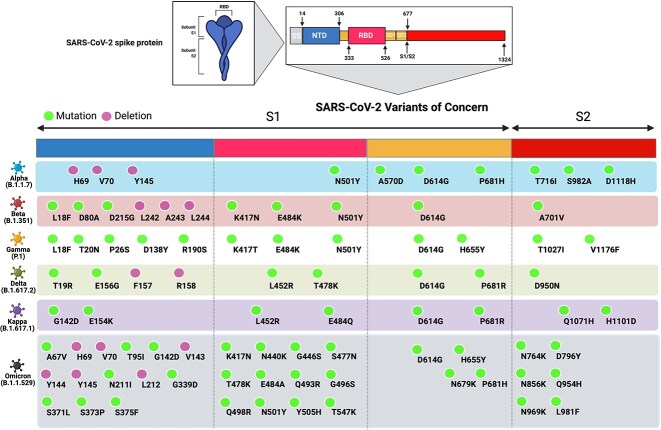
Schematic representation of SARS-CoV-2 VoCs amino acid deletions/mutations in their spike (S) proteins, which were utilized to construct the pseudoviruses in this study. The S protein consists of a secretion signal (SS); N-terminal domain (NTD); receptor-binding domain (RBD); subdomain 1 (SD1); subdomain 2 (SD2); protease cleavage site (S1/S2). Amino acid deletions and substitutions in each SARS-CoV-2 VoC are indicated relative to the reference Wuhan sequence.

**Figure 4:**
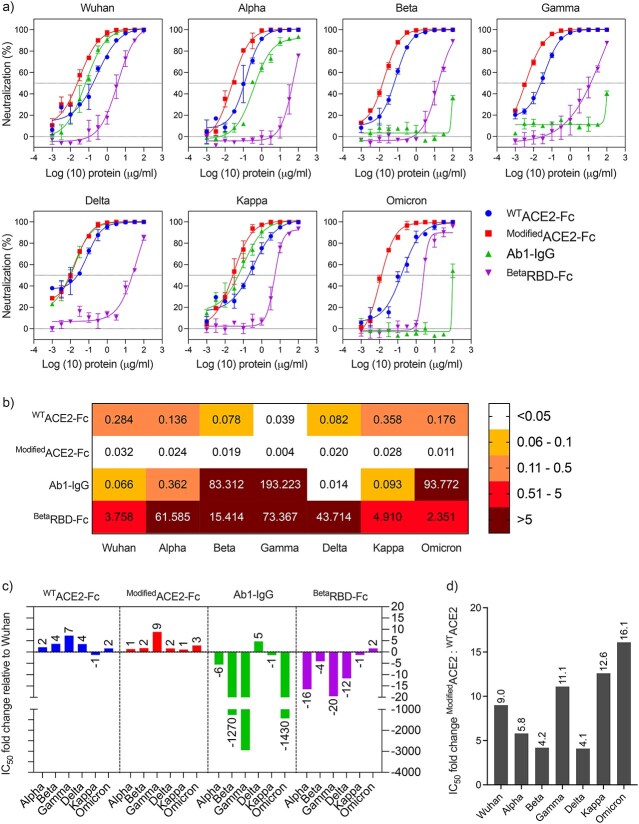
Neutralization of SARS-CoV-2 using receptor traps and mAb. ^WT^ACE2-Fc, ^Modified^ACE2-Fc, ^Beta^RBD-Fc and ab1-IgG were tested against ancestral Wuhan strain and Alpha, Beta, Gamma, Delta, Kappa and Omicron variants. (A) Neutralization activity of varying concentrations of ^WT^ACE2-Fc, ^Modified^ACE2-Fc, ab1-IgG, ^Beta^RBD-Fc against the different SARS-CoV-2 pseudoviruses. (B) Heat map showing the IC_50_ values of ^WT^ACE2-Fc, ^Modified^ACE2-Fc, ab1-IgG, ^Beta^RBD-Fc against the different SARS-CoV-2 pseudoviruses. (C) Neutralization fold change of ^WT^ACE2-Fc, ^Modified^ACE2-Fc, ab1-IgG, ^Beta^RBD-Fc against each SARS-CoV-2 variant relative to the ancestral Wuhan SARS-CoV-2 strain. (D) Neutralization potencies of the ^modified^ACE2-Fc decoy as fold change relative to the ^WT^ACE2-Fc for ancestral Wuhan SARS-CoV-2 strain and other variants. Data are shown as the mean ± SD of duplicates. Dotted lines represent cutoffs of the assays.

We generated a panel of VSV-based pseudoviruses to express the S protein of the ancestral Wuhan strain in addition to the following SARS-CoV-2 VoCs including Alpha (B.1.1.7), Beta (B.1.351), Gamma (P.1), Delta (B.1.617.2) and Omicron (B.1.1.529) as well as VoI Kappa (B.1.617.1) as shown in Fig. 3. The neutralization capacity of the developed receptor traps and ab-1 mAb showed that ^WT^ACE2-Fc and ^Modified^ACE2-Fc were able to inhibit all the variants at ng/mL concentrations (Fig. 4A–B). Both traps ^WT^ACE2-Fc and ^Modified^ACE2-Fc showed strong inhibition against the different variants in which we did not observe any loss of potency compared to ab-1 mAb or ^Beta^RBD-Fc (Fig. 4C). Superior IC_50_ values were observed with ^Modified^ACE2-Fc compared to ^WT^ACE2-Fc; in some instances, it reached up to 16-fold higher viral inhibition as depicted in Fig. 4D. On the other hand, ab-1 IgG was only able to neutralize Wuhan, Alpha, Delta and Kappa pseudoviruses (Fig. 4A–C), while ^Beta^RBD-Fc was able to cause modest inhibition by competition to the native ACE2, although the degree of inhibition was dependent on the variant affinity to the ACE2 receptor (Fig. 4A–C).

### *In vitro* neutralization of MERS-CoV pseudoviruses

**Figure 5:**
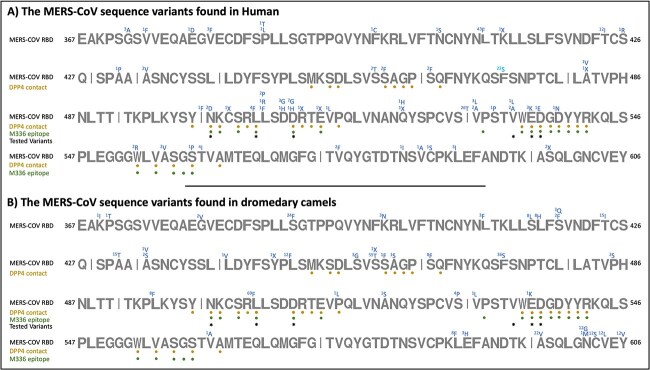
A schematic illustration of the MERS-CoV RBD sequences in humans (A) and camels (B). The representation shows the DPP4 and M336-IgG epitopes as well as the pseudovirus variants that are included in this report. Variants found from the multiple sequence alignment of the RBD sequences with their number of occurrences indicated at the top of the residues.

The number of extracted spike protein sequences from the NCBI virus database totaled 1397. The two datasets contain 572 and 825 sequences representing the MERS-CoV S protein found in humans and dromedary camels, respectively. After the extraction of the RBD regions from the S protein sequences, the numbers were decreased to 543 and 577 sequences, respectively, since some of the S proteins were partially sequenced. The alignment revealed several RBD sequence variants from isolates obtained from humans (Fig. 5A), wherein most of these variants have mutations in residues involved in the RBD binding to the DPP4 receptor (Fig. 5A).

From the retrieved 543 ^RBD^MERS-CoV sequences found in humans, variants with high recurrence were L411F^(43)^, F473S^(22)^, T424I^(12)^ and I529T^(26)^ (Fig. 5A). Moreover, a small number of MERS-CoV variants found in human showed that the virus can harbor more than one mutation (Table 1). In comparison, from the retrieved 577 MERS-CoV S RBD sequences in dromedary camels, variants with high number of recurrences were S390F^(24)^, T424I^(15)^, A431T^(15)^, L450F^(12)^, S459T^(55)^, F473S^(36)^, L506F^(69)^, A597V^(22)^, N602G^(12)^, C603S^(12)^, V604L^(12)^ and Y606V^(12)^ (Fig. 5B). Noteworthy, even if they represent few cases, some MERS-CoV variants found in dromedary camels showed that the virus can harbor more than one mutation (Table 2).

**Table 1:** MERS-CoV variants found in human represented in one sequence and their number of recurrences

**The variants**	**Number of appearances**
(E379D) and (V514L)	1
(L411F) and (C585S)	1
(L411F) and (S559P)	1
(L411F) and (I500F)	1
(A431V) and (A482V)	1
(F473S) and (E513X)	1
(V534L) and (S598X)	2

**Table 2:** MERS-CoV variants found in dromedary camels represented in one sequence and their number of recurrences

**The variants**	**Number of reappearances**
(P370T) and (A431T)	1
(G380V) and (A431T)	2
(A431T) and (N602M)	1
(S390F) and (A597V)	3
(S390F), (A434V) and (A597V)	1
(S390F), (L450F) and (A597V)	12
(S390F), (P515L) and (A597V)	1
(S390F), (S528P) and (A597V)	4
(S390F), (V561A) and (A597V)	1
(S390F) and (S459T)	1
(S419F) and (L443V)	1
(S419F) and (S465F)	1
(S459T) and (E536K)	1
(S416L) and (F473S)	5
(K400N), (S416L) and (F473S)	1
(K400N) and (S416L)	1
(L417H) and (F473S)	8
(L495F) and (L588F)	8
(L506F), (N602G), (C603S), (V604L), (Y606V)	12

Among these mutations, we selected RBD’s mutations that are located within the DPP4 and M336 binding domain to construct a panel of VSV-based pseudoviruses (Table 3). These key mutations were found in the RBD of different MERS-CoV strains isolated from various locations and at different periods as the virus evolved during its spread in humans. These mutations were included to test if these variants can escape DPP4-Fc or M336-IgG1. However, we were not able to rescue ^E536K^MERS-CoV and ^D537E^MERS-CoV pseudoviruses during the generation step. MERS-CoV acidic residues E536 and D537 are negatively charged residues that interact with the DPP4 positively charged K267 and R336 residues [15]. The charge–charge association with DPP4 would be disrupted by mutations in the MERS-CoV RBD residues E536 and D537, which would significantly reduce binding and viral entry levels.

**Table 3:** MERS-CoV variants generated for the pseudoviruses assay included in this study

**The variants**	**Number of appearances in human**	**Number of appearances in dromedary camels**
N501D	2	0
L506F	4	69
D510G	7	0
D510H	1	0
V534L	1	0
E536K*	3	1
D537E*	1	0

^*^Was not included in the pseudovirus assay because pseudovirus could not be rescued

**Figure 6:**
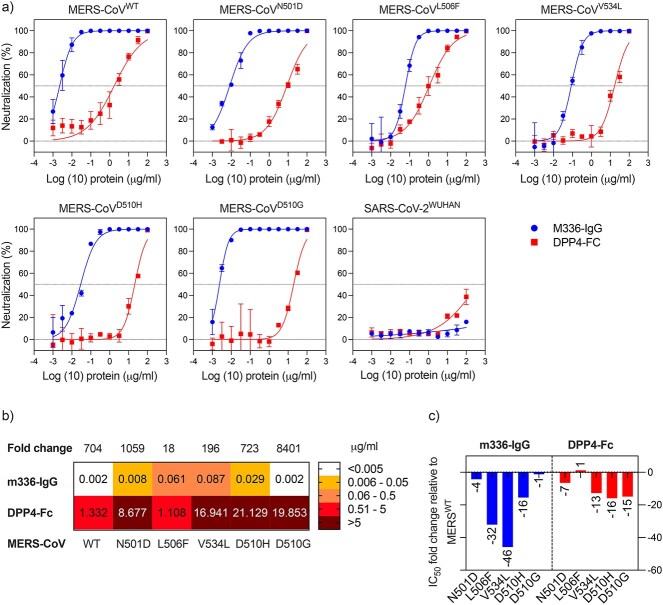
Neutralization of MERS-CoV pseudotyped virus using receptor trap and mAb. The inhibitory effect of varying concentrations of DPP4-Fc and M336-IgG was tested against MERS-CoV pseudovirus. IC_50_ values of DPP4-Fc and M336-IgG are shown. Data are shown as the mean ± SD of duplicates. Dotted lines represent cutoffs of the assays.

Superior IC_50_ values were observed with M336 IgG1 against all the MERS-CoV pseudotyped variants (Fig. 6). Although it is a potent anti-MERS-CoV mAb, the inhibition was inconsistent among the different variants (Fig. 6). While DPP4-Fc was able to inhibit all the pseudotyped variants, yet it was significantly less potent than M336 IgG1 (Fig. 6). For instance, DPP4-Fc was able to neutralize the pseudotyped ^WT^MERS-CoV, albeit less potent than the anti-MERS-CoV mAb M336-IgG (IC_50_: 1.332 vs. 0.002). Moreover, unlike M336-IgG, any single substitution within the RBD–DPP4 contact residues would negatively affect the decoy receptor potency. For example, DPP4-Fc was able to show modest neutralization against the pseudotyped ^D510G^MERS-CoV compared to M336-IgG (IC_50_: 19.853 vs. 0.002)**.**

## DISCUSSION

The S protein protruding from the surface of CoVs facilitates viral attachment and entry into host cells via its RBD through binding to their cell receptors (i.e. ACE2 for SARS-CoV-2 and DPP4 for MERS-CoV). Of note, SARS-CoV-2 VoCs contain different mutations in the whole S protein including the RBD, which is the primary target of many neutralizing antibodies, in addition to other mutations in other viral proteins [37–40]. Some of these mutations resulted in higher transmissibility, disease severity, risk of eluding detection tests, and ability to reduce vaccines and mAbs efficacy [41, 42]. Therefore, we generated VSV-based pseudoviruses to evaluate and measure the neutralizing abilities of the Fc-based receptor traps that we generated against these two highly pathogenic CoVs [35].

Antibody therapies based on human IgG1, IgG2 or IgG4 are widely employed in the treatment of cancer, immunology and infectious diseases [43]. Selecting a suitable IgG isotype is critical not only for the efficacy of the mAbs but also for their biophysical characteristics and manufacturability, which may accelerate and minimize the cost of process development and manufacturing. The vast majority of commercialized antibody-based biologics are human IgG1, indicating its superior developability over other IgG isotypes [44]. For instance, IgG1-based mAbs exhibit higher solubility and lower or equivalent viscosity to IgG2 or IgG4 mAbs with matching variable regions [43]. IgG1 remains monomeric in acidic pH, while IgG2 and IgG4 antibodies are more susceptible to form aggregates [45]. This is crucial because during biomanufacturing, antibodies are frequently exposed to low pH conditions during protein-A-based purification and viral inactivation. Moreover, IgG1 accounts for the majority of the approved high-concentration antibody products (HCAPs), which are usually ≥100 mg/mL. HCAPs have been extremely popular and successful because of their significant benefits of allowing self-administration and minimizing hospitalization, shipping and storage costs [46]. Thus, IgG1-Fc was used in our design decoy receptor since it has a higher average stability compared to other IgG isotypes, and they are well suited for high concentration antibody formulations and biomanufacturing design.

Different evolution platforms have been used to generate high-affinity, enzymatic deactivated ACE2-Fc decoy receptors to have antibody-like molecules with exclusive antiviral activity [47]. In this work, we tested ^WT^ACE2-Fc and ^Modified^ACE2-Fc, which has been engineered by Glasgow et al. [29], to be tested against several SARS-CoV-2 variants including VoCs. Glasgow et al. generated an affinity-matured and enzymatically inactive recombinant ACE2-Fc modified immunoadhesin [K31F, N33D, H34S, E35Q, H345L ACE2(740)-Fc], which efficiently neutralized pseudotyped and live SARS-CoV-2 viruses [29]. For comparative assessment, we included in our study a fully human anti-SARS-CoV-2 ab1-IgG as it strongly competes with ACE2 for binding to RBD and potently neutralized SARS-CoV-2 [30]. In addition, ^Beta^RBD-Fc was also included as a potential competitive inhibitor of binding between SARS-CoV-2 and ACE2. Similarly, to suppress MERS-CoV cell entry, we generated a DPP4-Fc construct to be an antibody-like molecule because of the formation of disulfide linkage in the Fc domain. As a control, we included anti-MERS-CoV M336-IgG mAb that showed high neutralizing activity against both pseudotyped and authentic MERS-CoV *in vitro* and *in vivo* [31, 48, 49].

While anti-^RBD^CoV mAbs are effective, there is a high possibility of emergence of neutralization escape variants, as seen with anti-SARS-CoV-2 ab-1 IgG mAb (Fig. 4). Unlike ab-1 IgG, ^WT^ACE2-Fc and ^Modified^ACE2-Fc displayed a strong antiviral activity even against Beta, Gamma and Omicron VoCs, which are known for their strong immune escape abilities compared to other variants [50].

Alpha, Beta and Gamma VoCs harbor mutations (E484K, N501Y, K417N/T) within their RBD, (Fig. 3), which improved their binding affinities to the ACE2 receptor and enhanced their resistance toward neutralizing antibodies compared to the ^Wuhan^SARS-CoV-2 (Fig. 4C). E484K and N501Y mutations influence ACE2 binding by causing particular rearrangements at the S protein–ACE2 interface and allowing the formation of intermolecular interactions that enhance the RBD/ACE2 interaction [51]. For instance, N501Y mutation in Alpha, Beta and Gamma VoCs enables forming π–π stacking interaction with ACE2-Y41, while E484K mutation in Beta and Gamma VoCs allows the formation of H-bond electrostatic interaction between RBD’s Q493 and H34 within ACE2. On the other hand, K417T/N, which are neutralizing antibody escape mutations, would abolish the salt bridge with D30 of ACE2 and therefore decrease the RBD’s affinity to ACE2 [51]. Although K417N/T mutations reduce Beta and Gamma variants’ affinity for ACE2, the existence of ACE2 affinity enhancing mutations, N501Y/E484K, represents a compensatory mechanism that strengthens the intermolecular interactions with the ACE2 receptor.

Similar to other reports [52, 53], Ab1-IgG showed effective, yet less neutralization activity against Alpha compared to Wuhan pseudoviruses because Alpha variant has N501Y mutation, which constitutes part of the ab1-IgG epitope. Beta and Gamma pseudotyped VoCs completely depleted the neutralization activity of ab1-IgG because they harbor the K417N/T mutations.

The binding affinity analysis using full S protein showed that Gamma VoC had the strongest affinity toward ACE2, followed by Beta and then Alpha VoCs [51, 54]. Although one RBD mutation differentiates between Beta and Gamma VoCs, K417N mutation reduced the Beta variant affinity toward ACE2 compared to K417T mutation in Gamma variant. Consistent with the binding affinity analysis, ^Modified^ACE2-Fc and ^WT^ACE2-Fc constructs exhibited more potent antiviral activity against Gamma and Beta compared to Alpha and Wuhan (Fig. 4B).

Delta’s RBD binds to hACE2 with binding affinity slightly higher than ^WT^RBD, and significantly less than Alpha, Beta and Gamma VoCs [55, 56]. However, ^Modified^ACE2-Fc and ^WT^ACE2-Fc constructs displayed comparable IC_50_ values for Delta and Beta pseudoviruses (Fig. 4) despite the lower binding affinity of ACE2 to the RBD of Delta compared to Beta variant. This could be related to other mutations within the S protein that contribute to ACE2 binding. Two Delta RBD mutations were observed, L452R and T478K, which are located outside the RBD/ACE2 binding interface and are not involved in ACE2 interaction. By providing a positive charge at position 452, the L452R RBD mutation slightly improves RBD/ACE2 binding affinity by increasing the electrostatic complementarity effects between the RBD and the highly negative charge residue, E35 of the ACE2 receptor [51, 55]. Although the binding affinity of Delta’s RBD with hACE2 does not differ significantly from that of ^WT^RBD, the Delta variant exhibits increased transmissibility and a shorter incubation period, indicating that there are other variables influencing viral transmission.

^Modified^ACE2-Fc and ^WT^ACE2-Fc constructs displayed less potent IC_50_ for ancestral Wuhan strain, and Kappa VoI (Fig. 4B), which might be due to their lower affinities to the ACE2 receptor. Compared to Wuhan strain, Kappa variant showed no significant increase in the binding affinity toward ACE2 [57]. The E484Q mutation in the Kappa variant disrupts the Van der Waals’ interactions between residue E484 and residue ACE2-K31, which presumably leads to weaker interactions with the receptor [57]. However, as previously described, the increased electrostatic complementarity provided by the L452R mutation may represent a compensation mechanism for restoring the ACE2 binding affinity. Unlike the Kappa VoI, the Delta VoC demonstrated moderate enhancement in ACE2 binding affinity because it preserves the E484 electrostatic interaction with ACE2-K31 and contains L452R substitution [51, 57]. As previously shown [57], ab1-IgG also displayed a significant increase in the neutralization potency for Delta VoC compared to Kappa VoI and ancestral Wuhan strain (Fig. 4B–C), which could be attributed to the improved electrostatic interactions provided by mutations found in Delta VoC.

When compared to other SARS2-CoV-2 variations, Omicron has the highest number of mutations, with 15 amino acid substitutions in its RBD (Fig. 3). Mutations among these RBD-specific residues, K417, T478, E484 and N501, were common in Alpha, Beta, Gamma and Delta VoCs, whereas the rest of the amino acid substitutions were novel. Similar to the Beta and Gamma VoCs, Omicron showed strong resistance against the RBD-specific ab1-IgG most likely due to the presence of K417N, T478K and N501Y mutations, which located within ab1-IgG paratopes [52, 56]. Previous reports showed that Omicron variant contains mutations in nearly all the sites that were identified in prior VoCs. Some of these mutations such as K417N, E484A and Y505H reduced the ^Omicron^RBD binding affinity toward ACE2 [55, 58]. The E484 in ^Wuhan^RBD forms Van der Waals’ interaction with ACE2-K31, and the substitution to A484 disrupts this interaction because of its short side chain. Moreover, Y505H mutation decreases the binding of Omicron RBD with hACE2 by forming fewer interactions with ACE2 [59]. However, other amino acid substitutions, such as G339D, N440K, S447N and Q498R, enable Omicron’s RBD to restore ACE2 binding efficiency by forming several H-bonds, salt-bridge and π–π stacking interactions [55, 58, 60]. For instance, S477N mutation confers two new H-bonds strengthening the ACE2/RBD interaction, and Q493R and Q498R substitutions enable the formation of new salt bridges with ACE2-E35 and -D38, respectively, instead of the H-bonds. Previous studies also suggested that the Omicron mutations K417N, E484A, Q493K and Q493R could be responsible for immune escape after mAbs treatment [61–66].

^Modified^ACE2-Fc and ^WT^ACE2-Fc constructs exhibited comparable neutralization potency against SARS-CoV-2 VoCs Alpha and Omicron (Fig. 4A–C), although Omicron has a lower binding affinity compared to Alpha. Despite the large number of amino acid substitutions on the surface of the S protein, including the RBD, Omicron evolved to increase neutralizing antibody evasion while maintaining its ability to bind strongly to hACE2, which explains its high transmissibility. Here, we used ^Beta^RBD-Fc construct in our performed neutralization assay as a control to confirm the expression of ACE2 on the cell surface of VeroE6 cells. Recombinant purified ^Beta^RBD-Fc was able to bind to native ACE2 cell surface receptors, which means that it can be utilized as a vaccine candidate to produce neutralizing antibodies against the notorious Beta VoCs. The presence of the Fc domain is important because it has been previously shown that it serves as an immunopotentiator to promote the immunogenicity of the vaccine [67].

^Beta^RBD-Fc IC_50_ values were dependent on the competition of the full S of the pseudovirus and the ^Beta^RBD-Fc to the native ACE2. ^Beta^RBD-Fc showed stronger viral inhibition against ancestral Wuhan strain, Kappa VoI and Omicron VoC, compared to other VoCs (Fig. 4B–C). On the other hand, ^Beta^RBD-Fc showed less inhibition against Alpha and Gamma VoC, which might be due to their stronger affinity to ACE2 compared to the Beta variant [61]. ^Beta^RBD-Fc showed weaker inhibition of Delta variant, which is different from what we expected, since ^Beta^RBD has a higher affinity to ACE2 receptor, compared to ^Delta^RBD [55]. This might be related to other amino acid substitutions in the Delta S protein outside the RBD domain that could contribute to the receptor binding.

Using DPP4 as a decoy receptor would be vital for a fast preventive response to MERS-CoV. The MERS-CoV neutralization assay outcome indicates that inhibition of MERS-CoV/DPP4 cell receptor interaction via DPP4-Fc can be a promising anti-MERS candidate. Key mutations, such as L506F, V534A and D510G, among others, were found in the RBD of different MERS-CoV strains isolated from various locations and at different periods as the virus evolved during its spread in humans [68]. When compared to the ^WT^RBD, ^RBD^MERS-CoV mutations like D510G, L506F and E536K show reduced affinity for their gateway receptor, DPP4, suggesting that MERS-CoV adaptation during spreading in human may be motivated by escaping neutralizing antibodies instead of evolution for a higher binding affinity toward the DPP4 receptor [69]. For instance, M336-IgG1, which is able to compete with the DPP4 receptor for binding to the MERS-CoV RBD with a strong affinity at certain amino acid positions [31] that are common with other identified variants. There is some evidence that mutations in the L506, D510 and E536 residues of the ^RBD^MERS-CoV increase MERS-CoV resistance to neutralizing antibodies, such as M336 [31, 70]*.*

Previously, it was found that there is a hydrophobic core consisting of the MERS-CoV RBD residues L506, W553 and V555, along with the DPP4 residues L294 and L295. A single mutation in the L506 residue is expected to change the hydrophobic core, reducing both binding and viral entry efficiency significantly [15]. However, the conserved hydrophobic L506F mutation may not substantially alter the hydrophobic core; therefore, it has minimal effects on RBD interaction with human DPP4 [71]*.* According to our study, the concentration of DPP4-Fc that was required for 50% inhibition of MERS-CoV^L506F^ was lower by one-fold compared to the WT (Fig. 6). While a 32-fold decrease in the neutralization potency of M336-IgG against MERS-CoV^L506F^ was detected.

MERS-CoV negatively charged RBD residue D510 forms a salt-bridge interaction with DPP4 positively charged residue R317 [69]. We showed that D501H/G mutation reduced the DPP4-Fc neutralization by at least 15-fold (Fig. 6) since single mutation in the D510 residue will interrupt the salt-bridge interaction with the DPP4 residue R317 hydrophobic core, reducing both binding and viral entry to variable degrees [15]*.*

A slight reduction in the DPP4-Fc and M336-IgG performance against MERS-CoV^N501D^ was observed in our assessment (Fig. 6). The binding interface between ^RBD^MERS-CoV and DPP4 reveals a range of hydrophilic residues that result in polar interactions. The main mediators of these interactions are side chain residues, such as RBD N501 [72].

Mutations in ^RBD^MERS-CoV residues L506, D510 and V534 can produce viral variants resistant to neutralizing antibodies [69]. This was evident in our study as a significant reduction in the M336-IgG and DPP4-Fc performance was observed. For instance, M336-IgG was 16-fold less potent against MERS-CoV^D510H^ pseudovirus compared to ^WT^MERS-CoV. However, a slight reduction against MERS-CoV^D510G^ was detected. A further assessment is needed to understand the effect of amino acid substitution at position 510 within the virus RBD. Moreover, a significant reduction in the potency of M336-IgG and DPP4-Fc was observed against MERS-CoV^V534L^ pseudovirus.

The MERS-CoV neutralization assay outcome indicates that inhibition of MERS-CoV/DPP4 cell receptor interaction via DPP4-Fc decoy receptor can be a promising anti-MERS candidate. However, further optimization strategies are required to have an enzymatically inactivated, affinity-matured DPP4-Fc decoy receptor, similar to what has been performed on ACE2-Fc [47]. For instance, the dipeptidyl peptidase activity of the DPP4 can be abolished by introducing single-point mutations such as Glu-205-Lys and Glu-206-Leu [73]. The role of DPP4-Fc in preventing viral entrance is not directly related to their enzymatic activity. Thus, it is crucial to introduce single-point mutations such as Glu-205-Lys and Glu-206-Leu to inactivate the unnecessary dipeptidyl peptidase activity and obtain an exclusive antiviral effect. In addition, other modifications such as multivalent Fc traps [74] or bispecific DPP4 with a flexible linker instead of the Fc could be developed and tested.

Several reports showed the efficacy of ACE2-like antibodies against pseudo- and live viruses, which can bind tightly to the spike protein and effectively block viral infection [47, 75]. DPP4-Fc decoy receptor, on the other hand, has received little attention, with only a few articles discussing its potential as an anti-MERS-CoV agent [76]. Recombinant ACE2-Fc and DPP4-Fc are still under preclinical investigations, yet the studies’ outcomes would pave the way for initiating clinical studies.

## CONCLUSION

In theory, Fc-fusion decoy receptors can be used against any infectious disease once their receptors are identified. They can be used as a quick therapeutic option for future viral outbreaks that use specific receptors for entry without the risk of virus mutational escape. These engineered antibody-like biomolecules would have the ability to act as prophylactic agents for those who are at high risk of virus infections such as healthcare workers, especially in the absence of effective mAbs and vaccines against new variants.

However, Fc-fusion proteins might produce anti-drug antibodies, which would negatively affect the safety and therapeutic efficacy [77, 78]. Patients might suffer detrimental effects if they develop immunological cross-reactivity against endogenous ACE2 or DPP4 after receiving recombinant Fc-fusion receptor traps treatment. As a result, physicians can employ well-validated approaches to identify and eliminate any unwanted immunological reactions during the course of ACE2-Fc or DPP4-Fc therapy. Also to avoid such effects, ACE-Fc and DPP4 decoy receptor could be further improved to include only the fragment of the protein that interacts with the SARS-CoV-2 and MERS-CoV RBDs. Such an approach has been used against HIV where only a short peptide of the CD4 protein was used as a receptor decoy to HIV entry to cells while maintaining efficacy and improving the safety profile [79].

A further issue is that the presence of Fc may boost complement and cytokine responses, worsening the patient’s state and contributing to infection progression. Since IgG4 has a low affinity toward Fc receptors, it can be employed as a backbone; hence, it is preferred when a host effector function is unwanted or superfluous. However, the stability of IgG4 antibodies is lower than IgG1 counterparts, making them less appealing as biologics [47]. As a mitigation strategy, mutations in the second constant domain CH2 of IgG1 such as LALA-S267K and LALA-P329A would diminish or abolish effector function while maintaining ^WT^IgG1 biophysical properties [80].

Nonetheless, engineering, developing and stockpiling immunoadhesins as receptor traps could play a vital role as a fast preventive response to emerging and reemerging CoVs, allowing future epidemics to be contained.

## Supplementary Material

Appendix_1_tbad030

## Data Availability

The data from this study are available from the corresponding author upon reasonable request.

## ABBREVIATIONS

ACE2, angiotensin-converting enzyme 2; CoVs, coronaviruses; DPP4, dipeptidyl peptidase 4 enzyme; IC_50_, half maximal inhibitory concentration; MERS-CoV, Middle East respiratory syndrome coronavirus; RBD, receptor-binding domain; SARS-CoV-2, severe acute respiratory syndrome coronavirus-2; S, spike protein; VoCs, variants of concern; VoIs, variants of interest.
