# Supplementary material for: ACE2-Fc and DPP4-Fc decoy receptors against SARS-CoV-2 and MERS-CoV variants: a quick therapeutic option for current and future coronaviruses outbreaks
Source: Antib Ther. 2023 Dec 12;7(1):53–66. doi: 10.1093/abt/tbad030 (PMC10873275; doi:10.1093/abt/tbad030)
Supplement: Appendix_1_tbad030 [file appendix_1_tbad030.docx]

**Human’s MERS-CoV Spike**

| Accession | Submitters | Release_Date | Molecule_type | Length | Sequence_Type | Protein | Country | Host | Collection_Date |
| --- | --- | --- | --- | --- | --- | --- | --- | --- | --- |
| AKS48062.1 | Kim,S.S., Nam,J.-G., Kang,H., Kim,Y.-J., Cheong,H.-M., Yang,J.-S., Park,S., Kim,H., Han,Y.W., Lee,H.S., Kim,A.-R., Heo,D.R., Kim,J.A., Kim,S.J., Jung,H.-D., Kim,D.-W., Yun,M.-R. | 2017-07-25T00:00:00Z | ssRNA(+) | 1353 | GenBank | S | South Korea | Homo sapiens | 22/05/2015 |
| AIZ74405.1 | Ar Gouilh,M., Thiberge,J.M., Diancourt,L., Vandenbogaert,M., Briand,D., Batejat,C., Coralie,G., Rousseau,C., Fichenick,F., van der Werf,S., Burguiere,A., Enouf,V., Manuguerra,J.C., Caro,V. | 2015-03-10T00:00:00Z | ssRNA(+) | 1353 | GenBank | S | France | Homo sapiens | 26/04/2013 |
| AIZ74417.1 | Ar Gouilh,M., Thiberge,J.M., Diancourt,L., Vandenbogaert,M., Briand,D., Batejat,C., Coralie,G., Rousseau,C., Fichenick,F., van der Werf,S., Burguiere,A., Enouf,V., Manuguerra,J.C., Caro,V. | 2015-03-10T00:00:00Z | ssRNA(+) | 1353 | GenBank | S | France | Homo sapiens | 26/04/2013 |
| AIZ74433.1 | Ar Gouilh,M., Thiberge,J.M., Diancourt,L., Vandenbogaert,M., Briand,D., Batejat,C., Coralie,G., Rousseau,C., Fichenick,F., van der Werf,S., Burguiere,A., Enouf,V., Manuguerra,J.C., Caro,V. | 2015-03-10T00:00:00Z | ssRNA(+) | 1353 | GenBank | S | France | Homo sapiens | 07/05/2013 |
| AIZ74439.1 | Ar Gouilh,M., Thiberge,J.M., Diancourt,L., Vandenbogaert,M., Briand,D., Batejat,C., Coralie,G., Rousseau,C., Fichenick,F., van der Werf,S., Burguiere,A., Enouf,V., Manuguerra,J.C., Caro,V. | 2015-03-10T00:00:00Z | ssRNA(+) | 1353 | GenBank | S | France | Homo sapiens | 07/05/2013 |
| AIZ74450.1 | Ar Gouilh,M., Thiberge,J.M., Diancourt,L., Vandenbogaert,M., Briand,D., Batejat,C., Coralie,G., Rousseau,C., Fichenick,F., van der Werf,S., Burguiere,A., Enouf,V., Manuguerra,J.C., Caro,V. | 2015-03-10T00:00:00Z | ssRNA(+) | 1353 | GenBank | S | France | Homo sapiens | 07/05/2013 |
| AHE78108.1 | Azhar,E.I., El-Kafrawy,S.A., Farraj,S.A., Hassan,A.M., Al-Saeed,M.S., Hashem,A.M., Madani,T.A., Alsaeed,M.S., Alawi,M.M. | 2014-05-01T00:00:00Z | ssRNA(+) | 1353 | GenBank | S | Saudi Arabia | Homo sapiens | 05/11/2013 |
| AHC74088.1 | Haagmans,B.L., Al Dhahiry,S.H., Reusken,C.B., Raj,V.S., Galiano,M., Myers,R., Godeke,G.J., Jonges,M., Farag,E., Diab,A., Ghobashy,H., Alhajri,F., Al-Thani,M., Al-Marri,S.A., Al Romaihi,H.E., Al Khal,A., Bermingham,A., Osterhaus,A.D., Alhajri,M.M., Koopmans,M.P., Ledesma,J., Lythgow,K., Sadler,C., Omeng-Kumi,N., Ellis,J., Al-Marri,S., Al-Romaihi,H., Al-Khal,A., Al-Dhahiry,S., Al-Hajri,M. | 2013-12-23T00:00:00Z | ssRNA(+) | 1353 | GenBank | S | Qatar | Homo sapiens | 13/10/2013 |
| AHC74098.1 | Haagmans,B.L., Al Dhahiry,S.H., Reusken,C.B., Raj,V.S., Galiano,M., Myers,R., Godeke,G.J., Jonges,M., Farag,E., Diab,A., Ghobashy,H., Alhajri,F., Al-Thani,M., Al-Marri,S.A., Al Romaihi,H.E., Al Khal,A., Bermingham,A., Osterhaus,A.D., Alhajri,M.M., Koopmans,M.P., Ledesma,J., Lythgow,K., Sadler,C., Omeng-Kumi,N., Ellis,J., Al-Marri,S., Al-Romaihi,H., Al-Khal,A., Al-Dhahiry,S., Al-Hajri,M. | 2013-12-23T00:00:00Z | ssRNA(+) | 1353 | GenBank | S | Qatar | Homo sapiens | 17/10/2013 |
| QLD97923.1 | Barry,M., Phan,M.V., Akkielah,L., Al-Majed,F., Alhetheel,A., Somily,A., Alsubaie,S.S., McNabb,S.J., Cotten,M., Zumla,A., Memish,Z.A., Phan,M.V.T. | 2020-07-12T00:00:00Z | ssRNA(+) | 1353 | GenBank | S protein | Saudi Arabia | Homo sapiens | 18/02/2015 |
| QLD97934.1 | Barry,M., Phan,M.V., Akkielah,L., Al-Majed,F., Alhetheel,A., Somily,A., Alsubaie,S.S., McNabb,S.J., Cotten,M., Zumla,A., Memish,Z.A., Phan,M.V.T. | 2020-07-12T00:00:00Z | ssRNA(+) | 1353 | GenBank | S protein | Saudi Arabia | Homo sapiens | 23/02/2015 |
| QLD97944.1 | Barry,M., Phan,M.V., Akkielah,L., Al-Majed,F., Alhetheel,A., Somily,A., Alsubaie,S.S., McNabb,S.J., Cotten,M., Zumla,A., Memish,Z.A., Phan,M.V.T. | 2020-07-12T00:00:00Z | ssRNA(+) | 1353 | GenBank | S protein | Saudi Arabia | Homo sapiens | 23/02/2015 |
| QLD97955.1 | Barry,M., Phan,M.V., Akkielah,L., Al-Majed,F., Alhetheel,A., Somily,A., Alsubaie,S.S., McNabb,S.J., Cotten,M., Zumla,A., Memish,Z.A., Phan,M.V.T. | 2020-07-12T00:00:00Z | ssRNA(+) | 1353 | GenBank | S protein | Saudi Arabia | Homo sapiens | 25/02/2015 |
| QLD97966.1 | Barry,M., Phan,M.V., Akkielah,L., Al-Majed,F., Alhetheel,A., Somily,A., Alsubaie,S.S., McNabb,S.J., Cotten,M., Zumla,A., Memish,Z.A., Phan,M.V.T. | 2020-07-12T00:00:00Z | ssRNA(+) | 1353 | GenBank | S protein | Saudi Arabia | Homo sapiens | 27/02/2015 |
| QLD97977.1 | Barry,M., Phan,M.V., Akkielah,L., Al-Majed,F., Alhetheel,A., Somily,A., Alsubaie,S.S., McNabb,S.J., Cotten,M., Zumla,A., Memish,Z.A., Phan,M.V.T. | 2020-07-12T00:00:00Z | ssRNA(+) | 1353 | GenBank | S protein | Saudi Arabia | Homo sapiens | 28/02/2015 |
| QLD97987.1 | Barry,M., Phan,M.V., Akkielah,L., Al-Majed,F., Alhetheel,A., Somily,A., Alsubaie,S.S., McNabb,S.J., Cotten,M., Zumla,A., Memish,Z.A., Phan,M.V.T. | 2020-07-12T00:00:00Z | ssRNA(+) | 1353 | GenBank | S protein | Saudi Arabia | Homo sapiens | 28/02/2015 |
| QLD97998.1 | Barry,M., Phan,M.V., Akkielah,L., Al-Majed,F., Alhetheel,A., Somily,A., Alsubaie,S.S., McNabb,S.J., Cotten,M., Zumla,A., Memish,Z.A., Phan,M.V.T. | 2020-07-12T00:00:00Z | ssRNA(+) | 1353 | GenBank | S protein | Saudi Arabia | Homo sapiens | 03/03/2015 |
| QLD98009.1 | Barry,M., Phan,M.V., Akkielah,L., Al-Majed,F., Alhetheel,A., Somily,A., Alsubaie,S.S., McNabb,S.J., Cotten,M., Zumla,A., Memish,Z.A., Phan,M.V.T. | 2020-07-12T00:00:00Z | ssRNA(+) | 1353 | GenBank | S protein | Saudi Arabia | Homo sapiens | 03/03/2015 |
| QLD98020.1 | Barry,M., Phan,M.V., Akkielah,L., Al-Majed,F., Alhetheel,A., Somily,A., Alsubaie,S.S., McNabb,S.J., Cotten,M., Zumla,A., Memish,Z.A., Phan,M.V.T. | 2020-07-12T00:00:00Z | ssRNA(+) | 1353 | GenBank | S protein | Saudi Arabia | Homo sapiens | 04/03/2015 |
| QLD98031.1 | Barry,M., Phan,M.V., Akkielah,L., Al-Majed,F., Alhetheel,A., Somily,A., Alsubaie,S.S., McNabb,S.J., Cotten,M., Zumla,A., Memish,Z.A., Phan,M.V.T. | 2020-07-12T00:00:00Z | ssRNA(+) | 1353 | GenBank | S protein | Saudi Arabia | Homo sapiens | 04/03/2015 |
| QLD98042.1 | Barry,M., Phan,M.V., Akkielah,L., Al-Majed,F., Alhetheel,A., Somily,A., Alsubaie,S.S., McNabb,S.J., Cotten,M., Zumla,A., Memish,Z.A., Phan,M.V.T. | 2020-07-12T00:00:00Z | ssRNA(+) | 1353 | GenBank | S protein | Saudi Arabia | Homo sapiens | 08/03/2015 |
| QLD98053.1 | Barry,M., Phan,M.V., Akkielah,L., Al-Majed,F., Alhetheel,A., Somily,A., Alsubaie,S.S., McNabb,S.J., Cotten,M., Zumla,A., Memish,Z.A., Phan,M.V.T. | 2020-07-12T00:00:00Z | ssRNA(+) | 1353 | GenBank | S protein | Saudi Arabia | Homo sapiens | 08/03/2015 |
| QLD98064.1 | Barry,M., Phan,M.V., Akkielah,L., Al-Majed,F., Alhetheel,A., Somily,A., Alsubaie,S.S., McNabb,S.J., Cotten,M., Zumla,A., Memish,Z.A., Phan,M.V.T. | 2020-07-12T00:00:00Z | ssRNA(+) | 1353 | GenBank | S protein | Saudi Arabia | Homo sapiens | 09/03/2015 |
| QLD98075.1 | Barry,M., Phan,M.V., Akkielah,L., Al-Majed,F., Alhetheel,A., Somily,A., Alsubaie,S.S., McNabb,S.J., Cotten,M., Zumla,A., Memish,Z.A., Phan,M.V.T. | 2020-07-12T00:00:00Z | ssRNA(+) | 1353 | GenBank | S protein | Saudi Arabia | Homo sapiens | 01/03/2015 |
| QLD98084.1 | Barry,M., Phan,M.V., Akkielah,L., Al-Majed,F., Alhetheel,A., Somily,A., Alsubaie,S.S., McNabb,S.J., Cotten,M., Zumla,A., Memish,Z.A., Phan,M.V.T. | 2020-07-12T00:00:00Z | ssRNA(+) | 1353 | GenBank | S protein | Saudi Arabia | Homo sapiens | 02/03/2015 |
| QLD98094.1 | Barry,M., Phan,M.V., Akkielah,L., Al-Majed,F., Alhetheel,A., Somily,A., Alsubaie,S.S., McNabb,S.J., Cotten,M., Zumla,A., Memish,Z.A., Phan,M.V.T. | 2020-07-12T00:00:00Z | ssRNA(+) | 1353 | GenBank | S protein | Saudi Arabia | Homo sapiens | 02/03/2015 |
| QLD98105.1 | Barry,M., Phan,M.V., Akkielah,L., Al-Majed,F., Alhetheel,A., Somily,A., Alsubaie,S.S., McNabb,S.J., Cotten,M., Zumla,A., Memish,Z.A., Phan,M.V.T. | 2020-07-12T00:00:00Z | ssRNA(+) | 1184 | GenBank | S protein | Saudi Arabia | Homo sapiens | 02/03/2015 |
| QFN25154.1 | Schroeder,S., Drosten,C., Corman,V.M., Muth,D., Muller,M.A. | 2020-05-01T00:00:00Z | ssRNA(+) | 1353 | GenBank | S protein | Saudi Arabia | Homo sapiens | 2014-04 |
| QFN25165.1 | Schroeder,S., Drosten,C., Corman,V.M., Muth,D., Muller,M.A. | 2020-05-01T00:00:00Z | ssRNA(+) | 1353 | GenBank | S protein | Saudi Arabia | Homo sapiens | 2014-04 |
| QFN25176.1 | Schroeder,S., Drosten,C., Corman,V.M., Muth,D., Muller,M.A. | 2020-05-01T00:00:00Z | ssRNA(+) | 1353 | GenBank | S protein | Saudi Arabia | Homo sapiens | 2014-04 |
| QFN25187.1 | Schroeder,S., Drosten,C., Corman,V.M., Muth,D., Muller,M.A. | 2020-05-01T00:00:00Z | ssRNA(+) | 1353 | GenBank | S protein | Saudi Arabia | Homo sapiens | 2014-05 |
| QFN25198.1 | Schroeder,S., Drosten,C., Corman,V.M., Muth,D., Muller,M.A. | 2020-05-01T00:00:00Z | ssRNA(+) | 1353 | GenBank | S protein | Saudi Arabia | Homo sapiens | 2014-05 |
| QFN25209.1 | Schroeder,S., Drosten,C., Corman,V.M., Muth,D., Muller,M.A. | 2020-05-01T00:00:00Z | ssRNA(+) | 1353 | GenBank | S protein | Saudi Arabia | Homo sapiens | 2014-06 |
| QFN25220.1 | Schroeder,S., Drosten,C., Corman,V.M., Muth,D., Muller,M.A. | 2020-05-01T00:00:00Z | ssRNA(+) | 1353 | GenBank | S protein | Saudi Arabia | Homo sapiens | 2014-04 |
| QFN25231.1 | Schroeder,S., Drosten,C., Corman,V.M., Muth,D., Muller,M.A. | 2020-05-01T00:00:00Z | ssRNA(+) | 1353 | GenBank | S protein | Saudi Arabia | Homo sapiens | 2014-04 |
| QFN25242.1 | Schroeder,S., Drosten,C., Corman,V.M., Muth,D., Muller,M.A. | 2020-05-01T00:00:00Z | ssRNA(+) | 1353 | GenBank | S protein | Saudi Arabia | Homo sapiens | 2014-04 |
| QFN25253.1 | Schroeder,S., Drosten,C., Corman,V.M., Muth,D., Muller,M.A. | 2020-05-01T00:00:00Z | ssRNA(+) | 1353 | GenBank | S protein | Saudi Arabia | Homo sapiens | 2014-04 |
| QFN25264.1 | Schroeder,S., Drosten,C., Corman,V.M., Muth,D., Muller,M.A. | 2020-05-01T00:00:00Z | ssRNA(+) | 1353 | GenBank | S protein | Saudi Arabia | Homo sapiens | 2014-04 |
| QFN25275.1 | Schroeder,S., Drosten,C., Corman,V.M., Muth,D., Muller,M.A. | 2020-05-01T00:00:00Z | ssRNA(+) | 1353 | GenBank | S protein | Saudi Arabia | Homo sapiens | 2014-04 |
| QFN25286.1 | Schroeder,S., Drosten,C., Corman,V.M., Muth,D., Muller,M.A. | 2020-05-01T00:00:00Z | ssRNA(+) | 1353 | GenBank | S protein | Saudi Arabia | Homo sapiens | 2014-04 |
| QFN25297.1 | Schroeder,S., Drosten,C., Corman,V.M., Muth,D., Muller,M.A. | 2020-05-01T00:00:00Z | ssRNA(+) | 1353 | GenBank | S protein | Saudi Arabia | Homo sapiens | 2014-04 |
| QFN25308.1 | Schroeder,S., Drosten,C., Corman,V.M., Muth,D., Muller,M.A. | 2020-05-01T00:00:00Z | ssRNA(+) | 1353 | GenBank | S protein | Saudi Arabia | Homo sapiens | 2014-04 |
| QFN25319.1 | Schroeder,S., Drosten,C., Corman,V.M., Muth,D., Muller,M.A. | 2020-05-01T00:00:00Z | ssRNA(+) | 1353 | GenBank | S protein | Saudi Arabia | Homo sapiens | 2015-09 |
| QFN25330.1 | Schroeder,S., Drosten,C., Corman,V.M., Muth,D., Muller,M.A. | 2020-05-01T00:00:00Z | ssRNA(+) | 1353 | GenBank | S protein | Saudi Arabia | Homo sapiens | 2015-09 |
| QFN25341.1 | Schroeder,S., Drosten,C., Corman,V.M., Muth,D., Muller,M.A. | 2020-05-01T00:00:00Z | ssRNA(+) | 1353 | GenBank | S protein | Saudi Arabia | Homo sapiens | 2015-09 |
| QFN25352.1 | Schroeder,S., Drosten,C., Corman,V.M., Muth,D., Muller,M.A. | 2020-05-01T00:00:00Z | ssRNA(+) | 1353 | GenBank | S protein | Saudi Arabia | Homo sapiens | 2015-11 |
| QFN25363.1 | Schroeder,S., Drosten,C., Corman,V.M., Muth,D., Muller,M.A. | 2020-05-01T00:00:00Z | ssRNA(+) | 1353 | GenBank | S protein | Saudi Arabia | Homo sapiens | 2015-11 |
| QFN25374.1 | Schroeder,S., Drosten,C., Corman,V.M., Muth,D., Muller,M.A. | 2020-05-01T00:00:00Z | ssRNA(+) | 1353 | GenBank | S protein | Saudi Arabia | Homo sapiens | 2015-11 |
| QFN25385.1 | Schroeder,S., Drosten,C., Corman,V.M., Muth,D., Muller,M.A. | 2020-05-01T00:00:00Z | ssRNA(+) | 1353 | GenBank | S protein | Saudi Arabia | Homo sapiens | 2015-11 |
| QFN25396.1 | Schroeder,S., Drosten,C., Corman,V.M., Muth,D., Muller,M.A. | 2020-05-01T00:00:00Z | ssRNA(+) | 1353 | GenBank | S protein | Saudi Arabia | Homo sapiens | 2015-11 |
| QEJ82215.1 | Lu,X., Assiri,A.M., Watson,J.T., Gerber,S.I., Lindstrom,S. | 2019-09-04T00:00:00Z | ssRNA(+) | 1353 | GenBank | S protein | Saudi Arabia | Homo sapiens | 28/01/2019 |
| QEJ82226.1 | Lu,X., Assiri,A.M., Watson,J.T., Gerber,S.I., Lindstrom,S. | 2019-09-04T00:00:00Z | ssRNA(+) | 1353 | GenBank | S protein | Saudi Arabia | Homo sapiens | 15/02/2019 |
| QBF80455.1 | Lu,X., Assiri,A.M., Watson,J.T., Gerber,S.I., Lindstrom,S. | 2019-03-02T00:00:00Z | ssRNA(+) | 1353 | GenBank | S protein | Saudi Arabia | Homo sapiens | 01/08/2017 |
| QBF80466.1 | Lu,X., Assiri,A.M., Watson,J.T., Gerber,S.I., Lindstrom,S. | 2019-03-02T00:00:00Z | ssRNA(+) | 1353 | GenBank | S protein | Saudi Arabia | Homo sapiens | 07/08/2017 |
| QBF80477.1 | Lu,X., Assiri,A.M., Watson,J.T., Gerber,S.I., Lindstrom,S. | 2019-03-02T00:00:00Z | ssRNA(+) | 1353 | GenBank | S protein | Saudi Arabia | Homo sapiens | 15/08/2017 |
| QBF80488.1 | Lu,X., Assiri,A.M., Watson,J.T., Gerber,S.I., Lindstrom,S. | 2019-03-02T00:00:00Z | ssRNA(+) | 1353 | GenBank | S protein | Saudi Arabia | Homo sapiens | 17/08/2017 |
| QBF80499.1 | Lu,X., Assiri,A.M., Watson,J.T., Gerber,S.I., Lindstrom,S. | 2019-03-02T00:00:00Z | ssRNA(+) | 1353 | GenBank | S protein | Saudi Arabia | Homo sapiens | 08/06/2018 |
| QBF80510.1 | Lu,X., Assiri,A.M., Watson,J.T., Gerber,S.I., Lindstrom,S. | 2019-03-02T00:00:00Z | ssRNA(+) | 1353 | GenBank | S protein | Saudi Arabia | Homo sapiens | 13/06/2018 |
| QBF80521.1 | Lu,X., Assiri,A.M., Watson,J.T., Gerber,S.I., Lindstrom,S. | 2019-03-02T00:00:00Z | ssRNA(+) | 1353 | GenBank | S protein | Saudi Arabia | Homo sapiens | 09/07/2018 |
| QBF80532.1 | Lu,X., Assiri,A.M., Watson,J.T., Gerber,S.I., Lindstrom,S. | 2019-03-02T00:00:00Z | ssRNA(+) | 1353 | GenBank | S protein | Saudi Arabia | Homo sapiens | 12/07/2018 |
| QBF80543.1 | Lu,X., Assiri,A.M., Watson,J.T., Gerber,S.I., Lindstrom,S. | 2019-03-02T00:00:00Z | ssRNA(+) | 1353 | GenBank | S protein | Saudi Arabia | Homo sapiens | 18/07/2018 |
| QBF80554.1 | Lu,X., Assiri,A.M., Watson,J.T., Gerber,S.I., Lindstrom,S. | 2019-03-02T00:00:00Z | ssRNA(+) | 1353 | GenBank | S protein | Saudi Arabia | Homo sapiens | 03/08/2018 |
| QBF80565.1 | Lu,X., Assiri,A.M., Watson,J.T., Gerber,S.I., Lindstrom,S. | 2019-03-02T00:00:00Z | ssRNA(+) | 1353 | GenBank | S protein | Saudi Arabia | Homo sapiens | 30/08/2018 |
| QBF80576.1 | Lu,X., Assiri,A.M., Watson,J.T., Gerber,S.I., Lindstrom,S. | 2019-03-02T00:00:00Z | ssRNA(+) | 1353 | GenBank | S protein | Saudi Arabia | Homo sapiens | 31/08/2018 |
| QBF80587.1 | Lu,X., Assiri,A.M., Watson,J.T., Gerber,S.I., Lindstrom,S. | 2019-03-02T00:00:00Z | ssRNA(+) | 1353 | GenBank | S protein | Saudi Arabia | Homo sapiens | 15/09/2018 |
| QBF80598.1 | Lu,X., Assiri,A.M., Watson,J.T., Gerber,S.I., Lindstrom,S. | 2019-03-02T00:00:00Z | ssRNA(+) | 1353 | GenBank | S protein | Saudi Arabia | Homo sapiens | 15/09/2018 |
| QBF80611.1 | Lu,X., Assiri,A.M., Watson,J.T., Gerber,S.I., Lindstrom,S. | 2019-03-02T00:00:00Z | ssRNA(+) | 1353 | GenBank | S protein | Saudi Arabia | Homo sapiens | 16/08/2018 |
| AXN73393.1 | Al-Abdely,H.M., Midgley,C.M., Alkhamis,A.M., Abedi,G.R., Lu,X., Binder,A.M., Alanazi,K., Banjar,W., Abdalla,O., Dahl,R.M., Mohammed,M., Tamin,A., Algarni,H.S., Sakthivel,S.K., Algwizani,A., Bafaqeeh,F., Alzahrani,A., Curns,A.T., Alsharef,A.A., Alhakeem,R.F., Thornburg,N.J., Jokhdar,H., Ghazal,S.S., Erdman,D.D., Assiri,A.M., Watson,J.T., Gerber,S.I. | 2019-01-31T00:00:00Z | ssRNA(+) | 1353 | GenBank | S protein | Saudi Arabia | Homo sapiens | 22/08/2015 |
| AXN73404.1 | Al-Abdely,H.M., Midgley,C.M., Alkhamis,A.M., Abedi,G.R., Lu,X., Binder,A.M., Alanazi,K., Banjar,W., Abdalla,O., Dahl,R.M., Mohammed,M., Tamin,A., Algarni,H.S., Sakthivel,S.K., Algwizani,A., Bafaqeeh,F., Alzahrani,A., Curns,A.T., Alsharef,A.A., Alhakeem,R.F., Thornburg,N.J., Jokhdar,H., Ghazal,S.S., Erdman,D.D., Assiri,A.M., Watson,J.T., Gerber,S.I. | 2019-01-31T00:00:00Z | ssRNA(+) | 1353 | GenBank | S protein | Saudi Arabia | Homo sapiens | 24/08/2015 |
| AXN73415.1 | Al-Abdely,H.M., Midgley,C.M., Alkhamis,A.M., Abedi,G.R., Lu,X., Binder,A.M., Alanazi,K., Banjar,W., Abdalla,O., Dahl,R.M., Mohammed,M., Tamin,A., Algarni,H.S., Sakthivel,S.K., Algwizani,A., Bafaqeeh,F., Alzahrani,A., Curns,A.T., Alsharef,A.A., Alhakeem,R.F., Thornburg,N.J., Jokhdar,H., Ghazal,S.S., Erdman,D.D., Assiri,A.M., Watson,J.T., Gerber,S.I. | 2019-01-31T00:00:00Z | ssRNA(+) | 1353 | GenBank | S protein | Saudi Arabia | Homo sapiens | 24/08/2015 |
| AXN73426.1 | Al-Abdely,H.M., Midgley,C.M., Alkhamis,A.M., Abedi,G.R., Lu,X., Binder,A.M., Alanazi,K., Banjar,W., Abdalla,O., Dahl,R.M., Mohammed,M., Tamin,A., Algarni,H.S., Sakthivel,S.K., Algwizani,A., Bafaqeeh,F., Alzahrani,A., Curns,A.T., Alsharef,A.A., Alhakeem,R.F., Thornburg,N.J., Jokhdar,H., Ghazal,S.S., Erdman,D.D., Assiri,A.M., Watson,J.T., Gerber,S.I. | 2019-01-31T00:00:00Z | ssRNA(+) | 1353 | GenBank | S protein | Saudi Arabia | Homo sapiens | 24/08/2015 |
| AXN73437.1 | Al-Abdely,H.M., Midgley,C.M., Alkhamis,A.M., Abedi,G.R., Lu,X., Binder,A.M., Alanazi,K., Banjar,W., Abdalla,O., Dahl,R.M., Mohammed,M., Tamin,A., Algarni,H.S., Sakthivel,S.K., Algwizani,A., Bafaqeeh,F., Alzahrani,A., Curns,A.T., Alsharef,A.A., Alhakeem,R.F., Thornburg,N.J., Jokhdar,H., Ghazal,S.S., Erdman,D.D., Assiri,A.M., Watson,J.T., Gerber,S.I. | 2019-01-31T00:00:00Z | ssRNA(+) | 1353 | GenBank | S protein | Saudi Arabia | Homo sapiens | 24/08/2015 |
| AXN73448.1 | Al-Abdely,H.M., Midgley,C.M., Alkhamis,A.M., Abedi,G.R., Lu,X., Binder,A.M., Alanazi,K., Banjar,W., Abdalla,O., Dahl,R.M., Mohammed,M., Tamin,A., Algarni,H.S., Sakthivel,S.K., Algwizani,A., Bafaqeeh,F., Alzahrani,A., Curns,A.T., Alsharef,A.A., Alhakeem,R.F., Thornburg,N.J., Jokhdar,H., Ghazal,S.S., Erdman,D.D., Assiri,A.M., Watson,J.T., Gerber,S.I. | 2019-01-31T00:00:00Z | ssRNA(+) | 1353 | GenBank | S protein | Saudi Arabia | Homo sapiens | 26/08/2015 |
| AXN73459.1 | Al-Abdely,H.M., Midgley,C.M., Alkhamis,A.M., Abedi,G.R., Lu,X., Binder,A.M., Alanazi,K., Banjar,W., Abdalla,O., Dahl,R.M., Mohammed,M., Tamin,A., Algarni,H.S., Sakthivel,S.K., Algwizani,A., Bafaqeeh,F., Alzahrani,A., Curns,A.T., Alsharef,A.A., Alhakeem,R.F., Thornburg,N.J., Jokhdar,H., Ghazal,S.S., Erdman,D.D., Assiri,A.M., Watson,J.T., Gerber,S.I. | 2019-01-31T00:00:00Z | ssRNA(+) | 1353 | GenBank | S protein | Saudi Arabia | Homo sapiens | 26/08/2015 |
| AXN73470.1 | Al-Abdely,H.M., Midgley,C.M., Alkhamis,A.M., Abedi,G.R., Lu,X., Binder,A.M., Alanazi,K., Banjar,W., Abdalla,O., Dahl,R.M., Mohammed,M., Tamin,A., Algarni,H.S., Sakthivel,S.K., Algwizani,A., Bafaqeeh,F., Alzahrani,A., Curns,A.T., Alsharef,A.A., Alhakeem,R.F., Thornburg,N.J., Jokhdar,H., Ghazal,S.S., Erdman,D.D., Assiri,A.M., Watson,J.T., Gerber,S.I. | 2019-01-31T00:00:00Z | ssRNA(+) | 1353 | GenBank | S protein | Saudi Arabia | Homo sapiens | 01/09/2015 |
| AXN73481.1 | Al-Abdely,H.M., Midgley,C.M., Alkhamis,A.M., Abedi,G.R., Lu,X., Binder,A.M., Alanazi,K., Banjar,W., Abdalla,O., Dahl,R.M., Mohammed,M., Tamin,A., Algarni,H.S., Sakthivel,S.K., Algwizani,A., Bafaqeeh,F., Alzahrani,A., Curns,A.T., Alsharef,A.A., Alhakeem,R.F., Thornburg,N.J., Jokhdar,H., Ghazal,S.S., Erdman,D.D., Assiri,A.M., Watson,J.T., Gerber,S.I. | 2019-01-31T00:00:00Z | ssRNA(+) | 1353 | GenBank | S protein | Saudi Arabia | Homo sapiens | 01/09/2015 |
| AXN73492.1 | Al-Abdely,H.M., Midgley,C.M., Alkhamis,A.M., Abedi,G.R., Lu,X., Binder,A.M., Alanazi,K., Banjar,W., Abdalla,O., Dahl,R.M., Mohammed,M., Tamin,A., Algarni,H.S., Sakthivel,S.K., Algwizani,A., Bafaqeeh,F., Alzahrani,A., Curns,A.T., Alsharef,A.A., Alhakeem,R.F., Thornburg,N.J., Jokhdar,H., Ghazal,S.S., Erdman,D.D., Assiri,A.M., Watson,J.T., Gerber,S.I. | 2019-01-31T00:00:00Z | ssRNA(+) | 1353 | GenBank | S protein | Saudi Arabia | Homo sapiens | 01/09/2015 |
| AXN73503.1 | Al-Abdely,H.M., Midgley,C.M., Alkhamis,A.M., Abedi,G.R., Lu,X., Binder,A.M., Alanazi,K., Banjar,W., Abdalla,O., Dahl,R.M., Mohammed,M., Tamin,A., Algarni,H.S., Sakthivel,S.K., Algwizani,A., Bafaqeeh,F., Alzahrani,A., Curns,A.T., Alsharef,A.A., Alhakeem,R.F., Thornburg,N.J., Jokhdar,H., Ghazal,S.S., Erdman,D.D., Assiri,A.M., Watson,J.T., Gerber,S.I. | 2019-01-31T00:00:00Z | ssRNA(+) | 1353 | GenBank | S protein | Saudi Arabia | Homo sapiens | 18/09/2015 |
| AXN73514.1 | Al-Abdely,H.M., Midgley,C.M., Alkhamis,A.M., Abedi,G.R., Lu,X., Binder,A.M., Alanazi,K., Banjar,W., Abdalla,O., Dahl,R.M., Mohammed,M., Tamin,A., Algarni,H.S., Sakthivel,S.K., Algwizani,A., Bafaqeeh,F., Alzahrani,A., Curns,A.T., Alsharef,A.A., Alhakeem,R.F., Thornburg,N.J., Jokhdar,H., Ghazal,S.S., Erdman,D.D., Assiri,A.M., Watson,J.T., Gerber,S.I. | 2019-01-31T00:00:00Z | ssRNA(+) | 1353 | GenBank | S protein | Saudi Arabia | Homo sapiens | 31/01/2016 |
| AXN73525.1 | Al-Abdely,H.M., Midgley,C.M., Alkhamis,A.M., Abedi,G.R., Lu,X., Binder,A.M., Alanazi,K., Banjar,W., Abdalla,O., Dahl,R.M., Mohammed,M., Tamin,A., Algarni,H.S., Sakthivel,S.K., Algwizani,A., Bafaqeeh,F., Alzahrani,A., Curns,A.T., Alsharef,A.A., Alhakeem,R.F., Thornburg,N.J., Jokhdar,H., Ghazal,S.S., Erdman,D.D., Assiri,A.M., Watson,J.T., Gerber,S.I. | 2019-01-31T00:00:00Z | ssRNA(+) | 1353 | GenBank | S protein | Saudi Arabia | Homo sapiens | 14/04/2016 |
| AZU90731.1 | Widagdo,W., Okba,N.M.A., Richard,M., de Meulder,D., Bestebroer,T.M., Lexmond,P., Farag,E.A.B.A., Al-Hajri,M., Stittelaar,K.J., de Waal,L., van Amerongen,G., van den Brand,J.M.A., Haagmans,B.L., Herfst,S., Farag,A.A.B.A. | 2019-01-13T00:00:00Z | ssRNA(+) | 1353 | GenBank | S protein | Qatar | Homo sapiens | 21/05/2015 |
| AXN73370.1 | Al-Abdely,H.M., Midgley,C.M., Alkhamis,A.M., Abedi,G.R., Tamin,A., Binder,A.M., Alanazi,K., Lu,X., Abdalla,O., Sakthivel,S.K., Mohammed,M., Queen,K., Algarni,H.S., Li,Y., Trivedi,S., Algwizani,A., Alhakeem,R.F., Thornburg,N.J., Tong,S., Ghazal,S.S., Erdman,D.D., Assiri,A.M., Gerber,S.I., Watson,J.T. | 2018-10-22T00:00:00Z | ssRNA(+) | 1353 | GenBank | S protein | Saudi Arabia | Homo sapiens | 13/10/2015 |
| AXN73381.1 | Al-Abdely,H.M., Midgley,C.M., Alkhamis,A.M., Abedi,G.R., Tamin,A., Binder,A.M., Alanazi,K., Lu,X., Abdalla,O., Sakthivel,S.K., Mohammed,M., Queen,K., Algarni,H.S., Li,Y., Trivedi,S., Algwizani,A., Alhakeem,R.F., Thornburg,N.J., Tong,S., Ghazal,S.S., Erdman,D.D., Assiri,A.M., Gerber,S.I., Watson,J.T. | 2018-10-22T00:00:00Z | ssRNA(+) | 1353 | GenBank | S protein | Saudi Arabia | Homo sapiens | 15/10/2015 |
| ASY99778.1 | Payne,D.C., Biggs,H.M., Al-Abdallat,M.M., Alqasrawi,S., Lu,X., Abedi,G.R., Haddadin,A., Iblan,I., Al-Sanouri,T., Al Nsour,M., Ali,S.S., Rha,B., Trivedi,S.U., Rasheed,M., Tamin,A., Lamers,M.M., Haagmans,B.L., Erdman,D.D., Thornburg,N., Gerber,S.I., Alsanouri,T. | 2018-08-31T00:00:00Z | ssRNA(+) | 1353 | GenBank | S protein | Jordan | Homo sapiens | 07/09/2015 |
| ASY99789.1 | Payne,D.C., Biggs,H.M., Al-Abdallat,M.M., Alqasrawi,S., Lu,X., Abedi,G.R., Haddadin,A., Iblan,I., Al-Sanouri,T., Al Nsour,M., Ali,S.S., Rha,B., Trivedi,S.U., Rasheed,M., Tamin,A., Lamers,M.M., Haagmans,B.L., Erdman,D.D., Thornburg,N., Gerber,S.I., Alsanouri,T. | 2018-08-31T00:00:00Z | ssRNA(+) | 1353 | GenBank | S protein | Jordan | Homo sapiens | 25/08/2015 |
| ASY99800.1 | Payne,D.C., Biggs,H.M., Al-Abdallat,M.M., Alqasrawi,S., Lu,X., Abedi,G.R., Haddadin,A., Iblan,I., Al-Sanouri,T., Al Nsour,M., Ali,S.S., Rha,B., Trivedi,S.U., Rasheed,M., Tamin,A., Lamers,M.M., Haagmans,B.L., Erdman,D.D., Thornburg,N., Gerber,S.I., Alsanouri,T. | 2018-08-31T00:00:00Z | ssRNA(+) | 1353 | GenBank | S protein | Jordan | Homo sapiens | 25/08/2015 |
| ASY99811.1 | Payne,D.C., Biggs,H.M., Al-Abdallat,M.M., Alqasrawi,S., Lu,X., Abedi,G.R., Haddadin,A., Iblan,I., Al-Sanouri,T., Al Nsour,M., Ali,S.S., Rha,B., Trivedi,S.U., Rasheed,M., Tamin,A., Lamers,M.M., Haagmans,B.L., Erdman,D.D., Thornburg,N., Gerber,S.I., Alsanouri,T. | 2018-08-31T00:00:00Z | ssRNA(+) | 1353 | GenBank | S protein | Jordan | Homo sapiens | 26/08/2015 |
| ASY99820.1 | Payne,D.C., Biggs,H.M., Al-Abdallat,M.M., Alqasrawi,S., Lu,X., Abedi,G.R., Haddadin,A., Iblan,I., Al-Sanouri,T., Al Nsour,M., Ali,S.S., Rha,B., Trivedi,S.U., Rasheed,M., Tamin,A., Lamers,M.M., Haagmans,B.L., Erdman,D.D., Thornburg,N., Gerber,S.I., Alsanouri,T. | 2018-08-31T00:00:00Z | ssRNA(+) | 1353 | GenBank | S protein | Jordan | Homo sapiens | 27/08/2015 |
| ASY99831.1 | Payne,D.C., Biggs,H.M., Al-Abdallat,M.M., Alqasrawi,S., Lu,X., Abedi,G.R., Haddadin,A., Iblan,I., Al-Sanouri,T., Al Nsour,M., Ali,S.S., Rha,B., Trivedi,S.U., Rasheed,M., Tamin,A., Lamers,M.M., Haagmans,B.L., Erdman,D.D., Thornburg,N., Gerber,S.I., Alsanouri,T. | 2018-08-31T00:00:00Z | ssRNA(+) | 1353 | GenBank | S protein | Jordan | Homo sapiens | 30/08/2015 |
| ASY99842.1 | Payne,D.C., Biggs,H.M., Al-Abdallat,M.M., Alqasrawi,S., Lu,X., Abedi,G.R., Haddadin,A., Iblan,I., Al-Sanouri,T., Al Nsour,M., Ali,S.S., Rha,B., Trivedi,S.U., Rasheed,M., Tamin,A., Lamers,M.M., Haagmans,B.L., Erdman,D.D., Thornburg,N., Gerber,S.I., Alsanouri,T. | 2018-08-31T00:00:00Z | ssRNA(+) | 1353 | GenBank | S protein | Jordan | Homo sapiens | 31/08/2015 |
| ASY99853.1 | Payne,D.C., Biggs,H.M., Al-Abdallat,M.M., Alqasrawi,S., Lu,X., Abedi,G.R., Haddadin,A., Iblan,I., Al-Sanouri,T., Al Nsour,M., Ali,S.S., Rha,B., Trivedi,S.U., Rasheed,M., Tamin,A., Lamers,M.M., Haagmans,B.L., Erdman,D.D., Thornburg,N., Gerber,S.I., Alsanouri,T. | 2018-08-31T00:00:00Z | ssRNA(+) | 1353 | GenBank | S protein | Jordan | Homo sapiens | 03/09/2015 |
| ASY99864.1 | Payne,D.C., Biggs,H.M., Al-Abdallat,M.M., Alqasrawi,S., Lu,X., Abedi,G.R., Haddadin,A., Iblan,I., Al-Sanouri,T., Al Nsour,M., Ali,S.S., Rha,B., Trivedi,S.U., Rasheed,M., Tamin,A., Lamers,M.M., Haagmans,B.L., Erdman,D.D., Thornburg,N., Gerber,S.I., Alsanouri,T. | 2018-08-31T00:00:00Z | ssRNA(+) | 1353 | GenBank | S protein | Jordan | Homo sapiens | 09/09/2015 |
| ASY99873.1 | Payne,D.C., Biggs,H.M., Al-Abdallat,M.M., Alqasrawi,S., Lu,X., Abedi,G.R., Haddadin,A., Iblan,I., Al-Sanouri,T., Al Nsour,M., Ali,S.S., Rha,B., Trivedi,S.U., Rasheed,M., Tamin,A., Lamers,M.M., Haagmans,B.L., Erdman,D.D., Thornburg,N., Gerber,S.I., Alsanouri,T. | 2018-08-31T00:00:00Z | ssRNA(+) | 1353 | GenBank | S protein | Jordan | Homo sapiens | 17/09/2015 |
| ASY99884.1 | Payne,D.C., Biggs,H.M., Al-Abdallat,M.M., Alqasrawi,S., Lu,X., Abedi,G.R., Haddadin,A., Iblan,I., Al-Sanouri,T., Al Nsour,M., Ali,S.S., Rha,B., Trivedi,S.U., Rasheed,M., Tamin,A., Lamers,M.M., Haagmans,B.L., Erdman,D.D., Thornburg,N., Gerber,S.I., Alsanouri,T. | 2018-08-31T00:00:00Z | ssRNA(+) | 1353 | GenBank | S protein | Jordan | Homo sapiens | 19/09/2015 |
| ASY99895.1 | Payne,D.C., Biggs,H.M., Al-Abdallat,M.M., Alqasrawi,S., Lu,X., Abedi,G.R., Haddadin,A., Iblan,I., Al-Sanouri,T., Al Nsour,M., Ali,S.S., Rha,B., Trivedi,S.U., Rasheed,M., Tamin,A., Lamers,M.M., Haagmans,B.L., Erdman,D.D., Thornburg,N., Gerber,S.I., Alsanouri,T. | 2018-08-31T00:00:00Z | ssRNA(+) | 1353 | GenBank | S protein | Jordan | Homo sapiens | 17/09/2015 |
| ASY99906.1 | Payne,D.C., Biggs,H.M., Al-Abdallat,M.M., Alqasrawi,S., Lu,X., Abedi,G.R., Haddadin,A., Iblan,I., Al-Sanouri,T., Al Nsour,M., Ali,S.S., Rha,B., Trivedi,S.U., Rasheed,M., Tamin,A., Lamers,M.M., Haagmans,B.L., Erdman,D.D., Thornburg,N., Gerber,S.I., Alsanouri,T. | 2018-08-31T00:00:00Z | ssRNA(+) | 1353 | GenBank | S protein | Jordan | Homo sapiens | 22/09/2015 |
| AXN92228.1 | Lu,X. | 2018-08-31T00:00:00Z | ssRNA(+) | 1353 | GenBank | S protein | Saudi Arabia | Homo sapiens | 05/05/2017 |
| AXN92238.1 | Lu,X. | 2018-08-31T00:00:00Z | ssRNA(+) | 1353 | GenBank | S protein | Saudi Arabia | Homo sapiens | 03/06/2017 |
| AXN92249.1 | Lu,X. | 2018-08-31T00:00:00Z | ssRNA(+) | 1353 | GenBank | S protein | Saudi Arabia | Homo sapiens | 06/06/2017 |
| AXN92260.1 | Lu,X. | 2018-08-31T00:00:00Z | ssRNA(+) | 1353 | GenBank | S protein | Saudi Arabia | Homo sapiens | 15/05/2017 |
| AXN73359.1 | Lu,X., Assiri,A.M., Sakthivel,S.K., Biggs,H., Watson,J.T., Gerber,S.I. | 2018-08-21T00:00:00Z | ssRNA(+) | 1353 | GenBank | S protein | Saudi Arabia | Homo sapiens | 02/06/2017 |
| AQZ41282.1 | Paden,C.R., Yusof,M.F.B.M., Al Hammadi,Z.M., Queen,K., Tao,Y., Eltahir,Y.M., Elsayed,E.A., Marzoug,B.A., Bensalah,O.K.A., Khalafalla,A.I., Al Mulla,M., Elkheir,K.A., Issa,Z.B., Pradeep,K., Elsaleh,F.N., Imambaccus,H., Sasse,J., Weber,S., Shi,M., Zhang,J., Li,Y., Pham,H., Kim,L., Hall,A.J., Gerber,S.I., Al Hosani,F.I., Tong,S., Al Muhairi,S.S.M., Al Muhairi,S.S., Al Hammadi,Z., Abou Elkheir,K., Al Bandar,Z., El Saleh,F., Hall,A., Gerber,S. | 2017-12-20T00:00:00Z | ssRNA(+) | 1353 | GenBank | S protein | United Arab Emirates | Homo sapiens | 12/07/2013 |
| AQZ41283.1 | Paden,C.R., Yusof,M.F.B.M., Al Hammadi,Z.M., Queen,K., Tao,Y., Eltahir,Y.M., Elsayed,E.A., Marzoug,B.A., Bensalah,O.K.A., Khalafalla,A.I., Al Mulla,M., Elkheir,K.A., Issa,Z.B., Pradeep,K., Elsaleh,F.N., Imambaccus,H., Sasse,J., Weber,S., Shi,M., Zhang,J., Li,Y., Pham,H., Kim,L., Hall,A.J., Gerber,S.I., Al Hosani,F.I., Tong,S., Al Muhairi,S.S.M., Al Muhairi,S.S., Al Hammadi,Z., Abou Elkheir,K., Al Bandar,Z., El Saleh,F., Hall,A., Gerber,S. | 2017-12-20T00:00:00Z | ssRNA(+) | 1353 | GenBank | S protein | United Arab Emirates | Homo sapiens | 25/04/2014 |
| ATG84679.1 | Lu,X., Assiri,A.M., Erdman,D.D., Midgley,C., Watson,J.T., Gerber,S.I. | 2017-10-03T00:00:00Z | ssRNA(+) | 1353 | GenBank | S protein | Saudi Arabia | Homo sapiens | 05/03/2016 |
| ATG84690.1 | Lu,X., Assiri,A.M., Erdman,D.D., Midgley,C., Watson,J.T., Gerber,S.I. | 2017-10-03T00:00:00Z | ssRNA(+) | 1353 | GenBank | S protein | Saudi Arabia | Homo sapiens | 06/03/2016 |
| ATG84701.1 | Lu,X., Assiri,A.M., Erdman,D.D., Midgley,C., Watson,J.T., Gerber,S.I. | 2017-10-03T00:00:00Z | ssRNA(+) | 1353 | GenBank | S protein | Saudi Arabia | Homo sapiens | 07/03/2016 |
| ATG84712.1 | Lu,X., Assiri,A.M., Erdman,D.D., Midgley,C., Watson,J.T., Gerber,S.I. | 2017-10-03T00:00:00Z | ssRNA(+) | 1353 | GenBank | S protein | Saudi Arabia | Homo sapiens | 12/03/2016 |
| ATG84723.1 | Lu,X., Assiri,A.M., Erdman,D.D., Midgley,C., Watson,J.T., Gerber,S.I. | 2017-10-03T00:00:00Z | ssRNA(+) | 1353 | GenBank | S protein | Saudi Arabia | Homo sapiens | 13/03/2016 |
| ATG84734.1 | Lu,X., Assiri,A.M., Erdman,D.D., Midgley,C., Watson,J.T., Gerber,S.I. | 2017-10-03T00:00:00Z | ssRNA(+) | 1353 | GenBank | S protein | Saudi Arabia | Homo sapiens | 18/03/2016 |
| ATG84745.1 | Lu,X., Assiri,A.M., Erdman,D.D., Midgley,C., Watson,J.T., Gerber,S.I. | 2017-10-03T00:00:00Z | ssRNA(+) | 1353 | GenBank | S protein | Saudi Arabia | Homo sapiens | 14/03/2016 |
| ATG84756.1 | Lu,X., Assiri,A.M., Erdman,D.D., Midgley,C., Watson,J.T., Gerber,S.I. | 2017-10-03T00:00:00Z | ssRNA(+) | 1353 | GenBank | S protein | Saudi Arabia | Homo sapiens | 13/03/2016 |
| ATG84767.1 | Lu,X., Assiri,A.M., Erdman,D.D., Midgley,C., Watson,J.T., Gerber,S.I. | 2017-10-03T00:00:00Z | ssRNA(+) | 1353 | GenBank | S protein | Saudi Arabia | Homo sapiens | 18/04/2016 |
| ATG84778.1 | Lu,X., Assiri,A.M., Erdman,D.D., Midgley,C., Watson,J.T., Gerber,S.I. | 2017-10-03T00:00:00Z | ssRNA(+) | 1353 | GenBank | S protein | Saudi Arabia | Homo sapiens | 13/04/2016 |
| ATG84789.1 | Lu,X., Assiri,A.M., Erdman,D.D., Midgley,C., Watson,J.T., Gerber,S.I. | 2017-10-03T00:00:00Z | ssRNA(+) | 1353 | GenBank | S protein | Saudi Arabia | Homo sapiens | 04/04/2016 |
| ATG84800.1 | Lu,X., Assiri,A.M., Erdman,D.D., Midgley,C., Watson,J.T., Gerber,S.I. | 2017-10-03T00:00:00Z | ssRNA(+) | 1353 | GenBank | S protein | Saudi Arabia | Homo sapiens | 10/04/2016 |
| ATG84811.1 | Lu,X., Assiri,A.M., Erdman,D.D., Midgley,C., Watson,J.T., Gerber,S.I. | 2017-10-03T00:00:00Z | ssRNA(+) | 1353 | GenBank | S protein | Saudi Arabia | Homo sapiens | 20/07/2016 |
| ATG84822.1 | Lu,X., Assiri,A.M., Erdman,D.D., Midgley,C., Watson,J.T., Gerber,S.I. | 2017-10-03T00:00:00Z | ssRNA(+) | 1353 | GenBank | S protein | Saudi Arabia | Homo sapiens | 01/06/2016 |
| ATG84833.1 | Lu,X., Assiri,A.M., Erdman,D.D., Midgley,C., Watson,J.T., Gerber,S.I. | 2017-10-03T00:00:00Z | ssRNA(+) | 1353 | GenBank | S protein | Saudi Arabia | Homo sapiens | 16/06/2016 |
| ATG84844.1 | Lu,X., Assiri,A.M., Erdman,D.D., Midgley,C., Watson,J.T., Gerber,S.I. | 2017-10-03T00:00:00Z | ssRNA(+) | 1353 | GenBank | S protein | Saudi Arabia | Homo sapiens | 01/07/2016 |
| ATG84855.1 | Lu,X., Assiri,A.M., Erdman,D.D., Midgley,C., Watson,J.T., Gerber,S.I. | 2017-10-03T00:00:00Z | ssRNA(+) | 1353 | GenBank | S protein | Saudi Arabia | Homo sapiens | 12/07/2016 |
| ATG84866.1 | Lu,X., Assiri,A.M., Erdman,D.D., Midgley,C., Watson,J.T., Gerber,S.I. | 2017-10-03T00:00:00Z | ssRNA(+) | 1353 | GenBank | S protein | Saudi Arabia | Homo sapiens | 21/09/2016 |
| ATG84877.1 | Lu,X., Assiri,A.M., Erdman,D.D., Midgley,C., Watson,J.T., Gerber,S.I. | 2017-10-03T00:00:00Z | ssRNA(+) | 1353 | GenBank | S protein | Saudi Arabia | Homo sapiens | 21/09/2016 |
| ATG84888.1 | Lu,X., Assiri,A.M., Erdman,D.D., Midgley,C., Watson,J.T., Gerber,S.I. | 2017-10-03T00:00:00Z | ssRNA(+) | 1353 | GenBank | S protein | Saudi Arabia | Homo sapiens | 29/03/2016 |
| ATG84899.1 | Lu,X., Assiri,A.M., Erdman,D.D., Midgley,C., Watson,J.T., Gerber,S.I. | 2017-10-03T00:00:00Z | ssRNA(+) | 1353 | GenBank | S protein | Saudi Arabia | Homo sapiens | 23/01/2017 |
| ATG84910.1 | Lu,X., Assiri,A.M., Erdman,D.D., Midgley,C., Watson,J.T., Gerber,S.I. | 2017-10-03T00:00:00Z | ssRNA(+) | 1353 | GenBank | S protein | Saudi Arabia | Homo sapiens | 10/01/2017 |
| ATG84921.1 | Lu,X., Assiri,A.M., Erdman,D.D., Midgley,C., Watson,J.T., Gerber,S.I. | 2017-10-03T00:00:00Z | ssRNA(+) | 1353 | GenBank | S protein | Saudi Arabia | Homo sapiens | 25/12/2016 |
| ANF29162.1 | Assiri,A.M., Midgley,C.M., Alessa,M., Hawaj,H.A.L., Saeed,A.B., Almasri,M.M., Lu,X., Abedi,G.R., Abdalla,O., Mohammed,M., Algarni,H.S., Alhakeem,R.F., Al-Abdely,H.M., Alomar,I.A., Alsharef,A.A., Nooh,R., Erdman,D.D., Watson,J.T., Gerber,S.I. | 2017-04-30T00:00:00Z | ssRNA(+) | 1353 | GenBank | S protein | Saudi Arabia | Homo sapiens | 2016-02 |
| ANF29173.1 | Assiri,A.M., Midgley,C.M., Alessa,M., Hawaj,H.A.L., Saeed,A.B., Almasri,M.M., Lu,X., Abedi,G.R., Abdalla,O., Mohammed,M., Algarni,H.S., Alhakeem,R.F., Al-Abdely,H.M., Alomar,I.A., Alsharef,A.A., Nooh,R., Erdman,D.D., Watson,J.T., Gerber,S.I. | 2017-04-30T00:00:00Z | ssRNA(+) | 1353 | GenBank | S protein | Saudi Arabia | Homo sapiens | 2016-02 |
| ANF29184.1 | Assiri,A.M., Midgley,C.M., Alessa,M., Hawaj,H.A.L., Saeed,A.B., Almasri,M.M., Lu,X., Abedi,G.R., Abdalla,O., Mohammed,M., Algarni,H.S., Alhakeem,R.F., Al-Abdely,H.M., Alomar,I.A., Alsharef,A.A., Nooh,R., Erdman,D.D., Watson,J.T., Gerber,S.I. | 2017-04-30T00:00:00Z | ssRNA(+) | 1353 | GenBank | S protein | Saudi Arabia | Homo sapiens | 2016-02 |
| ANF29195.1 | Assiri,A.M., Midgley,C.M., Alessa,M., Hawaj,H.A.L., Saeed,A.B., Almasri,M.M., Lu,X., Abedi,G.R., Abdalla,O., Mohammed,M., Algarni,H.S., Alhakeem,R.F., Al-Abdely,H.M., Alomar,I.A., Alsharef,A.A., Nooh,R., Erdman,D.D., Watson,J.T., Gerber,S.I. | 2017-04-30T00:00:00Z | ssRNA(+) | 1353 | GenBank | S protein | Saudi Arabia | Homo sapiens | 2016-02 |
| ANF29206.1 | Assiri,A.M., Midgley,C.M., Alessa,M., Hawaj,H.A.L., Saeed,A.B., Almasri,M.M., Lu,X., Abedi,G.R., Abdalla,O., Mohammed,M., Algarni,H.S., Alhakeem,R.F., Al-Abdely,H.M., Alomar,I.A., Alsharef,A.A., Nooh,R., Erdman,D.D., Watson,J.T., Gerber,S.I. | 2017-04-30T00:00:00Z | ssRNA(+) | 1353 | GenBank | S protein | Saudi Arabia | Homo sapiens | 2016-02 |
| ANF29217.1 | Assiri,A.M., Midgley,C.M., Alessa,M., Hawaj,H.A.L., Saeed,A.B., Almasri,M.M., Lu,X., Abedi,G.R., Abdalla,O., Mohammed,M., Algarni,H.S., Alhakeem,R.F., Al-Abdely,H.M., Alomar,I.A., Alsharef,A.A., Nooh,R., Erdman,D.D., Watson,J.T., Gerber,S.I. | 2017-04-30T00:00:00Z | ssRNA(+) | 1353 | GenBank | S protein | Saudi Arabia | Homo sapiens | 2016-01 |
| ANF29228.1 | Assiri,A.M., Midgley,C.M., Alessa,M., Hawaj,H.A.L., Saeed,A.B., Almasri,M.M., Lu,X., Abedi,G.R., Abdalla,O., Mohammed,M., Algarni,H.S., Alhakeem,R.F., Al-Abdely,H.M., Alomar,I.A., Alsharef,A.A., Nooh,R., Erdman,D.D., Watson,J.T., Gerber,S.I. | 2017-04-30T00:00:00Z | ssRNA(+) | 1353 | GenBank | S protein | Saudi Arabia | Homo sapiens | 2016-01 |
| ANF29239.1 | Assiri,A.M., Midgley,C.M., Alessa,M., Hawaj,H.A.L., Saeed,A.B., Almasri,M.M., Lu,X., Abedi,G.R., Abdalla,O., Mohammed,M., Algarni,H.S., Alhakeem,R.F., Al-Abdely,H.M., Alomar,I.A., Alsharef,A.A., Nooh,R., Erdman,D.D., Watson,J.T., Gerber,S.I. | 2017-04-30T00:00:00Z | ssRNA(+) | 1353 | GenBank | S protein | Saudi Arabia | Homo sapiens | 25/02/2016 |
| ANF29250.1 | Assiri,A.M., Midgley,C.M., Alessa,M., Hawaj,H.A.L., Saeed,A.B., Almasri,M.M., Lu,X., Abedi,G.R., Abdalla,O., Mohammed,M., Algarni,H.S., Alhakeem,R.F., Al-Abdely,H.M., Alomar,I.A., Alsharef,A.A., Nooh,R., Erdman,D.D., Watson,J.T., Gerber,S.I. | 2017-04-30T00:00:00Z | ssRNA(+) | 1353 | GenBank | S protein | Saudi Arabia | Homo sapiens | 27/02/2016 |
| ANF29261.1 | Assiri,A.M., Midgley,C.M., Alessa,M., Hawaj,H.A.L., Saeed,A.B., Almasri,M.M., Lu,X., Abedi,G.R., Abdalla,O., Mohammed,M., Algarni,H.S., Alhakeem,R.F., Al-Abdely,H.M., Alomar,I.A., Alsharef,A.A., Nooh,R., Erdman,D.D., Watson,J.T., Gerber,S.I. | 2017-04-30T00:00:00Z | ssRNA(+) | 1353 | GenBank | S protein | Saudi Arabia | Homo sapiens | 29/02/2016 |
| ANF29272.1 | Assiri,A.M., Midgley,C.M., Alessa,M., Hawaj,H.A.L., Saeed,A.B., Almasri,M.M., Lu,X., Abedi,G.R., Abdalla,O., Mohammed,M., Algarni,H.S., Alhakeem,R.F., Al-Abdely,H.M., Alomar,I.A., Alsharef,A.A., Nooh,R., Erdman,D.D., Watson,J.T., Gerber,S.I. | 2017-04-30T00:00:00Z | ssRNA(+) | 1353 | GenBank | S protein | Saudi Arabia | Homo sapiens | 29/02/2016 |
| ALX27232.1 | Lu,X., Al-Abdallat,M.M., Haddadin,A., Al-Sanouri,T., Erdman,D.D. | 2016-10-02T00:00:00Z | ssRNA(+) | 1353 | GenBank | S protein | Jordan | Homo sapiens | 22/01/2014 |
| ALX27243.1 | Lu,X., Al-Abdallat,M.M., Haddadin,A., Al-Sanouri,T., Erdman,D.D. | 2016-10-02T00:00:00Z | ssRNA(+) | 1353 | GenBank | S protein | Jordan | Homo sapiens | 21/04/2014 |
| AMQ48993.1 | Lu,X., Rowe,L.A., Frace,M., Stevens,J., Abedi,G.R., El Nile,O., Banassir,T., Al-Masri,M., Watson,J.T., Assiri,A., Erdman,D.D. | 2016-08-12T00:00:00Z | ssRNA(+) | 1353 | GenBank | S protein | Saudi Arabia | Homo sapiens | 04/11/2014 |
| AMQ49015.1 | Assiri,A.M., Biggs,H.M., Abedi,G.R., Saeed,A.B., Abdalla,O., Mohammed,M., Algarni,H.S., Alhakeem,R.F., Lu,X., Almasri,M.M., Alomar,I.A., Alsharef,A.A., Nooh,R., Erdman,D.D., Gerber,S.I., Watson,J.T. | 2016-06-27T00:00:00Z | ssRNA(+) | 1353 | GenBank | S protein | Saudi Arabia | Homo sapiens | 12/07/2015 |
| AMQ49026.1 | Assiri,A.M., Biggs,H.M., Abedi,G.R., Saeed,A.B., Abdalla,O., Mohammed,M., Algarni,H.S., Alhakeem,R.F., Lu,X., Almasri,M.M., Alomar,I.A., Alsharef,A.A., Nooh,R., Erdman,D.D., Gerber,S.I., Watson,J.T. | 2016-06-27T00:00:00Z | ssRNA(+) | 1353 | GenBank | S protein | Saudi Arabia | Homo sapiens | 24/08/2015 |
| AMQ49037.1 | Assiri,A.M., Biggs,H.M., Abedi,G.R., Saeed,A.B., Abdalla,O., Mohammed,M., Algarni,H.S., Alhakeem,R.F., Lu,X., Almasri,M.M., Alomar,I.A., Alsharef,A.A., Nooh,R., Erdman,D.D., Gerber,S.I., Watson,J.T. | 2016-06-27T00:00:00Z | ssRNA(+) | 1353 | GenBank | S protein | Saudi Arabia | Homo sapiens | 24/08/2015 |
| AMQ49048.1 | Assiri,A.M., Biggs,H.M., Abedi,G.R., Saeed,A.B., Abdalla,O., Mohammed,M., Algarni,H.S., Alhakeem,R.F., Lu,X., Almasri,M.M., Alomar,I.A., Alsharef,A.A., Nooh,R., Erdman,D.D., Gerber,S.I., Watson,J.T. | 2016-06-27T00:00:00Z | ssRNA(+) | 1353 | GenBank | S protein | Saudi Arabia | Homo sapiens | 24/08/2015 |
| AMQ49059.1 | Assiri,A.M., Biggs,H.M., Abedi,G.R., Saeed,A.B., Abdalla,O., Mohammed,M., Algarni,H.S., Alhakeem,R.F., Lu,X., Almasri,M.M., Alomar,I.A., Alsharef,A.A., Nooh,R., Erdman,D.D., Gerber,S.I., Watson,J.T. | 2016-06-27T00:00:00Z | ssRNA(+) | 1353 | GenBank | S protein | Saudi Arabia | Homo sapiens | 27/08/2015 |
| AMQ49070.1 | Assiri,A.M., Biggs,H.M., Abedi,G.R., Saeed,A.B., Abdalla,O., Mohammed,M., Algarni,H.S., Alhakeem,R.F., Lu,X., Almasri,M.M., Alomar,I.A., Alsharef,A.A., Nooh,R., Erdman,D.D., Gerber,S.I., Watson,J.T. | 2016-06-27T00:00:00Z | ssRNA(+) | 1353 | GenBank | S protein | Saudi Arabia | Homo sapiens | 24/08/2015 |
| AML60270.1 | Park,W.B., Kwon,N.J., Choe,P.G., Choi,S.J., Oh,H.S., Lee,S.M., Chong,H., Kim,J.I., Song,K.H., Bang,J.H., Kim,E.S., Kim,H.B., Park,S.W., Kim,N.J., Oh,M.D., Chong,H.Y., Kwon,N.-J., Song,K.-H., Kim,H.-B., Oh,M.-D., Seo,J.-S. | 2016-03-01T00:00:00Z | ssRNA(+) | 1353 | GenBank | S protein | South Korea | Homo sapiens | 08/06/2015 |
| ALW82636.1 | Lu,X., Assiri,A.M., Erdman,D.D. | 2016-01-13T00:00:00Z | ssRNA(+) | 1353 | GenBank | S protein | Saudi Arabia | Homo sapiens | 09/02/2015 |
| ALW82647.1 | Lu,X., Assiri,A.M., Erdman,D.D. | 2016-01-13T00:00:00Z | ssRNA(+) | 1353 | GenBank | S protein | Saudi Arabia | Homo sapiens | 22/02/2015 |
| ALW82658.1 | Lu,X., Assiri,A.M., Erdman,D.D. | 2016-01-13T00:00:00Z | ssRNA(+) | 1353 | GenBank | S protein | Saudi Arabia | Homo sapiens | 10/05/2015 |
| ALW82669.1 | Lu,X., Assiri,A.M., Erdman,D.D. | 2016-01-13T00:00:00Z | ssRNA(+) | 1353 | GenBank | S protein | Saudi Arabia | Homo sapiens | 27/03/2015 |
| ALW82680.1 | Lu,X., Assiri,A.M., Erdman,D.D. | 2016-01-13T00:00:00Z | ssRNA(+) | 1353 | GenBank | S protein | Saudi Arabia | Homo sapiens | 07/02/2015 |
| ALW82691.1 | Lu,X., Assiri,A.M., Erdman,D.D. | 2016-01-13T00:00:00Z | ssRNA(+) | 1353 | GenBank | S protein | Saudi Arabia | Homo sapiens | 15/02/2015 |
| ALW82702.1 | Lu,X., Assiri,A.M., Erdman,D.D. | 2016-01-13T00:00:00Z | ssRNA(+) | 1353 | GenBank | S protein | Saudi Arabia | Homo sapiens | 12/02/2015 |
| ALW82709.1 | Lu,X., Assiri,A.M., Erdman,D.D. | 2016-01-13T00:00:00Z | ssRNA(+) | 1353 | GenBank | S protein | Saudi Arabia | Homo sapiens | 05/02/2015 |
| ALW82720.1 | Lu,X., Assiri,A.M., Erdman,D.D. | 2016-01-13T00:00:00Z | ssRNA(+) | 1353 | GenBank | S protein | Saudi Arabia | Homo sapiens | 02/02/2015 |
| ALW82731.1 | Lu,X., Assiri,A.M., Erdman,D.D. | 2016-01-13T00:00:00Z | ssRNA(+) | 1353 | GenBank | S protein | Saudi Arabia | Homo sapiens | 02/02/2015 |
| ALW82742.1 | Lu,X., Assiri,A.M., Erdman,D.D. | 2016-01-13T00:00:00Z | ssRNA(+) | 1353 | GenBank | S protein | Saudi Arabia | Homo sapiens | 13/02/2015 |
| ALW82753.1 | Lu,X., Assiri,A.M., Erdman,D.D. | 2016-01-13T00:00:00Z | ssRNA(+) | 1353 | GenBank | S protein | Saudi Arabia | Homo sapiens | 10/02/2015 |
| ALB08246.1 | Seong,M.W., Kim,S.Y., Corman,V.M., Kim,T.S., Cho,S.I., Kim,M.J., Lee,S.J., Lee,J.S., Seo,S.H., Ahn,J.S., Yu,B.S., Park,N., Oh,M.D., Park,W.B., Lee,J.Y., Kim,G., Joh,J.S., Jeong,I., Kim,E.C., Drosten,C., Park,S.S., Seong,M.-W., Lee,J.-S., Oh,M.-D. | 2015-11-24T00:00:00Z | ssRNA(+) | 1353 | GenBank | S protein | South Korea | Homo sapiens | 29/06/2015 |
| ALB08257.1 | Seong,M.W., Kim,S.Y., Corman,V.M., Kim,T.S., Cho,S.I., Kim,M.J., Lee,S.J., Lee,J.S., Seo,S.H., Ahn,J.S., Yu,B.S., Park,N., Oh,M.D., Park,W.B., Lee,J.Y., Kim,G., Joh,J.S., Jeong,I., Kim,E.C., Drosten,C., Park,S.S., Seong,M.-W., Lee,J.-S., Oh,M.-D. | 2015-11-24T00:00:00Z | ssRNA(+) | 1353 | GenBank | S protein | South Korea | Homo sapiens | 19/06/2015 |
| ALB08267.1 | Seong,M.W., Kim,S.Y., Corman,V.M., Kim,T.S., Cho,S.I., Kim,M.J., Lee,S.J., Lee,J.S., Seo,S.H., Ahn,J.S., Yu,B.S., Park,N., Oh,M.D., Park,W.B., Lee,J.Y., Kim,G., Joh,J.S., Jeong,I., Kim,E.C., Drosten,C., Park,S.S., Seong,M.-W., Lee,J.-S., Oh,M.-D. | 2015-11-24T00:00:00Z | ssRNA(+) | 1353 | GenBank | S protein | South Korea | Homo sapiens | 31/05/2015 |
| ALB08278.1 | Seong,M.W., Kim,S.Y., Corman,V.M., Kim,T.S., Cho,S.I., Kim,M.J., Lee,S.J., Lee,J.S., Seo,S.H., Ahn,J.S., Yu,B.S., Park,N., Oh,M.D., Park,W.B., Lee,J.Y., Kim,G., Joh,J.S., Jeong,I., Kim,E.C., Drosten,C., Park,S.S., Seong,M.-W., Lee,J.-S., Oh,M.-D. | 2015-11-24T00:00:00Z | ssRNA(+) | 1353 | GenBank | S protein | South Korea | Homo sapiens | 13/06/2015 |
| ALB08289.1 | Seong,M.W., Kim,S.Y., Corman,V.M., Kim,T.S., Cho,S.I., Kim,M.J., Lee,S.J., Lee,J.S., Seo,S.H., Ahn,J.S., Yu,B.S., Park,N., Oh,M.D., Park,W.B., Lee,J.Y., Kim,G., Joh,J.S., Jeong,I., Kim,E.C., Drosten,C., Park,S.S., Seong,M.-W., Lee,J.-S., Oh,M.-D. | 2015-11-24T00:00:00Z | ssRNA(+) | 1353 | GenBank | S protein | South Korea | Homo sapiens | 03/06/2015 |
| ALB08300.1 | Seong,M.W., Kim,S.Y., Corman,V.M., Kim,T.S., Cho,S.I., Kim,M.J., Lee,S.J., Lee,J.S., Seo,S.H., Ahn,J.S., Yu,B.S., Park,N., Oh,M.D., Park,W.B., Lee,J.Y., Kim,G., Joh,J.S., Jeong,I., Kim,E.C., Drosten,C., Park,S.S., Seong,M.-W., Lee,J.-S., Oh,M.-D. | 2015-11-24T00:00:00Z | ssRNA(+) | 1353 | GenBank | S protein | South Korea | Homo sapiens | 18/06/2015 |
| ALB08311.1 | Seong,M.W., Kim,S.Y., Corman,V.M., Kim,T.S., Cho,S.I., Kim,M.J., Lee,S.J., Lee,J.S., Seo,S.H., Ahn,J.S., Yu,B.S., Park,N., Oh,M.D., Park,W.B., Lee,J.Y., Kim,G., Joh,J.S., Jeong,I., Kim,E.C., Drosten,C., Park,S.S., Seong,M.-W., Lee,J.-S., Oh,M.-D. | 2015-11-24T00:00:00Z | ssRNA(+) | 1353 | GenBank | S protein | South Korea | Homo sapiens | 21/06/2015 |
| ALB08322.1 | Seong,M.W., Kim,S.Y., Corman,V.M., Kim,T.S., Cho,S.I., Kim,M.J., Lee,S.J., Lee,J.S., Seo,S.H., Ahn,J.S., Yu,B.S., Park,N., Oh,M.D., Park,W.B., Lee,J.Y., Kim,G., Joh,J.S., Jeong,I., Kim,E.C., Drosten,C., Park,S.S., Seong,M.-W., Lee,J.-S., Oh,M.-D. | 2015-11-24T00:00:00Z | ssRNA(+) | 1353 | GenBank | S protein | South Korea | Homo sapiens | 24/06/2015 |
| ALM26400.1 | Lu,R., Wang,Y., Zhao,Y., Tan,W. | 2015-11-09T00:00:00Z | ssRNA(+) | 1353 | GenBank | S protein | China | Homo sapiens | 27/05/2015 |
| ALD51904.1 | Plipat,T., Buathong,R., Wacharapluesadee,S., Siriarayapon,P., Pittayawonganon,C., Sangsajja,C., Kaewpom,T., Petcharat,S., Ponpinit,T., Jumpasri,J., Joyjinda,Y., Rodpan,A., Ghai,S., Jittmittraphap,A., Khongwichit,S., Smith,D.R., Corman,V.M., Drosten,C., Hemachudha,T., Pittayawomganon,C., Sangsaija,C., Jittmitraphap,A. | 2015-09-10T00:00:00Z | ssRNA(+) | 1353 | GenBank | S protein | Thailand | Homo sapiens | 17/06/2015 |
| AKM76229.1 | Queen,K., Al-Jardani,A., Zhang,J., Al Kindi,H., Li,Y., Tao,Y., Al Baqlani,S., Al Mahrouqi,S., Tong,S. | 2015-06-23T00:00:00Z | ssRNA(+) | 1353 | GenBank | S protein | Oman | Homo sapiens | 28/10/2013 |
| AKM76239.1 | Queen,K., Al-Jardani,A., Zhang,J., Al Kindi,H., Li,Y., Tao,Y., Al Baqlani,S., Al Mahrouqi,S., Tong,S. | 2015-06-23T00:00:00Z | ssRNA(+) | 1353 | GenBank | S protein | Oman | Homo sapiens | 28/12/2013 |
| AKL80593.1 | Su,J., Xu,J., Zhang,B., Xie,Q., Zhao,W., Ke,C. | 2015-06-10T00:00:00Z | ssRNA(+) | 1353 | GenBank | S protein | China | Homo sapiens | 28/05/2015 |
| AKL80604.1 | Su,J., Wu,J., Zhang,B., Xie,Q., Zhao,W., Ke,C. | 2015-06-10T00:00:00Z | ssRNA(+) | 1353 | GenBank | S protein | China | Homo sapiens | 28/05/2015 |
| AKL80615.1 | Su,J., Wu,J., Zhang,B., Xie,Q., Zhao,W., Ke,C. | 2015-06-10T00:00:00Z | ssRNA(+) | 1353 | GenBank | S protein | China | Homo sapiens | 28/05/2015 |
| AKK52582.1 | Park,W.B., Kwon,N.J., Choe,P.G., Choi,S.J., Oh,H.S., Lee,S.M., Chong,H., Kim,J.I., Song,K.H., Bang,J.H., Kim,E.S., Kim,H.B., Park,S.W., Kim,N.J., Oh,M.D., Queen,K., Bin Saeed,A., Paden,C., Zhang,J., Li,Y., Tao,Y., Erdman,D., Tong,S. | 2015-06-08T00:00:00Z | ssRNA(+) | 1353 | GenBank | S protein | Saudi Arabia | Homo sapiens | 10/02/2015 |
| AKK52592.1 | Park,W.B., Kwon,N.J., Choe,P.G., Choi,S.J., Oh,H.S., Lee,S.M., Chong,H., Kim,J.I., Song,K.H., Bang,J.H., Kim,E.S., Kim,H.B., Park,S.W., Kim,N.J., Oh,M.D., Queen,K., Bin Saeed,A., Paden,C., Zhang,J., Li,Y., Tao,Y., Erdman,D., Tong,S. | 2015-06-08T00:00:00Z | ssRNA(+) | 1353 | GenBank | S protein | Saudi Arabia | Homo sapiens | 01/03/2015 |
| AKK52602.1 | Park,W.B., Kwon,N.J., Choe,P.G., Choi,S.J., Oh,H.S., Lee,S.M., Chong,H., Kim,J.I., Song,K.H., Bang,J.H., Kim,E.S., Kim,H.B., Park,S.W., Kim,N.J., Oh,M.D., Queen,K., Tamin,A., Bin Saeed,A., Paden,C., Zhang,J., Li,Y., Tao,Y., Erdman,D., Tong,S. | 2015-06-08T00:00:00Z | ssRNA(+) | 1353 | GenBank | S protein | Saudi Arabia | Homo sapiens | 10/02/2015 |
| AKK52612.1 | Park,W.B., Kwon,N.J., Choe,P.G., Choi,S.J., Oh,H.S., Lee,S.M., Chong,H., Kim,J.I., Song,K.H., Bang,J.H., Kim,E.S., Kim,H.B., Park,S.W., Kim,N.J., Oh,M.D., Queen,K., Tamin,A., Bin Saeed,A., Paden,C., Zhang,J., Li,Y., Tao,Y., Erdman,D., Tong,S. | 2015-06-08T00:00:00Z | ssRNA(+) | 1353 | GenBank | S protein | Saudi Arabia | Homo sapiens | 01/03/2015 |
| AKJ80137.2 | Lu,R., Wang,Y., Wang,W., Nie,K., Zhao,Y., Su,J., Deng,Y., Zhou,W., Li,Y., Wang,H., Ke,C., Ma,X., Wu,G., Tan,W., Zhang,Y., Zou,L., Niu,P., Zhang,S., Wu,J. | 2015-06-05T00:00:00Z | ssRNA(+) | 1353 | GenBank | S protein | China | Homo sapiens | 27/05/2015 |
| AKI29255.1 | Lu,X., Saeed,A.A.B., Erdman,D.D. | 2015-05-27T00:00:00Z | ssRNA(+) | 1353 | GenBank | S protein | Saudi Arabia | Homo sapiens | 21/01/2015 |
| AKI29265.1 | Lu,X., Saeed,A.A.B., Erdman,D.D. | 2015-05-27T00:00:00Z | ssRNA(+) | 1353 | GenBank | S protein | Saudi Arabia | Homo sapiens | 21/01/2015 |
| AKI29275.1 | Lu,X., Saeed,A.A.B., Erdman,D.D. | 2015-05-27T00:00:00Z | ssRNA(+) | 1353 | GenBank | S protein | Saudi Arabia | Homo sapiens | 26/01/2015 |
| AKI29284.1 | Lu,X., Saeed,A.A.B., Erdman,D.D. | 2015-05-27T00:00:00Z | ssRNA(+) | 1353 | GenBank | S protein | Saudi Arabia | Homo sapiens | 06/01/2015 |
| AJD81440.1 | Galiano,M., Myers,R., Bermingham,A., Gopal,R., Zambon,M. | 2015-01-18T00:00:00Z | ssRNA(+) | 1353 | GenBank | S protein | United Kingdom | Homo sapiens | 13/02/2013 |
| AJD81451.1 | Galiano,M., Myers,R., Bermingham,A., Gopal,R., Zambon,M. | 2015-01-18T00:00:00Z | ssRNA(+) | 1353 | GenBank | S protein | United Kingdom | Homo sapiens | 10/02/2013 |
| AIZ48760.1 | Tao,Y., Tamin,A., Paden,C., Queen,K., Li,Y., Tong,S. | 2014-12-14T00:00:00Z | ssRNA(+) | 1353 | GenBank | S protein | USA | Homo sapiens | 2014-06 |
| AIZ48769.1 | Tao,Y., Queen,K., Li,Y., Paden,C.R., Zhang,J., Al-Ameri,A., Amer,W.A., AlHamly,N., Weber,S., Imambaccus,H., Sasse,J., Gerber,S., Al Hosany,F.I., Tong,S. | 2014-12-14T00:00:00Z | ssRNA(+) | 1353 | GenBank | S protein | United Arab Emirates | Homo sapiens | 29/10/2013 |
| AIY60518.1 | Hunter,J.C., Nguyen,D., Aden,B., Al Bandar,Z., Al Dhaheri,W., Abu Elkheir,K., Khudair,A., Al Mulla,M., El Saleh,F., Imambaccus,H., Al Kaabi,N., Sheikh,F.A., Sasse,J., Turner,A., Abdel Wareth,L., Weber,S., Al Ameri,A., Abu Amer,W., Alami,N.N., Bunga,S., Haynes,L.M., Hall,A.J., Kallen,A.J., Kuhar,D., Pham,H., Pringle,K., Tong,S., Whitaker,B.L., Gerber,S.I., Al Hosani,F.I., Tao,Y., Queen,K., Li,Y., Paden,C., Zhang,J., Al-Ameri,A., Amer,W.A., AlHamly,N., Gerber,S., Al Hosany,F.I. | 2014-12-06T00:00:00Z | ssRNA(+) | 1353 | GenBank | S protein | United Arab Emirates | Homo sapiens | 07/04/2014 |
| AIY60528.1 | Hunter,J.C., Nguyen,D., Aden,B., Al Bandar,Z., Al Dhaheri,W., Abu Elkheir,K., Khudair,A., Al Mulla,M., El Saleh,F., Imambaccus,H., Al Kaabi,N., Sheikh,F.A., Sasse,J., Turner,A., Abdel Wareth,L., Weber,S., Al Ameri,A., Abu Amer,W., Alami,N.N., Bunga,S., Haynes,L.M., Hall,A.J., Kallen,A.J., Kuhar,D., Pham,H., Pringle,K., Tong,S., Whitaker,B.L., Gerber,S.I., Al Hosani,F.I., Tao,Y., Queen,K., Li,Y., Paden,C., Zhang,J., Al-Ameri,A., Amer,W.A., AlHamly,N., Gerber,S., Al Hosany,F.I. | 2014-12-06T00:00:00Z | ssRNA(+) | 1353 | GenBank | S protein | United Arab Emirates | Homo sapiens | 10/04/2014 |
| AIY60538.1 | Hunter,J.C., Nguyen,D., Aden,B., Al Bandar,Z., Al Dhaheri,W., Abu Elkheir,K., Khudair,A., Al Mulla,M., El Saleh,F., Imambaccus,H., Al Kaabi,N., Sheikh,F.A., Sasse,J., Turner,A., Abdel Wareth,L., Weber,S., Al Ameri,A., Abu Amer,W., Alami,N.N., Bunga,S., Haynes,L.M., Hall,A.J., Kallen,A.J., Kuhar,D., Pham,H., Pringle,K., Tong,S., Whitaker,B.L., Gerber,S.I., Al Hosani,F.I., Tao,Y., Queen,K., Li,Y., Paden,C., Zhang,J., Al-Ameri,A., Amer,W.A., AlHamly,N., Gerber,S., Al Hosany,F.I. | 2014-12-06T00:00:00Z | ssRNA(+) | 1353 | GenBank | S protein | United Arab Emirates | Homo sapiens | 10/04/2014 |
| AIY60548.1 | Hunter,J.C., Nguyen,D., Aden,B., Al Bandar,Z., Al Dhaheri,W., Abu Elkheir,K., Khudair,A., Al Mulla,M., El Saleh,F., Imambaccus,H., Al Kaabi,N., Sheikh,F.A., Sasse,J., Turner,A., Abdel Wareth,L., Weber,S., Al Ameri,A., Abu Amer,W., Alami,N.N., Bunga,S., Haynes,L.M., Hall,A.J., Kallen,A.J., Kuhar,D., Pham,H., Pringle,K., Tong,S., Whitaker,B.L., Gerber,S.I., Al Hosani,F.I., Tao,Y., Queen,K., Li,Y., Paden,C., Zhang,J., Al-Ameri,A., Amer,W.A., AlHamly,N., Gerber,S., Al Hosany,F.I. | 2014-12-06T00:00:00Z | ssRNA(+) | 1353 | GenBank | S protein | United Arab Emirates | Homo sapiens | 19/04/2014 |
| AIY60558.1 | Hunter,J.C., Nguyen,D., Aden,B., Al Bandar,Z., Al Dhaheri,W., Abu Elkheir,K., Khudair,A., Al Mulla,M., El Saleh,F., Imambaccus,H., Al Kaabi,N., Sheikh,F.A., Sasse,J., Turner,A., Abdel Wareth,L., Weber,S., Al Ameri,A., Abu Amer,W., Alami,N.N., Bunga,S., Haynes,L.M., Hall,A.J., Kallen,A.J., Kuhar,D., Pham,H., Pringle,K., Tong,S., Whitaker,B.L., Gerber,S.I., Al Hosani,F.I., Tao,Y., Queen,K., Li,Y., Paden,C., Zhang,J., Al-Ameri,A., Amer,W.A., AlHamly,N., Gerber,S., Al Hosany,F.I. | 2014-12-06T00:00:00Z | ssRNA(+) | 1353 | GenBank | S protein | United Arab Emirates | Homo sapiens | 07/03/2014 |
| AIY60568.1 | Hunter,J.C., Nguyen,D., Aden,B., Al Bandar,Z., Al Dhaheri,W., Abu Elkheir,K., Khudair,A., Al Mulla,M., El Saleh,F., Imambaccus,H., Al Kaabi,N., Sheikh,F.A., Sasse,J., Turner,A., Abdel Wareth,L., Weber,S., Al Ameri,A., Abu Amer,W., Alami,N.N., Bunga,S., Haynes,L.M., Hall,A.J., Kallen,A.J., Kuhar,D., Pham,H., Pringle,K., Tong,S., Whitaker,B.L., Gerber,S.I., Al Hosani,F.I., Tao,Y., Queen,K., Li,Y., Paden,C., Zhang,J., Al-Ameri,A., Amer,W.A., AlHamly,N., Gerber,S., Al Hosany,F.I. | 2014-12-06T00:00:00Z | ssRNA(+) | 1353 | GenBank | S protein | United Arab Emirates | Homo sapiens | 17/04/2014 |
| AIY60578.1 | Hunter,J.C., Nguyen,D., Aden,B., Al Bandar,Z., Al Dhaheri,W., Abu Elkheir,K., Khudair,A., Al Mulla,M., El Saleh,F., Imambaccus,H., Al Kaabi,N., Sheikh,F.A., Sasse,J., Turner,A., Abdel Wareth,L., Weber,S., Al Ameri,A., Abu Amer,W., Alami,N.N., Bunga,S., Haynes,L.M., Hall,A.J., Kallen,A.J., Kuhar,D., Pham,H., Pringle,K., Tong,S., Whitaker,B.L., Gerber,S.I., Al Hosani,F.I., Tao,Y., Queen,K., Li,Y., Paden,C., Zhang,J., Al-Ameri,A., Amer,W.A., AlHamly,N., Gerber,S., Al Hosany,F.I. | 2014-12-06T00:00:00Z | ssRNA(+) | 1353 | GenBank | S protein | United Arab Emirates | Homo sapiens | 15/11/2013 |
| AIY60588.1 | Hunter,J.C., Nguyen,D., Aden,B., Al Bandar,Z., Al Dhaheri,W., Abu Elkheir,K., Khudair,A., Al Mulla,M., El Saleh,F., Imambaccus,H., Al Kaabi,N., Sheikh,F.A., Sasse,J., Turner,A., Abdel Wareth,L., Weber,S., Al Ameri,A., Abu Amer,W., Alami,N.N., Bunga,S., Haynes,L.M., Hall,A.J., Kallen,A.J., Kuhar,D., Pham,H., Pringle,K., Tong,S., Whitaker,B.L., Gerber,S.I., Al Hosani,F.I., Tao,Y., Queen,K., Li,Y., Paden,C., Zhang,J., Al-Ameri,A., Amer,W.A., AlHamly,N., Gerber,S., Al Hosany,F.I. | 2014-12-06T00:00:00Z | ssRNA(+) | 1353 | GenBank | S protein | United Arab Emirates | Homo sapiens | 13/04/2014 |
| AID50418.1 | Galiano,M., Myers,R., Bermingham,A., Gopal,R., Zambon,M. | 2014-06-18T00:00:00Z | ssRNA(+) | 1353 | GenBank | S protein | United Kingdom | Homo sapiens | 10/02/2013 |
| AHZ64057.1 | Tao,Y., Queen,K., Li,Y., Lu,X., Sakthivel,S., Gerber,S.I., Cannons,A., Crespo,A., Matthews,S.D., Heberlein-Larson,L., Aguilera,A., Erdman,D., Tong,S. | 2014-05-14T00:00:00Z | ssRNA(+) | 1353 | GenBank | S protein | USA | Homo sapiens | 10/05/2014 |
| AHZ20790.1 | Tsiodras,S., Baka,A., Mentis,A., Iliopoulos,D., Dedoukou,X., Papamavrou,G., Karadima,S., Emmanouil,M., Kossyvakis,A., Spanakis,N., Pavli,A., Maltezou,H., Karageorgou,A., Spala,G., Pitiriga,V., Kosmas,E., Tsiagklis,S., Gkatzias,S., Koulouris,N., Koutsoukou,A., Bakakos,P., Markozanhs,E., Dionellis,G., Pontikis,K., Rovina,N., Kyriakopoulou,M., Efstathiou,P., Papadimitriou,T., Kremastinou,J., Tsakris,A., Saroglou,G., Tao,Y., Lu,X., Kossivakis,A., Pogka,V., Tong,S., Erdman,D., Antoniadis,A. | 2014-05-13T00:00:00Z | ssRNA(+) | 1353 | GenBank | S protein | Greece | Homo sapiens | 18/04/2014 |
| AHZ58501.1 | Kapoor,M., Pringle,K., Kumar,A., Dearth,S., Liu,L., Lovchik,J., Perez,O., Pontones,P., Richards,S., Yeadon-Fagbohun,J., Breakwell,L., Chea,N., Cohen,N.J., Schneider,E., Erdman,D., Haynes,L., Pallansch,M., Tao,Y., Tong,S., Gerber,S., Swerdlow,D., Feikin,D.R., Li,Y., Lu,X., Dalton,L., Queen,K., Sakthivel,S., Paden,C., Patel,P., Gerber,S.I. | 2014-05-13T00:00:00Z | ssRNA(+) | 1353 | GenBank | S protein | USA | Homo sapiens | 30/04/2014 |
| AHN10812.1 | Memish,Z.A., Cotten,M., Meyer,B., Watson,S.J., Alsahafi,A.J., Al Rabeeah,A.A., Corman,V.M., Sieberg,A., Makhdoom,H.Q., Assiri,A., Al Masri,M., Aldabbagh,S., Bosch,B.J., Beer,M., Muller,M.A., Kellam,P., Drosten,C., Palser,A.L., Zumla,A. | 2014-03-24T00:00:00Z | ssRNA(+) | 1353 | GenBank | S protein | Saudi Arabia | Homo sapiens | 06/11/2013 |
| AHI48517.1 | Cotten,M., Watson,S.J., Zumla,A.I., Makhdoom,H.Q., Palser,A.L., Ong,S.H., Al Rabeeah,A.A., Alhakeem,R.F., Assiri,A., Al-Tawfiq,J.A., Albarrak,A., Barry,M., Shibl,A., Alrabiah,F.A., Hajjar,S., Balkhy,H.H., Flemban,H., Rambaut,A., Kellam,P., Memish,Z.A., Zumla,A. | 2014-02-06T00:00:00Z | ssRNA(+) | 1353 | GenBank | S protein | Saudi Arabia | Homo sapiens | 02/05/2013 |
| AHI48528.1 | Cotten,M., Watson,S.J., Zumla,A.I., Makhdoom,H.Q., Palser,A.L., Ong,S.H., Al Rabeeah,A.A., Alhakeem,R.F., Assiri,A., Al-Tawfiq,J.A., Albarrak,A., Barry,M., Shibl,A., Alrabiah,F.A., Hajjar,S., Balkhy,H.H., Flemban,H., Rambaut,A., Kellam,P., Memish,Z.A., Zumla,A. | 2014-02-06T00:00:00Z | ssRNA(+) | 1353 | GenBank | S protein | Saudi Arabia | Homo sapiens | 17/07/2013 |
| AHI48539.1 | Cotten,M., Watson,S.J., Zumla,A.I., Makhdoom,H.Q., Palser,A.L., Ong,S.H., Al Rabeeah,A.A., Alhakeem,R.F., Assiri,A., Al-Tawfiq,J.A., Albarrak,A., Barry,M., Shibl,A., Alrabiah,F.A., Hajjar,S., Balkhy,H.H., Flemban,H., Rambaut,A., Kellam,P., Memish,Z.A., Zumla,A. | 2014-02-06T00:00:00Z | ssRNA(+) | 1353 | GenBank | S protein | Saudi Arabia | Homo sapiens | 28/08/2013 |
| AHI48550.1 | Cotten,M., Watson,S.J., Zumla,A.I., Makhdoom,H.Q., Palser,A.L., Ong,S.H., Al Rabeeah,A.A., Alhakeem,R.F., Assiri,A., Al-Tawfiq,J.A., Albarrak,A., Barry,M., Shibl,A., Alrabiah,F.A., Hajjar,S., Balkhy,H.H., Flemban,H., Rambaut,A., Kellam,P., Memish,Z.A., Zumla,A. | 2014-02-06T00:00:00Z | ssRNA(+) | 1353 | GenBank | S protein | Saudi Arabia | Homo sapiens | 12/06/2013 |
| AHI48561.1 | Cotten,M., Watson,S.J., Zumla,A.I., Makhdoom,H.Q., Palser,A.L., Ong,S.H., Al Rabeeah,A.A., Alhakeem,R.F., Assiri,A., Al-Tawfiq,J.A., Albarrak,A., Barry,M., Shibl,A., Alrabiah,F.A., Hajjar,S., Balkhy,H.H., Flemban,H., Rambaut,A., Kellam,P., Memish,Z.A., Zumla,A. | 2014-02-06T00:00:00Z | ssRNA(+) | 1353 | GenBank | S protein | Saudi Arabia | Homo sapiens | 05/08/2013 |
| AHI48572.1 | Cotten,M., Watson,S.J., Zumla,A.I., Makhdoom,H.Q., Palser,A.L., Ong,S.H., Al Rabeeah,A.A., Alhakeem,R.F., Assiri,A., Al-Tawfiq,J.A., Albarrak,A., Barry,M., Shibl,A., Alrabiah,F.A., Hajjar,S., Balkhy,H.H., Flemban,H., Rambaut,A., Kellam,P., Memish,Z.A., Zumla,A. | 2014-02-06T00:00:00Z | ssRNA(+) | 1353 | GenBank | S protein | Saudi Arabia | Homo sapiens | 15/08/2013 |
| AHI48583.1 | Cotten,M., Watson,S.J., Zumla,A.I., Makhdoom,H.Q., Palser,A.L., Ong,S.H., Al Rabeeah,A.A., Alhakeem,R.F., Assiri,A., Al-Tawfiq,J.A., Albarrak,A., Barry,M., Shibl,A., Alrabiah,F.A., Hajjar,S., Balkhy,H.H., Flemban,H., Rambaut,A., Kellam,P., Memish,Z.A., Zumla,A. | 2014-02-06T00:00:00Z | ssRNA(+) | 1353 | GenBank | S protein | Saudi Arabia | Homo sapiens | 02/07/2013 |
| AHI48594.1 | Cotten,M., Watson,S.J., Zumla,A.I., Makhdoom,H.Q., Palser,A.L., Ong,S.H., Al Rabeeah,A.A., Alhakeem,R.F., Assiri,A., Al-Tawfiq,J.A., Albarrak,A., Barry,M., Shibl,A., Alrabiah,F.A., Hajjar,S., Balkhy,H.H., Flemban,H., Rambaut,A., Kellam,P., Memish,Z.A., Zumla,A. | 2014-02-06T00:00:00Z | ssRNA(+) | 1353 | GenBank | S protein | Saudi Arabia | Homo sapiens | 12/06/2013 |
| AHI48605.1 | Cotten,M., Watson,S.J., Zumla,A.I., Makhdoom,H.Q., Palser,A.L., Ong,S.H., Al Rabeeah,A.A., Alhakeem,R.F., Assiri,A., Al-Tawfiq,J.A., Albarrak,A., Barry,M., Shibl,A., Alrabiah,F.A., Hajjar,S., Balkhy,H.H., Flemban,H., Rambaut,A., Kellam,P., Memish,Z.A., Zumla,A. | 2014-02-06T00:00:00Z | ssRNA(+) | 1353 | GenBank | S protein | Saudi Arabia | Homo sapiens | 01/03/2013 |
| AHI48616.1 | Cotten,M., Watson,S.J., Zumla,A.I., Makhdoom,H.Q., Palser,A.L., Ong,S.H., Al Rabeeah,A.A., Alhakeem,R.F., Assiri,A., Al-Tawfiq,J.A., Albarrak,A., Barry,M., Shibl,A., Alrabiah,F.A., Hajjar,S., Balkhy,H.H., Flemban,H., Rambaut,A., Kellam,P., Memish,Z.A., Zumla,A. | 2014-02-06T00:00:00Z | ssRNA(+) | 1353 | GenBank | S protein | Saudi Arabia | Homo sapiens | 11/09/2013 |
| AHI48626.1 | Cotten,M., Watson,S.J., Zumla,A.I., Makhdoom,H.Q., Palser,A.L., Ong,S.H., Al Rabeeah,A.A., Alhakeem,R.F., Assiri,A., Al-Tawfiq,J.A., Albarrak,A., Barry,M., Shibl,A., Alrabiah,F.A., Hajjar,S., Balkhy,H.H., Flemban,H., Rambaut,A., Kellam,P., Memish,Z.A., Zumla,A. | 2014-02-06T00:00:00Z | ssRNA(+) | 1353 | GenBank | S protein | Saudi Arabia | Homo sapiens | 17/07/2013 |
| AHI48652.1 | Cotten,M., Watson,S.J., Zumla,A.I., Makhdoom,H.Q., Palser,A.L., Ong,S.H., Al Rabeeah,A.A., Alhakeem,R.F., Assiri,A., Al-Tawfiq,J.A., Albarrak,A., Barry,M., Shibl,A., Alrabiah,F.A., Hajjar,S., Balkhy,H.H., Flemban,H., Rambaut,A., Kellam,P., Memish,Z.A., Zumla,A. | 2014-02-06T00:00:00Z | ssRNA(+) | 1353 | GenBank | S protein | Saudi Arabia | Homo sapiens | 18/06/2013 |
| AHI48662.1 | Cotten,M., Watson,S.J., Zumla,A.I., Makhdoom,H.Q., Palser,A.L., Ong,S.H., Al Rabeeah,A.A., Alhakeem,R.F., Assiri,A., Al-Tawfiq,J.A., Albarrak,A., Barry,M., Shibl,A., Alrabiah,F.A., Hajjar,S., Balkhy,H.H., Flemban,H., Rambaut,A., Kellam,P., Memish,Z.A., Zumla,A. | 2014-02-06T00:00:00Z | ssRNA(+) | 1353 | GenBank | S protein | Saudi Arabia | Homo sapiens | 13/08/2013 |
| AHI48672.1 | Cotten,M., Watson,S.J., Zumla,A.I., Makhdoom,H.Q., Palser,A.L., Ong,S.H., Al Rabeeah,A.A., Alhakeem,R.F., Assiri,A., Al-Tawfiq,J.A., Albarrak,A., Barry,M., Shibl,A., Alrabiah,F.A., Hajjar,S., Balkhy,H.H., Flemban,H., Rambaut,A., Kellam,P., Memish,Z.A., Zumla,A. | 2014-02-06T00:00:00Z | ssRNA(+) | 1353 | GenBank | S protein | Saudi Arabia | Homo sapiens | 12/06/2013 |
| AHI48682.1 | Cotten,M., Watson,S.J., Zumla,A.I., Makhdoom,H.Q., Palser,A.L., Ong,S.H., Al Rabeeah,A.A., Alhakeem,R.F., Assiri,A., Al-Tawfiq,J.A., Albarrak,A., Barry,M., Shibl,A., Alrabiah,F.A., Hajjar,S., Balkhy,H.H., Flemban,H., Rambaut,A., Kellam,P., Memish,Z.A., Zumla,A. | 2014-02-06T00:00:00Z | ssRNA(+) | 1353 | GenBank | S protein | Saudi Arabia | Homo sapiens | 08/08/2013 |
| AHI48692.1 | Cotten,M., Watson,S.J., Zumla,A.I., Makhdoom,H.Q., Palser,A.L., Ong,S.H., Al Rabeeah,A.A., Alhakeem,R.F., Assiri,A., Al-Tawfiq,J.A., Albarrak,A., Barry,M., Shibl,A., Alrabiah,F.A., Hajjar,S., Balkhy,H.H., Flemban,H., Rambaut,A., Kellam,P., Memish,Z.A., Zumla,A. | 2014-02-06T00:00:00Z | ssRNA(+) | 1353 | GenBank | S protein | Saudi Arabia | Homo sapiens | 15/07/2013 |
| AHI48702.1 | Cotten,M., Watson,S.J., Zumla,A.I., Makhdoom,H.Q., Palser,A.L., Ong,S.H., Al Rabeeah,A.A., Alhakeem,R.F., Assiri,A., Al-Tawfiq,J.A., Albarrak,A., Barry,M., Shibl,A., Alrabiah,F.A., Hajjar,S., Balkhy,H.H., Flemban,H., Rambaut,A., Kellam,P., Memish,Z.A., Zumla,A. | 2014-02-06T00:00:00Z | ssRNA(+) | 1353 | GenBank | S protein | Saudi Arabia | Homo sapiens | 06/08/2013 |
| AHI48711.1 | Cotten,M., Watson,S.J., Zumla,A.I., Makhdoom,H.Q., Palser,A.L., Ong,S.H., Al Rabeeah,A.A., Alhakeem,R.F., Assiri,A., Al-Tawfiq,J.A., Albarrak,A., Barry,M., Shibl,A., Alrabiah,F.A., Hajjar,S., Balkhy,H.H., Flemban,H., Rambaut,A., Kellam,P., Memish,Z.A., Zumla,A. | 2014-02-06T00:00:00Z | ssRNA(+) | 1353 | GenBank | S protein | Saudi Arabia | Homo sapiens | 19/06/2013 |
| AHI48721.1 | Cotten,M., Watson,S.J., Zumla,A.I., Makhdoom,H.Q., Palser,A.L., Ong,S.H., Al Rabeeah,A.A., Alhakeem,R.F., Assiri,A., Al-Tawfiq,J.A., Albarrak,A., Barry,M., Shibl,A., Alrabiah,F.A., Hajjar,S., Balkhy,H.H., Flemban,H., Rambaut,A., Kellam,P., Memish,Z.A., Zumla,A. | 2014-02-06T00:00:00Z | ssRNA(+) | 313 | GenBank | S protein | Saudi Arabia | Homo sapiens | 13/06/2013 |
| AHI48723.1 | Cotten,M., Watson,S.J., Zumla,A.I., Makhdoom,H.Q., Palser,A.L., Ong,S.H., Al Rabeeah,A.A., Alhakeem,R.F., Assiri,A., Al-Tawfiq,J.A., Albarrak,A., Barry,M., Shibl,A., Alrabiah,F.A., Hajjar,S., Balkhy,H.H., Flemban,H., Rambaut,A., Kellam,P., Memish,Z.A., Zumla,A. | 2014-02-06T00:00:00Z | ssRNA(+) | 250 | GenBank | S protein | Saudi Arabia | Homo sapiens | 19/08/2013 |
| AHI48725.1 | Cotten,M., Watson,S.J., Zumla,A.I., Makhdoom,H.Q., Palser,A.L., Ong,S.H., Al Rabeeah,A.A., Alhakeem,R.F., Assiri,A., Al-Tawfiq,J.A., Albarrak,A., Barry,M., Shibl,A., Alrabiah,F.A., Hajjar,S., Balkhy,H.H., Flemban,H., Rambaut,A., Kellam,P., Memish,Z.A., Zumla,A. | 2014-02-06T00:00:00Z | ssRNA(+) | 613 | GenBank | S protein | Saudi Arabia | Homo sapiens | 02/07/2013 |
| AHI48727.1 | Cotten,M., Watson,S.J., Zumla,A.I., Makhdoom,H.Q., Palser,A.L., Ong,S.H., Al Rabeeah,A.A., Alhakeem,R.F., Assiri,A., Al-Tawfiq,J.A., Albarrak,A., Barry,M., Shibl,A., Alrabiah,F.A., Hajjar,S., Balkhy,H.H., Flemban,H., Rambaut,A., Kellam,P., Memish,Z.A., Zumla,A. | 2014-02-06T00:00:00Z | ssRNA(+) | 1188 | GenBank | S protein | Saudi Arabia | Homo sapiens | 02/07/2013 |
| AHI48729.1 | Cotten,M., Watson,S.J., Zumla,A.I., Makhdoom,H.Q., Palser,A.L., Ong,S.H., Al Rabeeah,A.A., Alhakeem,R.F., Assiri,A., Al-Tawfiq,J.A., Albarrak,A., Barry,M., Shibl,A., Alrabiah,F.A., Hajjar,S., Balkhy,H.H., Flemban,H., Rambaut,A., Kellam,P., Memish,Z.A., Zumla,A. | 2014-02-06T00:00:00Z | ssRNA(+) | 966 | GenBank | S protein | Saudi Arabia | Homo sapiens | 13/05/2013 |
| AHI48731.1 | Cotten,M., Watson,S.J., Zumla,A.I., Makhdoom,H.Q., Palser,A.L., Ong,S.H., Al Rabeeah,A.A., Alhakeem,R.F., Assiri,A., Al-Tawfiq,J.A., Albarrak,A., Barry,M., Shibl,A., Alrabiah,F.A., Hajjar,S., Balkhy,H.H., Flemban,H., Rambaut,A., Kellam,P., Memish,Z.A., Zumla,A. | 2014-02-06T00:00:00Z | ssRNA(+) | 1008 | GenBank | S protein | Saudi Arabia | Homo sapiens | 22/06/2013 |
| AHI48733.1 | Cotten,M., Watson,S.J., Zumla,A.I., Makhdoom,H.Q., Palser,A.L., Ong,S.H., Al Rabeeah,A.A., Alhakeem,R.F., Assiri,A., Al-Tawfiq,J.A., Albarrak,A., Barry,M., Shibl,A., Alrabiah,F.A., Hajjar,S., Balkhy,H.H., Flemban,H., Rambaut,A., Kellam,P., Memish,Z.A., Zumla,A. | 2014-02-06T00:00:00Z | ssRNA(+) | 1179 | GenBank | S protein | Saudi Arabia | Homo sapiens | 05/08/2013 |
| AHI48735.1 | Cotten,M., Watson,S.J., Zumla,A.I., Makhdoom,H.Q., Palser,A.L., Ong,S.H., Al Rabeeah,A.A., Alhakeem,R.F., Assiri,A., Al-Tawfiq,J.A., Albarrak,A., Barry,M., Shibl,A., Alrabiah,F.A., Hajjar,S., Balkhy,H.H., Flemban,H., Rambaut,A., Kellam,P., Memish,Z.A., Zumla,A. | 2014-02-06T00:00:00Z | ssRNA(+) | 983 | GenBank | S protein | Saudi Arabia | Homo sapiens | 25/08/2013 |
| AHI48737.1 | Cotten,M., Watson,S.J., Zumla,A.I., Makhdoom,H.Q., Palser,A.L., Ong,S.H., Al Rabeeah,A.A., Alhakeem,R.F., Assiri,A., Al-Tawfiq,J.A., Albarrak,A., Barry,M., Shibl,A., Alrabiah,F.A., Hajjar,S., Balkhy,H.H., Flemban,H., Rambaut,A., Kellam,P., Memish,Z.A., Zumla,A. | 2014-02-06T00:00:00Z | ssRNA(+) | 1353 | GenBank | S protein | Saudi Arabia | Homo sapiens | 26/08/2013 |
| AHI48739.1 | Cotten,M., Watson,S.J., Zumla,A.I., Makhdoom,H.Q., Palser,A.L., Ong,S.H., Al Rabeeah,A.A., Alhakeem,R.F., Assiri,A., Al-Tawfiq,J.A., Albarrak,A., Barry,M., Shibl,A., Alrabiah,F.A., Hajjar,S., Balkhy,H.H., Flemban,H., Rambaut,A., Kellam,P., Memish,Z.A., Zumla,A. | 2014-02-06T00:00:00Z | ssRNA(+) | 1353 | GenBank | S protein | Saudi Arabia | Homo sapiens | 05/08/2013 |
| AHI48799.1 | Cotten,M., Watson,S.J., Zumla,A.I., Makhdoom,H.Q., Palser,A.L., Ong,S.H., Al Rabeeah,A.A., Alhakeem,R.F., Assiri,A., Al-Tawfiq,J.A., Albarrak,A., Barry,M., Shibl,A., Alrabiah,F.A., Hajjar,S., Balkhy,H.H., Flemban,H., Rambaut,A., Kellam,P., Memish,Z.A., Zumla,A. | 2014-02-06T00:00:00Z | ssRNA(+) | 442 | GenBank | S protein | Saudi Arabia | Homo sapiens | 19/08/2013 |
| AHI48800.1 | Cotten,M., Watson,S.J., Zumla,A.I., Makhdoom,H.Q., Palser,A.L., Ong,S.H., Al Rabeeah,A.A., Alhakeem,R.F., Assiri,A., Al-Tawfiq,J.A., Albarrak,A., Barry,M., Shibl,A., Alrabiah,F.A., Hajjar,S., Balkhy,H.H., Flemban,H., Rambaut,A., Kellam,P., Memish,Z.A., Zumla,A. | 2014-02-06T00:00:00Z | ssRNA(+) | 748 | GenBank | S protein | Saudi Arabia | Homo sapiens | 25/08/2013 |
| AHI48801.1 | Cotten,M., Watson,S.J., Zumla,A.I., Makhdoom,H.Q., Palser,A.L., Ong,S.H., Al Rabeeah,A.A., Alhakeem,R.F., Assiri,A., Al-Tawfiq,J.A., Albarrak,A., Barry,M., Shibl,A., Alrabiah,F.A., Hajjar,S., Balkhy,H.H., Flemban,H., Rambaut,A., Kellam,P., Memish,Z.A., Zumla,A. | 2014-02-06T00:00:00Z | ssRNA(+) | 700 | GenBank | S protein | Saudi Arabia | Homo sapiens | 01/05/2013 |
| AHI48802.1 | Cotten,M., Watson,S.J., Zumla,A.I., Makhdoom,H.Q., Palser,A.L., Ong,S.H., Al Rabeeah,A.A., Alhakeem,R.F., Assiri,A., Al-Tawfiq,J.A., Albarrak,A., Barry,M., Shibl,A., Alrabiah,F.A., Hajjar,S., Balkhy,H.H., Flemban,H., Rambaut,A., Kellam,P., Memish,Z.A., Zumla,A. | 2014-02-06T00:00:00Z | ssRNA(+) | 253 | GenBank | S protein | Saudi Arabia | Homo sapiens | 01/09/2013 |
| AHI48803.1 | Cotten,M., Watson,S.J., Zumla,A.I., Makhdoom,H.Q., Palser,A.L., Ong,S.H., Al Rabeeah,A.A., Alhakeem,R.F., Assiri,A., Al-Tawfiq,J.A., Albarrak,A., Barry,M., Shibl,A., Alrabiah,F.A., Hajjar,S., Balkhy,H.H., Flemban,H., Rambaut,A., Kellam,P., Memish,Z.A., Zumla,A. | 2014-02-06T00:00:00Z | ssRNA(+) | 349 | GenBank | S protein | Saudi Arabia | Homo sapiens | 13/06/2013 |
| AHB33326.1 | Enouf,V., Briand,D., van der Werf,S. | 2013-12-07T00:00:00Z | ssRNA(+) | 1353 | GenBank | S protein | France | Homo sapiens | 07/05/2013 |
| AGV08379.1 | Cotten,M., Watson,S.J., Kellam,P., Al-Rabeeah,A.A., Makhdoom,H.Q., Assiri,A., Al-Tawfiq,J.A., Alhakeem,R.F., Madani,H., AlRabiah,F.A., Al Hajjar,S., Al-nassir,W.N., Albarrak,A., Flemban,H., Balkhy,H.H., Alsubaie,S., Palser,A.L., Gall,A., Bashford-Rogers,R., Rambaut,A., Zumla,A.I., Memish,Z.A., Ong,S.H., Al Rabeeah,A.A., Barry,M., Shibl,A., Alrabiah,F.A., Hajjar,S., Zumla,A. | 2013-09-17T00:00:00Z | ssRNA(+) | 1353 | GenBank | S protein | Saudi Arabia | Homo sapiens | 23/10/2012 |
| AGV08390.1 | Cotten,M., Watson,S.J., Kellam,P., Al-Rabeeah,A.A., Makhdoom,H.Q., Assiri,A., Al-Tawfiq,J.A., Alhakeem,R.F., Madani,H., AlRabiah,F.A., Al Hajjar,S., Al-nassir,W.N., Albarrak,A., Flemban,H., Balkhy,H.H., Alsubaie,S., Palser,A.L., Gall,A., Bashford-Rogers,R., Rambaut,A., Zumla,A.I., Memish,Z.A., Zumla,A. | 2013-09-17T00:00:00Z | ssRNA(+) | 1353 | GenBank | S protein | Saudi Arabia | Homo sapiens | 05/02/2013 |
| AGV08400.1 | Cotten,M., Watson,S.J., Kellam,P., Al-Rabeeah,A.A., Makhdoom,H.Q., Assiri,A., Al-Tawfiq,J.A., Alhakeem,R.F., Madani,H., AlRabiah,F.A., Al Hajjar,S., Al-nassir,W.N., Albarrak,A., Flemban,H., Balkhy,H.H., Alsubaie,S., Palser,A.L., Gall,A., Bashford-Rogers,R., Rambaut,A., Zumla,A.I., Memish,Z.A., Zumla,A. | 2013-09-17T00:00:00Z | ssRNA(+) | 313 | GenBank | S protein | Saudi Arabia | Homo sapiens | 02/05/2013 |
| AGV08408.1 | Cotten,M., Watson,S.J., Kellam,P., Al-Rabeeah,A.A., Makhdoom,H.Q., Assiri,A., Al-Tawfiq,J.A., Alhakeem,R.F., Madani,H., AlRabiah,F.A., Al Hajjar,S., Al-nassir,W.N., Albarrak,A., Flemban,H., Balkhy,H.H., Alsubaie,S., Palser,A.L., Gall,A., Bashford-Rogers,R., Rambaut,A., Zumla,A.I., Memish,Z.A., Zumla,A. | 2013-09-17T00:00:00Z | ssRNA(+) | 1353 | GenBank | S protein | Saudi Arabia | Homo sapiens | 19/06/2012 |
| AGV08420.1 | Cotten,M., Watson,S.J., Kellam,P., Al-Rabeeah,A.A., Makhdoom,H.Q., Assiri,A., Al-Tawfiq,J.A., Alhakeem,R.F., Madani,H., AlRabiah,F.A., Al Hajjar,S., Al-nassir,W.N., Albarrak,A., Flemban,H., Balkhy,H.H., Alsubaie,S., Palser,A.L., Gall,A., Bashford-Rogers,R., Rambaut,A., Zumla,A.I., Memish,Z.A., Zumla,A. | 2013-09-17T00:00:00Z | ssRNA(+) | 393 | GenBank | S protein | Saudi Arabia | Homo sapiens | 01/05/2013 |
| AGV08426.1 | Cotten,M., Watson,S.J., Kellam,P., Al-Rabeeah,A.A., Makhdoom,H.Q., Assiri,A., Al-Tawfiq,J.A., Alhakeem,R.F., Madani,H., AlRabiah,F.A., Al Hajjar,S., Al-nassir,W.N., Albarrak,A., Flemban,H., Balkhy,H.H., Alsubaie,S., Palser,A.L., Gall,A., Bashford-Rogers,R., Rambaut,A., Zumla,A.I., Memish,Z.A., Zumla,A. | 2013-09-17T00:00:00Z | ssRNA(+) | 1353 | GenBank | S protein | Saudi Arabia | Homo sapiens | 01/05/2013 |
| AGV08438.1 | Cotten,M., Watson,S.J., Kellam,P., Al-Rabeeah,A.A., Makhdoom,H.Q., Assiri,A., Al-Tawfiq,J.A., Alhakeem,R.F., Madani,H., AlRabiah,F.A., Al Hajjar,S., Al-nassir,W.N., Albarrak,A., Flemban,H., Balkhy,H.H., Alsubaie,S., Palser,A.L., Gall,A., Bashford-Rogers,R., Rambaut,A., Zumla,A.I., Memish,Z.A., Zumla,A. | 2013-09-17T00:00:00Z | ssRNA(+) | 1353 | GenBank | S protein | Saudi Arabia | Homo sapiens | 01/05/2013 |
| AGV08444.1 | Cotten,M., Watson,S.J., Kellam,P., Al-Rabeeah,A.A., Makhdoom,H.Q., Assiri,A., Al-Tawfiq,J.A., Alhakeem,R.F., Madani,H., AlRabiah,F.A., Al Hajjar,S., Al-nassir,W.N., Albarrak,A., Flemban,H., Balkhy,H.H., Alsubaie,S., Palser,A.L., Gall,A., Bashford-Rogers,R., Rambaut,A., Zumla,A.I., Memish,Z.A., Zumla,A. | 2013-09-17T00:00:00Z | ssRNA(+) | 1353 | GenBank | S protein | Saudi Arabia | Homo sapiens | 07/05/2013 |
| AGV08455.1 | Cotten,M., Watson,S.J., Kellam,P., Al-Rabeeah,A.A., Makhdoom,H.Q., Assiri,A., Al-Tawfiq,J.A., Alhakeem,R.F., Madani,H., AlRabiah,F.A., Al Hajjar,S., Al-nassir,W.N., Albarrak,A., Flemban,H., Balkhy,H.H., Alsubaie,S., Palser,A.L., Gall,A., Bashford-Rogers,R., Rambaut,A., Zumla,A.I., Memish,Z.A., Zumla,A. | 2013-09-17T00:00:00Z | ssRNA(+) | 1353 | GenBank | S protein | Saudi Arabia | Homo sapiens | 04/06/2013 |
| AGV08467.1 | Cotten,M., Watson,S.J., Kellam,P., Al-Rabeeah,A.A., Makhdoom,H.Q., Assiri,A., Al-Tawfiq,J.A., Alhakeem,R.F., Madani,H., AlRabiah,F.A., Al Hajjar,S., Al-nassir,W.N., Albarrak,A., Flemban,H., Balkhy,H.H., Alsubaie,S., Palser,A.L., Gall,A., Bashford-Rogers,R., Rambaut,A., Zumla,A.I., Memish,Z.A., Zumla,A. | 2013-09-17T00:00:00Z | ssRNA(+) | 1353 | GenBank | S protein | Saudi Arabia | Homo sapiens | 13/05/2013 |
| AGV08477.1 | Cotten,M., Watson,S.J., Kellam,P., Al-Rabeeah,A.A., Makhdoom,H.Q., Assiri,A., Al-Tawfiq,J.A., Alhakeem,R.F., Madani,H., AlRabiah,F.A., Al Hajjar,S., Al-nassir,W.N., Albarrak,A., Flemban,H., Balkhy,H.H., Alsubaie,S., Palser,A.L., Gall,A., Bashford-Rogers,R., Rambaut,A., Zumla,A.I., Memish,Z.A., Zumla,A. | 2013-09-17T00:00:00Z | ssRNA(+) | 983 | GenBank | S protein | Saudi Arabia | Homo sapiens | 09/05/2013 |
| AGV08480.1 | Cotten,M., Watson,S.J., Kellam,P., Al-Rabeeah,A.A., Makhdoom,H.Q., Assiri,A., Al-Tawfiq,J.A., Alhakeem,R.F., Madani,H., AlRabiah,F.A., Al Hajjar,S., Al-nassir,W.N., Albarrak,A., Flemban,H., Balkhy,H.H., Alsubaie,S., Palser,A.L., Gall,A., Bashford-Rogers,R., Rambaut,A., Zumla,A.I., Memish,Z.A., Zumla,A. | 2013-09-17T00:00:00Z | ssRNA(+) | 1353 | GenBank | S protein | Saudi Arabia | Homo sapiens | 23/05/2013 |
| AGV08492.1 | Cotten,M., Watson,S.J., Kellam,P., Al-Rabeeah,A.A., Makhdoom,H.Q., Assiri,A., Al-Tawfiq,J.A., Alhakeem,R.F., Madani,H., AlRabiah,F.A., Al Hajjar,S., Al-nassir,W.N., Albarrak,A., Flemban,H., Balkhy,H.H., Alsubaie,S., Palser,A.L., Gall,A., Bashford-Rogers,R., Rambaut,A., Zumla,A.I., Memish,Z.A., Zumla,A. | 2013-09-17T00:00:00Z | ssRNA(+) | 1353 | GenBank | S protein | Saudi Arabia | Homo sapiens | 30/05/2013 |
| AGV08505.1 | Cotten,M., Watson,S.J., Kellam,P., Al-Rabeeah,A.A., Makhdoom,H.Q., Assiri,A., Al-Tawfiq,J.A., Alhakeem,R.F., Madani,H., AlRabiah,F.A., Al Hajjar,S., Al-nassir,W.N., Albarrak,A., Flemban,H., Balkhy,H.H., Alsubaie,S., Palser,A.L., Gall,A., Bashford-Rogers,R., Rambaut,A., Zumla,A.I., Memish,Z.A., Zumla,A. | 2013-09-17T00:00:00Z | ssRNA(+) | 1353 | GenBank | S protein | Saudi Arabia | Homo sapiens | 03/05/2013 |
| AGV08520.1 | Cotten,M., Watson,S.J., Kellam,P., Al-Rabeeah,A.A., Makhdoom,H.Q., Assiri,A., Al-Tawfiq,J.A., Alhakeem,R.F., Madani,H., AlRabiah,F.A., Al Hajjar,S., Al-nassir,W.N., Albarrak,A., Flemban,H., Balkhy,H.H., Alsubaie,S., Palser,A.L., Gall,A., Bashford-Rogers,R., Rambaut,A., Zumla,A.I., Memish,Z.A., Zumla,A. | 2013-09-17T00:00:00Z | ssRNA(+) | 238 | GenBank | S protein | Saudi Arabia | Homo sapiens | 07/05/2013 |
| AGV08524.1 | Cotten,M., Watson,S.J., Kellam,P., Al-Rabeeah,A.A., Makhdoom,H.Q., Assiri,A., Al-Tawfiq,J.A., Alhakeem,R.F., Madani,H., AlRabiah,F.A., Al Hajjar,S., Al-nassir,W.N., Albarrak,A., Flemban,H., Balkhy,H.H., Alsubaie,S., Palser,A.L., Gall,A., Bashford-Rogers,R., Rambaut,A., Zumla,A.I., Memish,Z.A., Zumla,A. | 2013-09-17T00:00:00Z | ssRNA(+) | 1353 | GenBank | S protein | Saudi Arabia | Homo sapiens | 08/05/2013 |
| AGV08535.1 | Cotten,M., Watson,S.J., Kellam,P., Al-Rabeeah,A.A., Makhdoom,H.Q., Assiri,A., Al-Tawfiq,J.A., Alhakeem,R.F., Madani,H., AlRabiah,F.A., Al Hajjar,S., Al-nassir,W.N., Albarrak,A., Flemban,H., Balkhy,H.H., Alsubaie,S., Palser,A.L., Gall,A., Bashford-Rogers,R., Rambaut,A., Zumla,A.I., Memish,Z.A., Zumla,A. | 2013-09-17T00:00:00Z | ssRNA(+) | 1353 | GenBank | S protein | Saudi Arabia | Homo sapiens | 12/05/2013 |
| AGV08546.1 | Cotten,M., Watson,S.J., Kellam,P., Al-Rabeeah,A.A., Makhdoom,H.Q., Assiri,A., Al-Tawfiq,J.A., Alhakeem,R.F., Madani,H., AlRabiah,F.A., Al Hajjar,S., Al-nassir,W.N., Albarrak,A., Flemban,H., Balkhy,H.H., Alsubaie,S., Palser,A.L., Gall,A., Bashford-Rogers,R., Rambaut,A., Zumla,A.I., Memish,Z.A., Zumla,A. | 2013-09-17T00:00:00Z | ssRNA(+) | 1353 | GenBank | S protein | Saudi Arabia | Homo sapiens | 11/05/2013 |
| AGV08558.1 | Cotten,M., Watson,S.J., Kellam,P., Al-Rabeeah,A.A., Makhdoom,H.Q., Assiri,A., Al-Tawfiq,J.A., Alhakeem,R.F., Madani,H., AlRabiah,F.A., Al Hajjar,S., Al-nassir,W.N., Albarrak,A., Flemban,H., Balkhy,H.H., Alsubaie,S., Palser,A.L., Gall,A., Bashford-Rogers,R., Rambaut,A., Zumla,A.I., Memish,Z.A., Zumla,A. | 2013-09-17T00:00:00Z | ssRNA(+) | 1353 | GenBank | S protein | Saudi Arabia | Homo sapiens | 15/05/2013 |
| AGV08569.1 | Cotten,M., Watson,S.J., Kellam,P., Al-Rabeeah,A.A., Makhdoom,H.Q., Assiri,A., Al-Tawfiq,J.A., Alhakeem,R.F., Madani,H., AlRabiah,F.A., Al Hajjar,S., Al-nassir,W.N., Albarrak,A., Flemban,H., Balkhy,H.H., Alsubaie,S., Palser,A.L., Gall,A., Bashford-Rogers,R., Rambaut,A., Zumla,A.I., Memish,Z.A., Zumla,A. | 2013-09-17T00:00:00Z | ssRNA(+) | 313 | GenBank | S protein | Saudi Arabia | Homo sapiens | 01/05/2013 |
| AGV08573.1 | Cotten,M., Watson,S.J., Kellam,P., Al-Rabeeah,A.A., Makhdoom,H.Q., Assiri,A., Al-Tawfiq,J.A., Alhakeem,R.F., Madani,H., AlRabiah,F.A., Al Hajjar,S., Al-nassir,W.N., Albarrak,A., Flemban,H., Balkhy,H.H., Alsubaie,S., Palser,A.L., Gall,A., Bashford-Rogers,R., Rambaut,A., Zumla,A.I., Memish,Z.A., Zumla,A. | 2013-09-17T00:00:00Z | ssRNA(+) | 1353 | GenBank | S protein | Saudi Arabia | Homo sapiens | 23/05/2013 |
| AGV08584.1 | Cotten,M., Watson,S.J., Kellam,P., Al-Rabeeah,A.A., Makhdoom,H.Q., Assiri,A., Al-Tawfiq,J.A., Alhakeem,R.F., Madani,H., AlRabiah,F.A., Al Hajjar,S., Al-nassir,W.N., Albarrak,A., Flemban,H., Balkhy,H.H., Alsubaie,S., Palser,A.L., Gall,A., Bashford-Rogers,R., Rambaut,A., Zumla,A.I., Memish,Z.A., Zumla,A. | 2013-09-17T00:00:00Z | ssRNA(+) | 1353 | GenBank | S protein | Saudi Arabia | Homo sapiens | 30/10/2012 |
| AGN70929.1 | Cotten,M., Watson,S.J., Kellam,P., Al-Rabeeah,A.A., Makhdoom,H.Q., Assiri,A., Al-Tawfiq,J.A., Alhakeem,R.F., Madani,H., AlRabiah,F.A., Al Hajjar,S., Al-nassir,W.N., Albarrak,A., Flemban,H., Balkhy,H.H., Alsubaie,S., Palser,A.L., Gall,A., Bashford-Rogers,R., Rambaut,A., Zumla,A.I., Memish,Z.A., Zumla,A. | 2013-06-10T00:00:00Z | ssRNA(+) | 1353 | GenBank | S protein | Saudi Arabia | Homo sapiens | 01/05/2013 |
| AGN70951.1 | Cotten,M., Watson,S.J., Kellam,P., Al-Rabeeah,A.A., Makhdoom,H.Q., Assiri,A., Al-Tawfiq,J.A., Alhakeem,R.F., Madani,H., AlRabiah,F.A., Al Hajjar,S., Al-nassir,W.N., Albarrak,A., Flemban,H., Balkhy,H.H., Alsubaie,S., Palser,A.L., Gall,A., Bashford-Rogers,R., Rambaut,A., Zumla,A.I., Memish,Z.A., Zumla,A. | 2013-06-10T00:00:00Z | ssRNA(+) | 1353 | GenBank | S protein | Saudi Arabia | Homo sapiens | 21/04/2013 |
| AGN70962.1 | Cotten,M., Watson,S.J., Kellam,P., Al-Rabeeah,A.A., Makhdoom,H.Q., Assiri,A., Al-Tawfiq,J.A., Alhakeem,R.F., Madani,H., AlRabiah,F.A., Al Hajjar,S., Al-nassir,W.N., Albarrak,A., Flemban,H., Balkhy,H.H., Alsubaie,S., Palser,A.L., Gall,A., Bashford-Rogers,R., Rambaut,A., Zumla,A.I., Memish,Z.A., Zumla,A. | 2013-06-10T00:00:00Z | ssRNA(+) | 1353 | GenBank | S protein | Saudi Arabia | Homo sapiens | 09/05/2013 |
| AGN70973.1 | Cotten,M., Watson,S.J., Kellam,P., Al-Rabeeah,A.A., Makhdoom,H.Q., Assiri,A., Al-Tawfiq,J.A., Alhakeem,R.F., Madani,H., AlRabiah,F.A., Al Hajjar,S., Al-nassir,W.N., Albarrak,A., Flemban,H., Balkhy,H.H., Alsubaie,S., Palser,A.L., Gall,A., Bashford-Rogers,R., Rambaut,A., Zumla,A.I., Memish,Z.A., Zumla,A. | 2013-06-10T00:00:00Z | ssRNA(+) | 1353 | GenBank | S protein | Saudi Arabia | Homo sapiens | 22/04/2013 |
| AGH58717.1 | Elassal,E.M., Demian,P.N., Defang,G.N., Rozanski,C.H., Curry,J.A., Oyofo,B.A., Al-Sanouri,T.M., Haddadin,A., Mohareb,E.W. | 2013-03-25T00:00:00Z | ssRNA(+) | 1353 | GenBank | S protein | Jordan | Homo sapiens | 2012-04 |
| AGG22542.1 | Cotten,M., Lam,T.T., Watson,S.J., Palser,A.L., Petrova,V., Grant,P., Pybus,O.G., Rambaut,A., Guan,Y., Pillay,D., Kellam,P., Nastouli,E., Cotten,M.L., Palser,A., Pybus,O. | 2013-02-27T00:00:00Z | ssRNA(+) | 1353 | GenBank | S protein | United Kingdom | Homo sapiens | 19/09/2012 |
| AFY13307.1 | Galiano,M., Myers,R., Bermingham,A., Gopal,R., Zambon,M. | 2012-12-05T00:00:00Z | ssRNA(+) | 1353 | GenBank | S protein | United Kingdom | Homo sapiens | 11/09/2012 |
| AFS88936.1 | van Boheemen,S., de Graaf,M., Lauber,C., Bestebroer,T.M., Raj,V.S., Zaki,A.M., Osterhaus,A.D., Haagmans,B.L., Gorbalenya,A.E., Snijder,E.J., Fouchier,R.A., Victor,S.R., Osterhaus,A.D.M.E., Fouchier,R.A.M. | 2012-09-27T00:00:00Z | ssRNA(+) | 1353 | GenBank | S protein |  | Homo sapiens | 13/06/2012 |
| QJX19957.1 | Kim,S., Yi,H. | 2020-05-25T00:00:00Z | ssRNA(+) | 1353 | GenBank | Spike protein | South Korea | Homo sapiens | 20/05/2015 |
| AGO06002.1 | Drosten,C., Seilmaier,M., Corman,V.M., Hartmann,W., Scheible,G., Sack,S., Guggemos,W., Kallies,R., Muth,D., Junglen,S., Muller,M.A., Haas,W., Guberina,H., Rohnisch,T., Schmid-Wendtner,M., Aldabbagh,S., Dittmer,U., Gold,H., Graf,P., Bonin,F., Rambaut,A., Wendtner,C.M. | 2013-06-23T00:00:00Z | ssRNA(+) | 45 | GenBank | spike | Qatar | Homo sapiens | 2012 |
| QGW51898.1 | Queen,K., Tamin,A., Assiri,A.M., Paden,C.R., Thornburg,N.J., Tong,S. | 2019-12-18T00:00:00Z | ssRNA(+) | 1353 | GenBank | spike glycoprotein | Saudi Arabia | Homo sapiens | 01/08/2017 |
| QGW51909.1 | Queen,K., Tamin,A., Assiri,A.M., Paden,C.R., Thornburg,N.J., Tong,S. | 2019-12-18T00:00:00Z | ssRNA(+) | 1353 | GenBank | spike glycoprotein | Saudi Arabia | Homo sapiens | 17/08/2017 |
| QGW51920.1 | Queen,K., Tamin,A., Assiri,A.M., Paden,C.R., Thornburg,N.J., Tong,S. | 2019-12-18T00:00:00Z | ssRNA(+) | 1353 | GenBank | spike glycoprotein | Saudi Arabia | Homo sapiens | 30/08/2018 |
| QGW51938.1 | Naeem,A., Alosaimi,B., Enani,M., Alsaran,H. | 2019-12-18T00:00:00Z | ssRNA(+) | 1353 | GenBank | spike glycoprotein | Saudi Arabia | Homo sapiens | 28/09/2015 |
| QGW51939.1 | Naeem,A., Alosaimi,B., Enani,M., Alsaran,H. | 2019-12-18T00:00:00Z | ssRNA(+) | 1353 | GenBank | spike glycoprotein | Saudi Arabia | Homo sapiens | 03/05/2016 |
| QGW51940.1 | Naeem,A., Alosaimi,B., Enani,M., Alsaran,H. | 2019-12-18T00:00:00Z | ssRNA(+) | 1353 | GenBank | spike glycoprotein | Saudi Arabia | Homo sapiens | 23/06/2017 |
| QGW51941.1 | Naeem,A., Alosaimi,B., Enani,M., Alsaran,H. | 2019-12-18T00:00:00Z | ssRNA(+) | 1353 | GenBank | spike glycoprotein | Saudi Arabia | Homo sapiens | 16/11/2017 |
| QDP16195.1 | AlBalwi,M., Anis,K., UdayaRaja G,K., Al Deeris,M., AlAbdulkareem,I. | 2019-07-27T00:00:00Z | ssRNA(+) | 1353 | GenBank | spike glycoprotein | Saudi Arabia | Homo sapiens | 27/03/2019 |
| QDP16206.1 | Al Balwi,M., Khan,A., UdayaRaja G,K., Al Drees,M., AlAbdulkareem,M. | 2019-07-27T00:00:00Z | ssRNA(+) | 1353 | GenBank | spike glycoprotein | Saudi Arabia | Homo sapiens | 28/03/2019 |
| QCQ28828.1 | Alosaimi,B., Naeem,A., Assiri,A.M., Enani,M. | 2019-05-20T00:00:00Z | ssRNA(+) | 1353 | GenBank | spike glycoprotein | Saudi Arabia | Homo sapiens | 17/10/2016 |
| QCQ28829.1 | Alosaimi,B., Naeem,A., Assiri,A.M., Enani,M. | 2019-05-20T00:00:00Z | ssRNA(+) | 1353 | GenBank | spike glycoprotein | Saudi Arabia | Homo sapiens | 17/11/2016 |
| QCQ28830.1 | Alosaimi,B., Naeem,A., Assiri,A.M., Enani,M. | 2019-05-20T00:00:00Z | ssRNA(+) | 1353 | GenBank | spike glycoprotein | Saudi Arabia | Homo sapiens | 28/11/2016 |
| QCQ28831.1 | Alosaimi,B., Naeem,A., Assiri,A.M., Enani,M. | 2019-05-20T00:00:00Z | ssRNA(+) | 1353 | GenBank | spike glycoprotein | Saudi Arabia | Homo sapiens | 19/12/2016 |
| QCQ28832.1 | Alosaimi,B., Naeem,A., Assiri,A.M., Enani,M. | 2019-05-20T00:00:00Z | ssRNA(+) | 1353 | GenBank | spike glycoprotein | Saudi Arabia | Homo sapiens | 31/05/2017 |
| QCQ28833.1 | Alosaimi,B., Naeem,A., Assiri,A.M., Enani,M. | 2019-05-20T00:00:00Z | ssRNA(+) | 1353 | GenBank | spike glycoprotein | Saudi Arabia | Homo sapiens | 03/06/2017 |
| QCQ28834.1 | Alosaimi,B., Naeem,A., Assiri,A.M., Enani,M. | 2019-05-20T00:00:00Z | ssRNA(+) | 1353 | GenBank | spike glycoprotein | Saudi Arabia | Homo sapiens | 03/06/2017 |
| QCQ28835.1 | Alosaimi,B., Naeem,A., Assiri,A.M., Enani,M. | 2019-05-20T00:00:00Z | ssRNA(+) | 1353 | GenBank | spike glycoprotein | Saudi Arabia | Homo sapiens | 03/06/2017 |
| QCQ28836.1 | Alosaimi,B., Naeem,A., Assiri,A.M., Enani,M. | 2019-05-20T00:00:00Z | ssRNA(+) | 1353 | GenBank | spike glycoprotein | Saudi Arabia | Homo sapiens | 05/06/2017 |
| AYO86699.1 | Li,Y., Al-Abdely,H.M., Tamin,A., Queen,K., Thornburg,N., Tong,S. | 2019-03-06T00:00:00Z | ssRNA(+) | 1353 | GenBank | spike glycoprotein | Saudi Arabia | Homo sapiens | 26/08/2015 |
| AYV64549.1 | Paden,C.R., Tamin,A., AL-Abdallat,M.M., Haddadin,A., Al-sanouri,T., Queen,K., Li,Y., Payne,D.C., Tong,S. | 2018-11-19T00:00:00Z | ssRNA(+) | 1353 | GenBank | spike glycoprotein | Jordan | Homo sapiens | 21/04/2014 |
| AYV64560.1 | Paden,C.R., Tamin,A., AL-Abdallat,M.M., Haddadin,A., Al-sanouri,T., Queen,K., Li,Y., Payne,D.C., Tong,S. | 2018-11-19T00:00:00Z | ssRNA(+) | 1353 | GenBank | spike glycoprotein | Jordan | Homo sapiens | 23/05/2014 |
| AYP10298.1 | Queen,K., Paden,C.R., Al-Abdallat,M.M., Haddadin,A., Al-Sanouri,T., Li,Y., Lu,X., Erdman,D.D., Payne,D.C., Tong,S. | 2018-11-05T00:00:00Z | ssRNA(+) | 1353 | GenBank | spike glycoprotein | Jordan | Homo sapiens | 23/05/2014 |
| AYJ71470.1 | Al-Abdely,H.M., Midgley,C.M., Alkhamis,A.M., Abedi,G.R., Tamin,A., Binder,A.M., Alanazi,K., Lu,X., Abdalla,O., Sakthivel,S.K., Mohammed,M., Queen,K., Algarni,H.S., Li,Y., Trivedi,S., Algwizani,A., Alhakeem,R.F., Thornburg,N.J., Tong,S., Ghazal,S.S., Erdman,D.D., Assiri,A.M., Gerber,S.I., Watson,J.T., Thornburg,N. | 2018-10-20T00:00:00Z | ssRNA(+) | 1353 | GenBank | spike glycoprotein | Saudi Arabia | Homo sapiens | 13/10/2015 |
| AYJ71481.1 | Al-Abdely,H.M., Midgley,C.M., Alkhamis,A.M., Abedi,G.R., Tamin,A., Binder,A.M., Alanazi,K., Lu,X., Abdalla,O., Sakthivel,S.K., Mohammed,M., Queen,K., Algarni,H.S., Li,Y., Trivedi,S., Algwizani,A., Alhakeem,R.F., Thornburg,N.J., Tong,S., Ghazal,S.S., Erdman,D.D., Assiri,A.M., Gerber,S.I., Watson,J.T., Thornburg,N. | 2018-10-20T00:00:00Z | ssRNA(+) | 1353 | GenBank | spike glycoprotein | Saudi Arabia | Homo sapiens | 15/10/2015 |
| AYM48030.1 | Galiano,M., Myers,R., Ellis,J., Gopal,R., Zambon,M. | 2018-10-19T00:00:00Z | ssRNA(+) | 1353 | GenBank | spike glycoprotein | United Kingdom | Homo sapiens | 22/08/2018 |
| ARQ84735.1 | Paden,C.R., Tamin,A., AL-Abdallat,M.M., Haddadin,A., Al-sanouri,T., Queen,K., Li,Y., Zhang,J., Payne,D.C., Tong,S. | 2018-10-15T00:00:00Z | ssRNA(+) | 1353 | GenBank | spike glycoprotein | Jordan | Homo sapiens | 25/08/2015 |
| ARQ84746.1 | Paden,C.R., Tamin,A., AL-Abdallat,M.M., Haddadin,A., Al-sanouri,T., Queen,K., Li,Y., Zhang,J., Payne,D.C., Tong,S. | 2018-10-15T00:00:00Z | ssRNA(+) | 1353 | GenBank | spike glycoprotein | Jordan | Homo sapiens | 19/09/2015 |
| ARQ84757.1 | Paden,C.R., Tamin,A., AL-Abdallat,M.M., Haddadin,A., Al-sanouri,T., Queen,K., Li,Y., Zhang,J., Payne,D.C., Tong,S. | 2018-10-15T00:00:00Z | ssRNA(+) | 1353 | GenBank | spike glycoprotein | Jordan | Homo sapiens | 07/09/2015 |
| ARQ84768.1 | Paden,C.R., Tamin,A., AL-Abdallat,M.M., Haddadin,A., Al-sanouri,T., Queen,K., Li,Y., Zhang,J., Payne,D.C., Tong,S. | 2018-10-15T00:00:00Z | ssRNA(+) | 1353 | GenBank | spike glycoprotein | Jordan | Homo sapiens | 30/08/2015 |
| AVK87318.1 | Queen,K., Li,Y., Tao,Y., Zhang,J., Paden,C.R., Assiri,A.M., Tong,S. | 2018-08-31T00:00:00Z | ssRNA(+) | 1353 | GenBank | spike glycoprotein | Saudi Arabia | Homo sapiens | 31/05/2017 |
| AVK87329.1 | Queen,K., Li,Y., Tao,Y., Zhang,J., Paden,C.R., Assiri,A.M., Tong,S. | 2018-08-31T00:00:00Z | ssRNA(+) | 1353 | GenBank | spike glycoprotein | Saudi Arabia | Homo sapiens | 02/06/2017 |
| AVK87340.1 | Queen,K., Li,Y., Tao,Y., Zhang,J., Paden,C.R., Assiri,A.M., Tong,S. | 2018-08-31T00:00:00Z | ssRNA(+) | 1353 | GenBank | spike glycoprotein | Saudi Arabia | Homo sapiens | 02/06/2017 |
| AVK87351.1 | Queen,K., Li,Y., Tao,Y., Zhang,J., Paden,C.R., Assiri,A.M., Tong,S. | 2018-08-31T00:00:00Z | ssRNA(+) | 1353 | GenBank | spike glycoprotein | Saudi Arabia | Homo sapiens | 03/06/2017 |
| AVK87362.1 | Queen,K., Li,Y., Tao,Y., Zhang,J., Paden,C.R., Assiri,A.M., Tong,S. | 2018-08-31T00:00:00Z | ssRNA(+) | 1353 | GenBank | spike glycoprotein | Saudi Arabia | Homo sapiens | 07/06/2017 |
| AVK87373.1 | Queen,K., Li,Y., Tao,Y., Zhang,J., Paden,C.R., Assiri,A.M., Tong,S. | 2018-08-31T00:00:00Z | ssRNA(+) | 1353 | GenBank | spike glycoprotein | Saudi Arabia | Homo sapiens | 08/06/2017 |
| AVK87384.1 | Queen,K., Li,Y., Tao,Y., Zhang,J., Paden,C.R., Assiri,A.M., Tong,S. | 2018-08-31T00:00:00Z | ssRNA(+) | 1353 | GenBank | spike glycoprotein | Saudi Arabia | Homo sapiens | 10/06/2017 |
| AVK87395.1 | Queen,K., Li,Y., Tao,Y., Zhang,J., Paden,C.R., Assiri,A.M., Tong,S. | 2018-08-31T00:00:00Z | ssRNA(+) | 1353 | GenBank | spike glycoprotein | Saudi Arabia | Homo sapiens | 12/06/2017 |
| AVK87406.1 | Queen,K., Li,Y., Tao,Y., Zhang,J., Paden,C.R., Assiri,A.M., Tong,S. | 2018-08-31T00:00:00Z | ssRNA(+) | 1353 | GenBank | spike glycoprotein | Saudi Arabia | Homo sapiens | 12/06/2017 |
| AVK87417.1 | Queen,K., Li,Y., Tao,Y., Zhang,J., Paden,C.R., Assiri,A.M., Tong,S. | 2018-08-31T00:00:00Z | ssRNA(+) | 1353 | GenBank | spike glycoprotein | Saudi Arabia | Homo sapiens | 15/06/2017 |
| AVK87428.1 | Queen,K., Li,Y., Tao,Y., Zhang,J., Paden,C.R., Assiri,A.M., Tong,S. | 2018-08-31T00:00:00Z | ssRNA(+) | 1353 | GenBank | spike glycoprotein | Saudi Arabia | Homo sapiens | 17/06/2017 |
| AVK87439.1 | Queen,K., Li,Y., Tao,Y., Zhang,J., Paden,C.R., Assiri,A.M., Tong,S. | 2018-08-31T00:00:00Z | ssRNA(+) | 1353 | GenBank | spike glycoprotein | Saudi Arabia | Homo sapiens | 14/06/2017 |
| AVK87450.1 | Queen,K., Li,Y., Tao,Y., Zhang,J., Paden,C.R., Assiri,A.M., Tong,S. | 2018-08-31T00:00:00Z | ssRNA(+) | 1353 | GenBank | spike glycoprotein | Saudi Arabia | Homo sapiens | 11/06/2017 |
| AVK87461.1 | Queen,K., Li,Y., Tao,Y., Zhang,J., Paden,C.R., Assiri,A.M., Tong,S. | 2018-08-31T00:00:00Z | ssRNA(+) | 1353 | GenBank | spike glycoprotein | Saudi Arabia | Homo sapiens | 14/06/2017 |
| AVK87471.1 | Queen,K., Li,Y., Tao,Y., Zhang,J., Paden,C.R., Assiri,A.M., Tong,S. | 2018-08-31T00:00:00Z | ssRNA(+) | 1353 | GenBank | spike glycoprotein | Saudi Arabia | Homo sapiens | 31/05/2017 |
| AXG21654.1 | AlBalwi,M.A., Khan,A., UdayaRaja,K., AlAbdulkareem,I., AlMasoud,A., AlAsiri,A., Balavenkatesh Mani,M., AlHarbi,W., El-Saed,A., Balkhy,H. | 2018-07-31T00:00:00Z | ssRNA(+) | 1353 | GenBank | spike glycoprotein | Saudi Arabia | Homo sapiens | 04/12/2016 |
| AXG21665.1 | Al Balwi,M., UdayaRaja G,K., Khan,A., AlAbdulkareem,I., AlMasoud,A., AlAsiri,A., Balavenkatesh Mani,M., AlHarbi,W., El-Saed,A., Balkhy,H. | 2018-07-31T00:00:00Z | ssRNA(+) | 1353 | GenBank | spike glycoprotein | Saudi Arabia | Homo sapiens | 28/02/2017 |
| AWU59321.1 | Al Balwi,M., Khan,A., UdayaRaja,G.K., AlAbdulkareem,I., AlMasoud,A., AlAsiri,A., Balavenkatesh Mani,M., AlHarbi,W., El-Saed,A., H Balkhy,H., Siddiqui,Z. | 2018-06-19T00:00:00Z | ssRNA(+) | 1353 | GenBank | spike glycoprotein | Saudi Arabia | Homo sapiens | 06/12/2016 |
| AWM99582.1 | Al Balwi,M.A., Khan,A., Al Abdulkareem,I., AlMasoud,A., Al Asiri,A., Al Harbi,W., El Saed,A., UdayaRaja,G.K., Balavenkatesh Mani,M., Balkhy,H.H. | 2018-05-30T00:00:00Z | ssRNA(+) | 1353 | GenBank | spike glycoprotein | Saudi Arabia | Homo sapiens | 01/08/2016 |
| AVV61900.1 | Al Balwi,M., UdayaRaja G,K. | 2018-04-10T00:00:00Z | ssRNA(+) | 1353 | GenBank | spike glycoprotein | Saudi Arabia | Homo sapiens | 15/10/2015 |
| AQZ41285.1 | Queen,K., Al-Jardani,A., Zhang,J., Al Kindi,H., Li,Y., Tao,Y., Al Baqlani,S., Al Mahrouqi,S., Tong,S. | 2017-12-31T00:00:00Z | ssRNA(+) | 1353 | GenBank | spike glycoprotein | Oman | Homo sapiens | 06/01/2015 |
| AQZ41306.1 | Queen,K., Al-Jardani,A., Zhang,J., Al Kindi,H., Li,Y., Tao,Y., Al Baqlani,S., Al Mahrouqi,S., Tong,S. | 2017-12-31T00:00:00Z | ssRNA(+) | 1353 | GenBank | spike glycoprotein | Oman | Homo sapiens | 16/01/2015 |
| AQZ41311.1 | Paden,C.R., Tamin,A., Assiri,A.M., Queen,K., Li,Y., Zhang,J., Tong,S. | 2017-12-31T00:00:00Z | ssRNA(+) | 1353 | GenBank | spike glycoprotein | Saudi Arabia | Homo sapiens | 07/02/2015 |
| AQZ41322.1 | Paden,C.R., Tamin,A., Assiri,A.M., Queen,K., Li,Y., Zhang,J., Tong,S. | 2017-12-31T00:00:00Z | ssRNA(+) | 1353 | GenBank | spike glycoprotein | Saudi Arabia | Homo sapiens | 01/05/2015 |
| AQZ41332.1 | Paden,C.R., Tamin,A., Assiri,A.M., Queen,K., Li,Y., Zhang,J., Tong,S. | 2017-12-31T00:00:00Z | ssRNA(+) | 1353 | GenBank | spike glycoprotein | Saudi Arabia | Homo sapiens | 10/05/2015 |
| AQZ41343.1 | Paden,C.R., Tamin,A., Assiri,A.M., Queen,K., Li,Y., Zhang,J., Tong,S. | 2017-12-31T00:00:00Z | ssRNA(+) | 1353 | GenBank | spike glycoprotein | Saudi Arabia | Homo sapiens | 22/05/2015 |
| AQZ41354.1 | Paden,C.R., Tamin,A., Assiri,A.M., Queen,K., Li,Y., Zhang,J., Tong,S. | 2017-12-31T00:00:00Z | ssRNA(+) | 1353 | GenBank | spike glycoprotein | Saudi Arabia | Homo sapiens | 27/05/2015 |
| AQZ41365.1 | Paden,C.R., Tamin,A., Assiri,A.M., Queen,K., Li,Y., Zhang,J., Tong,S. | 2017-12-31T00:00:00Z | ssRNA(+) | 1353 | GenBank | spike glycoprotein | Saudi Arabia | Homo sapiens | 13/05/2015 |
| AQZ41376.1 | Paden,C.R., Tamin,A., Assiri,A.M., Queen,K., Li,Y., Zhang,J., Tong,S. | 2017-12-31T00:00:00Z | ssRNA(+) | 1353 | GenBank | spike glycoprotein | Saudi Arabia | Homo sapiens | 20/05/2015 |
| ASU45696.1 | Paden,C.R., Yusof,M.F.B.M., Al Hammadi,Z.M., Queen,K., Tao,Y., Eltahir,Y.M., Elsayed,E.A., Marzoug,B.A., Bensalah,O.K.A., Khalafalla,A.I., Al Mulla,M., Elkheir,K.A., Issa,Z.B., Pradeep,K., Elsaleh,F.N., Imambaccus,H., Sasse,J., Weber,S., Shi,M., Zhang,J., Li,Y., Pham,H., Kim,L., Hall,A.J., Gerber,S.I., Al Hosani,F.I., Tong,S., Al Muhairi,S.S.M., Al Muhairi,S.S., Al Hammadi,Z., Abou Elkheir,K., Al Bandar,Z., El Saleh,F., Hall,A., Gerber,S. | 2017-12-20T00:00:00Z | ssRNA(+) | 1353 | GenBank | spike glycoprotein | United Arab Emirates | Homo sapiens | 10/07/2013 |
| ASU45708.1 | Paden,C.R., Yusof,M.F.B.M., Al Hammadi,Z.M., Queen,K., Tao,Y., Eltahir,Y.M., Elsayed,E.A., Marzoug,B.A., Bensalah,O.K.A., Khalafalla,A.I., Al Mulla,M., Elkheir,K.A., Issa,Z.B., Pradeep,K., Elsaleh,F.N., Imambaccus,H., Sasse,J., Weber,S., Shi,M., Zhang,J., Li,Y., Pham,H., Kim,L., Hall,A.J., Gerber,S.I., Al Hosani,F.I., Tong,S., Al Muhairi,S.S.M., Al Muhairi,S.S., Al Hammadi,Z., Abou Elkheir,K., Al Bandar,Z., El Saleh,F., Hall,A., Gerber,S. | 2017-12-20T00:00:00Z | ssRNA(+) | 1353 | GenBank | spike glycoprotein | United Arab Emirates | Homo sapiens | 12/07/2013 |
| ASU45719.1 | Paden,C.R., Yusof,M.F.B.M., Al Hammadi,Z.M., Queen,K., Tao,Y., Eltahir,Y.M., Elsayed,E.A., Marzoug,B.A., Bensalah,O.K.A., Khalafalla,A.I., Al Mulla,M., Elkheir,K.A., Issa,Z.B., Pradeep,K., Elsaleh,F.N., Imambaccus,H., Sasse,J., Weber,S., Shi,M., Zhang,J., Li,Y., Pham,H., Kim,L., Hall,A.J., Gerber,S.I., Al Hosani,F.I., Tong,S., Al Muhairi,S.S.M., Al Muhairi,S.S., Al Hammadi,Z., Abou Elkheir,K., Al Bandar,Z., El Saleh,F., Hall,A., Gerber,S. | 2017-12-20T00:00:00Z | ssRNA(+) | 1353 | GenBank | spike glycoprotein | United Arab Emirates | Homo sapiens | 10/04/2014 |
| ASU45730.1 | Paden,C.R., Yusof,M.F.B.M., Al Hammadi,Z.M., Queen,K., Tao,Y., Eltahir,Y.M., Elsayed,E.A., Marzoug,B.A., Bensalah,O.K.A., Khalafalla,A.I., Al Mulla,M., Elkheir,K.A., Issa,Z.B., Pradeep,K., Elsaleh,F.N., Imambaccus,H., Sasse,J., Weber,S., Shi,M., Zhang,J., Li,Y., Pham,H., Kim,L., Hall,A.J., Gerber,S.I., Al Hosani,F.I., Tong,S., Al Muhairi,S.S.M., Al Muhairi,S.S., Al Hammadi,Z., Abou Elkheir,K., Al Bandar,Z., El Saleh,F., Hall,A., Gerber,S. | 2017-12-20T00:00:00Z | ssRNA(+) | 1353 | GenBank | spike glycoprotein | United Arab Emirates | Homo sapiens | 23/12/2013 |
| ASU45741.1 | Paden,C.R., Yusof,M.F.B.M., Al Hammadi,Z.M., Queen,K., Tao,Y., Eltahir,Y.M., Elsayed,E.A., Marzoug,B.A., Bensalah,O.K.A., Khalafalla,A.I., Al Mulla,M., Elkheir,K.A., Issa,Z.B., Pradeep,K., Elsaleh,F.N., Imambaccus,H., Sasse,J., Weber,S., Shi,M., Zhang,J., Li,Y., Pham,H., Kim,L., Hall,A.J., Gerber,S.I., Al Hosani,F.I., Tong,S., Al Muhairi,S.S.M., Al Muhairi,S.S., Al Hammadi,Z., Abou Elkheir,K., Al Bandar,Z., El Saleh,F., Hall,A., Gerber,S. | 2017-12-20T00:00:00Z | ssRNA(+) | 1353 | GenBank | spike glycoprotein | United Arab Emirates | Homo sapiens | 10/04/2014 |
| ASU45752.1 | Paden,C.R., Yusof,M.F.B.M., Al Hammadi,Z.M., Queen,K., Tao,Y., Eltahir,Y.M., Elsayed,E.A., Marzoug,B.A., Bensalah,O.K.A., Khalafalla,A.I., Al Mulla,M., Elkheir,K.A., Issa,Z.B., Pradeep,K., Elsaleh,F.N., Imambaccus,H., Sasse,J., Weber,S., Shi,M., Zhang,J., Li,Y., Pham,H., Kim,L., Hall,A.J., Gerber,S.I., Al Hosani,F.I., Tong,S., Al Muhairi,S.S.M., Al Muhairi,S.S., Al Hammadi,Z., Abou Elkheir,K., Al Bandar,Z., El Saleh,F., Hall,A., Gerber,S. | 2017-12-20T00:00:00Z | ssRNA(+) | 1353 | GenBank | spike glycoprotein | United Arab Emirates | Homo sapiens | 10/04/2014 |
| ASU45763.1 | Paden,C.R., Yusof,M.F.B.M., Al Hammadi,Z.M., Queen,K., Tao,Y., Eltahir,Y.M., Elsayed,E.A., Marzoug,B.A., Bensalah,O.K.A., Khalafalla,A.I., Al Mulla,M., Elkheir,K.A., Issa,Z.B., Pradeep,K., Elsaleh,F.N., Imambaccus,H., Sasse,J., Weber,S., Shi,M., Zhang,J., Li,Y., Pham,H., Kim,L., Hall,A.J., Gerber,S.I., Al Hosani,F.I., Tong,S., Al Muhairi,S.S.M., Al Muhairi,S.S., Al Hammadi,Z., Abou Elkheir,K., Al Bandar,Z., El Saleh,F., Hall,A., Gerber,S. | 2017-12-20T00:00:00Z | ssRNA(+) | 1353 | GenBank | spike glycoprotein | United Arab Emirates | Homo sapiens | 12/04/2014 |
| ASU45774.1 | Paden,C.R., Yusof,M.F.B.M., Al Hammadi,Z.M., Queen,K., Tao,Y., Eltahir,Y.M., Elsayed,E.A., Marzoug,B.A., Bensalah,O.K.A., Khalafalla,A.I., Al Mulla,M., Elkheir,K.A., Issa,Z.B., Pradeep,K., Elsaleh,F.N., Imambaccus,H., Sasse,J., Weber,S., Shi,M., Zhang,J., Li,Y., Pham,H., Kim,L., Hall,A.J., Gerber,S.I., Al Hosani,F.I., Tong,S., Al Muhairi,S.S.M., Al Muhairi,S.S., Al Hammadi,Z., Abou Elkheir,K., Al Bandar,Z., El Saleh,F., Hall,A., Gerber,S. | 2017-12-20T00:00:00Z | ssRNA(+) | 1353 | GenBank | spike glycoprotein | United Arab Emirates | Homo sapiens | 14/04/2014 |
| ASU45785.1 | Paden,C.R., Yusof,M.F.B.M., Al Hammadi,Z.M., Queen,K., Tao,Y., Eltahir,Y.M., Elsayed,E.A., Marzoug,B.A., Bensalah,O.K.A., Khalafalla,A.I., Al Mulla,M., Elkheir,K.A., Issa,Z.B., Pradeep,K., Elsaleh,F.N., Imambaccus,H., Sasse,J., Weber,S., Shi,M., Zhang,J., Li,Y., Pham,H., Kim,L., Hall,A.J., Gerber,S.I., Al Hosani,F.I., Tong,S., Al Muhairi,S.S.M., Al Muhairi,S.S., Al Hammadi,Z., Abou Elkheir,K., Al Bandar,Z., El Saleh,F., Hall,A., Gerber,S. | 2017-12-20T00:00:00Z | ssRNA(+) | 1353 | GenBank | spike glycoprotein | United Arab Emirates | Homo sapiens | 15/04/2014 |
| ASU45796.1 | Paden,C.R., Yusof,M.F.B.M., Al Hammadi,Z.M., Queen,K., Tao,Y., Eltahir,Y.M., Elsayed,E.A., Marzoug,B.A., Bensalah,O.K.A., Khalafalla,A.I., Al Mulla,M., Elkheir,K.A., Issa,Z.B., Pradeep,K., Elsaleh,F.N., Imambaccus,H., Sasse,J., Weber,S., Shi,M., Zhang,J., Li,Y., Pham,H., Kim,L., Hall,A.J., Gerber,S.I., Al Hosani,F.I., Tong,S., Al Muhairi,S.S.M., Al Muhairi,S.S., Al Hammadi,Z., Abou Elkheir,K., Al Bandar,Z., El Saleh,F., Hall,A., Gerber,S. | 2017-12-20T00:00:00Z | ssRNA(+) | 1353 | GenBank | spike glycoprotein | United Arab Emirates | Homo sapiens | 20/04/2014 |
| ASU45807.1 | Paden,C.R., Yusof,M.F.B.M., Al Hammadi,Z.M., Queen,K., Tao,Y., Eltahir,Y.M., Elsayed,E.A., Marzoug,B.A., Bensalah,O.K.A., Khalafalla,A.I., Al Mulla,M., Elkheir,K.A., Issa,Z.B., Pradeep,K., Elsaleh,F.N., Imambaccus,H., Sasse,J., Weber,S., Shi,M., Zhang,J., Li,Y., Pham,H., Kim,L., Hall,A.J., Gerber,S.I., Al Hosani,F.I., Tong,S., Al Muhairi,S.S.M., Al Muhairi,S.S., Al Hammadi,Z., Abou Elkheir,K., Al Bandar,Z., El Saleh,F., Hall,A., Gerber,S. | 2017-12-20T00:00:00Z | ssRNA(+) | 1353 | GenBank | spike glycoprotein | United Arab Emirates | Homo sapiens | 2014 |
| ATY74381.1 | Queen,K., Li,Y., Tao,Y., Zhang,J., Paden,C.R., Assiri,A.M., Tong,S. | 2017-12-06T00:00:00Z | ssRNA(+) | 1353 | GenBank | spike glycoprotein | Saudi Arabia | Homo sapiens | 02/06/2015 |
| ATY74392.1 | Queen,K., Li,Y., Tao,Y., Zhang,J., Paden,C.R., Assiri,A.M., Tong,S. | 2017-12-06T00:00:00Z | ssRNA(+) | 1353 | GenBank | spike glycoprotein | Saudi Arabia | Homo sapiens | 07/06/2015 |
| ATY74403.1 | Queen,K., Li,Y., Tao,Y., Zhang,J., Paden,C.R., Assiri,A.M., Tong,S. | 2017-12-06T00:00:00Z | ssRNA(+) | 1353 | GenBank | spike glycoprotein | Saudi Arabia | Homo sapiens | 07/06/2015 |
| ATY74414.1 | Queen,K., Li,Y., Tao,Y., Zhang,J., Paden,C.R., Assiri,A.M., Tong,S. | 2017-12-06T00:00:00Z | ssRNA(+) | 1353 | GenBank | spike glycoprotein | Saudi Arabia | Homo sapiens | 15/06/2015 |
| ALK80192.1 | Kim,Y., Cheon,S., Min,C.K., Sohn,K.M., Kang,Y.J., Cha,Y.J., Kang,J.I., Han,S.K., Ha,N.Y., Kim,G., Aigerim,A., Shin,H.M., Choi,M.S., Kim,S., Cho,H.S., Kim,Y.S., Cho,N.H., Kim,Y.-S., Cho,N.-H. | 2016-03-09T00:00:00Z | ssRNA(+) | 1353 | GenBank | spike glycoprotein | South Korea | Homo sapiens | 11/06/2015 |
| ALK80202.1 | Kim,Y., Cheon,S., Min,C.K., Sohn,K.M., Kang,Y.J., Cha,Y.J., Kang,J.I., Han,S.K., Ha,N.Y., Kim,G., Aigerim,A., Shin,H.M., Choi,M.S., Kim,S., Cho,H.S., Kim,Y.S., Cho,N.H., Kim,Y.-S., Cho,N.-H. | 2016-03-09T00:00:00Z | ssRNA(+) | 1353 | GenBank | spike glycoprotein | South Korea | Homo sapiens | 11/06/2015 |
| ALK80212.1 | Kim,Y., Cheon,S., Min,C.K., Sohn,K.M., Kang,Y.J., Cha,Y.J., Kang,J.I., Han,S.K., Ha,N.Y., Kim,G., Aigerim,A., Shin,H.M., Choi,M.S., Kim,S., Cho,H.S., Kim,Y.S., Cho,N.H., Kim,Y.-S., Cho,N.-H. | 2016-03-09T00:00:00Z | ssRNA(+) | 1353 | GenBank | spike glycoprotein | South Korea | Homo sapiens | 08/06/2015 |
| ALK80222.1 | Kim,Y., Cheon,S., Min,C.K., Sohn,K.M., Kang,Y.J., Cha,Y.J., Kang,J.I., Han,S.K., Ha,N.Y., Kim,G., Aigerim,A., Shin,H.M., Choi,M.S., Kim,S., Cho,H.S., Kim,Y.S., Cho,N.H., Kim,Y.-S., Cho,N.-H. | 2016-03-09T00:00:00Z | ssRNA(+) | 1353 | GenBank | spike glycoprotein | South Korea | Homo sapiens | 08/06/2015 |
| ALK80232.1 | Kim,Y., Cheon,S., Min,C.K., Sohn,K.M., Kang,Y.J., Cha,Y.J., Kang,J.I., Han,S.K., Ha,N.Y., Kim,G., Aigerim,A., Shin,H.M., Choi,M.S., Kim,S., Cho,H.S., Kim,Y.S., Cho,N.H., Kim,Y.-S., Cho,N.-H. | 2016-03-09T00:00:00Z | ssRNA(+) | 1353 | GenBank | spike glycoprotein | South Korea | Homo sapiens | 11/06/2015 |
| ALK80242.1 | Kim,Y., Cheon,S., Min,C.K., Sohn,K.M., Kang,Y.J., Cha,Y.J., Kang,J.I., Han,S.K., Ha,N.Y., Kim,G., Aigerim,A., Shin,H.M., Choi,M.S., Kim,S., Cho,H.S., Kim,Y.S., Cho,N.H., Kim,Y.-S., Cho,N.-H. | 2016-03-09T00:00:00Z | ssRNA(+) | 1353 | GenBank | spike glycoprotein | South Korea | Homo sapiens | 10/06/2015 |
| ALK80251.1 | Kim,Y., Cheon,S., Min,C.K., Sohn,K.M., Kang,Y.J., Cha,Y.J., Kang,J.I., Han,S.K., Ha,N.Y., Kim,G., Aigerim,A., Shin,H.M., Choi,M.S., Kim,S., Cho,H.S., Kim,Y.S., Cho,N.H., Kim,Y.-S., Cho,N.-H. | 2016-03-09T00:00:00Z | ssRNA(+) | 1353 | GenBank | spike glycoprotein | South Korea | Homo sapiens | 09/06/2015 |
| ALK80261.1 | Kim,Y., Cheon,S., Min,C.K., Sohn,K.M., Kang,Y.J., Cha,Y.J., Kang,J.I., Han,S.K., Ha,N.Y., Kim,G., Aigerim,A., Shin,H.M., Choi,M.S., Kim,S., Cho,H.S., Kim,Y.S., Cho,N.H., Kim,Y.-S., Cho,N.-H. | 2016-03-09T00:00:00Z | ssRNA(+) | 1353 | GenBank | spike glycoprotein | South Korea | Homo sapiens | 10/06/2015 |
| ALK80271.1 | Kim,Y., Cheon,S., Min,C.K., Sohn,K.M., Kang,Y.J., Cha,Y.J., Kang,J.I., Han,S.K., Ha,N.Y., Kim,G., Aigerim,A., Shin,H.M., Choi,M.S., Kim,S., Cho,H.S., Kim,Y.S., Cho,N.H., Kim,Y.-S., Cho,N.-H. | 2016-03-09T00:00:00Z | ssRNA(+) | 1353 | GenBank | spike glycoprotein | South Korea | Homo sapiens | 10/06/2015 |
| ALK80281.1 | Kim,Y., Cheon,S., Min,C.K., Sohn,K.M., Kang,Y.J., Cha,Y.J., Kang,J.I., Han,S.K., Ha,N.Y., Kim,G., Aigerim,A., Shin,H.M., Choi,M.S., Kim,S., Cho,H.S., Kim,Y.S., Cho,N.H., Kim,Y.-S., Cho,N.-H. | 2016-03-09T00:00:00Z | ssRNA(+) | 1353 | GenBank | spike glycoprotein | South Korea | Homo sapiens | 11/06/2015 |
| ALK80291.1 | Kim,Y., Cheon,S., Min,C.K., Sohn,K.M., Kang,Y.J., Cha,Y.J., Kang,J.I., Han,S.K., Ha,N.Y., Kim,G., Aigerim,A., Shin,H.M., Choi,M.S., Kim,S., Cho,H.S., Kim,Y.S., Cho,N.H., Kim,Y.-S., Cho,N.-H. | 2016-03-09T00:00:00Z | ssRNA(+) | 1353 | GenBank | spike glycoprotein | South Korea | Homo sapiens | 10/06/2015 |
| ALK80301.1 | Kim,Y., Cheon,S., Min,C.K., Sohn,K.M., Kang,Y.J., Cha,Y.J., Kang,J.I., Han,S.K., Ha,N.Y., Kim,G., Aigerim,A., Shin,H.M., Choi,M.S., Kim,S., Cho,H.S., Kim,Y.S., Cho,N.H., Kim,Y.-S., Cho,N.-H. | 2016-03-09T00:00:00Z | ssRNA(+) | 1353 | GenBank | spike glycoprotein | South Korea | Homo sapiens | 10/06/2015 |
| ALK80311.1 | Kim,Y., Cheon,S., Min,C.K., Sohn,K.M., Kang,Y.J., Cha,Y.J., Kang,J.I., Han,S.K., Ha,N.Y., Kim,G., Aigerim,A., Shin,H.M., Choi,M.S., Kim,S., Cho,H.S., Kim,Y.S., Cho,N.H., Kim,Y.-S., Cho,N.-H. | 2016-03-09T00:00:00Z | ssRNA(+) | 1353 | GenBank | spike glycoprotein | South Korea | Homo sapiens | 22/06/2015 |
| AMD02841.1 | Almajhdi,F.N., Amer,H.M., Hajomar,W., Farrag,M.A., Assiri,A., BinSaeed,A. | 2016-02-07T00:00:00Z | ssRNA(+) | 890 | GenBank | spike glycoprotein | Saudi Arabia | Homo sapiens | 15/12/2008 |
| AMD02842.1 | Almajhdi,F.N., Amer,H.M., Hajomar,W., Farrag,M.A., Assiri,A., BinSaeed,A. | 2016-02-07T00:00:00Z | ssRNA(+) | 890 | GenBank | spike glycoprotein | Saudi Arabia | Homo sapiens | 15/12/2008 |
| AMD02843.1 | Almajhdi,F.N., Amer,H.M., Hajomar,W., Farrag,M.A., Assiri,A., BinSaeed,A. | 2016-02-07T00:00:00Z | ssRNA(+) | 890 | GenBank | spike glycoprotein | Saudi Arabia | Homo sapiens | 15/12/2008 |
| AMD02844.1 | Almajhdi,F.N., Amer,H.M., Hajomar,W., Farrag,M.A., Assiri,A., BinSaeed,A. | 2016-02-07T00:00:00Z | ssRNA(+) | 890 | GenBank | spike glycoprotein | Saudi Arabia | Homo sapiens | 15/12/2008 |
| AGN52936.1 | Drosten,C., Seilmaier,M., Corman,V.M., Hartmann,W., Scheible,G., Sack,S., Guggemos,W., Kallies,R., Muth,D., Junglen,S., Muller,M.A., Haas,W., Guberina,H., Rohnisch,T., Schmid-Wendtner,M., Aldabbagh,S., Dittmer,U., Gold,H., Graf,P., Bonin,F., Rambaut,A., Wendtner,C.M. | 2013-06-10T00:00:00Z | ssRNA(+) | 1353 | GenBank | spike glycoprotein | United Arab Emirates | Homo sapiens | 2013 |
| YP_009047204.1 | Zaki,A.M., van Boheemen,S., Bestebroer,T.M., Osterhaus,A.D., Fouchier,R.A., de Graaf,M., Lauber,C., Raj,V.S., Haagmans,B.L., Gorbalenya,A.E., Snijder,E.J., Victor,S.R., Osterhaus,A.D.M.E., Fouchier,R.A.M. | 2014-07-23T00:00:00Z | ssRNA(+) | 1353 | RefSeq | spike protein | | Homo sapiens | 13/06/2012 |
| YP_007188579.1 | de Groot,R.J., Baker,S.C., Baric,R.S., Brown,C.S., Drosten,C., Enjuanes,L., Fouchier,R.A., Galiano,M., Gorbalenya,A.E., Memish,Z.A., Perlman,S., Poon,L.L., Snijder,E.J., Stephens,G.M., Woo,P.C., Zaki,A.M., Zambon,M., Ziebuhr,J., Cotten,M., Lam,T.T., Watson,S.J., Palser,A.L., Petrova,V., Grant,P., Pybus,O.G., Rambaut,A., Guan,Y., Pillay,D., Kellam,P., Nastouli,E., Myers,R., Bermingham,A., Gopal,R. | 2012-12-13T00:00:00Z | ssRNA(+) | 1353 | RefSeq | spike protein | United Kingdom | Homo sapiens | 11/09/2012 |
| UHI99911.1 | Aljabr W, Alruwaili M, Penrice-Randal R, Alrezaihi A, Harrison AJ, Ryan Y, Bentley E, Jones B, Alhatlani BY, AlShahrani D, Mahmood Z, Rickett NY, Alosaimi B, Naeem A, Alamri S, Alsran H, Hamed ME, Dong X, Assiri AM, Alrasheed AR, Hamza M, Carroll MW, Gemmell M, Darby A, Donovan-Banfield I, Stewart JP, Matthews DA, Davidson AD, Hiscox JA, Aljabr,W., Penrice-Randal,R., Alruwaili,M., Hiscox,J.A. | 2021-12-25T00:00:00Z | ssRNA(+) | 1353 | GenBank | spike protein | Saudi Arabia | Homo sapiens | 03/01/2017 |
| UHI99922.1 | Aljabr W, Alruwaili M, Penrice-Randal R, Alrezaihi A, Harrison AJ, Ryan Y, Bentley E, Jones B, Alhatlani BY, AlShahrani D, Mahmood Z, Rickett NY, Alosaimi B, Naeem A, Alamri S, Alsran H, Hamed ME, Dong X, Assiri AM, Alrasheed AR, Hamza M, Carroll MW, Gemmell M, Darby A, Donovan-Banfield I, Stewart JP, Matthews DA, Davidson AD, Hiscox JA, Aljabr,W., Penrice-Randal,R., Alruwaili,M., Hiscox,J.A. | 2021-12-25T00:00:00Z | ssRNA(+) | 1353 | GenBank | spike protein | Saudi Arabia | Homo sapiens | 03/04/2019 |
| QEU56412.1 | Sohrab,S.S., Azhar,E.I. | 2019-10-01T00:00:00Z | ssRNA(+) | 1353 | GenBank | spike protein | Saudi Arabia | Homo sapiens | 2019-03 |
| QCQ29075.1 | Kim,S., Yi,H., Kim,K. | 2019-05-20T00:00:00Z | ssRNA(+) | 1353 | GenBank | spike protein | South Korea | Homo sapiens | 20/05/2015 |
| QBF80607.1 | Lu,X., Assiri,A.M., Watson,J.T., Gerber,S.I., Lindstrom,S. | 2019-03-02T00:00:00Z | ssRNA(+) | 1353 | GenBank | spike protein | Saudi Arabia | Homo sapiens | 01/06/2018 |
| QBF80608.1 | Lu,X., Assiri,A.M., Watson,J.T., Gerber,S.I., Lindstrom,S. | 2019-03-02T00:00:00Z | ssRNA(+) | 1353 | GenBank | spike protein | Saudi Arabia | Homo sapiens | 24/07/2018 |
| AZK15900.1 | Chung,Y.S., Kim,J.M., Man Kim,H., Park,K.R., Lee,A., Lee,N.J., Kim,M.S., Kim,J.S., Kim,C.K., Lee,J.I., Kang,C., Chung,Y.-S., Kim,J.-M., Kim,H.M., Kim,C.-K., Kim,J.-S. | 2019-02-26T00:00:00Z | ssRNA(+) | 1353 | GenBank | spike protein | South Korea | Homo sapiens | 20/10/2018 |
| QBF44113.1 | Chung,Y.S., Kim,J.M., Man Kim,H., Park,K.R., Lee,A., Lee,N.J., Kim,M.S., Kim,J.S., Kim,C.K., Lee,J.I., Kang,C., Chung,Y.-S., Kim,J.-M., Kim,H.M., Lee,N., Kim,J.-S., Lee,J.-I. | 2019-02-26T00:00:00Z | ssRNA(+) | 1353 | GenBank | spike protein | South Korea | Homo sapiens | 08/09/2018 |
| QBF44114.1 | Chung,Y.S., Kim,J.M., Man Kim,H., Park,K.R., Lee,A., Lee,N.J., Kim,M.S., Kim,J.S., Kim,C.K., Lee,J.I., Kang,C., Chung,Y.-S., Kim,J.-M., Kim,H.M., Lee,N., Kim,J.-S., Lee,J.-I. | 2019-02-26T00:00:00Z | ssRNA(+) | 1353 | GenBank | spike protein | South Korea | Homo sapiens | 08/09/2018 |
| QBF44115.1 | Chung,Y.-S., Kim,J.-M., Kim,H.M., Kim,C.-K., Lee,A., Kim,J.-S., Kang,C. | 2019-02-26T00:00:00Z | ssRNA(+) | 1353 | GenBank | spike protein | South Korea | Homo sapiens | 12/09/2018 |
| ALX27225.1 | Lu,X., Al-Abdallat,M.M., Haddadin,A., Al-Sanouri,T., Erdman,D.D. | 2016-09-30T00:00:00Z | ssRNA(+) | 1353 | GenBank | spike protein | Jordan | Homo sapiens | 07/04/2014 |
| ALX27226.1 | Lu,X., Al-Abdallat,M.M., Haddadin,A., Al-Sanouri,T., Erdman,D.D. | 2016-09-30T00:00:00Z | ssRNA(+) | 1353 | GenBank | spike protein | Jordan | Homo sapiens | 30/04/2014 |
| ALX27227.1 | Lu,X., Al-Abdallat,M.M., Haddadin,A., Al-Sanouri,T., Erdman,D.D. | 2016-09-30T00:00:00Z | ssRNA(+) | 1353 | GenBank | spike protein | Jordan | Homo sapiens | 10/05/2014 |
| ALX27228.1 | Lu,X., Al-Abdallat,M.M., Haddadin,A., Al-Sanouri,T., Erdman,D.D. | 2016-09-30T00:00:00Z | ssRNA(+) | 1353 | GenBank | spike protein | Jordan | Homo sapiens | 24/12/2014 |
| ALX27229.1 | Lu,X., Al-Abdallat,M.M., Haddadin,A., Al-Sanouri,T., Erdman,D.D. | 2016-09-30T00:00:00Z | ssRNA(+) | 1353 | GenBank | spike protein | Jordan | Homo sapiens | 04/05/2014 |
| ANC28634.1 | Park,D., Huh,H.J., Kim,Y.J., Son,D.-S., Jeon,H.-J., Im,E.-H., Kim,J.-W., Lee,N.Y., Kang,E.-S., Chung,D.R., Choi,S.S., Ahn,J.-H., Peck,K.R., Ki,C.-S., Park,W.-Y. | 2016-05-08T00:00:00Z | ssRNA(+) | 1353 | GenBank | spike protein | South Korea | Homo sapiens | 30/05/2015 |
| ANC28645.1 | Park,D., Huh,H.J., Kim,Y.J., Son,D.-S., Jeon,H.-J., Im,E.-H., Kim,J.-W., Lee,N.Y., Kang,E.-S., Chung,D.R., Choi,S.S., Ahn,J.-H., Peck,K.R., Ki,C.-S., Park,W.-Y. | 2016-05-08T00:00:00Z | ssRNA(+) | 1353 | GenBank | spike protein | South Korea | Homo sapiens | 11/06/2015 |
| ANC28656.1 | Park,D., Huh,H.J., Kim,Y.J., Son,D.-S., Jeon,H.-J., Im,E.-H., Kim,J.-W., Lee,N.Y., Kang,E.-S., Chung,D.R., Choi,S.S., Ahn,J.-H., Peck,K.R., Ki,C.-S., Park,W.-Y. | 2016-05-08T00:00:00Z | ssRNA(+) | 1353 | GenBank | spike protein | South Korea | Homo sapiens | 04/06/2015 |
| ANC28667.1 | Park,D., Huh,H.J., Kim,Y.J., Son,D.-S., Jeon,H.-J., Im,E.-H., Kim,J.-W., Lee,N.Y., Kang,E.-S., Chung,D.R., Choi,S.S., Ahn,J.-H., Peck,K.R., Ki,C.-S., Park,W.-Y. | 2016-05-08T00:00:00Z | ssRNA(+) | 1353 | GenBank | spike protein | South Korea | Homo sapiens | 17/06/2015 |
| ANC28678.1 | Park,D., Huh,H.J., Kim,Y.J., Son,D.-S., Jeon,H.-J., Im,E.-H., Kim,J.-W., Lee,N.Y., Kang,E.-S., Chung,D.R., Choi,S.S., Ahn,J.-H., Peck,K.R., Ki,C.-S., Park,W.-Y. | 2016-05-08T00:00:00Z | ssRNA(+) | 1353 | GenBank | spike protein | South Korea | Homo sapiens | 17/06/2015 |
| ANC28689.1 | Park,D., Huh,H.J., Kim,Y.J., Son,D.-S., Jeon,H.-J., Im,E.-H., Kim,J.-W., Lee,N.Y., Kang,E.-S., Chung,D.R., Choi,S.S., Ahn,J.-H., Peck,K.R., Ki,C.-S., Park,W.-Y. | 2016-05-08T00:00:00Z | ssRNA(+) | 1353 | GenBank | spike protein | South Korea | Homo sapiens | 22/06/2015 |
| ANC28700.1 | Park,D., Huh,H.J., Kim,Y.J., Son,D.-S., Jeon,H.-J., Im,E.-H., Kim,J.-W., Lee,N.Y., Kang,E.-S., Chung,D.R., Choi,S.S., Ahn,J.-H., Peck,K.R., Ki,C.-S., Park,W.-Y. | 2016-05-08T00:00:00Z | ssRNA(+) | 1353 | GenBank | spike protein | South Korea | Homo sapiens | 26/06/2015 |
| ANC28711.1 | Park,D., Huh,H.J., Kim,Y.J., Son,D.-S., Jeon,H.-J., Im,E.-H., Kim,J.-W., Lee,N.Y., Kang,E.-S., Chung,D.R., Choi,S.S., Ahn,J.-H., Peck,K.R., Ki,C.-S., Park,W.-Y. | 2016-05-08T00:00:00Z | ssRNA(+) | 1353 | GenBank | spike protein | South Korea | Homo sapiens | 03/07/2015 |
| AMW90836.1 | Corman,V.M., Albarrak,A.M., Omrani,A.S., Albarrak,M.M., Farah,M.E., Almasri,M., Muth,D., Sieberg,A., Meyer,B., Assiri,A.M., Binger,T., Steinhagen,K., Lattwein,E., Al-Tawfiq,J., Muller,M.A., Drosten,C., Memish,Z.A. | 2016-04-12T00:00:00Z | ssRNA(+) | 912 | GenBank | spike protein | Saudi Arabia | Homo sapiens | 2014 |
| AMW90837.1 | Corman,V.M., Albarrak,A.M., Omrani,A.S., Albarrak,M.M., Farah,M.E., Almasri,M., Muth,D., Sieberg,A., Meyer,B., Assiri,A.M., Binger,T., Steinhagen,K., Lattwein,E., Al-Tawfiq,J., Muller,M.A., Drosten,C., Memish,Z.A. | 2016-04-12T00:00:00Z | ssRNA(+) | 232 | GenBank | spike protein | Saudi Arabia | Homo sapiens | 2014 |
| AMW90838.1 | Corman,V.M., Albarrak,A.M., Omrani,A.S., Albarrak,M.M., Farah,M.E., Almasri,M., Muth,D., Sieberg,A., Meyer,B., Assiri,A.M., Binger,T., Steinhagen,K., Lattwein,E., Al-Tawfiq,J., Muller,M.A., Drosten,C., Memish,Z.A. | 2016-04-12T00:00:00Z | ssRNA(+) | 599 | GenBank | spike protein | Saudi Arabia | Homo sapiens | 2014 |
| AMW90839.1 | Corman,V.M., Albarrak,A.M., Omrani,A.S., Albarrak,M.M., Farah,M.E., Almasri,M., Muth,D., Sieberg,A., Meyer,B., Assiri,A.M., Binger,T., Steinhagen,K., Lattwein,E., Al-Tawfiq,J., Muller,M.A., Drosten,C., Memish,Z.A. | 2016-04-12T00:00:00Z | ssRNA(+) | 835 | GenBank | spike protein | Saudi Arabia | Homo sapiens | 2014 |
| AMW90840.1 | Corman,V.M., Albarrak,A.M., Omrani,A.S., Albarrak,M.M., Farah,M.E., Almasri,M., Muth,D., Sieberg,A., Meyer,B., Assiri,A.M., Binger,T., Steinhagen,K., Lattwein,E., Al-Tawfiq,J., Muller,M.A., Drosten,C., Memish,Z.A. | 2016-04-12T00:00:00Z | ssRNA(+) | 96 | GenBank | spike protein | Saudi Arabia | Homo sapiens | 2014 |
| AMW90841.1 | Corman,V.M., Albarrak,A.M., Omrani,A.S., Albarrak,M.M., Farah,M.E., Almasri,M., Muth,D., Sieberg,A., Meyer,B., Assiri,A.M., Binger,T., Steinhagen,K., Lattwein,E., Al-Tawfiq,J., Muller,M.A., Drosten,C., Memish,Z.A. | 2016-04-12T00:00:00Z | ssRNA(+) | 585 | GenBank | spike protein | Saudi Arabia | Homo sapiens | 2014 |
| AMW90842.1 | Corman,V.M., Albarrak,A.M., Omrani,A.S., Albarrak,M.M., Farah,M.E., Almasri,M., Muth,D., Sieberg,A., Meyer,B., Assiri,A.M., Binger,T., Steinhagen,K., Lattwein,E., Al-Tawfiq,J., Muller,M.A., Drosten,C., Memish,Z.A. | 2016-04-12T00:00:00Z | ssRNA(+) | 96 | GenBank | spike protein | Saudi Arabia | Homo sapiens | 2014 |
| AMW90843.1 | Corman,V.M., Albarrak,A.M., Omrani,A.S., Albarrak,M.M., Farah,M.E., Almasri,M., Muth,D., Sieberg,A., Meyer,B., Assiri,A.M., Binger,T., Steinhagen,K., Lattwein,E., Al-Tawfiq,J., Muller,M.A., Drosten,C., Memish,Z.A. | 2016-04-12T00:00:00Z | ssRNA(+) | 1089 | GenBank | spike protein | Saudi Arabia | Homo sapiens | 2014 |
| AMW90844.1 | Corman,V.M., Albarrak,A.M., Omrani,A.S., Albarrak,M.M., Farah,M.E., Almasri,M., Muth,D., Sieberg,A., Meyer,B., Assiri,A.M., Binger,T., Steinhagen,K., Lattwein,E., Al-Tawfiq,J., Muller,M.A., Drosten,C., Memish,Z.A. | 2016-04-12T00:00:00Z | ssRNA(+) | 1353 | GenBank | spike protein | Saudi Arabia | Homo sapiens | 2014 |
| AMW90845.1 | Corman,V.M., Albarrak,A.M., Omrani,A.S., Albarrak,M.M., Farah,M.E., Almasri,M., Muth,D., Sieberg,A., Meyer,B., Assiri,A.M., Binger,T., Steinhagen,K., Lattwein,E., Al-Tawfiq,J., Muller,M.A., Drosten,C., Memish,Z.A. | 2016-04-12T00:00:00Z | ssRNA(+) | 1353 | GenBank | spike protein | Saudi Arabia | Homo sapiens | 2014 |
| AMW90846.1 | Corman,V.M., Albarrak,A.M., Omrani,A.S., Albarrak,M.M., Farah,M.E., Almasri,M., Muth,D., Sieberg,A., Meyer,B., Assiri,A.M., Binger,T., Steinhagen,K., Lattwein,E., Al-Tawfiq,J., Muller,M.A., Drosten,C., Memish,Z.A. | 2016-04-12T00:00:00Z | ssRNA(+) | 1353 | GenBank | spike protein | Saudi Arabia | Homo sapiens | 2014 |
| AMW90847.1 | Corman,V.M., Albarrak,A.M., Omrani,A.S., Albarrak,M.M., Farah,M.E., Almasri,M., Muth,D., Sieberg,A., Meyer,B., Assiri,A.M., Binger,T., Steinhagen,K., Lattwein,E., Al-Tawfiq,J., Muller,M.A., Drosten,C., Memish,Z.A. | 2016-04-12T00:00:00Z | ssRNA(+) | 1353 | GenBank | spike protein | Saudi Arabia | Homo sapiens | 2014 |
| AMW90848.1 | Corman,V.M., Albarrak,A.M., Omrani,A.S., Albarrak,M.M., Farah,M.E., Almasri,M., Muth,D., Sieberg,A., Meyer,B., Assiri,A.M., Binger,T., Steinhagen,K., Lattwein,E., Al-Tawfiq,J., Muller,M.A., Drosten,C., Memish,Z.A. | 2016-04-12T00:00:00Z | ssRNA(+) | 1353 | GenBank | spike protein | Saudi Arabia | Homo sapiens | 2014 |
| AMW90849.1 | Corman,V.M., Albarrak,A.M., Omrani,A.S., Albarrak,M.M., Farah,M.E., Almasri,M., Muth,D., Sieberg,A., Meyer,B., Assiri,A.M., Binger,T., Steinhagen,K., Lattwein,E., Al-Tawfiq,J., Muller,M.A., Drosten,C., Memish,Z.A. | 2016-04-12T00:00:00Z | ssRNA(+) | 1353 | GenBank | spike protein | Saudi Arabia | Homo sapiens | 2014 |
| AMW90850.1 | Corman,V.M., Albarrak,A.M., Omrani,A.S., Albarrak,M.M., Farah,M.E., Almasri,M., Muth,D., Sieberg,A., Meyer,B., Assiri,A.M., Binger,T., Steinhagen,K., Lattwein,E., Al-Tawfiq,J., Muller,M.A., Drosten,C., Memish,Z.A. | 2016-04-12T00:00:00Z | ssRNA(+) | 1353 | GenBank | spike protein | Saudi Arabia | Homo sapiens | 2014 |
| AMW90851.1 | Corman,V.M., Albarrak,A.M., Omrani,A.S., Albarrak,M.M., Farah,M.E., Almasri,M., Muth,D., Sieberg,A., Meyer,B., Assiri,A.M., Binger,T., Steinhagen,K., Lattwein,E., Al-Tawfiq,J., Muller,M.A., Drosten,C., Memish,Z.A. | 2016-04-12T00:00:00Z | ssRNA(+) | 1353 | GenBank | spike protein | Saudi Arabia | Homo sapiens | 2014 |
| AMW90852.1 | Corman,V.M., Albarrak,A.M., Omrani,A.S., Albarrak,M.M., Farah,M.E., Almasri,M., Muth,D., Sieberg,A., Meyer,B., Assiri,A.M., Binger,T., Steinhagen,K., Lattwein,E., Al-Tawfiq,J., Muller,M.A., Drosten,C., Memish,Z.A. | 2016-04-12T00:00:00Z | ssRNA(+) | 1353 | GenBank | spike protein | Saudi Arabia | Homo sapiens | 2014 |
| AMW90853.1 | Corman,V.M., Albarrak,A.M., Omrani,A.S., Albarrak,M.M., Farah,M.E., Almasri,M., Muth,D., Sieberg,A., Meyer,B., Assiri,A.M., Binger,T., Steinhagen,K., Lattwein,E., Al-Tawfiq,J., Muller,M.A., Drosten,C., Memish,Z.A. | 2016-04-12T00:00:00Z | ssRNA(+) | 1353 | GenBank | spike protein | Saudi Arabia | Homo sapiens | 2014 |
| AMW90854.1 | Corman,V.M., Albarrak,A.M., Omrani,A.S., Albarrak,M.M., Farah,M.E., Almasri,M., Muth,D., Sieberg,A., Meyer,B., Assiri,A.M., Binger,T., Steinhagen,K., Lattwein,E., Al-Tawfiq,J., Muller,M.A., Drosten,C., Memish,Z.A. | 2016-04-12T00:00:00Z | ssRNA(+) | 1353 | GenBank | spike protein | Saudi Arabia | Homo sapiens | 2014 |
| AKN11071.1 | Kim,D.W., Kim,Y.J., Park,S.H., Yun,M.R., Yang,J.S., Kang,H.J., Han,Y.W., Lee,H.S., Man Kim,H., Kim,H., Kim,A.R., Heo,D.R., Kim,S.J., Jeon,J.H., Park,D., Kim,J.A., Cheong,H.M., Nam,J.G., Kim,K., Kim,S.S., Nam,J.-G., Kim,Y.-J., Cheong,H.-M., Yang,J.-S., Park,S., Kim,H.M., Kim,D.-W., Yun,M.-R. | 2016-04-07T00:00:00Z | ssRNA(+) | 1353 | GenBank | spike protein | South Korea | Homo sapiens | 28/05/2015 |
| AKN11072.1 | Kim,D.W., Kim,Y.J., Park,S.H., Yun,M.R., Yang,J.S., Kang,H.J., Han,Y.W., Lee,H.S., Man Kim,H., Kim,H., Kim,A.R., Heo,D.R., Kim,S.J., Jeon,J.H., Park,D., Kim,J.A., Cheong,H.M., Nam,J.G., Kim,K., Kim,S.S., Nam,J.-G., Kim,Y.-J., Cheong,H.-M., Yang,J.-S., Park,S., Kim,H.M., Kim,D.-W., Yun,M.-R. | 2016-04-07T00:00:00Z | ssRNA(+) | 1353 | GenBank | spike protein | South Korea | Homo sapiens | 28/05/2015 |
| AKN11073.1 | Kim,D.W., Kim,Y.J., Park,S.H., Yun,M.R., Yang,J.S., Kang,H.J., Han,Y.W., Lee,H.S., Man Kim,H., Kim,H., Kim,A.R., Heo,D.R., Kim,S.J., Jeon,J.H., Park,D., Kim,J.A., Cheong,H.M., Nam,J.G., Kim,K., Kim,S.S., Nam,J.-G., Kim,Y.-J., Cheong,H.-M., Yang,J.-S., Park,S., Kim,H.M., Kim,D.-W., Yun,M.-R. | 2016-04-07T00:00:00Z | ssRNA(+) | 1353 | GenBank | spike protein | South Korea | Homo sapiens | 28/05/2015 |
| AKN11074.1 | Kim,D.W., Kim,Y.J., Park,S.H., Yun,M.R., Yang,J.S., Kang,H.J., Han,Y.W., Lee,H.S., Man Kim,H., Kim,H., Kim,A.R., Heo,D.R., Kim,S.J., Jeon,J.H., Park,D., Kim,J.A., Cheong,H.M., Nam,J.G., Kim,K., Kim,S.S., Nam,J.-G., Kim,Y.-J., Cheong,H.-M., Yang,J.-S., Park,S., Kim,H.M., Kim,D.-W., Yun,M.-R. | 2016-04-07T00:00:00Z | ssRNA(+) | 1353 | GenBank | spike protein | South Korea | Homo sapiens | 30/05/2015 |
| AKN11075.1 | Kim,D.W., Kim,Y.J., Park,S.H., Yun,M.R., Yang,J.S., Kang,H.J., Han,Y.W., Lee,H.S., Man Kim,H., Kim,H., Kim,A.R., Heo,D.R., Kim,S.J., Jeon,J.H., Park,D., Kim,J.A., Cheong,H.M., Nam,J.G., Kim,K., Kim,S.S., Nam,J.-G., Kim,Y.-J., Cheong,H.-M., Yang,J.-S., Park,S., Kim,H.M., Kim,D.-W., Yun,M.-R. | 2016-04-07T00:00:00Z | ssRNA(+) | 1353 | GenBank | spike protein | South Korea | Homo sapiens | 30/05/2015 |
| AKN11076.1 | Kim,D.W., Kim,Y.J., Park,S.H., Yun,M.R., Yang,J.S., Kang,H.J., Han,Y.W., Lee,H.S., Man Kim,H., Kim,H., Kim,A.R., Heo,D.R., Kim,S.J., Jeon,J.H., Park,D., Kim,J.A., Cheong,H.M., Nam,J.G., Kim,K., Kim,S.S., Nam,J.-G., Kim,Y.-J., Cheong,H.-M., Yang,J.-S., Park,S., Kim,H.M., Kim,D.-W., Yun,M.-R. | 2016-04-07T00:00:00Z | ssRNA(+) | 1329 | GenBank | spike protein | South Korea | Homo sapiens | 19/05/2015 |
| ALT66802.1 | Lamers,M.M., Raj,V.S., Shafei,M., Ali,S.S., Abdallh,S.M., Gazo,M., Nofal,S., Lu,X., Erdman,D.D., Koopmans,M.P., Abdallat,M., Haddadin,A., Haagmans,B.L., Stalin Raj,V., Koopmans,M.P.G. | 2016-04-07T00:00:00Z | ssRNA(+) | 1353 | GenBank | spike protein | Jordan | Homo sapiens | 2015 |
| ALT66813.1 | Lamers,M.M., Raj,V.S., Shafei,M., Ali,S.S., Abdallh,S.M., Gazo,M., Nofal,S., Lu,X., Erdman,D.D., Koopmans,M.P., Abdallat,M., Haddadin,A., Haagmans,B.L., Stalin Raj,V., Koopmans,M.P.G. | 2016-04-07T00:00:00Z | ssRNA(+) | 1353 | GenBank | spike protein | Jordan | Homo sapiens | 2015 |
| ALT66824.1 | Lamers,M.M., Raj,V.S., Shafei,M., Ali,S.S., Abdallh,S.M., Gazo,M., Nofal,S., Lu,X., Erdman,D.D., Koopmans,M.P., Abdallat,M., Haddadin,A., Haagmans,B.L., Stalin Raj,V., Koopmans,M.P.G. | 2016-04-07T00:00:00Z | ssRNA(+) | 1353 | GenBank | spike protein | Jordan | Homo sapiens | 2015 |
| ALJ54441.1 | Lu,X., Assiri,A.M., Erdman,D.D. | 2016-01-07T00:00:00Z | ssRNA(+) | 1353 | GenBank | spike protein | Saudi Arabia | Homo sapiens | 14/01/2015 |
| ALJ54442.1 | Lu,X., Assiri,A.M., Erdman,D.D. | 2016-01-07T00:00:00Z | ssRNA(+) | 1353 | GenBank | spike protein | Saudi Arabia | Homo sapiens | 18/01/2015 |
| ALJ54443.1 | Lu,X., Assiri,A.M., Erdman,D.D. | 2016-01-07T00:00:00Z | ssRNA(+) | 1353 | GenBank | spike protein | Saudi Arabia | Homo sapiens | 20/01/2015 |
| ALJ54444.1 | Lu,X., Assiri,A.M., Erdman,D.D. | 2016-01-07T00:00:00Z | ssRNA(+) | 1353 | GenBank | spike protein | Saudi Arabia | Homo sapiens | 02/02/2015 |
| ALJ54445.1 | Lu,X., Assiri,A.M., Erdman,D.D. | 2016-01-07T00:00:00Z | ssRNA(+) | 1353 | GenBank | spike protein | Saudi Arabia | Homo sapiens | 03/02/2015 |
| ALJ54446.1 | Lu,X., Assiri,A.M., Erdman,D.D. | 2016-01-07T00:00:00Z | ssRNA(+) | 1353 | GenBank | spike protein | Saudi Arabia | Homo sapiens | 20/02/2015 |
| ALJ54447.1 | Lu,X., Assiri,A.M., Erdman,D.D. | 2016-01-07T00:00:00Z | ssRNA(+) | 1353 | GenBank | spike protein | Saudi Arabia | Homo sapiens | 18/02/2015 |
| ALJ54448.1 | Lu,X., Assiri,A.M., Erdman,D.D. | 2016-01-07T00:00:00Z | ssRNA(+) | 1353 | GenBank | spike protein | Saudi Arabia | Homo sapiens | 15/02/2015 |
| ALJ54449.1 | Lu,X., Assiri,A.M., Erdman,D.D. | 2016-01-07T00:00:00Z | ssRNA(+) | 1353 | GenBank | spike protein | Saudi Arabia | Homo sapiens | 14/02/2015 |
| ALJ54450.1 | Lu,X., Assiri,A.M., Erdman,D.D. | 2016-01-07T00:00:00Z | ssRNA(+) | 1353 | GenBank | spike protein | Saudi Arabia | Homo sapiens | 12/02/2015 |
| ALJ54451.1 | Lu,X., Assiri,A.M., Erdman,D.D. | 2016-01-07T00:00:00Z | ssRNA(+) | 1353 | GenBank | spike protein | Saudi Arabia | Homo sapiens | 14/02/2015 |
| ALJ54452.1 | Lu,X., Assiri,A.M., Erdman,D.D. | 2016-01-07T00:00:00Z | ssRNA(+) | 1353 | GenBank | spike protein | Saudi Arabia | Homo sapiens | 14/02/2015 |
| ALJ54453.1 | Lu,X., Assiri,A.M., Erdman,D.D. | 2016-01-07T00:00:00Z | ssRNA(+) | 1353 | GenBank | spike protein | Saudi Arabia | Homo sapiens | 06/02/2015 |
| ALJ54454.1 | Lu,X., Assiri,A.M., Erdman,D.D. | 2016-01-07T00:00:00Z | ssRNA(+) | 1353 | GenBank | spike protein | Saudi Arabia | Homo sapiens | 28/02/2015 |
| ALJ54455.1 | Lu,X., Assiri,A.M., Erdman,D.D. | 2016-01-07T00:00:00Z | ssRNA(+) | 1353 | GenBank | spike protein | Saudi Arabia | Homo sapiens | 28/02/2015 |
| ALJ54456.1 | Lu,X., Assiri,A.M., Erdman,D.D. | 2016-01-07T00:00:00Z | ssRNA(+) | 1353 | GenBank | spike protein | Saudi Arabia | Homo sapiens | 26/02/2015 |
| ALJ54457.1 | Lu,X., Assiri,A.M., Erdman,D.D. | 2016-01-07T00:00:00Z | ssRNA(+) | 1353 | GenBank | spike protein | Saudi Arabia | Homo sapiens | 23/02/2015 |
| ALJ54458.1 | Lu,X., Assiri,A.M., Erdman,D.D. | 2016-01-07T00:00:00Z | ssRNA(+) | 1353 | GenBank | spike protein | Saudi Arabia | Homo sapiens | 23/02/2015 |
| ALJ54459.1 | Lu,X., Assiri,A.M., Erdman,D.D. | 2016-01-07T00:00:00Z | ssRNA(+) | 1353 | GenBank | spike protein | Saudi Arabia | Homo sapiens | 21/02/2015 |
| ALJ54460.1 | Lu,X., Assiri,A.M., Erdman,D.D. | 2016-01-07T00:00:00Z | ssRNA(+) | 1353 | GenBank | spike protein | Saudi Arabia | Homo sapiens | 21/02/2015 |
| ALJ54461.1 | Lu,X., Assiri,A.M., Erdman,D.D. | 2016-01-07T00:00:00Z | ssRNA(+) | 1353 | GenBank | spike protein | Saudi Arabia | Homo sapiens | 10/02/2015 |
| ALJ54462.1 | Lu,X., Assiri,A.M., Erdman,D.D. | 2016-01-07T00:00:00Z | ssRNA(+) | 1353 | GenBank | spike protein | Saudi Arabia | Homo sapiens | 30/01/2015 |
| ALJ54463.1 | Lu,X., Assiri,A.M., Erdman,D.D. | 2016-01-07T00:00:00Z | ssRNA(+) | 1353 | GenBank | spike protein | Saudi Arabia | Homo sapiens | 01/02/2015 |
| ALJ54464.1 | Lu,X., Assiri,A.M., Erdman,D.D. | 2016-01-07T00:00:00Z | ssRNA(+) | 1353 | GenBank | spike protein | Saudi Arabia | Homo sapiens | 08/02/2015 |
| ALJ54465.1 | Lu,X., Assiri,A.M., Erdman,D.D. | 2016-01-07T00:00:00Z | ssRNA(+) | 1353 | GenBank | spike protein | Saudi Arabia | Homo sapiens | 07/03/2015 |
| ALJ54466.1 | Lu,X., Assiri,A.M., Erdman,D.D. | 2016-01-07T00:00:00Z | ssRNA(+) | 1353 | GenBank | spike protein | Saudi Arabia | Homo sapiens | 12/03/2015 |
| ALJ54467.1 | Lu,X., Assiri,A.M., Erdman,D.D. | 2016-01-07T00:00:00Z | ssRNA(+) | 1353 | GenBank | spike protein | Saudi Arabia | Homo sapiens | 12/05/2015 |
| ALJ54468.1 | Lu,X., Assiri,A.M., Erdman,D.D. | 2016-01-07T00:00:00Z | ssRNA(+) | 1353 | GenBank | spike protein | Saudi Arabia | Homo sapiens | 10/05/2015 |
| ALJ54469.1 | Lu,X., Assiri,A.M., Erdman,D.D. | 2016-01-07T00:00:00Z | ssRNA(+) | 1353 | GenBank | spike protein | Saudi Arabia | Homo sapiens | 13/05/2015 |
| ALJ54470.1 | Lu,X., Assiri,A.M., Erdman,D.D. | 2016-01-07T00:00:00Z | ssRNA(+) | 1353 | GenBank | spike protein | Saudi Arabia | Homo sapiens | 10/05/2015 |
| ALJ54471.1 | Lu,X., Assiri,A.M., Erdman,D.D. | 2016-01-07T00:00:00Z | ssRNA(+) | 1353 | GenBank | spike protein | Saudi Arabia | Homo sapiens | 08/05/2015 |
| ALJ54472.1 | Lu,X., Assiri,A.M., Erdman,D.D. | 2016-01-07T00:00:00Z | ssRNA(+) | 1353 | GenBank | spike protein | Saudi Arabia | Homo sapiens | 01/05/2015 |
| ALJ54473.1 | Lu,X., Assiri,A.M., Erdman,D.D. | 2016-01-07T00:00:00Z | ssRNA(+) | 1353 | GenBank | spike protein | Saudi Arabia | Homo sapiens | 05/03/2015 |
| ALJ54474.1 | Lu,X., Assiri,A.M., Erdman,D.D. | 2016-01-07T00:00:00Z | ssRNA(+) | 1353 | GenBank | spike protein | Saudi Arabia | Homo sapiens | 09/03/2015 |
| ALJ54475.1 | Lu,X., Assiri,A.M., Erdman,D.D. | 2016-01-07T00:00:00Z | ssRNA(+) | 1353 | GenBank | spike protein | Saudi Arabia | Homo sapiens | 20/03/2015 |
| ALJ54476.1 | Lu,X., Assiri,A.M., Erdman,D.D. | 2016-01-07T00:00:00Z | ssRNA(+) | 1353 | GenBank | spike protein | Saudi Arabia | Homo sapiens | 21/03/2015 |
| ALJ54477.1 | Lu,X., Assiri,A.M., Erdman,D.D. | 2016-01-07T00:00:00Z | ssRNA(+) | 1353 | GenBank | spike protein | Saudi Arabia | Homo sapiens | 29/03/2015 |
| ALJ54478.1 | Lu,X., Assiri,A.M., Erdman,D.D. | 2016-01-07T00:00:00Z | ssRNA(+) | 1353 | GenBank | spike protein | Saudi Arabia | Homo sapiens | 29/03/2015 |
| ALJ54479.1 | Lu,X., Assiri,A.M., Erdman,D.D. | 2016-01-07T00:00:00Z | ssRNA(+) | 1353 | GenBank | spike protein | Saudi Arabia | Homo sapiens | 01/04/2015 |
| ALJ54480.1 | Lu,X., Assiri,A.M., Erdman,D.D. | 2016-01-07T00:00:00Z | ssRNA(+) | 1353 | GenBank | spike protein | Saudi Arabia | Homo sapiens | 10/02/2015 |
| ALJ54481.1 | Lu,X., Assiri,A.M., Erdman,D.D. | 2016-01-07T00:00:00Z | ssRNA(+) | 1353 | GenBank | spike protein | Saudi Arabia | Homo sapiens | 13/02/2015 |
| ALJ54482.1 | Lu,X., Assiri,A.M., Erdman,D.D. | 2016-01-07T00:00:00Z | ssRNA(+) | 1353 | GenBank | spike protein | Saudi Arabia | Homo sapiens | 13/02/2015 |
| ALJ54483.1 | Lu,X., Assiri,A.M., Erdman,D.D. | 2016-01-07T00:00:00Z | ssRNA(+) | 1353 | GenBank | spike protein | Saudi Arabia | Homo sapiens | 13/02/2015 |
| ALJ54484.1 | Lu,X., Assiri,A.M., Erdman,D.D. | 2016-01-07T00:00:00Z | ssRNA(+) | 1353 | GenBank | spike protein | Saudi Arabia | Homo sapiens | 14/02/2015 |
| ALJ54485.1 | Lu,X., Assiri,A.M., Erdman,D.D. | 2016-01-07T00:00:00Z | ssRNA(+) | 1353 | GenBank | spike protein | Saudi Arabia | Homo sapiens | 25/02/2015 |
| ALJ54486.1 | Lu,X., Assiri,A.M., Erdman,D.D. | 2016-01-07T00:00:00Z | ssRNA(+) | 1353 | GenBank | spike protein | Saudi Arabia | Homo sapiens | 28/02/2015 |
| ALJ54487.1 | Lu,X., Assiri,A.M., Erdman,D.D. | 2016-01-07T00:00:00Z | ssRNA(+) | 1353 | GenBank | spike protein | Saudi Arabia | Homo sapiens | 04/03/2015 |
| ALJ54488.1 | Lu,X., Assiri,A.M., Erdman,D.D. | 2016-01-07T00:00:00Z | ssRNA(+) | 1353 | GenBank | spike protein | Saudi Arabia | Homo sapiens | 04/03/2015 |
| ALJ54489.1 | Lu,X., Assiri,A.M., Erdman,D.D. | 2016-01-07T00:00:00Z | ssRNA(+) | 1353 | GenBank | spike protein | Saudi Arabia | Homo sapiens | 08/03/2015 |
| ALJ54490.1 | Lu,X., Assiri,A.M., Erdman,D.D. | 2016-01-07T00:00:00Z | ssRNA(+) | 1353 | GenBank | spike protein | Saudi Arabia | Homo sapiens | 24/03/2015 |
| ALJ54491.1 | Lu,X., Assiri,A.M., Erdman,D.D. | 2016-01-07T00:00:00Z | ssRNA(+) | 1353 | GenBank | spike protein | Saudi Arabia | Homo sapiens | 25/03/2015 |
| ALJ54492.1 | Lu,X., Assiri,A.M., Erdman,D.D. | 2016-01-07T00:00:00Z | ssRNA(+) | 1353 | GenBank | spike protein | Saudi Arabia | Homo sapiens | 30/03/2015 |
| ALJ54493.1 | Lu,X., Assiri,A.M., Erdman,D.D. | 2016-01-07T00:00:00Z | ssRNA(+) | 1353 | GenBank | spike protein | Saudi Arabia | Homo sapiens | 04/04/2015 |
| ALJ54494.1 | Lu,X., Assiri,A.M., Erdman,D.D. | 2016-01-07T00:00:00Z | ssRNA(+) | 1353 | GenBank | spike protein | Saudi Arabia | Homo sapiens | 04/04/2015 |
| ALJ54495.1 | Lu,X., Assiri,A.M., Erdman,D.D. | 2016-01-07T00:00:00Z | ssRNA(+) | 1353 | GenBank | spike protein | Saudi Arabia | Homo sapiens | 13/04/2015 |
| ALJ54496.1 | Lu,X., Assiri,A.M., Erdman,D.D. | 2016-01-07T00:00:00Z | ssRNA(+) | 1353 | GenBank | spike protein | Saudi Arabia | Homo sapiens | 16/04/2015 |
| ALJ54497.1 | Lu,X., Assiri,A.M., Erdman,D.D. | 2016-01-07T00:00:00Z | ssRNA(+) | 1353 | GenBank | spike protein | Saudi Arabia | Homo sapiens | 09/05/2015 |
| ALJ54498.1 | Lu,X., Assiri,A.M., Erdman,D.D. | 2016-01-07T00:00:00Z | ssRNA(+) | 1353 | GenBank | spike protein | Saudi Arabia | Homo sapiens | 09/05/2015 |
| ALJ54499.1 | Lu,X., Assiri,A.M., Erdman,D.D. | 2016-01-07T00:00:00Z | ssRNA(+) | 1353 | GenBank | spike protein | Saudi Arabia | Homo sapiens | 09/05/2015 |
| ALJ54500.1 | Lu,X., Assiri,A.M., Erdman,D.D. | 2016-01-07T00:00:00Z | ssRNA(+) | 1353 | GenBank | spike protein | Saudi Arabia | Homo sapiens | 10/05/2015 |
| ALJ54501.1 | Lu,X., Assiri,A.M., Erdman,D.D. | 2016-01-07T00:00:00Z | ssRNA(+) | 1353 | GenBank | spike protein | Saudi Arabia | Homo sapiens | 21/03/2015 |
| ALJ54502.1 | Lu,X., Assiri,A.M., Erdman,D.D. | 2016-01-07T00:00:00Z | ssRNA(+) | 1353 | GenBank | spike protein | Saudi Arabia | Homo sapiens | 12/05/2015 |
| ALJ54503.1 | Lu,X., Assiri,A.M., Erdman,D.D. | 2016-01-07T00:00:00Z | ssRNA(+) | 1353 | GenBank | spike protein | Saudi Arabia | Homo sapiens | 17/05/2015 |
| ALJ54504.1 | Lu,X., Assiri,A.M., Erdman,D.D. | 2016-01-07T00:00:00Z | ssRNA(+) | 1353 | GenBank | spike protein | Saudi Arabia | Homo sapiens | 20/05/2015 |
| ALJ54505.1 | Lu,X., Assiri,A.M., Erdman,D.D. | 2016-01-07T00:00:00Z | ssRNA(+) | 1353 | GenBank | spike protein | Saudi Arabia | Homo sapiens | 22/05/2015 |
| ALJ54506.1 | Lu,X., Assiri,A.M., Erdman,D.D. | 2016-01-07T00:00:00Z | ssRNA(+) | 1353 | GenBank | spike protein | Saudi Arabia | Homo sapiens | 23/05/2015 |
| ALJ54507.1 | Lu,X., Assiri,A.M., Erdman,D.D. | 2016-01-07T00:00:00Z | ssRNA(+) | 1353 | GenBank | spike protein | Saudi Arabia | Homo sapiens | 29/05/2015 |
| ALJ54508.1 | Lu,X., Assiri,A.M., Erdman,D.D. | 2016-01-07T00:00:00Z | ssRNA(+) | 1353 | GenBank | spike protein | Saudi Arabia | Homo sapiens | 29/05/2015 |
| ALJ54509.1 | Lu,X., Assiri,A.M., Erdman,D.D. | 2016-01-07T00:00:00Z | ssRNA(+) | 1353 | GenBank | spike protein | Saudi Arabia | Homo sapiens | 28/05/2015 |
| ALJ54510.1 | Lu,X., Assiri,A.M., Erdman,D.D. | 2016-01-07T00:00:00Z | ssRNA(+) | 1353 | GenBank | spike protein | Saudi Arabia | Homo sapiens | 28/05/2015 |
| ALJ54511.1 | Lu,X., Assiri,A.M., Erdman,D.D. | 2016-01-07T00:00:00Z | ssRNA(+) | 1353 | GenBank | spike protein | Saudi Arabia | Homo sapiens | 27/05/2015 |
| ALJ54512.1 | Lu,X., Assiri,A.M., Erdman,D.D. | 2016-01-07T00:00:00Z | ssRNA(+) | 1353 | GenBank | spike protein | Saudi Arabia | Homo sapiens | 27/05/2015 |
| ALJ54513.1 | Lu,X., Assiri,A.M., Erdman,D.D. | 2016-01-07T00:00:00Z | ssRNA(+) | 1353 | GenBank | spike protein | Saudi Arabia | Homo sapiens | 25/04/2015 |
| ALJ54514.1 | Lu,X., Assiri,A.M., Erdman,D.D. | 2016-01-07T00:00:00Z | ssRNA(+) | 1353 | GenBank | spike protein | Saudi Arabia | Homo sapiens | 29/05/2015 |
| ALJ54515.1 | Lu,X., Assiri,A.M., Erdman,D.D. | 2016-01-07T00:00:00Z | ssRNA(+) | 1353 | GenBank | spike protein | Saudi Arabia | Homo sapiens | 01/06/2015 |
| ALJ54516.1 | Lu,X., Assiri,A.M., Erdman,D.D. | 2016-01-07T00:00:00Z | ssRNA(+) | 1353 | GenBank | spike protein | Saudi Arabia | Homo sapiens | 02/06/2015 |
| ALJ54517.1 | Lu,X., Assiri,A.M., Erdman,D.D. | 2016-01-07T00:00:00Z | ssRNA(+) | 1353 | GenBank | spike protein | Saudi Arabia | Homo sapiens | 03/06/2015 |
| ALJ54518.1 | Lu,X., Assiri,A.M., Erdman,D.D. | 2016-01-07T00:00:00Z | ssRNA(+) | 1353 | GenBank | spike protein | Saudi Arabia | Homo sapiens | 04/06/2015 |
| ALJ54519.1 | Lu,X., Assiri,A.M., Erdman,D.D. | 2016-01-07T00:00:00Z | ssRNA(+) | 1353 | GenBank | spike protein | Saudi Arabia | Homo sapiens | 07/06/2015 |
| ALJ54520.1 | Lu,X., Assiri,A.M., Erdman,D.D. | 2016-01-07T00:00:00Z | ssRNA(+) | 1353 | GenBank | spike protein | Saudi Arabia | Homo sapiens | 13/06/2015 |
| ALJ54521.1 | Lu,X., Assiri,A.M., Erdman,D.D. | 2016-01-07T00:00:00Z | ssRNA(+) | 1353 | GenBank | spike protein | Saudi Arabia | Homo sapiens | 12/05/2015 |
| AKQ21083.1 | Al Hammadi,Z.M., Chu,D.K., Eltahir,Y.M., Al Hosani,F., Al Mulla,M., Tarnini,W., Hall,A.J., Perera,R.A., Abdelkhalek,M.M., Peiris,J.S., Al Muhairi,S.S., Poon,L.L., Chu,D.K.W., Peiris,M.J.S., Poon,L.L.M. | 2015-12-17T00:00:00Z | ssRNA(+) | 144 | GenBank | spike protein | United Arab Emirates | Homo sapiens | 09/05/2015 |
| AKQ21085.1 | Al Hammadi,Z.M., Chu,D.K., Eltahir,Y.M., Al Hosani,F., Al Mulla,M., Tarnini,W., Hall,A.J., Perera,R.A., Abdelkhalek,M.M., Peiris,J.S., Al Muhairi,S.S., Poon,L.L., Chu,D.K.W., Peiris,M.J.S., Poon,L.L.M. | 2015-12-17T00:00:00Z | ssRNA(+) | 144 | GenBank | spike protein | United Arab Emirates | Homo sapiens | 09/05/2015 |
| AKN24749.1 | Fagbo,S.F., Skakni,L., Chu,D.K., Garbati,M.A., Joseph,M., Peiris,M., Hakawi,A.M., Chu,D.K.W., Peiris,M.J.S. | 2015-11-17T00:00:00Z | ssRNA(+) | 1353 | GenBank | spike protein | Saudi Arabia | Homo sapiens | 12/05/2014 |
| AKN24758.1 | Fagbo,S.F., Skakni,L., Chu,D.K., Garbati,M.A., Joseph,M., Peiris,M., Hakawi,A.M., Chu,D.K.W., Peiris,M.J.S. | 2015-11-17T00:00:00Z | ssRNA(+) | 1353 | GenBank | spike protein | Saudi Arabia | Homo sapiens | 09/05/2014 |
| AKN24767.1 | Fagbo,S.F., Skakni,L., Chu,D.K., Garbati,M.A., Joseph,M., Peiris,M., Hakawi,A.M., Chu,D.K.W., Peiris,M.J.S. | 2015-11-17T00:00:00Z | ssRNA(+) | 1353 | GenBank | spike protein | Saudi Arabia | Homo sapiens | 07/05/2014 |
| AKN24776.1 | Fagbo,S.F., Skakni,L., Chu,D.K., Garbati,M.A., Joseph,M., Peiris,M., Hakawi,A.M., Chu,D.K.W., Peiris,M.J.S. | 2015-11-17T00:00:00Z | ssRNA(+) | 1353 | GenBank | spike protein | Saudi Arabia | Homo sapiens | 12/05/2014 |
| AKN24785.1 | Fagbo,S.F., Skakni,L., Chu,D.K., Garbati,M.A., Joseph,M., Peiris,M., Hakawi,A.M., Chu,D.K.W., Peiris,M.J.S. | 2015-11-17T00:00:00Z | ssRNA(+) | 1353 | GenBank | spike protein | Saudi Arabia | Homo sapiens | 18/05/2014 |
| AKN24794.1 | Fagbo,S.F., Skakni,L., Chu,D.K., Garbati,M.A., Joseph,M., Peiris,M., Hakawi,A.M., Chu,D.K.W., Peiris,M.J.S. | 2015-11-17T00:00:00Z | ssRNA(+) | 1353 | GenBank | spike protein | Saudi Arabia | Homo sapiens | 11/05/2014 |
| AKN24803.1 | Fagbo,S.F., Skakni,L., Chu,D.K., Garbati,M.A., Joseph,M., Peiris,M., Hakawi,A.M., Chu,D.K.W., Peiris,M.J.S. | 2015-11-17T00:00:00Z | ssRNA(+) | 1353 | GenBank | spike protein | Saudi Arabia | Homo sapiens | 01/05/2014 |
| AKN24812.1 | Fagbo,S.F., Skakni,L., Chu,D.K., Garbati,M.A., Joseph,M., Peiris,M., Hakawi,A.M., Chu,D.K.W., Peiris,M.J.S. | 2015-11-17T00:00:00Z | ssRNA(+) | 1353 | GenBank | spike protein | Saudi Arabia | Homo sapiens | 30/04/2014 |
| AKN24821.1 | Fagbo,S.F., Skakni,L., Chu,D.K., Garbati,M.A., Joseph,M., Peiris,M., Hakawi,A.M., Chu,D.K.W., Peiris,M.J.S. | 2015-11-17T00:00:00Z | ssRNA(+) | 1353 | GenBank | spike protein | Saudi Arabia | Homo sapiens | 28/04/2014 |
| AKN24830.1 | Fagbo,S.F., Skakni,L., Chu,D.K., Garbati,M.A., Joseph,M., Peiris,M., Hakawi,A.M., Chu,D.K.W., Peiris,M.J.S. | 2015-11-17T00:00:00Z | ssRNA(+) | 1353 | GenBank | spike protein | Saudi Arabia | Homo sapiens | 03/05/2014 |
| ALJ76277.1 | Lu,X., Saeed,A.A.B., Erdman,D.D. | 2015-10-21T00:00:00Z | ssRNA(+) | 1353 | GenBank | spike protein | Saudi Arabia | Homo sapiens | 28/11/2014 |
| ALJ76278.1 | Lu,X., Saeed,A.A.B., Erdman,D.D. | 2015-10-21T00:00:00Z | ssRNA(+) | 1353 | GenBank | spike protein | Saudi Arabia | Homo sapiens | 04/11/2014 |
| ALJ76279.1 | Lu,X., Saeed,A.A.B., Erdman,D.D. | 2015-10-21T00:00:00Z | ssRNA(+) | 1353 | GenBank | spike protein | Saudi Arabia | Homo sapiens | 11/11/2014 |
| ALJ76280.1 | Lu,X., Saeed,A.A.B., Erdman,D.D. | 2015-10-21T00:00:00Z | ssRNA(+) | 1353 | GenBank | spike protein | Saudi Arabia | Homo sapiens | 27/10/2014 |
| ALJ76281.1 | Lu,X., Saeed,A.A.B., Erdman,D.D. | 2015-10-21T00:00:00Z | ssRNA(+) | 1353 | GenBank | spike protein | Saudi Arabia | Homo sapiens | 10/09/2014 |
| ALJ76282.1 | Lu,X., Saeed,A.A.B., Erdman,D.D. | 2015-10-21T00:00:00Z | ssRNA(+) | 1353 | GenBank | spike protein | Saudi Arabia | Homo sapiens | 24/10/2014 |
| ALJ76283.1 | Lu,X., Saeed,A.A.B., Erdman,D.D. | 2015-10-21T00:00:00Z | ssRNA(+) | 1353 | GenBank | spike protein | Saudi Arabia | Homo sapiens | 27/10/2014 |
| ALJ76284.1 | Lu,X., Saeed,A.A.B., Erdman,D.D. | 2015-10-21T00:00:00Z | ssRNA(+) | 1353 | GenBank | spike protein | Saudi Arabia | Homo sapiens | 27/10/2014 |
| ALJ76285.1 | Lu,X., Saeed,A.A.B., Erdman,D.D. | 2015-10-21T00:00:00Z | ssRNA(+) | 1353 | GenBank | spike protein | Saudi Arabia | Homo sapiens | 12/11/2014 |
| ALJ76286.1 | Lu,X., Saeed,A.A.B., Erdman,D.D. | 2015-10-21T00:00:00Z | ssRNA(+) | 1353 | GenBank | spike protein | Saudi Arabia | Homo sapiens | 22/11/2014 |
| ALG00414.1 | Kim,Y.-S., Cho,N.-H. | 2015-10-03T00:00:00Z | ssRNA(+) | 205 | GenBank | spike protein | South Korea | Homo sapiens | 11/06/2015 |
| ALG00415.1 | Kim,Y.-S., Cho,N.-H. | 2015-10-03T00:00:00Z | ssRNA(+) | 205 | GenBank | spike protein | South Korea | Homo sapiens | 11/06/2015 |
| ALG00416.1 | Kim,Y.-S., Cho,N.-H. | 2015-10-03T00:00:00Z | ssRNA(+) | 205 | GenBank | spike protein | South Korea | Homo sapiens | 08/06/2015 |
| ALG00417.1 | Kim,Y.-S., Cho,N.-H. | 2015-10-03T00:00:00Z | ssRNA(+) | 205 | GenBank | spike protein | South Korea | Homo sapiens | 08/06/2015 |
| ALG00418.1 | Kim,Y.-S., Cho,N.-H. | 2015-10-03T00:00:00Z | ssRNA(+) | 205 | GenBank | spike protein | South Korea | Homo sapiens | 08/06/2015 |
| ALG00419.1 | Kim,Y.-S., Cho,N.-H. | 2015-10-03T00:00:00Z | ssRNA(+) | 205 | GenBank | spike protein | South Korea | Homo sapiens | 10/06/2015 |
| ALG00420.1 | Kim,Y.-S., Cho,N.-H. | 2015-10-03T00:00:00Z | ssRNA(+) | 205 | GenBank | spike protein | South Korea | Homo sapiens | 09/06/2015 |
| ALG00421.1 | Kim,Y.-S., Cho,N.-H. | 2015-10-03T00:00:00Z | ssRNA(+) | 205 | GenBank | spike protein | South Korea | Homo sapiens | 10/06/2015 |
| ALG00422.1 | Kim,Y.-S., Cho,N.-H. | 2015-10-03T00:00:00Z | ssRNA(+) | 205 | GenBank | spike protein | South Korea | Homo sapiens | 10/06/2015 |
| ALG00423.1 | Kim,Y.-S., Cho,N.-H. | 2015-10-03T00:00:00Z | ssRNA(+) | 205 | GenBank | spike protein | South Korea | Homo sapiens | 11/06/2015 |
| ALG00424.1 | Kim,Y.-S., Cho,N.-H. | 2015-10-03T00:00:00Z | ssRNA(+) | 205 | GenBank | spike protein | South Korea | Homo sapiens | 10/06/2015 |
| ALG00425.1 | Kim,Y.-S., Cho,N.-H. | 2015-10-03T00:00:00Z | ssRNA(+) | 205 | GenBank | spike protein | South Korea | Homo sapiens | 15/06/2015 |
| ALG00426.1 | Kim,Y.-S., Cho,N.-H. | 2015-10-03T00:00:00Z | ssRNA(+) | 205 | GenBank | spike protein | South Korea | Homo sapiens | 2015-06 |
| AKL59401.1 | Kim,Y.J., Cho,Y.J., Kim,D.W., Yang,J.S., Kim,H., Park,S., Han,Y.W., Yun,M.R., Lee,H.S., Kim,A.R., Heo,D.R., Kim,J.A., Kim,S.J., Jung,H.D., Kim,N., Yoon,S.H., Nam,J.G., Kang,H.J., Cheong,H.M., Lee,J.S., Chun,J., Kim,S.S., Nam,J.-G., Kim,Y.-J., Cheong,H.-M., Yang,J.-S., Cho,Y.-J., Yoon,S.-H., Yun,M.-R., Kim,D.-W. | 2015-06-09T00:00:00Z | ssRNA(+) | 1353 | GenBank | spike protein | South Korea | Homo sapiens | 20/05/2015 |
| AID55066.1 | Drosten,C., Muth,D., Corman,V.M., Hussain,R., Al Masri,M., HajOmar,W., Landt,O., Assiri,A., Eckerle,I., Al Shangiti,A., Al-Tawfiq,J.A., Albarrak,A., Zumla,A., Rambaut,A., Memish,Z.A., Memish,Z. | 2014-11-12T00:00:00Z | ssRNA(+) | 1353 | GenBank | spike protein | Saudi Arabia | Homo sapiens | 2014 |
| AID55067.1 | Drosten,C., Muth,D., Corman,V.M., Hussain,R., Al Masri,M., HajOmar,W., Landt,O., Assiri,A., Eckerle,I., Al Shangiti,A., Al-Tawfiq,J.A., Albarrak,A., Zumla,A., Rambaut,A., Memish,Z.A., Memish,Z. | 2014-11-12T00:00:00Z | ssRNA(+) | 1353 | GenBank | spike protein | Saudi Arabia | Homo sapiens | 2014 |
| AID55068.1 | Drosten,C., Muth,D., Corman,V.M., Hussain,R., Al Masri,M., HajOmar,W., Landt,O., Assiri,A., Eckerle,I., Al Shangiti,A., Al-Tawfiq,J.A., Albarrak,A., Zumla,A., Rambaut,A., Memish,Z.A., Memish,Z. | 2014-11-12T00:00:00Z | ssRNA(+) | 1353 | GenBank | spike protein | Saudi Arabia | Homo sapiens | 07/04/2014 |
| AID55069.1 | Drosten,C., Muth,D., Corman,V.M., Hussain,R., Al Masri,M., HajOmar,W., Landt,O., Assiri,A., Eckerle,I., Al Shangiti,A., Al-Tawfiq,J.A., Albarrak,A., Zumla,A., Rambaut,A., Memish,Z.A., Memish,Z. | 2014-11-12T00:00:00Z | ssRNA(+) | 1353 | GenBank | spike protein | Saudi Arabia | Homo sapiens | 12/04/2014 |
| AID55070.1 | Drosten,C., Muth,D., Corman,V.M., Hussain,R., Al Masri,M., HajOmar,W., Landt,O., Assiri,A., Eckerle,I., Al Shangiti,A., Al-Tawfiq,J.A., Albarrak,A., Zumla,A., Rambaut,A., Memish,Z.A., Memish,Z. | 2014-11-12T00:00:00Z | ssRNA(+) | 1353 | GenBank | spike protein | Saudi Arabia | Homo sapiens | 14/04/2014 |
| AID55071.1 | Drosten,C., Muth,D., Corman,V.M., Hussain,R., Al Masri,M., HajOmar,W., Landt,O., Assiri,A., Eckerle,I., Al Shangiti,A., Al-Tawfiq,J.A., Albarrak,A., Zumla,A., Rambaut,A., Memish,Z.A., Memish,Z. | 2014-11-12T00:00:00Z | ssRNA(+) | 1353 | GenBank | spike protein | Saudi Arabia | Homo sapiens | 21/04/2014 |
| AID55072.1 | Drosten,C., Muth,D., Corman,V.M., Hussain,R., Al Masri,M., HajOmar,W., Landt,O., Assiri,A., Eckerle,I., Al Shangiti,A., Al-Tawfiq,J.A., Albarrak,A., Zumla,A., Rambaut,A., Memish,Z.A., Memish,Z. | 2014-11-12T00:00:00Z | ssRNA(+) | 1353 | GenBank | spike protein | Saudi Arabia | Homo sapiens | 15/04/2014 |
| AID55073.1 | Drosten,C., Muth,D., Corman,V.M., Hussain,R., Al Masri,M., HajOmar,W., Landt,O., Assiri,A., Eckerle,I., Al Shangiti,A., Al-Tawfiq,J.A., Albarrak,A., Zumla,A., Rambaut,A., Memish,Z.A., Memish,Z. | 2014-11-12T00:00:00Z | ssRNA(+) | 1353 | GenBank | spike protein | Saudi Arabia | Homo sapiens | 22/04/2014 |
| AID55074.1 | Drosten,C., Muth,D., Corman,V.M., Hussain,R., Al Masri,M., HajOmar,W., Landt,O., Assiri,A., Eckerle,I., Al Shangiti,A., Al-Tawfiq,J.A., Albarrak,A., Zumla,A., Rambaut,A., Memish,Z.A., Memish,Z. | 2014-11-12T00:00:00Z | ssRNA(+) | 1353 | GenBank | spike protein | Saudi Arabia | Homo sapiens | 2014 |
| AID55075.1 | Drosten,C., Muth,D., Corman,V.M., Hussain,R., Al Masri,M., HajOmar,W., Landt,O., Assiri,A., Eckerle,I., Al Shangiti,A., Al-Tawfiq,J.A., Albarrak,A., Zumla,A., Rambaut,A., Memish,Z.A., Memish,Z. | 2014-11-12T00:00:00Z | ssRNA(+) | 1353 | GenBank | spike protein | Saudi Arabia | Homo sapiens | 2014 |
| AID55076.1 | Drosten,C., Muth,D., Corman,V.M., Hussain,R., Al Masri,M., HajOmar,W., Landt,O., Assiri,A., Eckerle,I., Al Shangiti,A., Al-Tawfiq,J.A., Albarrak,A., Zumla,A., Rambaut,A., Memish,Z.A., Memish,Z. | 2014-11-12T00:00:00Z | ssRNA(+) | 1353 | GenBank | spike protein | Saudi Arabia | Homo sapiens | 2014 |
| AID55077.1 | Drosten,C., Muth,D., Corman,V.M., Hussain,R., Al Masri,M., HajOmar,W., Landt,O., Assiri,A., Eckerle,I., Al Shangiti,A., Al-Tawfiq,J.A., Albarrak,A., Zumla,A., Rambaut,A., Memish,Z.A., Memish,Z. | 2014-11-12T00:00:00Z | ssRNA(+) | 1353 | GenBank | spike protein | Saudi Arabia | Homo sapiens | 2014 |
| AID55078.1 | Drosten,C., Muth,D., Corman,V.M., Hussain,R., Al Masri,M., HajOmar,W., Landt,O., Assiri,A., Eckerle,I., Al Shangiti,A., Al-Tawfiq,J.A., Albarrak,A., Zumla,A., Rambaut,A., Memish,Z.A., Memish,Z. | 2014-11-12T00:00:00Z | ssRNA(+) | 1353 | GenBank | spike protein | Saudi Arabia | Homo sapiens | 2014 |
| AID55079.1 | Drosten,C., Muth,D., Corman,V.M., Hussain,R., Al Masri,M., HajOmar,W., Landt,O., Assiri,A., Eckerle,I., Al Shangiti,A., Al-Tawfiq,J.A., Albarrak,A., Zumla,A., Rambaut,A., Memish,Z.A., Memish,Z. | 2014-11-12T00:00:00Z | ssRNA(+) | 1353 | GenBank | spike protein | Saudi Arabia | Homo sapiens | 2014 |
| AID55080.1 | Drosten,C., Muth,D., Corman,V.M., Hussain,R., Al Masri,M., HajOmar,W., Landt,O., Assiri,A., Eckerle,I., Al Shangiti,A., Al-Tawfiq,J.A., Albarrak,A., Zumla,A., Rambaut,A., Memish,Z.A., Memish,Z. | 2014-11-12T00:00:00Z | ssRNA(+) | 1353 | GenBank | spike protein | Saudi Arabia | Homo sapiens | 2014 |
| AID55081.1 | Drosten,C., Muth,D., Corman,V.M., Hussain,R., Al Masri,M., HajOmar,W., Landt,O., Assiri,A., Eckerle,I., Al Shangiti,A., Al-Tawfiq,J.A., Albarrak,A., Zumla,A., Rambaut,A., Memish,Z.A., Memish,Z. | 2014-11-12T00:00:00Z | ssRNA(+) | 1353 | GenBank | spike protein | Saudi Arabia | Homo sapiens | 2014 |
| AID55082.1 | Drosten,C., Muth,D., Corman,V.M., Hussain,R., Al Masri,M., HajOmar,W., Landt,O., Assiri,A., Eckerle,I., Al Shangiti,A., Al-Tawfiq,J.A., Albarrak,A., Zumla,A., Rambaut,A., Memish,Z.A., Memish,Z. | 2014-11-12T00:00:00Z | ssRNA(+) | 1353 | GenBank | spike protein | Saudi Arabia | Homo sapiens | 2014 |
| AID55083.1 | Drosten,C., Muth,D., Corman,V.M., Hussain,R., Al Masri,M., HajOmar,W., Landt,O., Assiri,A., Eckerle,I., Al Shangiti,A., Al-Tawfiq,J.A., Albarrak,A., Zumla,A., Rambaut,A., Memish,Z.A., Memish,Z. | 2014-11-12T00:00:00Z | ssRNA(+) | 1353 | GenBank | spike protein | Saudi Arabia | Homo sapiens | 2014 |
| AID55084.1 | Drosten,C., Muth,D., Corman,V.M., Hussain,R., Al Masri,M., HajOmar,W., Landt,O., Assiri,A., Eckerle,I., Al Shangiti,A., Al-Tawfiq,J.A., Albarrak,A., Zumla,A., Rambaut,A., Memish,Z.A., Memish,Z. | 2014-11-12T00:00:00Z | ssRNA(+) | 1353 | GenBank | spike protein | Saudi Arabia | Homo sapiens | 2014 |
| AID55085.1 | Drosten,C., Muth,D., Corman,V.M., Hussain,R., Al Masri,M., HajOmar,W., Landt,O., Assiri,A., Eckerle,I., Al Shangiti,A., Al-Tawfiq,J.A., Albarrak,A., Zumla,A., Rambaut,A., Memish,Z.A., Memish,Z. | 2014-11-12T00:00:00Z | ssRNA(+) | 1353 | GenBank | spike protein | Saudi Arabia | Homo sapiens | 2014 |
| AID55086.1 | Drosten,C., Muth,D., Corman,V.M., Hussain,R., Al Masri,M., HajOmar,W., Landt,O., Assiri,A., Eckerle,I., Al Shangiti,A., Al-Tawfiq,J.A., Albarrak,A., Zumla,A., Rambaut,A., Memish,Z.A., Memish,Z. | 2014-11-12T00:00:00Z | ssRNA(+) | 1353 | GenBank | spike protein | Saudi Arabia | Homo sapiens | 2014 |
| AID55087.1 | Drosten,C., Muth,D., Corman,V.M., Hussain,R., Al Masri,M., HajOmar,W., Landt,O., Assiri,A., Eckerle,I., Al Shangiti,A., Al-Tawfiq,J.A., Albarrak,A., Zumla,A., Rambaut,A., Memish,Z.A., Memish,Z. | 2014-11-12T00:00:00Z | ssRNA(+) | 1353 | GenBank | spike protein | Saudi Arabia | Homo sapiens | 2014 |
| AID55088.1 | Drosten,C., Muth,D., Corman,V.M., Hussain,R., Al Masri,M., HajOmar,W., Landt,O., Assiri,A., Eckerle,I., Al Shangiti,A., Al-Tawfiq,J.A., Albarrak,A., Zumla,A., Rambaut,A., Memish,Z.A., Memish,Z. | 2014-11-12T00:00:00Z | ssRNA(+) | 1353 | GenBank | spike protein | Saudi Arabia | Homo sapiens | 2014 |
| AID55089.1 | Drosten,C., Muth,D., Corman,V.M., Hussain,R., Al Masri,M., HajOmar,W., Landt,O., Assiri,A., Eckerle,I., Al Shangiti,A., Al-Tawfiq,J.A., Albarrak,A., Zumla,A., Rambaut,A., Memish,Z.A., Memish,Z. | 2014-11-12T00:00:00Z | ssRNA(+) | 1353 | GenBank | spike protein | Saudi Arabia | Homo sapiens | 2014 |
| AID55090.1 | Drosten,C., Muth,D., Corman,V.M., Hussain,R., Al Masri,M., HajOmar,W., Landt,O., Assiri,A., Eckerle,I., Al Shangiti,A., Al-Tawfiq,J.A., Albarrak,A., Zumla,A., Rambaut,A., Memish,Z.A., Memish,Z. | 2014-11-12T00:00:00Z | ssRNA(+) | 1353 | GenBank | spike protein | Saudi Arabia | Homo sapiens | 2014 |
| AID55091.1 | Drosten,C., Muth,D., Corman,V.M., Hussain,R., Al Masri,M., HajOmar,W., Landt,O., Assiri,A., Eckerle,I., Al Shangiti,A., Al-Tawfiq,J.A., Albarrak,A., Zumla,A., Rambaut,A., Memish,Z.A., Memish,Z. | 2014-11-12T00:00:00Z | ssRNA(+) | 1353 | GenBank | spike protein | Saudi Arabia | Homo sapiens | 2014 |
| AID55092.1 | Drosten,C., Muth,D., Corman,V.M., Hussain,R., Al Masri,M., HajOmar,W., Landt,O., Assiri,A., Eckerle,I., Al Shangiti,A., Al-Tawfiq,J.A., Albarrak,A., Zumla,A., Rambaut,A., Memish,Z.A., Memish,Z. | 2014-11-12T00:00:00Z | ssRNA(+) | 1353 | GenBank | spike protein | Saudi Arabia | Homo sapiens | 2014 |
| AID55093.1 | Drosten,C., Muth,D., Corman,V.M., Hussain,R., Al Masri,M., HajOmar,W., Landt,O., Assiri,A., Eckerle,I., Al Shangiti,A., Al-Tawfiq,J.A., Albarrak,A., Zumla,A., Rambaut,A., Memish,Z.A., Memish,Z. | 2014-11-12T00:00:00Z | ssRNA(+) | 1353 | GenBank | spike protein | Saudi Arabia | Homo sapiens | 2014 |
| AID55094.1 | Drosten,C., Muth,D., Corman,V.M., Hussain,R., Al Masri,M., HajOmar,W., Landt,O., Assiri,A., Eckerle,I., Al Shangiti,A., Al-Tawfiq,J.A., Albarrak,A., Zumla,A., Rambaut,A., Memish,Z.A., Memish,Z. | 2014-11-12T00:00:00Z | ssRNA(+) | 1353 | GenBank | spike protein | Saudi Arabia | Homo sapiens | 2014 |
| AID55095.1 | Drosten,C., Muth,D., Corman,V.M., Hussain,R., Al Masri,M., HajOmar,W., Landt,O., Assiri,A., Eckerle,I., Al Shangiti,A., Al-Tawfiq,J.A., Albarrak,A., Zumla,A., Rambaut,A., Memish,Z.A., Memish,Z. | 2014-11-12T00:00:00Z | ssRNA(+) | 1353 | GenBank | spike protein | Saudi Arabia | Homo sapiens | 2014 |
| AID55096.1 | Drosten,C., Muth,D., Corman,V.M., Hussain,R., Al Masri,M., HajOmar,W., Landt,O., Assiri,A., Eckerle,I., Al Shangiti,A., Al-Tawfiq,J.A., Albarrak,A., Zumla,A., Rambaut,A., Memish,Z.A., Memish,Z. | 2014-11-12T00:00:00Z | ssRNA(+) | 1353 | GenBank | spike protein | Saudi Arabia | Homo sapiens | 2014 |
| AID55097.1 | Drosten,C., Muth,D., Corman,V.M., Hussain,R., Al Masri,M., HajOmar,W., Landt,O., Assiri,A., Eckerle,I., Al Shangiti,A., Al-Tawfiq,J.A., Albarrak,A., Zumla,A., Rambaut,A., Memish,Z.A., Memish,Z. | 2014-11-12T00:00:00Z | ssRNA(+) | 1353 | GenBank | spike protein | Saudi Arabia | Homo sapiens | 2014 |
| AID55098.1 | Drosten,C., Muth,D., Corman,V.M., Hussain,R., Al Masri,M., HajOmar,W., Landt,O., Assiri,A., Eckerle,I., Al Shangiti,A., Al-Tawfiq,J.A., Albarrak,A., Zumla,A., Rambaut,A., Memish,Z.A., Memish,Z. | 2014-11-12T00:00:00Z | ssRNA(+) | 1353 | GenBank | spike protein | Saudi Arabia | Homo sapiens | 2014 |
| AID55099.1 | Drosten,C., Muth,D., Corman,V.M., Hussain,R., Al Masri,M., HajOmar,W., Landt,O., Assiri,A., Eckerle,I., Al Shangiti,A., Al-Tawfiq,J.A., Albarrak,A., Zumla,A., Rambaut,A., Memish,Z.A., Memish,Z. | 2014-11-12T00:00:00Z | ssRNA(+) | 1353 | GenBank | spike protein | Saudi Arabia | Homo sapiens | 2014 |
| AID55100.1 | Drosten,C., Muth,D., Corman,V.M., Hussain,R., Al Masri,M., HajOmar,W., Landt,O., Assiri,A., Eckerle,I., Al Shangiti,A., Al-Tawfiq,J.A., Albarrak,A., Zumla,A., Rambaut,A., Memish,Z.A., Memish,Z. | 2014-11-12T00:00:00Z | ssRNA(+) | 1353 | GenBank | spike protein | Saudi Arabia | Homo sapiens | 2014 |
| AID55101.1 | Drosten,C., Muth,D., Corman,V.M., Hussain,R., Al Masri,M., HajOmar,W., Landt,O., Assiri,A., Eckerle,I., Al Shangiti,A., Al-Tawfiq,J.A., Albarrak,A., Zumla,A., Rambaut,A., Memish,Z.A., Memish,Z. | 2014-11-12T00:00:00Z | ssRNA(+) | 1353 | GenBank | spike protein | Saudi Arabia | Homo sapiens | 2014 |
| AID55102.1 | Drosten,C., Muth,D., Corman,V.M., Hussain,R., Al Masri,M., HajOmar,W., Landt,O., Assiri,A., Eckerle,I., Al Shangiti,A., Al-Tawfiq,J.A., Albarrak,A., Zumla,A., Rambaut,A., Memish,Z.A., Memish,Z. | 2014-11-12T00:00:00Z | ssRNA(+) | 1353 | GenBank | spike protein | Saudi Arabia | Homo sapiens | 2014 |
| AID55103.1 | Drosten,C., Muth,D., Corman,V.M., Hussain,R., Al Masri,M., HajOmar,W., Landt,O., Assiri,A., Eckerle,I., Al Shangiti,A., Al-Tawfiq,J.A., Albarrak,A., Zumla,A., Rambaut,A., Memish,Z.A., Memish,Z. | 2014-11-12T00:00:00Z | ssRNA(+) | 1353 | GenBank | spike protein | Saudi Arabia | Homo sapiens | 2014 |
| AIA22859.1 | Stalin Raj,V., Jonges,M., Koopmans,M.P.G., Haagmans,B.L. | 2014-05-22T00:00:00Z | ssRNA(+) | 185 | GenBank | spike protein | Netherlands | Homo sapiens | 14/05/2014 |
| AIA22860.1 | Stalin Raj,V., Jonges,M., Koopmans,M.P.G., Haagmans,B.L. | 2014-05-22T00:00:00Z | ssRNA(+) | 361 | GenBank | spike protein | Netherlands | Homo sapiens | 14/05/2014 |
| AIA22864.1 | Stalin Raj,V., Jonges,M., Koopmans,M.P.G., Haagmans,B.L. | 2014-05-22T00:00:00Z | ssRNA(+) | 291 | GenBank | spike protein | Netherlands | Homo sapiens | 13/05/2014 |
| AIA22865.1 | Stalin Raj,V., Jonges,M., Koopmans,M.P.G., Haagmans,B.L. | 2014-05-22T00:00:00Z | ssRNA(+) | 212 | GenBank | spike protein | Netherlands | Homo sapiens | 13/05/2014 |
| AHZ90568.1 | Abroug,F., Slim,A., Ouanes-Besbes,L., Kacem,M.A., Dachraoui,F., Ouanes,I., Lu,X., Tao,Y., Paden,C., Caidi,H., Miao,C., Al-Hajri,M.M., Zorraga,M., Ghaouar,W., BenSalah,A., Gerber,S.I., Erdman,D.D. | 2014-05-19T00:00:00Z | ssRNA(+) | 1353 | GenBank | spike protein | Tunisia | Homo sapiens | 08/05/2013 |
| AGO06003.1 | Drosten,C., Seilmaier,M., Corman,V.M., Hartmann,W., Scheible,G., Sack,S., Guggemos,W., Kallies,R., Muth,D., Junglen,S., Muller,M.A., Haas,W., Guberina,H., Rohnisch,T., Schmid-Wendtner,M., Aldabbagh,S., Dittmer,U., Gold,H., Graf,P., Bonin,F., Rambaut,A., Wendtner,C.M. | 2013-06-23T00:00:00Z | ssRNA(+) | 141 | GenBank | spike protein | Qatar | Homo sapiens | 2012 |
| AMQ49004.1 | Lu,X., Rowe,L.A., Frace,M., Stevens,J., Abedi,G.R., El Nile,O., Banassir,T., Al-Masri,M., Watson,J.T., Assiri,A., Erdman,D.D. | 2016-08-12T00:00:00Z | ssRNA(+) | 801 | GenBank | truncated S protein | Saudi Arabia | Homo sapiens | 04/11/2014 |

**Camel’s MERS-CoV Spike**

| Accession | Submitters | Release_Date | Molecule_type | Length | Protein | Country | Host | |
| --- | --- | --- | --- | --- | --- | --- | --- | --- |
| QFQ59587.1 | Rodon,J., Okba,N.M.A., Nigeer,T., van Dieren,B., Bosch,B.-J., Bensaid,A., Segales,J., Haagmans,B.L., Vergara-Alert,J. | 2019-10-23T00:00:00Z | ssRNA(+) | 1353 | S | Qatar | Lama glama | |
| AJG44058.1 | Wernery,U., Corman,V.M., Drosten,C. | 2015-02-09T00:00:00Z | ssRNA(+) | 1353 | S | United Arab Emirates | Camelus dromedarius | |
| AJG44069.1 | Wernery,U., Corman,V.M., Drosten,C. | 2015-02-09T00:00:00Z | ssRNA(+) | 1353 | S | United Arab Emirates | Camelus dromedarius | |
| AJG44080.1 | Wernery,U., Corman,V.M., Drosten,C. | 2015-02-09T00:00:00Z | ssRNA(+) | 1353 | S | United Arab Emirates | Camelus dromedarius | |
| AJG44091.1 | Wernery,U., Corman,V.M., Drosten,C. | 2015-02-09T00:00:00Z | ssRNA(+) | 1353 | S | United Arab Emirates | Camelus dromedarius | |
| AJG44102.1 | Wernery,U., Corman,V.M., Drosten,C. | 2015-02-09T00:00:00Z | ssRNA(+) | 1353 | S | United Arab Emirates | Camelus dromedarius | |
| AJG44113.1 | Wernery,U., Corman,V.M., Drosten,C. | 2015-02-09T00:00:00Z | ssRNA(+) | 1353 | S | United Arab Emirates | Camelus dromedarius | |
| AJG44124.1 | Wernery,U., Corman,V.M., Drosten,C. | 2015-02-09T00:00:00Z | ssRNA(+) | 1353 | S | United Arab Emirates | Camelus dromedarius | |
| AHE78097.1 | Azhar,E.I., El-Kafrawy,S.A., Farraj,S.A., Hassan,A.M., Al-Saeed,M.S., Hashem,A.M., Madani,T.A., Alsaeed,M.S., Alawi,M.M. | 2014-05-01T00:00:00Z | ssRNA(+) | 1353 | S | Saudi Arabia | Camelus | |
| AHL18090.1 | Chu,D.K., Poon,L.L., Gomaa,M.M., Shehata,M.M., Perera,R.A., Abu Zeid,D., El Rifay,A.S., Siu,L.Y., Guan,Y., Webby,R.J., Ali,M.A., Peiris,M., Kayali,G., Chu,D.K.W., Poon,L.L.M., Ali,M., Peiris,M.J.S. | 2014-03-04T00:00:00Z | ssRNA(+) | 1352 | S | Egypt | Camelus dromedarius | |
| WBY50246.1 | Zhou,Z., Ali,A., Walelign,E., Demissie,G.F., Masry,I.E., Abayneh,T., Getachew,B., Krishnan,P., Ng,D.Y.M., Gardner,E., Makonnen,Y., Miguel,E., Chevalier,V., Chu,D.K., So,R.T.Y., Von Dobschuetz,S., Mamo,G., Poon,L.L.M., Peiris,M., Masry,I., Dobschuetz,S., Poon,L. | 2023-01-28T00:00:00Z | ssRNA(+) | 1353 | S protein | Ethiopia | Camelus dromedarius | |
| WBY50257.1 | Zhou,Z., Ali,A., Walelign,E., Demissie,G.F., Masry,I.E., Abayneh,T., Getachew,B., Krishnan,P., Ng,D.Y.M., Gardner,E., Makonnen,Y., Miguel,E., Chevalier,V., Chu,D.K., So,R.T.Y., Von Dobschuetz,S., Mamo,G., Poon,L.L.M., Peiris,M., Masry,I., Dobschuetz,S., Poon,L. | 2023-01-28T00:00:00Z | ssRNA(+) | 1353 | S protein | Ethiopia | Camelus dromedarius | |
| WBY50268.1 | Zhou,Z., Ali,A., Walelign,E., Demissie,G.F., Masry,I.E., Abayneh,T., Getachew,B., Krishnan,P., Ng,D.Y.M., Gardner,E., Makonnen,Y., Miguel,E., Chevalier,V., Chu,D.K., So,R.T.Y., Von Dobschuetz,S., Mamo,G., Poon,L.L.M., Peiris,M., Masry,I., Dobschuetz,S., Poon,L. | 2023-01-28T00:00:00Z | ssRNA(+) | 1353 | S protein | Ethiopia | Camelus dromedarius | |
| WBY50279.1 | Zhou,Z., Ali,A., Walelign,E., Demissie,G.F., Masry,I.E., Abayneh,T., Getachew,B., Krishnan,P., Ng,D.Y.M., Gardner,E., Makonnen,Y., Miguel,E., Chevalier,V., Chu,D.K., So,R.T.Y., Von Dobschuetz,S., Mamo,G., Poon,L.L.M., Peiris,M., Masry,I., Dobschuetz,S., Poon,L. | 2023-01-28T00:00:00Z | ssRNA(+) | 1353 | S protein | Ethiopia | Camelus dromedarius | |
| WBY50290.1 | Zhou,Z., Ali,A., Walelign,E., Demissie,G.F., Masry,I.E., Abayneh,T., Getachew,B., Krishnan,P., Ng,D.Y.M., Gardner,E., Makonnen,Y., Miguel,E., Chevalier,V., Chu,D.K., So,R.T.Y., Von Dobschuetz,S., Mamo,G., Poon,L.L.M., Peiris,M., Masry,I., Dobschuetz,S., Poon,L. | 2023-01-28T00:00:00Z | ssRNA(+) | 1353 | S protein | Ethiopia | Camelus dromedarius | |
| WBY50301.1 | Zhou,Z., Ali,A., Walelign,E., Demissie,G.F., Masry,I.E., Abayneh,T., Getachew,B., Krishnan,P., Ng,D.Y.M., Gardner,E., Makonnen,Y., Miguel,E., Chevalier,V., Chu,D.K., So,R.T.Y., Von Dobschuetz,S., Mamo,G., Poon,L.L.M., Peiris,M., Masry,I., Dobschuetz,S., Poon,L. | 2023-01-28T00:00:00Z | ssRNA(+) | 1353 | S protein | Ethiopia | Camelus dromedarius | |
| WBY50312.1 | Zhou,Z., Ali,A., Walelign,E., Demissie,G.F., Masry,I.E., Abayneh,T., Getachew,B., Krishnan,P., Ng,D.Y.M., Gardner,E., Makonnen,Y., Miguel,E., Chevalier,V., Chu,D.K., So,R.T.Y., Von Dobschuetz,S., Mamo,G., Poon,L.L.M., Peiris,M., Masry,I., Dobschuetz,S., Poon,L. | 2023-01-28T00:00:00Z | ssRNA(+) | 1353 | S protein | Ethiopia | Camelus dromedarius | |
| WBY50323.1 | Zhou,Z., Ali,A., Walelign,E., Demissie,G.F., Masry,I.E., Abayneh,T., Getachew,B., Krishnan,P., Ng,D.Y.M., Gardner,E., Makonnen,Y., Miguel,E., Chevalier,V., Chu,D.K., So,R.T.Y., Von Dobschuetz,S., Mamo,G., Poon,L.L.M., Peiris,M., Masry,I., Dobschuetz,S., Poon,L. | 2023-01-28T00:00:00Z | ssRNA(+) | 1353 | S protein | Ethiopia | Camelus dromedarius | |
| WBY50334.1 | Zhou,Z., Ali,A., Walelign,E., Demissie,G.F., Masry,I.E., Abayneh,T., Getachew,B., Krishnan,P., Ng,D.Y.M., Gardner,E., Makonnen,Y., Miguel,E., Chevalier,V., Chu,D.K., So,R.T.Y., Von Dobschuetz,S., Mamo,G., Poon,L.L.M., Peiris,M., Masry,I., Dobschuetz,S., Poon,L. | 2023-01-28T00:00:00Z | ssRNA(+) | 1353 | S protein | Ethiopia | Camelus dromedarius | |
| WBY50345.1 | Zhou,Z., Ali,A., Walelign,E., Demissie,G.F., Masry,I.E., Abayneh,T., Getachew,B., Krishnan,P., Ng,D.Y.M., Gardner,E., Makonnen,Y., Miguel,E., Chevalier,V., Chu,D.K., So,R.T.Y., Von Dobschuetz,S., Mamo,G., Poon,L.L.M., Peiris,M., Masry,I., Dobschuetz,S., Poon,L. | 2023-01-28T00:00:00Z | ssRNA(+) | 1353 | S protein | Ethiopia | Camelus dromedarius | |
| WBY50356.1 | Zhou,Z., Ali,A., Walelign,E., Demissie,G.F., Masry,I.E., Abayneh,T., Getachew,B., Krishnan,P., Ng,D.Y.M., Gardner,E., Makonnen,Y., Miguel,E., Chevalier,V., Chu,D.K., So,R.T.Y., Von Dobschuetz,S., Mamo,G., Poon,L.L.M., Peiris,M., Masry,I., Dobschuetz,S., Poon,L. | 2023-01-28T00:00:00Z | ssRNA(+) | 1353 | S protein | Ethiopia | Camelus dromedarius | |
| WBY50367.1 | Zhou,Z., Ali,A., Walelign,E., Demissie,G.F., Masry,I.E., Abayneh,T., Getachew,B., Krishnan,P., Ng,D.Y.M., Gardner,E., Makonnen,Y., Miguel,E., Chevalier,V., Chu,D.K., So,R.T.Y., Von Dobschuetz,S., Mamo,G., Poon,L.L.M., Peiris,M., Masry,I., Dobschuetz,S., Poon,L. | 2023-01-28T00:00:00Z | ssRNA(+) | 1353 | S protein | Ethiopia | Camelus dromedarius | |
| WBY50376.1 | Zhou,Z., Ali,A., Walelign,E., Demissie,G.F., Masry,I.E., Abayneh,T., Getachew,B., Krishnan,P., Ng,D.Y.M., Gardner,E., Makonnen,Y., Miguel,E., Chevalier,V., Chu,D.K., So,R.T.Y., Von Dobschuetz,S., Mamo,G., Poon,L.L.M., Peiris,M., Masry,I., Dobschuetz,S., Poon,L. | 2023-01-28T00:00:00Z | ssRNA(+) | 1353 | S protein | Ethiopia | Camelus dromedarius | |
| WBY50387.1 | Zhou,Z., Ali,A., Walelign,E., Demissie,G.F., Masry,I.E., Abayneh,T., Getachew,B., Krishnan,P., Ng,D.Y.M., Gardner,E., Makonnen,Y., Miguel,E., Chevalier,V., Chu,D.K., So,R.T.Y., Von Dobschuetz,S., Mamo,G., Poon,L.L.M., Peiris,M., Masry,I., Dobschuetz,S., Poon,L. | 2023-01-28T00:00:00Z | ssRNA(+) | 1353 | S protein | Ethiopia | Camelus dromedarius | |
| WBY50398.1 | Zhou,Z., Ali,A., Walelign,E., Demissie,G.F., Masry,I.E., Abayneh,T., Getachew,B., Krishnan,P., Ng,D.Y.M., Gardner,E., Makonnen,Y., Miguel,E., Chevalier,V., Chu,D.K., So,R.T.Y., Von Dobschuetz,S., Mamo,G., Poon,L.L.M., Peiris,M., Masry,I., Dobschuetz,S., Poon,L. | 2023-01-28T00:00:00Z | ssRNA(+) | 1353 | S protein | Ethiopia | Camelus dromedarius | |
| WBY50409.1 | Zhou,Z., Ali,A., Walelign,E., Demissie,G.F., Masry,I.E., Abayneh,T., Getachew,B., Krishnan,P., Ng,D.Y.M., Gardner,E., Makonnen,Y., Miguel,E., Chevalier,V., Chu,D.K., So,R.T.Y., Von Dobschuetz,S., Mamo,G., Poon,L.L.M., Peiris,M., Masry,I., Dobschuetz,S., Poon,L. | 2023-01-28T00:00:00Z | ssRNA(+) | 1353 | S protein | Ethiopia | Camelus dromedarius | |
| WBY50420.1 | Zhou,Z., Ali,A., Walelign,E., Demissie,G.F., Masry,I.E., Abayneh,T., Getachew,B., Krishnan,P., Ng,D.Y.M., Gardner,E., Makonnen,Y., Miguel,E., Chevalier,V., Chu,D.K., So,R.T.Y., Von Dobschuetz,S., Mamo,G., Poon,L.L.M., Peiris,M., Masry,I., Dobschuetz,S., Poon,L. | 2023-01-28T00:00:00Z | ssRNA(+) | 1353 | S protein | Ethiopia | Camelus dromedarius | |
| WBY50431.1 | Zhou,Z., Ali,A., Walelign,E., Demissie,G.F., Masry,I.E., Abayneh,T., Getachew,B., Krishnan,P., Ng,D.Y.M., Gardner,E., Makonnen,Y., Miguel,E., Chevalier,V., Chu,D.K., So,R.T.Y., Von Dobschuetz,S., Mamo,G., Poon,L.L.M., Peiris,M., Masry,I., Dobschuetz,S., Poon,L. | 2023-01-28T00:00:00Z | ssRNA(+) | 1353 | S protein | Ethiopia | Camelus dromedarius | |
| WBY50442.1 | Zhou,Z., Ali,A., Walelign,E., Demissie,G.F., Masry,I.E., Abayneh,T., Getachew,B., Krishnan,P., Ng,D.Y.M., Gardner,E., Makonnen,Y., Miguel,E., Chevalier,V., Chu,D.K., So,R.T.Y., Von Dobschuetz,S., Mamo,G., Poon,L.L.M., Peiris,M., Masry,I., Dobschuetz,S., Poon,L. | 2023-01-28T00:00:00Z | ssRNA(+) | 1353 | S protein | Ethiopia | Camelus dromedarius | |
| WBY50453.1 | Zhou,Z., Ali,A., Walelign,E., Demissie,G.F., Masry,I.E., Abayneh,T., Getachew,B., Krishnan,P., Ng,D.Y.M., Gardner,E., Makonnen,Y., Miguel,E., Chevalier,V., Chu,D.K., So,R.T.Y., Von Dobschuetz,S., Mamo,G., Poon,L.L.M., Peiris,M., Masry,I., Dobschuetz,S., Poon,L. | 2023-01-28T00:00:00Z | ssRNA(+) | 1353 | S protein | Ethiopia | Camelus dromedarius | |
| WBY50464.1 | Zhou,Z., Ali,A., Walelign,E., Demissie,G.F., Masry,I.E., Abayneh,T., Getachew,B., Krishnan,P., Ng,D.Y.M., Gardner,E., Makonnen,Y., Miguel,E., Chevalier,V., Chu,D.K., So,R.T.Y., Von Dobschuetz,S., Mamo,G., Poon,L.L.M., Peiris,M., Masry,I., Dobschuetz,S., Poon,L. | 2023-01-28T00:00:00Z | ssRNA(+) | 1353 | S protein | Ethiopia | Camelus dromedarius | |
| WAM65128.1 | Rodon,J., Mykytyn,A.Z., Te,N., Okba,N.M.A., Lamers,M.M., Pailler-Garcia,L., Cantero,G., Albulescu,I., Bosch,B.-J., Peiris,M., Bensaid,A., Vergara-Alert,J., Haagmans,B., Segales,J. | 2022-12-17T00:00:00Z | ssRNA(+) | 1353 | S protein | Egypt | Camelus dromedarius | |
| QWQ31042.1 | Zhou,Z., Hui,K.P.Y., So,R.T.Y., Lv,H., Perera,R.A.P.M., Chu,D.K.W., Gelaye,E., Oyas,H., Njagi,O., Abayneh,T., Kuria,W., Walelign,E., Wanglia,R., El Masry,I., Von Dobschuetz,S., Kalpravidh,W., Chevalier,V., Miguel,E., Fassi-Fihri,O., Trarore,A., Liang,W., Wang,Y., Nicholls,J.M., Zhao,J., Chan,M.C.W., Poon,L.L.M., Mok,C.K.P., Peiris,M., Getachew,B., Adamu,K., Rufael,T., El-Masry,I., Dobschuetz,S.V., Chu,D.K. | 2022-08-31T00:00:00Z | ssRNA(+) | 1353 | S protein | Ethiopia | Camelus dromedarius | |
| QWQ31053.1 | Zhou,Z., Hui,K.P.Y., So,R.T.Y., Lv,H., Perera,R.A.P.M., Chu,D.K.W., Gelaye,E., Oyas,H., Njagi,O., Abayneh,T., Kuria,W., Walelign,E., Wanglia,R., El Masry,I., Von Dobschuetz,S., Kalpravidh,W., Chevalier,V., Miguel,E., Fassi-Fihri,O., Trarore,A., Liang,W., Wang,Y., Nicholls,J.M., Zhao,J., Chan,M.C.W., Poon,L.L.M., Mok,C.K.P., Peiris,M., El-Masry,I., Dobschuetz,S.V., Chu,D.K. | 2022-08-31T00:00:00Z | ssRNA(+) | 1353 | S protein | Kenya | Camelus dromedarius | |
| USF97409.1 | Kubacki,J., Wiederkehr,M., Fraefel,C. | 2022-06-21T00:00:00Z | ssRNA(+) | 1354 | S protein | Switzerland | Vespertilio murinus | |
| USF97418.1 | Kubacki,J., Wiederkehr,M., Fraefel,C. | 2022-06-21T00:00:00Z | ssRNA(+) | 1348 | S protein | Switzerland | Vespertilio murinus | |
| QMS54774.1 | Hardmeier,I.S., Aeberhard,N., Qi,W., Kraettli,H., Fraefel,C., Kubacki,J. | 2021-01-30T00:00:00Z | ssRNA(+) | 568 | S protein | Switzerland | Vespertilio murinus | |
| QHB14951.1 | Chu,D.K.W., Hemida,M.G., Perera,R.A.P.M., Chan,C.-k., Krishnan,P., Chor,Y.-y., Chan,K.-h., Ng,D.Y.M., Poon,L.M., Alnaeem,A., Peiris,M. | 2021-01-03T00:00:00Z | ssRNA(+) | 1353 | S protein | Saudi Arabia | Camelus dromedarius | |
| QHB14962.1 | Chu,D.K.W., Hemida,M.G., Perera,R.A.P.M., Chan,C.-k., Krishnan,P., Chor,Y.-y., Chan,K.-h., Ng,D.Y.M., Poon,L.M., Alnaeem,A., Peiris,M. | 2021-01-03T00:00:00Z | ssRNA(+) | 1353 | S protein | Saudi Arabia | Camelus dromedarius | |
| QHB14973.1 | Chu,D.K.W., Hemida,M.G., Perera,R.A.P.M., Chan,C.-k., Krishnan,P., Chor,Y.-y., Chan,K.-h., Ng,D.Y.M., Poon,L.M., Alnaeem,A., Peiris,M. | 2021-01-03T00:00:00Z | ssRNA(+) | 1353 | S protein | Saudi Arabia | Camelus dromedarius | |
| QHB14984.1 | Chu,D.K.W., Hemida,M.G., Perera,R.A.P.M., Chan,C.-k., Krishnan,P., Chor,Y.-y., Chan,K.-h., Ng,D.Y.M., Poon,L.M., Alnaeem,A., Peiris,M. | 2021-01-03T00:00:00Z | ssRNA(+) | 1353 | S protein | Saudi Arabia | Camelus dromedarius | |
| QHB14995.1 | Chu,D.K.W., Hemida,M.G., Perera,R.A.P.M., Chan,C.-k., Krishnan,P., Chor,Y.-y., Chan,K.-h., Ng,D.Y.M., Poon,L.M., Alnaeem,A., Peiris,M. | 2021-01-03T00:00:00Z | ssRNA(+) | 1353 | S protein | Saudi Arabia | Camelus dromedarius | |
| QHB15006.1 | Chu,D.K.W., Hemida,M.G., Perera,R.A.P.M., Chan,C.-k., Krishnan,P., Chor,Y.-y., Chan,K.-h., Ng,D.Y.M., Poon,L.M., Alnaeem,A., Peiris,M. | 2021-01-03T00:00:00Z | ssRNA(+) | 1353 | S protein | Saudi Arabia | Camelus dromedarius | |
| QHB15017.1 | Chu,D.K.W., Hemida,M.G., Perera,R.A.P.M., Chan,C.-k., Krishnan,P., Chor,Y.-y., Chan,K.-h., Ng,D.Y.M., Poon,L.M., Alnaeem,A., Peiris,M. | 2021-01-03T00:00:00Z | ssRNA(+) | 1353 | S protein | Saudi Arabia | Camelus dromedarius | |
| QHB15028.1 | Chu,D.K.W., Hemida,M.G., Perera,R.A.P.M., Chan,C.-k., Krishnan,P., Chor,Y.-y., Chan,K.-h., Ng,D.Y.M., Poon,L.M., Alnaeem,A., Peiris,M. | 2021-01-03T00:00:00Z | ssRNA(+) | 1353 | S protein | Saudi Arabia | Camelus dromedarius | |
| QHB15039.1 | Chu,D.K.W., Hemida,M.G., Perera,R.A.P.M., Chan,C.-k., Krishnan,P., Chor,Y.-y., Chan,K.-h., Ng,D.Y.M., Poon,L.M., Alnaeem,A., Peiris,M. | 2021-01-03T00:00:00Z | ssRNA(+) | 1353 | S protein | Saudi Arabia | Camelus dromedarius | |
| QGW51391.1 | Chu,D.K.W., Hemida,M., Chor,Y., Cheng,S.M., Alnaeem,A., Poon,L.M., Peiris,M. | 2020-12-17T00:00:00Z | ssRNA(+) | 1353 | S protein | Saudi Arabia | Camelus dromedarius | |
| QGW51401.1 | Chu,D.K.W., Hemida,M., Chor,Y., Cheng,S.M., Alnaeem,A., Poon,L.M., Peiris,M. | 2020-12-17T00:00:00Z | ssRNA(+) | 1353 | S protein | Saudi Arabia | Camelus dromedarius | |
| QGW51411.1 | Chu,D.K.W., Hemida,M., Chor,Y., Cheng,S.M., Alnaeem,A., Poon,L.M., Peiris,M. | 2020-12-17T00:00:00Z | ssRNA(+) | 1353 | S protein | Saudi Arabia | Camelus dromedarius | |
| QGW51421.1 | Chu,D.K.W., Hemida,M., Chor,Y., Cheng,S.M., Alnaeem,A., Poon,L.M., Peiris,M. | 2020-12-17T00:00:00Z | ssRNA(+) | 1353 | S protein | Saudi Arabia | Camelus dromedarius | |
| QGW51431.1 | Chu,D.K.W., Hemida,M., Chor,Y., Cheng,S.M., Alnaeem,A., Poon,L.M., Peiris,M. | 2020-12-17T00:00:00Z | ssRNA(+) | 1353 | S protein | Saudi Arabia | Camelus dromedarius | |
| QGW51441.1 | Chu,D.K.W., Hemida,M., Chor,Y., Cheng,S.M., Alnaeem,A., Poon,L.M., Peiris,M. | 2020-12-17T00:00:00Z | ssRNA(+) | 1353 | S protein | Saudi Arabia | Camelus dromedarius | |
| QGW51451.1 | Chu,D.K.W., Hemida,M., Chor,Y., Cheng,S.M., Alnaeem,A., Poon,L.M., Peiris,M. | 2020-12-17T00:00:00Z | ssRNA(+) | 1353 | S protein | Saudi Arabia | Camelus dromedarius | |
| QGW51461.1 | Chu,D.K.W., Hemida,M., Chor,Y., Cheng,S.M., Alnaeem,A., Poon,L.M., Peiris,M. | 2020-12-17T00:00:00Z | ssRNA(+) | 1353 | S protein | Saudi Arabia | Camelus dromedarius | |
| QGW51471.1 | Chu,D.K.W., Hemida,M., Chor,Y., Cheng,S.M., Alnaeem,A., Poon,L.M., Peiris,M. | 2020-12-17T00:00:00Z | ssRNA(+) | 1353 | S protein | Saudi Arabia | Camelus dromedarius | |
| QGW51481.1 | Chu,D.K.W., Hemida,M., Chor,Y., Cheng,S.M., Alnaeem,A., Poon,L.M., Peiris,M. | 2020-12-17T00:00:00Z | ssRNA(+) | 1353 | S protein | Saudi Arabia | Camelus dromedarius | |
| QGW51491.1 | Chu,D.K.W., Hemida,M., Chor,Y., Cheng,S.M., Alnaeem,A., Poon,L.M., Peiris,M. | 2020-12-17T00:00:00Z | ssRNA(+) | 1353 | S protein | Saudi Arabia | Camelus dromedarius | |
| QGW51501.1 | Chu,D.K.W., Hemida,M., Chor,Y., Cheng,S.M., Alnaeem,A., Poon,L.M., Peiris,M. | 2020-12-17T00:00:00Z | ssRNA(+) | 1353 | S protein | Saudi Arabia | Camelus dromedarius | |
| QGW51511.1 | Chu,D.K.W., Hemida,M., Chor,Y., Cheng,S.M., Alnaeem,A., Poon,L.M., Peiris,M. | 2020-12-17T00:00:00Z | ssRNA(+) | 1353 | S protein | Saudi Arabia | Camelus dromedarius | |
| QGW51521.1 | Chu,D.K.W., Hemida,M., Chor,Y., Cheng,S.M., Alnaeem,A., Poon,L.M., Peiris,M. | 2020-12-17T00:00:00Z | ssRNA(+) | 1353 | S protein | Saudi Arabia | Camelus dromedarius | |
| QGW51531.1 | Chu,D.K.W., Hemida,M., Chor,Y., Cheng,S.M., Alnaeem,A., Poon,L.M., Peiris,M. | 2020-12-17T00:00:00Z | ssRNA(+) | 1353 | S protein | Saudi Arabia | Camelus dromedarius | |
| QGW51541.1 | Chu,D.K.W., Hemida,M., Chor,Y., Cheng,S.M., Alnaeem,A., Poon,L.M., Peiris,M. | 2020-12-17T00:00:00Z | ssRNA(+) | 1353 | S protein | Saudi Arabia | Camelus dromedarius | |
| QGW51551.1 | Chu,D.K.W., Hemida,M., Chor,Y., Cheng,S.M., Alnaeem,A., Poon,L.M., Peiris,M. | 2020-12-17T00:00:00Z | ssRNA(+) | 1353 | S protein | Saudi Arabia | Camelus dromedarius | |
| QGW51561.1 | Chu,D.K.W., Hemida,M., Chor,Y., Cheng,S.M., Alnaeem,A., Poon,L.M., Peiris,M. | 2020-12-17T00:00:00Z | ssRNA(+) | 1353 | S protein | Saudi Arabia | Camelus dromedarius | |
| QGW51571.1 | Chu,D.K.W., Hemida,M., Chor,Y., Cheng,S.M., Alnaeem,A., Poon,L.M., Peiris,M. | 2020-12-17T00:00:00Z | ssRNA(+) | 1353 | S protein | Saudi Arabia | Camelus dromedarius | |
| QGW51581.1 | Chu,D.K.W., Hemida,M., Chor,Y., Cheng,S.M., Alnaeem,A., Poon,L.M., Peiris,M. | 2020-12-17T00:00:00Z | ssRNA(+) | 1353 | S protein | Saudi Arabia | Camelus dromedarius | |
| QGW51591.1 | Chu,D.K.W., Hemida,M., Chor,Y., Cheng,S.M., Alnaeem,A., Poon,L.M., Peiris,M. | 2020-12-17T00:00:00Z | ssRNA(+) | 1353 | S protein | Saudi Arabia | Camelus dromedarius | |
| QGW51601.1 | Chu,D.K.W., Hemida,M., Chor,Y., Cheng,S.M., Alnaeem,A., Poon,L.M., Peiris,M. | 2020-12-17T00:00:00Z | ssRNA(+) | 1353 | S protein | Saudi Arabia | Camelus dromedarius | |
| QGW51611.1 | Chu,D.K.W., Hemida,M., Chor,Y., Cheng,S.M., Alnaeem,A., Poon,L.M., Peiris,M. | 2020-12-17T00:00:00Z | ssRNA(+) | 1353 | S protein | Saudi Arabia | Camelus dromedarius | |
| QGW51621.1 | Chu,D.K.W., Hemida,M., Chor,Y., Cheng,S.M., Alnaeem,A., Poon,L.M., Peiris,M. | 2020-12-17T00:00:00Z | ssRNA(+) | 1353 | S protein | Saudi Arabia | Camelus dromedarius | |
| QGW51631.1 | Chu,D.K.W., Hemida,M., Chor,Y., Cheng,S.M., Alnaeem,A., Poon,L.M., Peiris,M. | 2020-12-17T00:00:00Z | ssRNA(+) | 1353 | S protein | Saudi Arabia | Camelus dromedarius | |
| QGW51641.1 | Chu,D.K.W., Hemida,M., Chor,Y., Cheng,S.M., Alnaeem,A., Poon,L.M., Peiris,M. | 2020-12-17T00:00:00Z | ssRNA(+) | 1353 | S protein | Saudi Arabia | Camelus dromedarius | |
| QGW51651.1 | Chu,D.K.W., Hemida,M., Chor,Y., Cheng,S.M., Alnaeem,A., Poon,L.M., Peiris,M. | 2020-12-17T00:00:00Z | ssRNA(+) | 1353 | S protein | Saudi Arabia | Camelus dromedarius | |
| QGW51661.1 | Chu,D.K.W., Hemida,M., Chor,Y., Cheng,S.M., Alnaeem,A., Poon,L.M., Peiris,M. | 2020-12-17T00:00:00Z | ssRNA(+) | 1353 | S protein | Saudi Arabia | Camelus dromedarius | |
| QGW51671.1 | Chu,D.K.W., Hemida,M., Chor,Y., Cheng,S.M., Alnaeem,A., Poon,L.M., Peiris,M. | 2020-12-17T00:00:00Z | ssRNA(+) | 1353 | S protein | Saudi Arabia | Camelus dromedarius | |
| QGW51681.1 | Chu,D.K.W., Hemida,M., Chor,Y., Cheng,S.M., Alnaeem,A., Poon,L.M., Peiris,M. | 2020-12-17T00:00:00Z | ssRNA(+) | 1353 | S protein | Saudi Arabia | Camelus dromedarius | |
| QGW51691.1 | Chu,D.K.W., Hemida,M., Chor,Y., Cheng,S.M., Alnaeem,A., Poon,L.M., Peiris,M. | 2020-12-17T00:00:00Z | ssRNA(+) | 1353 | S protein | Saudi Arabia | Camelus dromedarius | |
| QGW51701.1 | Chu,D.K.W., Hemida,M., Chor,Y., Cheng,S.M., Alnaeem,A., Poon,L.M., Peiris,M. | 2020-12-17T00:00:00Z | ssRNA(+) | 1353 | S protein | Saudi Arabia | Camelus dromedarius | |
| QGW51711.1 | Chu,D.K.W., Hemida,M., Chor,Y., Cheng,S.M., Alnaeem,A., Poon,L.M., Peiris,M. | 2020-12-17T00:00:00Z | ssRNA(+) | 1353 | S protein | Saudi Arabia | Camelus dromedarius | |
| QGW51721.1 | Chu,D.K.W., Hemida,M., Chor,Y., Cheng,S.M., Alnaeem,A., Poon,L.M., Peiris,M. | 2020-12-17T00:00:00Z | ssRNA(+) | 1353 | S protein | Saudi Arabia | Camelus dromedarius | |
| QGW51731.1 | Chu,D.K.W., Hemida,M., Chor,Y., Cheng,S.M., Alnaeem,A., Poon,L.M., Peiris,M. | 2020-12-17T00:00:00Z | ssRNA(+) | 1353 | S protein | Saudi Arabia | Camelus dromedarius | |
| QGW51741.1 | Chu,D.K.W., Hemida,M., Chor,Y., Cheng,S.M., Alnaeem,A., Poon,L.M., Peiris,M. | 2020-12-17T00:00:00Z | ssRNA(+) | 1353 | S protein | Saudi Arabia | Camelus dromedarius | |
| QGW51751.1 | Chu,D.K.W., Hemida,M., Chor,Y., Cheng,S.M., Alnaeem,A., Poon,L.M., Peiris,M. | 2020-12-17T00:00:00Z | ssRNA(+) | 1353 | S protein | Saudi Arabia | Camelus dromedarius | |
| QGW51761.1 | Chu,D.K.W., Hemida,M., Chor,Y., Cheng,S.M., Alnaeem,A., Poon,L.M., Peiris,M. | 2020-12-17T00:00:00Z | ssRNA(+) | 1353 | S protein | Saudi Arabia | Camelus dromedarius | |
| QGW51771.1 | Chu,D.K.W., Hemida,M., Chor,Y., Cheng,S.M., Alnaeem,A., Poon,L.M., Peiris,M. | 2020-12-17T00:00:00Z | ssRNA(+) | 1353 | S protein | Saudi Arabia | Camelus dromedarius | |
| QGW51781.1 | Chu,D.K.W., Hemida,M., Chor,Y., Cheng,S.M., Alnaeem,A., Poon,L.M., Peiris,M. | 2020-12-17T00:00:00Z | ssRNA(+) | 1353 | S protein | Saudi Arabia | Camelus dromedarius | |
| QGW51791.1 | Chu,D.K.W., Hemida,M., Chor,Y., Cheng,S.M., Alnaeem,A., Poon,L.M., Peiris,M. | 2020-12-17T00:00:00Z | ssRNA(+) | 1353 | S protein | Saudi Arabia | Camelus dromedarius | |
| QGW51801.1 | Chu,D.K.W., Hemida,M., Chor,Y., Cheng,S.M., Alnaeem,A., Poon,L.M., Peiris,M. | 2020-12-17T00:00:00Z | ssRNA(+) | 1353 | S protein | Saudi Arabia | Camelus dromedarius | |
| QGW51811.1 | Chu,D.K.W., Hemida,M., Chor,Y., Cheng,S.M., Alnaeem,A., Poon,L.M., Peiris,M. | 2020-12-17T00:00:00Z | ssRNA(+) | 1353 | S protein | Saudi Arabia | Camelus dromedarius | |
| QGW51821.1 | Chu,D.K.W., Hemida,M., Chor,Y., Cheng,S.M., Alnaeem,A., Poon,L.M., Peiris,M. | 2020-12-17T00:00:00Z | ssRNA(+) | 1353 | S protein | Saudi Arabia | Camelus dromedarius | |
| QGW51831.1 | Chu,D.K.W., Hemida,M., Chor,Y., Cheng,S.M., Alnaeem,A., Poon,L.M., Peiris,M. | 2020-12-17T00:00:00Z | ssRNA(+) | 1353 | S protein | Saudi Arabia | Camelus dromedarius | |
| QGW51841.1 | Chu,D.K.W., Hemida,M., Chor,Y., Cheng,S.M., Alnaeem,A., Poon,L.M., Peiris,M. | 2020-12-17T00:00:00Z | ssRNA(+) | 1353 | S protein | Saudi Arabia | Camelus dromedarius | |
| QGW51851.1 | Chu,D.K.W., Hemida,M., Chor,Y., Cheng,S.M., Alnaeem,A., Poon,L.M., Peiris,M. | 2020-12-17T00:00:00Z | ssRNA(+) | 1353 | S protein | Saudi Arabia | Camelus dromedarius | |
| QGW51861.1 | Chu,D.K.W., Hemida,M., Chor,Y., Cheng,S.M., Alnaeem,A., Poon,L.M., Peiris,M. | 2020-12-17T00:00:00Z | ssRNA(+) | 1353 | S protein | Saudi Arabia | Camelus dromedarius | |
| QOU08497.1 | Seifert,S.N., Munster,V.J. | 2020-11-03T00:00:00Z | ssRNA(+) | 1353 | S protein | Jordan | Camelus dromedarius | |
| QOU08508.1 | Seifert,S.N., Munster,V.J. | 2020-11-03T00:00:00Z | ssRNA(+) | 1353 | S protein | Jordan | Camelus dromedarius | |
| QOU08519.1 | Seifert,S.N., Munster,V.J. | 2020-11-03T00:00:00Z | ssRNA(+) | 1353 | S protein | Jordan | Camelus dromedarius | |
| QOU08530.1 | Seifert,S.N., Munster,V.J. | 2020-11-03T00:00:00Z | ssRNA(+) | 1353 | S protein | Jordan | Camelus dromedarius | |
| QOU08541.1 | Seifert,S.N., Munster,V.J. | 2020-11-03T00:00:00Z | ssRNA(+) | 1353 | S protein | Jordan | Camelus dromedarius | |
| QOU08552.1 | Seifert,S.N., Munster,V.J. | 2020-11-03T00:00:00Z | ssRNA(+) | 1353 | S protein | Jordan | Camelus dromedarius | |
| QOU08563.1 | Seifert,S.N., Munster,V.J. | 2020-11-03T00:00:00Z | ssRNA(+) | 1353 | S protein | Jordan | Camelus dromedarius | |
| QOU08574.1 | Seifert,S.N., Munster,V.J. | 2020-11-03T00:00:00Z | ssRNA(+) | 1353 | S protein | Jordan | Camelus dromedarius | |
| QOU08585.1 | Seifert,S.N., Munster,V.J. | 2020-11-03T00:00:00Z | ssRNA(+) | 1353 | S protein | Jordan | Camelus dromedarius | |
| QOU08594.1 | Seifert,S.N., Munster,V.J. | 2020-11-03T00:00:00Z | ssRNA(+) | 1353 | S protein | Jordan | Camelus dromedarius | |
| QOU08603.1 | Seifert,S.N., Munster,V.J. | 2020-11-03T00:00:00Z | ssRNA(+) | 1353 | S protein | Jordan | Camelus dromedarius | |
| QOU08614.1 | Seifert,S.N., Munster,V.J. | 2020-11-03T00:00:00Z | ssRNA(+) | 1353 | S protein | Jordan | Camelus dromedarius | |
| QOU08625.1 | Seifert,S.N., Munster,V.J. | 2020-11-03T00:00:00Z | ssRNA(+) | 1353 | S protein | Jordan | Camelus dromedarius | |
| QOU08636.1 | Seifert,S.N., Munster,V.J. | 2020-11-03T00:00:00Z | ssRNA(+) | 1079 | S protein | Jordan | Camelus dromedarius | |
| AWH65943.1 | Luo,C., Wang,N., Yang,X.-L., Liu,H.-Z., Zhang,W., Li,B., Hu,B., Peng,C., Zhu,G.-J., Shi,Z.-L. | 2020-02-23T00:00:00Z | ssRNA(+) | 1346 | S protein | China |  | |
| AWH65954.1 | Luo,C., Wang,N., Yang,X.-L., Liu,H.-Z., Zhang,W., Li,B., Hu,B., Peng,C., Zhu,G.-J., Shi,Z.-L. | 2020-02-23T00:00:00Z | ssRNA(+) | 1347 | S protein | China |  | |
| QFS19563.1 | El-Kafrawy,S.A., Corman,V.M., Tolah,A.M., Al Masaudi,S.B., Hassan,A.M., Muller,M.A., Bleicker,T., Harakeh,S.M., Alzahrani,A.A., Alsaaidi,G.A., Alagili,A.N., Hashem,A.M., Zumla,A., Drosten,C., Azhar,E.I. | 2020-01-06T00:00:00Z | ssRNA(+) | 348 | S protein | Saudi Arabia | Camelus dromedarius | |
| QFS19564.1 | El-Kafrawy,S.A., Corman,V.M., Tolah,A.M., Al Masaudi,S.B., Hassan,A.M., Muller,M.A., Bleicker,T., Harakeh,S.M., Alzahrani,A.A., Alsaaidi,G.A., Alagili,A.N., Hashem,A.M., Zumla,A., Drosten,C., Azhar,E.I. | 2020-01-06T00:00:00Z | ssRNA(+) | 348 | S protein | Saudi Arabia | Camelus dromedarius | |
| QFS19565.1 | El-Kafrawy,S.A., Corman,V.M., Tolah,A.M., Al Masaudi,S.B., Hassan,A.M., Muller,M.A., Bleicker,T., Harakeh,S.M., Alzahrani,A.A., Alsaaidi,G.A., Alagili,A.N., Hashem,A.M., Zumla,A., Drosten,C., Azhar,E.I. | 2020-01-06T00:00:00Z | ssRNA(+) | 348 | S protein | Saudi Arabia | Camelus dromedarius | |
| QFS19566.1 | El-Kafrawy,S.A., Corman,V.M., Tolah,A.M., Al Masaudi,S.B., Hassan,A.M., Muller,M.A., Bleicker,T., Harakeh,S.M., Alzahrani,A.A., Alsaaidi,G.A., Alagili,A.N., Hashem,A.M., Zumla,A., Drosten,C., Azhar,E.I. | 2020-01-06T00:00:00Z | ssRNA(+) | 348 | S protein | Saudi Arabia | Camelus dromedarius | |
| QFS19567.1 | El-Kafrawy,S.A., Corman,V.M., Tolah,A.M., Al Masaudi,S.B., Hassan,A.M., Muller,M.A., Bleicker,T., Harakeh,S.M., Alzahrani,A.A., Alsaaidi,G.A., Alagili,A.N., Hashem,A.M., Zumla,A., Drosten,C., Azhar,E.I. | 2020-01-06T00:00:00Z | ssRNA(+) | 348 | S protein | Saudi Arabia | Camelus dromedarius | |
| QFS19568.1 | El-Kafrawy,S.A., Corman,V.M., Tolah,A.M., Al Masaudi,S.B., Hassan,A.M., Muller,M.A., Bleicker,T., Harakeh,S.M., Alzahrani,A.A., Alsaaidi,G.A., Alagili,A.N., Hashem,A.M., Zumla,A., Drosten,C., Azhar,E.I. | 2020-01-06T00:00:00Z | ssRNA(+) | 348 | S protein | Saudi Arabia | Camelus dromedarius | |
| QFS19569.1 | El-Kafrawy,S.A., Corman,V.M., Tolah,A.M., Al Masaudi,S.B., Hassan,A.M., Muller,M.A., Bleicker,T., Harakeh,S.M., Alzahrani,A.A., Alsaaidi,G.A., Alagili,A.N., Hashem,A.M., Zumla,A., Drosten,C., Azhar,E.I. | 2020-01-06T00:00:00Z | ssRNA(+) | 348 | S protein | Saudi Arabia | Camelus dromedarius | |
| QFS19570.1 | El-Kafrawy,S.A., Corman,V.M., Tolah,A.M., Al Masaudi,S.B., Hassan,A.M., Muller,M.A., Bleicker,T., Harakeh,S.M., Alzahrani,A.A., Alsaaidi,G.A., Alagili,A.N., Hashem,A.M., Zumla,A., Drosten,C., Azhar,E.I. | 2020-01-06T00:00:00Z | ssRNA(+) | 348 | S protein | Saudi Arabia | Camelus dromedarius | |
| QFS19571.1 | El-Kafrawy,S.A., Corman,V.M., Tolah,A.M., Al Masaudi,S.B., Hassan,A.M., Muller,M.A., Bleicker,T., Harakeh,S.M., Alzahrani,A.A., Alsaaidi,G.A., Alagili,A.N., Hashem,A.M., Zumla,A., Drosten,C., Azhar,E.I. | 2020-01-06T00:00:00Z | ssRNA(+) | 348 | S protein | Saudi Arabia | Camelus dromedarius | |
| QFS19572.1 | El-Kafrawy,S.A., Corman,V.M., Tolah,A.M., Al Masaudi,S.B., Hassan,A.M., Muller,M.A., Bleicker,T., Harakeh,S.M., Alzahrani,A.A., Alsaaidi,G.A., Alagili,A.N., Hashem,A.M., Zumla,A., Drosten,C., Azhar,E.I. | 2020-01-06T00:00:00Z | ssRNA(+) | 348 | S protein | Saudi Arabia | Camelus dromedarius | |
| QFS19573.1 | El-Kafrawy,S.A., Corman,V.M., Tolah,A.M., Al Masaudi,S.B., Hassan,A.M., Muller,M.A., Bleicker,T., Harakeh,S.M., Alzahrani,A.A., Alsaaidi,G.A., Alagili,A.N., Hashem,A.M., Zumla,A., Drosten,C., Azhar,E.I. | 2020-01-06T00:00:00Z | ssRNA(+) | 348 | S protein | Saudi Arabia | Camelus dromedarius | |
| QFS19574.1 | El-Kafrawy,S.A., Corman,V.M., Tolah,A.M., Al Masaudi,S.B., Hassan,A.M., Muller,M.A., Bleicker,T., Harakeh,S.M., Alzahrani,A.A., Alsaaidi,G.A., Alagili,A.N., Hashem,A.M., Zumla,A., Drosten,C., Azhar,E.I. | 2020-01-06T00:00:00Z | ssRNA(+) | 348 | S protein | Saudi Arabia | Camelus dromedarius | |
| QFS19575.1 | El-Kafrawy,S.A., Corman,V.M., Tolah,A.M., Al Masaudi,S.B., Hassan,A.M., Muller,M.A., Bleicker,T., Harakeh,S.M., Alzahrani,A.A., Alsaaidi,G.A., Alagili,A.N., Hashem,A.M., Zumla,A., Drosten,C., Azhar,E.I. | 2020-01-06T00:00:00Z | ssRNA(+) | 348 | S protein | Saudi Arabia | Camelus dromedarius | |
| QFS19576.1 | El-Kafrawy,S.A., Corman,V.M., Tolah,A.M., Al Masaudi,S.B., Hassan,A.M., Muller,M.A., Bleicker,T., Harakeh,S.M., Alzahrani,A.A., Alsaaidi,G.A., Alagili,A.N., Hashem,A.M., Zumla,A., Drosten,C., Azhar,E.I. | 2020-01-06T00:00:00Z | ssRNA(+) | 348 | S protein | Saudi Arabia | Camelus dromedarius | |
| QFS19577.1 | El-Kafrawy,S.A., Corman,V.M., Tolah,A.M., Al Masaudi,S.B., Hassan,A.M., Muller,M.A., Bleicker,T., Harakeh,S.M., Alzahrani,A.A., Alsaaidi,G.A., Alagili,A.N., Hashem,A.M., Zumla,A., Drosten,C., Azhar,E.I. | 2020-01-06T00:00:00Z | ssRNA(+) | 348 | S protein | Saudi Arabia | Camelus dromedarius | |
| QFS19578.1 | El-Kafrawy,S.A., Corman,V.M., Tolah,A.M., Al Masaudi,S.B., Hassan,A.M., Muller,M.A., Bleicker,T., Harakeh,S.M., Alzahrani,A.A., Alsaaidi,G.A., Alagili,A.N., Hashem,A.M., Zumla,A., Drosten,C., Azhar,E.I. | 2020-01-06T00:00:00Z | ssRNA(+) | 348 | S protein | Saudi Arabia | Camelus dromedarius | |
| QFS19579.1 | El-Kafrawy,S.A., Corman,V.M., Tolah,A.M., Al Masaudi,S.B., Hassan,A.M., Muller,M.A., Bleicker,T., Harakeh,S.M., Alzahrani,A.A., Alsaaidi,G.A., Alagili,A.N., Hashem,A.M., Zumla,A., Drosten,C., Azhar,E.I. | 2020-01-06T00:00:00Z | ssRNA(+) | 348 | S protein | Saudi Arabia | Camelus dromedarius | |
| QFS19580.1 | El-Kafrawy,S.A., Corman,V.M., Tolah,A.M., Al Masaudi,S.B., Hassan,A.M., Muller,M.A., Bleicker,T., Harakeh,S.M., Alzahrani,A.A., Alsaaidi,G.A., Alagili,A.N., Hashem,A.M., Zumla,A., Drosten,C., Azhar,E.I. | 2020-01-06T00:00:00Z | ssRNA(+) | 348 | S protein | Saudi Arabia | Camelus dromedarius | |
| QFS19581.1 | El-Kafrawy,S.A., Corman,V.M., Tolah,A.M., Al Masaudi,S.B., Hassan,A.M., Muller,M.A., Bleicker,T., Harakeh,S.M., Alzahrani,A.A., Alsaaidi,G.A., Alagili,A.N., Hashem,A.M., Zumla,A., Drosten,C., Azhar,E.I. | 2020-01-06T00:00:00Z | ssRNA(+) | 348 | S protein | Saudi Arabia | Camelus dromedarius | |
| QFS19582.1 | El-Kafrawy,S.A., Corman,V.M., Tolah,A.M., Al Masaudi,S.B., Hassan,A.M., Muller,M.A., Bleicker,T., Harakeh,S.M., Alzahrani,A.A., Alsaaidi,G.A., Alagili,A.N., Hashem,A.M., Zumla,A., Drosten,C., Azhar,E.I. | 2020-01-06T00:00:00Z | ssRNA(+) | 348 | S protein | Saudi Arabia | Camelus dromedarius | |
| QFS19583.1 | El-Kafrawy,S.A., Corman,V.M., Tolah,A.M., Al Masaudi,S.B., Hassan,A.M., Muller,M.A., Bleicker,T., Harakeh,S.M., Alzahrani,A.A., Alsaaidi,G.A., Alagili,A.N., Hashem,A.M., Zumla,A., Drosten,C., Azhar,E.I. | 2020-01-06T00:00:00Z | ssRNA(+) | 348 | S protein | Saudi Arabia | Camelus dromedarius | |
| QFS19584.1 | El-Kafrawy,S.A., Corman,V.M., Tolah,A.M., Al Masaudi,S.B., Hassan,A.M., Muller,M.A., Bleicker,T., Harakeh,S.M., Alzahrani,A.A., Alsaaidi,G.A., Alagili,A.N., Hashem,A.M., Zumla,A., Drosten,C., Azhar,E.I. | 2020-01-06T00:00:00Z | ssRNA(+) | 348 | S protein | Saudi Arabia | Camelus dromedarius | |
| QFS19585.1 | El-Kafrawy,S.A., Corman,V.M., Tolah,A.M., Al Masaudi,S.B., Hassan,A.M., Muller,M.A., Bleicker,T., Harakeh,S.M., Alzahrani,A.A., Alsaaidi,G.A., Alagili,A.N., Hashem,A.M., Zumla,A., Drosten,C., Azhar,E.I. | 2020-01-06T00:00:00Z | ssRNA(+) | 348 | S protein | Saudi Arabia | Camelus dromedarius | |
| QFS19586.1 | El-Kafrawy,S.A., Corman,V.M., Tolah,A.M., Al Masaudi,S.B., Hassan,A.M., Muller,M.A., Bleicker,T., Harakeh,S.M., Alzahrani,A.A., Alsaaidi,G.A., Alagili,A.N., Hashem,A.M., Zumla,A., Drosten,C., Azhar,E.I. | 2020-01-06T00:00:00Z | ssRNA(+) | 348 | S protein | Saudi Arabia | Camelus dromedarius | |
| QFS19587.1 | El-Kafrawy,S.A., Corman,V.M., Tolah,A.M., Al Masaudi,S.B., Hassan,A.M., Muller,M.A., Bleicker,T., Harakeh,S.M., Alzahrani,A.A., Alsaaidi,G.A., Alagili,A.N., Hashem,A.M., Zumla,A., Drosten,C., Azhar,E.I. | 2020-01-06T00:00:00Z | ssRNA(+) | 348 | S protein | Djibouti | Camelus dromedarius | |
| QFS19588.1 | El-Kafrawy,S.A., Corman,V.M., Tolah,A.M., Al Masaudi,S.B., Hassan,A.M., Muller,M.A., Bleicker,T., Harakeh,S.M., Alzahrani,A.A., Alsaaidi,G.A., Alagili,A.N., Hashem,A.M., Zumla,A., Drosten,C., Azhar,E.I. | 2020-01-06T00:00:00Z | ssRNA(+) | 348 | S protein | Sudan | Camelus dromedarius | |
| QFS19589.1 | El-Kafrawy,S.A., Corman,V.M., Tolah,A.M., Al Masaudi,S.B., Hassan,A.M., Muller,M.A., Bleicker,T., Harakeh,S.M., Alzahrani,A.A., Alsaaidi,G.A., Alagili,A.N., Hashem,A.M., Zumla,A., Drosten,C., Azhar,E.I. | 2020-01-06T00:00:00Z | ssRNA(+) | 348 | S protein | Djibouti | Camelus dromedarius | |
| QFS19590.1 | El-Kafrawy,S.A., Corman,V.M., Tolah,A.M., Al Masaudi,S.B., Hassan,A.M., Muller,M.A., Bleicker,T., Harakeh,S.M., Alzahrani,A.A., Alsaaidi,G.A., Alagili,A.N., Hashem,A.M., Zumla,A., Drosten,C., Azhar,E.I. | 2020-01-06T00:00:00Z | ssRNA(+) | 348 | S protein | Djibouti | Camelus dromedarius | |
| QFS19591.1 | El-Kafrawy,S.A., Corman,V.M., Tolah,A.M., Al Masaudi,S.B., Hassan,A.M., Muller,M.A., Bleicker,T., Harakeh,S.M., Alzahrani,A.A., Alsaaidi,G.A., Alagili,A.N., Hashem,A.M., Zumla,A., Drosten,C., Azhar,E.I. | 2020-01-06T00:00:00Z | ssRNA(+) | 348 | S protein | Djibouti | Camelus dromedarius | |
| QFS19592.1 | El-Kafrawy,S.A., Corman,V.M., Tolah,A.M., Al Masaudi,S.B., Hassan,A.M., Muller,M.A., Bleicker,T., Harakeh,S.M., Alzahrani,A.A., Alsaaidi,G.A., Alagili,A.N., Hashem,A.M., Zumla,A., Drosten,C., Azhar,E.I. | 2020-01-06T00:00:00Z | ssRNA(+) | 348 | S protein | Djibouti | Camelus dromedarius | |
| QFS19593.1 | El-Kafrawy,S.A., Corman,V.M., Tolah,A.M., Al Masaudi,S.B., Hassan,A.M., Muller,M.A., Bleicker,T., Harakeh,S.M., Alzahrani,A.A., Alsaaidi,G.A., Alagili,A.N., Hashem,A.M., Zumla,A., Drosten,C., Azhar,E.I. | 2020-01-06T00:00:00Z | ssRNA(+) | 348 | S protein | Djibouti | Camelus dromedarius | |
| QFS19594.1 | El-Kafrawy,S.A., Corman,V.M., Tolah,A.M., Al Masaudi,S.B., Hassan,A.M., Muller,M.A., Bleicker,T., Harakeh,S.M., Alzahrani,A.A., Alsaaidi,G.A., Alagili,A.N., Hashem,A.M., Zumla,A., Drosten,C., Azhar,E.I. | 2020-01-06T00:00:00Z | ssRNA(+) | 348 | S protein | Djibouti | Camelus dromedarius | |
| QFS19595.1 | El-Kafrawy,S.A., Corman,V.M., Tolah,A.M., Al Masaudi,S.B., Hassan,A.M., Muller,M.A., Bleicker,T., Harakeh,S.M., Alzahrani,A.A., Alsaaidi,G.A., Alagili,A.N., Hashem,A.M., Zumla,A., Drosten,C., Azhar,E.I. | 2020-01-06T00:00:00Z | ssRNA(+) | 348 | S protein | Djibouti | Camelus dromedarius | |
| QFS19596.1 | El-Kafrawy,S.A., Corman,V.M., Tolah,A.M., Al Masaudi,S.B., Hassan,A.M., Muller,M.A., Bleicker,T., Harakeh,S.M., Alzahrani,A.A., Alsaaidi,G.A., Alagili,A.N., Hashem,A.M., Zumla,A., Drosten,C., Azhar,E.I. | 2020-01-06T00:00:00Z | ssRNA(+) | 348 | S protein | Djibouti | Camelus dromedarius | |
| QFS19597.1 | El-Kafrawy,S.A., Corman,V.M., Tolah,A.M., Al Masaudi,S.B., Hassan,A.M., Muller,M.A., Bleicker,T., Harakeh,S.M., Alzahrani,A.A., Alsaaidi,G.A., Alagili,A.N., Hashem,A.M., Zumla,A., Drosten,C., Azhar,E.I. | 2020-01-06T00:00:00Z | ssRNA(+) | 348 | S protein | Sudan | Camelus dromedarius | |
| QFS19598.1 | El-Kafrawy,S.A., Corman,V.M., Tolah,A.M., Al Masaudi,S.B., Hassan,A.M., Muller,M.A., Bleicker,T., Harakeh,S.M., Alzahrani,A.A., Alsaaidi,G.A., Alagili,A.N., Hashem,A.M., Zumla,A., Drosten,C., Azhar,E.I. | 2020-01-06T00:00:00Z | ssRNA(+) | 348 | S protein | Sudan | Camelus dromedarius | |
| QFS19599.1 | El-Kafrawy,S.A., Corman,V.M., Tolah,A.M., Al Masaudi,S.B., Hassan,A.M., Muller,M.A., Bleicker,T., Harakeh,S.M., Alzahrani,A.A., Alsaaidi,G.A., Alagili,A.N., Hashem,A.M., Zumla,A., Drosten,C., Azhar,E.I. | 2020-01-06T00:00:00Z | ssRNA(+) | 348 | S protein | Sudan | Camelus dromedarius | |
| QFS19600.1 | El-Kafrawy,S.A., Corman,V.M., Tolah,A.M., Al Masaudi,S.B., Hassan,A.M., Muller,M.A., Bleicker,T., Harakeh,S.M., Alzahrani,A.A., Alsaaidi,G.A., Alagili,A.N., Hashem,A.M., Zumla,A., Drosten,C., Azhar,E.I. | 2020-01-06T00:00:00Z | ssRNA(+) | 348 | S protein | Sudan | Camelus dromedarius | |
| QFS19601.1 | El-Kafrawy,S.A., Corman,V.M., Tolah,A.M., Al Masaudi,S.B., Hassan,A.M., Muller,M.A., Bleicker,T., Harakeh,S.M., Alzahrani,A.A., Alsaaidi,G.A., Alagili,A.N., Hashem,A.M., Zumla,A., Drosten,C., Azhar,E.I. | 2020-01-06T00:00:00Z | ssRNA(+) | 348 | S protein | Sudan | Camelus dromedarius | |
| QFS19602.1 | El-Kafrawy,S.A., Corman,V.M., Tolah,A.M., Al Masaudi,S.B., Hassan,A.M., Muller,M.A., Bleicker,T., Harakeh,S.M., Alzahrani,A.A., Alsaaidi,G.A., Alagili,A.N., Hashem,A.M., Zumla,A., Drosten,C., Azhar,E.I. | 2020-01-06T00:00:00Z | ssRNA(+) | 348 | S protein | Sudan | Camelus dromedarius | |
| QFS19603.1 | El-Kafrawy,S.A., Corman,V.M., Tolah,A.M., Al Masaudi,S.B., Hassan,A.M., Muller,M.A., Bleicker,T., Harakeh,S.M., Alzahrani,A.A., Alsaaidi,G.A., Alagili,A.N., Hashem,A.M., Zumla,A., Drosten,C., Azhar,E.I. | 2020-01-06T00:00:00Z | ssRNA(+) | 348 | S protein | Sudan | Camelus dromedarius | |
| QFS19604.1 | El-Kafrawy,S.A., Corman,V.M., Tolah,A.M., Al Masaudi,S.B., Hassan,A.M., Muller,M.A., Bleicker,T., Harakeh,S.M., Alzahrani,A.A., Alsaaidi,G.A., Alagili,A.N., Hashem,A.M., Zumla,A., Drosten,C., Azhar,E.I. | 2020-01-06T00:00:00Z | ssRNA(+) | 348 | S protein | Sudan | Camelus dromedarius | |
| QFS19605.1 | El-Kafrawy,S.A., Corman,V.M., Tolah,A.M., Al Masaudi,S.B., Hassan,A.M., Muller,M.A., Bleicker,T., Harakeh,S.M., Alzahrani,A.A., Alsaaidi,G.A., Alagili,A.N., Hashem,A.M., Zumla,A., Drosten,C., Azhar,E.I. | 2020-01-06T00:00:00Z | ssRNA(+) | 348 | S protein | Sudan | Camelus dromedarius | |
| QFS19606.1 | El-Kafrawy,S.A., Corman,V.M., Tolah,A.M., Al Masaudi,S.B., Hassan,A.M., Muller,M.A., Bleicker,T., Harakeh,S.M., Alzahrani,A.A., Alsaaidi,G.A., Alagili,A.N., Hashem,A.M., Zumla,A., Drosten,C., Azhar,E.I. | 2020-01-06T00:00:00Z | ssRNA(+) | 348 | S protein | Sudan | Camelus dromedarius | |
| QFS19607.1 | El-Kafrawy,S.A., Corman,V.M., Tolah,A.M., Al Masaudi,S.B., Hassan,A.M., Muller,M.A., Bleicker,T., Harakeh,S.M., Alzahrani,A.A., Alsaaidi,G.A., Alagili,A.N., Hashem,A.M., Zumla,A., Drosten,C., Azhar,E.I. | 2020-01-06T00:00:00Z | ssRNA(+) | 348 | S protein | Sudan | Camelus dromedarius | |
| QFS19608.1 | El-Kafrawy,S.A., Corman,V.M., Tolah,A.M., Al Masaudi,S.B., Hassan,A.M., Muller,M.A., Bleicker,T., Harakeh,S.M., Alzahrani,A.A., Alsaaidi,G.A., Alagili,A.N., Hashem,A.M., Zumla,A., Drosten,C., Azhar,E.I. | 2020-01-06T00:00:00Z | ssRNA(+) | 348 | S protein | Sudan | Camelus dromedarius | |
| QFS19609.1 | El-Kafrawy,S.A., Corman,V.M., Tolah,A.M., Al Masaudi,S.B., Hassan,A.M., Muller,M.A., Bleicker,T., Harakeh,S.M., Alzahrani,A.A., Alsaaidi,G.A., Alagili,A.N., Hashem,A.M., Zumla,A., Drosten,C., Azhar,E.I. | 2020-01-06T00:00:00Z | ssRNA(+) | 348 | S protein | Sudan | Camelus dromedarius | |
| QFS19610.1 | El-Kafrawy,S.A., Corman,V.M., Tolah,A.M., Al Masaudi,S.B., Hassan,A.M., Muller,M.A., Bleicker,T., Harakeh,S.M., Alzahrani,A.A., Alsaaidi,G.A., Alagili,A.N., Hashem,A.M., Zumla,A., Drosten,C., Azhar,E.I. | 2020-01-06T00:00:00Z | ssRNA(+) | 348 | S protein | Sudan | Camelus dromedarius | |
| QFS19611.1 | El-Kafrawy,S.A., Corman,V.M., Tolah,A.M., Al Masaudi,S.B., Hassan,A.M., Muller,M.A., Bleicker,T., Harakeh,S.M., Alzahrani,A.A., Alsaaidi,G.A., Alagili,A.N., Hashem,A.M., Zumla,A., Drosten,C., Azhar,E.I. | 2020-01-06T00:00:00Z | ssRNA(+) | 348 | S protein | Sudan | Camelus dromedarius | |
| QFS19612.1 | El-Kafrawy,S.A., Corman,V.M., Tolah,A.M., Al Masaudi,S.B., Hassan,A.M., Muller,M.A., Bleicker,T., Harakeh,S.M., Alzahrani,A.A., Alsaaidi,G.A., Alagili,A.N., Hashem,A.M., Zumla,A., Drosten,C., Azhar,E.I. | 2020-01-06T00:00:00Z | ssRNA(+) | 348 | S protein | Sudan | Camelus dromedarius | |
| QFS19613.1 | El-Kafrawy,S.A., Corman,V.M., Tolah,A.M., Al Masaudi,S.B., Hassan,A.M., Muller,M.A., Bleicker,T., Harakeh,S.M., Alzahrani,A.A., Alsaaidi,G.A., Alagili,A.N., Hashem,A.M., Zumla,A., Drosten,C., Azhar,E.I. | 2020-01-06T00:00:00Z | ssRNA(+) | 348 | S protein | Sudan | Camelus dromedarius | |
| QFS19614.1 | El-Kafrawy,S.A., Corman,V.M., Tolah,A.M., Al Masaudi,S.B., Hassan,A.M., Muller,M.A., Bleicker,T., Harakeh,S.M., Alzahrani,A.A., Alsaaidi,G.A., Alagili,A.N., Hashem,A.M., Zumla,A., Drosten,C., Azhar,E.I. | 2020-01-06T00:00:00Z | ssRNA(+) | 348 | S protein | Sudan | Camelus dromedarius | |
| QFS19615.1 | El-Kafrawy,S.A., Corman,V.M., Tolah,A.M., Al Masaudi,S.B., Hassan,A.M., Muller,M.A., Bleicker,T., Harakeh,S.M., Alzahrani,A.A., Alsaaidi,G.A., Alagili,A.N., Hashem,A.M., Zumla,A., Drosten,C., Azhar,E.I. | 2020-01-06T00:00:00Z | ssRNA(+) | 348 | S protein | Sudan | Camelus dromedarius | |
| QFS19616.1 | El-Kafrawy,S.A., Corman,V.M., Tolah,A.M., Al Masaudi,S.B., Hassan,A.M., Muller,M.A., Bleicker,T., Harakeh,S.M., Alzahrani,A.A., Alsaaidi,G.A., Alagili,A.N., Hashem,A.M., Zumla,A., Drosten,C., Azhar,E.I. | 2020-01-06T00:00:00Z | ssRNA(+) | 348 | S protein | Sudan | Camelus dromedarius | |
| QFS19617.1 | El-Kafrawy,S.A., Corman,V.M., Tolah,A.M., Al Masaudi,S.B., Hassan,A.M., Muller,M.A., Bleicker,T., Harakeh,S.M., Alzahrani,A.A., Alsaaidi,G.A., Alagili,A.N., Hashem,A.M., Zumla,A., Drosten,C., Azhar,E.I. | 2020-01-06T00:00:00Z | ssRNA(+) | 348 | S protein | Sudan | Camelus dromedarius | |
| QFS19618.1 | El-Kafrawy,S.A., Corman,V.M., Tolah,A.M., Al Masaudi,S.B., Hassan,A.M., Muller,M.A., Bleicker,T., Harakeh,S.M., Alzahrani,A.A., Alsaaidi,G.A., Alagili,A.N., Hashem,A.M., Zumla,A., Drosten,C., Azhar,E.I. | 2020-01-06T00:00:00Z | ssRNA(+) | 348 | S protein | Sudan | Camelus dromedarius | |
| QFS19619.1 | El-Kafrawy,S.A., Corman,V.M., Tolah,A.M., Al Masaudi,S.B., Hassan,A.M., Muller,M.A., Bleicker,T., Harakeh,S.M., Alzahrani,A.A., Alsaaidi,G.A., Alagili,A.N., Hashem,A.M., Zumla,A., Drosten,C., Azhar,E.I. | 2020-01-06T00:00:00Z | ssRNA(+) | 348 | S protein | Sudan | Camelus dromedarius | |
| QFS19620.1 | El-Kafrawy,S.A., Corman,V.M., Tolah,A.M., Al Masaudi,S.B., Hassan,A.M., Muller,M.A., Bleicker,T., Harakeh,S.M., Alzahrani,A.A., Alsaaidi,G.A., Alagili,A.N., Hashem,A.M., Zumla,A., Drosten,C., Azhar,E.I. | 2020-01-06T00:00:00Z | ssRNA(+) | 348 | S protein | Sudan | Camelus dromedarius | |
| QFS19621.1 | El-Kafrawy,S.A., Corman,V.M., Tolah,A.M., Al Masaudi,S.B., Hassan,A.M., Muller,M.A., Bleicker,T., Harakeh,S.M., Alzahrani,A.A., Alsaaidi,G.A., Alagili,A.N., Hashem,A.M., Zumla,A., Drosten,C., Azhar,E.I. | 2020-01-06T00:00:00Z | ssRNA(+) | 348 | S protein | Djibouti | Camelus dromedarius | |
| QFS19622.1 | El-Kafrawy,S.A., Corman,V.M., Tolah,A.M., Al Masaudi,S.B., Hassan,A.M., Muller,M.A., Bleicker,T., Harakeh,S.M., Alzahrani,A.A., Alsaaidi,G.A., Alagili,A.N., Hashem,A.M., Zumla,A., Drosten,C., Azhar,E.I. | 2020-01-06T00:00:00Z | ssRNA(+) | 348 | S protein | Sudan | Camelus dromedarius | |
| QFS19623.1 | El-Kafrawy,S.A., Corman,V.M., Tolah,A.M., Al Masaudi,S.B., Hassan,A.M., Muller,M.A., Bleicker,T., Harakeh,S.M., Alzahrani,A.A., Alsaaidi,G.A., Alagili,A.N., Hashem,A.M., Zumla,A., Drosten,C., Azhar,E.I. | 2020-01-06T00:00:00Z | ssRNA(+) | 348 | S protein | Sudan | Camelus dromedarius | |
| QFS19624.1 | El-Kafrawy,S.A., Corman,V.M., Tolah,A.M., Al Masaudi,S.B., Hassan,A.M., Muller,M.A., Bleicker,T., Harakeh,S.M., Alzahrani,A.A., Alsaaidi,G.A., Alagili,A.N., Hashem,A.M., Zumla,A., Drosten,C., Azhar,E.I. | 2020-01-06T00:00:00Z | ssRNA(+) | 348 | S protein | Djibouti | Camelus dromedarius | |
| QFS19625.1 | El-Kafrawy,S.A., Corman,V.M., Tolah,A.M., Al Masaudi,S.B., Hassan,A.M., Muller,M.A., Bleicker,T., Harakeh,S.M., Alzahrani,A.A., Alsaaidi,G.A., Alagili,A.N., Hashem,A.M., Zumla,A., Drosten,C., Azhar,E.I. | 2020-01-06T00:00:00Z | ssRNA(+) | 348 | S protein | Sudan | Camelus dromedarius | |
| QFS19626.1 | El-Kafrawy,S.A., Corman,V.M., Tolah,A.M., Al Masaudi,S.B., Hassan,A.M., Muller,M.A., Bleicker,T., Harakeh,S.M., Alzahrani,A.A., Alsaaidi,G.A., Alagili,A.N., Hashem,A.M., Zumla,A., Drosten,C., Azhar,E.I. | 2020-01-06T00:00:00Z | ssRNA(+) | 348 | S protein | Sudan | Camelus dromedarius | |
| QFS19627.1 | El-Kafrawy,S.A., Corman,V.M., Tolah,A.M., Al Masaudi,S.B., Hassan,A.M., Muller,M.A., Bleicker,T., Harakeh,S.M., Alzahrani,A.A., Alsaaidi,G.A., Alagili,A.N., Hashem,A.M., Zumla,A., Drosten,C., Azhar,E.I. | 2020-01-06T00:00:00Z | ssRNA(+) | 348 | S protein | Sudan | Camelus dromedarius | |
| QFS19628.1 | El-Kafrawy,S.A., Corman,V.M., Tolah,A.M., Al Masaudi,S.B., Hassan,A.M., Muller,M.A., Bleicker,T., Harakeh,S.M., Alzahrani,A.A., Alsaaidi,G.A., Alagili,A.N., Hashem,A.M., Zumla,A., Drosten,C., Azhar,E.I. | 2020-01-06T00:00:00Z | ssRNA(+) | 348 | S protein | Sudan | Camelus dromedarius | |
| QFS19629.1 | El-Kafrawy,S.A., Corman,V.M., Tolah,A.M., Al Masaudi,S.B., Hassan,A.M., Muller,M.A., Bleicker,T., Harakeh,S.M., Alzahrani,A.A., Alsaaidi,G.A., Alagili,A.N., Hashem,A.M., Zumla,A., Drosten,C., Azhar,E.I. | 2020-01-06T00:00:00Z | ssRNA(+) | 348 | S protein | Sudan | Camelus dromedarius | |
| QFS19630.1 | El-Kafrawy,S.A., Corman,V.M., Tolah,A.M., Al Masaudi,S.B., Hassan,A.M., Muller,M.A., Bleicker,T., Harakeh,S.M., Alzahrani,A.A., Alsaaidi,G.A., Alagili,A.N., Hashem,A.M., Zumla,A., Drosten,C., Azhar,E.I. | 2020-01-06T00:00:00Z | ssRNA(+) | 348 | S protein | Sudan | Camelus dromedarius | |
| QFS19631.1 | El-Kafrawy,S.A., Corman,V.M., Tolah,A.M., Al Masaudi,S.B., Hassan,A.M., Muller,M.A., Bleicker,T., Harakeh,S.M., Alzahrani,A.A., Alsaaidi,G.A., Alagili,A.N., Hashem,A.M., Zumla,A., Drosten,C., Azhar,E.I. | 2020-01-06T00:00:00Z | ssRNA(+) | 348 | S protein | Sudan | Camelus dromedarius | |
| QFS19632.1 | El-Kafrawy,S.A., Corman,V.M., Tolah,A.M., Al Masaudi,S.B., Hassan,A.M., Muller,M.A., Bleicker,T., Harakeh,S.M., Alzahrani,A.A., Alsaaidi,G.A., Alagili,A.N., Hashem,A.M., Zumla,A., Drosten,C., Azhar,E.I. | 2020-01-06T00:00:00Z | ssRNA(+) | 348 | S protein | Sudan | Camelus dromedarius | |
| QFS19633.1 | El-Kafrawy,S.A., Corman,V.M., Tolah,A.M., Al Masaudi,S.B., Hassan,A.M., Muller,M.A., Bleicker,T., Harakeh,S.M., Alzahrani,A.A., Alsaaidi,G.A., Alagili,A.N., Hashem,A.M., Zumla,A., Drosten,C., Azhar,E.I. | 2020-01-06T00:00:00Z | ssRNA(+) | 348 | S protein | Sudan | Camelus dromedarius | |
| QFS19634.1 | El-Kafrawy,S.A., Corman,V.M., Tolah,A.M., Al Masaudi,S.B., Hassan,A.M., Muller,M.A., Bleicker,T., Harakeh,S.M., Alzahrani,A.A., Alsaaidi,G.A., Alagili,A.N., Hashem,A.M., Zumla,A., Drosten,C., Azhar,E.I. | 2020-01-06T00:00:00Z | ssRNA(+) | 348 | S protein | Sudan | Camelus dromedarius | |
| QFS19635.1 | El-Kafrawy,S.A., Corman,V.M., Tolah,A.M., Al Masaudi,S.B., Hassan,A.M., Muller,M.A., Bleicker,T., Harakeh,S.M., Alzahrani,A.A., Alsaaidi,G.A., Alagili,A.N., Hashem,A.M., Zumla,A., Drosten,C., Azhar,E.I. | 2020-01-06T00:00:00Z | ssRNA(+) | 348 | S protein | Sudan | Camelus dromedarius | |
| QFS19636.1 | El-Kafrawy,S.A., Corman,V.M., Tolah,A.M., Al Masaudi,S.B., Hassan,A.M., Muller,M.A., Bleicker,T., Harakeh,S.M., Alzahrani,A.A., Alsaaidi,G.A., Alagili,A.N., Hashem,A.M., Zumla,A., Drosten,C., Azhar,E.I. | 2020-01-06T00:00:00Z | ssRNA(+) | 348 | S protein | Sudan | Camelus dromedarius | |
| QFS19637.1 | El-Kafrawy,S.A., Corman,V.M., Tolah,A.M., Al Masaudi,S.B., Hassan,A.M., Muller,M.A., Bleicker,T., Harakeh,S.M., Alzahrani,A.A., Alsaaidi,G.A., Alagili,A.N., Hashem,A.M., Zumla,A., Drosten,C., Azhar,E.I. | 2020-01-06T00:00:00Z | ssRNA(+) | 348 | S protein | Sudan | Camelus dromedarius | |
| QFS19638.1 | El-Kafrawy,S.A., Corman,V.M., Tolah,A.M., Al Masaudi,S.B., Hassan,A.M., Muller,M.A., Bleicker,T., Harakeh,S.M., Alzahrani,A.A., Alsaaidi,G.A., Alagili,A.N., Hashem,A.M., Zumla,A., Drosten,C., Azhar,E.I. | 2020-01-06T00:00:00Z | ssRNA(+) | 348 | S protein | Sudan | Camelus dromedarius | |
| QFS19639.1 | El-Kafrawy,S.A., Corman,V.M., Tolah,A.M., Al Masaudi,S.B., Hassan,A.M., Muller,M.A., Bleicker,T., Harakeh,S.M., Alzahrani,A.A., Alsaaidi,G.A., Alagili,A.N., Hashem,A.M., Zumla,A., Drosten,C., Azhar,E.I. | 2020-01-06T00:00:00Z | ssRNA(+) | 348 | S protein | Sudan | Camelus dromedarius | |
| QFS19640.1 | El-Kafrawy,S.A., Corman,V.M., Tolah,A.M., Al Masaudi,S.B., Hassan,A.M., Muller,M.A., Bleicker,T., Harakeh,S.M., Alzahrani,A.A., Alsaaidi,G.A., Alagili,A.N., Hashem,A.M., Zumla,A., Drosten,C., Azhar,E.I. | 2020-01-06T00:00:00Z | ssRNA(+) | 348 | S protein | Sudan | Camelus dromedarius | |
| QFS19641.1 | El-Kafrawy,S.A., Corman,V.M., Tolah,A.M., Al Masaudi,S.B., Hassan,A.M., Muller,M.A., Bleicker,T., Harakeh,S.M., Alzahrani,A.A., Alsaaidi,G.A., Alagili,A.N., Hashem,A.M., Zumla,A., Drosten,C., Azhar,E.I. | 2020-01-06T00:00:00Z | ssRNA(+) | 348 | S protein | Sudan | Camelus dromedarius | |
| QFS19642.1 | El-Kafrawy,S.A., Corman,V.M., Tolah,A.M., Al Masaudi,S.B., Hassan,A.M., Muller,M.A., Bleicker,T., Harakeh,S.M., Alzahrani,A.A., Alsaaidi,G.A., Alagili,A.N., Hashem,A.M., Zumla,A., Drosten,C., Azhar,E.I. | 2020-01-06T00:00:00Z | ssRNA(+) | 348 | S protein | Sudan | Camelus dromedarius | |
| QFS19643.1 | El-Kafrawy,S.A., Corman,V.M., Tolah,A.M., Al Masaudi,S.B., Hassan,A.M., Muller,M.A., Bleicker,T., Harakeh,S.M., Alzahrani,A.A., Alsaaidi,G.A., Alagili,A.N., Hashem,A.M., Zumla,A., Drosten,C., Azhar,E.I. | 2020-01-06T00:00:00Z | ssRNA(+) | 348 | S protein | Sudan | Camelus dromedarius | |
| QFS19644.1 | El-Kafrawy,S.A., Corman,V.M., Tolah,A.M., Al Masaudi,S.B., Hassan,A.M., Muller,M.A., Bleicker,T., Harakeh,S.M., Alzahrani,A.A., Alsaaidi,G.A., Alagili,A.N., Hashem,A.M., Zumla,A., Drosten,C., Azhar,E.I. | 2020-01-06T00:00:00Z | ssRNA(+) | 348 | S protein | Sudan | Camelus dromedarius | |
| QFS19645.1 | El-Kafrawy,S.A., Corman,V.M., Tolah,A.M., Al Masaudi,S.B., Hassan,A.M., Muller,M.A., Bleicker,T., Harakeh,S.M., Alzahrani,A.A., Alsaaidi,G.A., Alagili,A.N., Hashem,A.M., Zumla,A., Drosten,C., Azhar,E.I. | 2020-01-06T00:00:00Z | ssRNA(+) | 348 | S protein | Sudan | Camelus dromedarius | |
| QFS19646.1 | El-Kafrawy,S.A., Corman,V.M., Tolah,A.M., Al Masaudi,S.B., Hassan,A.M., Muller,M.A., Bleicker,T., Harakeh,S.M., Alzahrani,A.A., Alsaaidi,G.A., Alagili,A.N., Hashem,A.M., Zumla,A., Drosten,C., Azhar,E.I. | 2020-01-06T00:00:00Z | ssRNA(+) | 348 | S protein | Sudan | Camelus dromedarius | |
| QFS19647.1 | El-Kafrawy,S.A., Corman,V.M., Tolah,A.M., Al Masaudi,S.B., Hassan,A.M., Muller,M.A., Bleicker,T., Harakeh,S.M., Alzahrani,A.A., Alsaaidi,G.A., Alagili,A.N., Hashem,A.M., Zumla,A., Drosten,C., Azhar,E.I. | 2020-01-06T00:00:00Z | ssRNA(+) | 348 | S protein | Sudan | Camelus dromedarius | |
| QFS19648.1 | El-Kafrawy,S.A., Corman,V.M., Tolah,A.M., Al Masaudi,S.B., Hassan,A.M., Muller,M.A., Bleicker,T., Harakeh,S.M., Alzahrani,A.A., Alsaaidi,G.A., Alagili,A.N., Hashem,A.M., Zumla,A., Drosten,C., Azhar,E.I. | 2020-01-06T00:00:00Z | ssRNA(+) | 348 | S protein | Sudan | Camelus dromedarius | |
| QFS19649.1 | El-Kafrawy,S.A., Corman,V.M., Tolah,A.M., Al Masaudi,S.B., Hassan,A.M., Muller,M.A., Bleicker,T., Harakeh,S.M., Alzahrani,A.A., Alsaaidi,G.A., Alagili,A.N., Hashem,A.M., Zumla,A., Drosten,C., Azhar,E.I. | 2020-01-06T00:00:00Z | ssRNA(+) | 348 | S protein | Sudan | Camelus dromedarius | |
| QFS19650.1 | El-Kafrawy,S.A., Corman,V.M., Tolah,A.M., Al Masaudi,S.B., Hassan,A.M., Muller,M.A., Bleicker,T., Harakeh,S.M., Alzahrani,A.A., Alsaaidi,G.A., Alagili,A.N., Hashem,A.M., Zumla,A., Drosten,C., Azhar,E.I. | 2020-01-06T00:00:00Z | ssRNA(+) | 348 | S protein | Sudan | Camelus dromedarius | |
| QFS19651.1 | El-Kafrawy,S.A., Corman,V.M., Tolah,A.M., Al Masaudi,S.B., Hassan,A.M., Muller,M.A., Bleicker,T., Harakeh,S.M., Alzahrani,A.A., Alsaaidi,G.A., Alagili,A.N., Hashem,A.M., Zumla,A., Drosten,C., Azhar,E.I. | 2020-01-06T00:00:00Z | ssRNA(+) | 348 | S protein | Sudan | Camelus dromedarius | |
| QFS19652.1 | El-Kafrawy,S.A., Corman,V.M., Tolah,A.M., Al Masaudi,S.B., Hassan,A.M., Muller,M.A., Bleicker,T., Harakeh,S.M., Alzahrani,A.A., Alsaaidi,G.A., Alagili,A.N., Hashem,A.M., Zumla,A., Drosten,C., Azhar,E.I. | 2020-01-06T00:00:00Z | ssRNA(+) | 348 | S protein | Sudan | Camelus dromedarius | |
| QFS19653.1 | El-Kafrawy,S.A., Corman,V.M., Tolah,A.M., Al Masaudi,S.B., Hassan,A.M., Muller,M.A., Bleicker,T., Harakeh,S.M., Alzahrani,A.A., Alsaaidi,G.A., Alagili,A.N., Hashem,A.M., Zumla,A., Drosten,C., Azhar,E.I. | 2020-01-06T00:00:00Z | ssRNA(+) | 348 | S protein | Sudan | Camelus dromedarius | |
| QFS19654.1 | El-Kafrawy,S.A., Corman,V.M., Tolah,A.M., Al Masaudi,S.B., Hassan,A.M., Muller,M.A., Bleicker,T., Harakeh,S.M., Alzahrani,A.A., Alsaaidi,G.A., Alagili,A.N., Hashem,A.M., Zumla,A., Drosten,C., Azhar,E.I. | 2020-01-06T00:00:00Z | ssRNA(+) | 348 | S protein | Sudan | Camelus dromedarius | |
| QFS19655.1 | El-Kafrawy,S.A., Corman,V.M., Tolah,A.M., Al Masaudi,S.B., Hassan,A.M., Muller,M.A., Bleicker,T., Harakeh,S.M., Alzahrani,A.A., Alsaaidi,G.A., Alagili,A.N., Hashem,A.M., Zumla,A., Drosten,C., Azhar,E.I. | 2020-01-06T00:00:00Z | ssRNA(+) | 348 | S protein | Sudan | Camelus dromedarius | |
| QFS19656.1 | El-Kafrawy,S.A., Corman,V.M., Tolah,A.M., Al Masaudi,S.B., Hassan,A.M., Muller,M.A., Bleicker,T., Harakeh,S.M., Alzahrani,A.A., Alsaaidi,G.A., Alagili,A.N., Hashem,A.M., Zumla,A., Drosten,C., Azhar,E.I. | 2020-01-06T00:00:00Z | ssRNA(+) | 348 | S protein | Sudan | Camelus dromedarius | |
| QFS19657.1 | El-Kafrawy,S.A., Corman,V.M., Tolah,A.M., Al Masaudi,S.B., Hassan,A.M., Muller,M.A., Bleicker,T., Harakeh,S.M., Alzahrani,A.A., Alsaaidi,G.A., Alagili,A.N., Hashem,A.M., Zumla,A., Drosten,C., Azhar,E.I. | 2020-01-06T00:00:00Z | ssRNA(+) | 348 | S protein | Sudan | Camelus dromedarius | |
| QFS19658.1 | El-Kafrawy,S.A., Corman,V.M., Tolah,A.M., Al Masaudi,S.B., Hassan,A.M., Muller,M.A., Bleicker,T., Harakeh,S.M., Alzahrani,A.A., Alsaaidi,G.A., Alagili,A.N., Hashem,A.M., Zumla,A., Drosten,C., Azhar,E.I. | 2020-01-06T00:00:00Z | ssRNA(+) | 348 | S protein | Sudan | Camelus dromedarius | |
| QFS19659.1 | El-Kafrawy,S.A., Corman,V.M., Tolah,A.M., Al Masaudi,S.B., Hassan,A.M., Muller,M.A., Bleicker,T., Harakeh,S.M., Alzahrani,A.A., Alsaaidi,G.A., Alagili,A.N., Hashem,A.M., Zumla,A., Drosten,C., Azhar,E.I. | 2020-01-06T00:00:00Z | ssRNA(+) | 348 | S protein | Sudan | Camelus dromedarius | |
| QFS19660.1 | El-Kafrawy,S.A., Corman,V.M., Tolah,A.M., Al Masaudi,S.B., Hassan,A.M., Muller,M.A., Bleicker,T., Harakeh,S.M., Alzahrani,A.A., Alsaaidi,G.A., Alagili,A.N., Hashem,A.M., Zumla,A., Drosten,C., Azhar,E.I. | 2020-01-06T00:00:00Z | ssRNA(+) | 348 | S protein | Sudan | Camelus dromedarius | |
| QFS19661.1 | El-Kafrawy,S.A., Corman,V.M., Tolah,A.M., Al Masaudi,S.B., Hassan,A.M., Muller,M.A., Bleicker,T., Harakeh,S.M., Alzahrani,A.A., Alsaaidi,G.A., Alagili,A.N., Hashem,A.M., Zumla,A., Drosten,C., Azhar,E.I. | 2020-01-06T00:00:00Z | ssRNA(+) | 348 | S protein | Sudan | Camelus dromedarius | |
| QFS19662.1 | El-Kafrawy,S.A., Corman,V.M., Tolah,A.M., Al Masaudi,S.B., Hassan,A.M., Muller,M.A., Bleicker,T., Harakeh,S.M., Alzahrani,A.A., Alsaaidi,G.A., Alagili,A.N., Hashem,A.M., Zumla,A., Drosten,C., Azhar,E.I. | 2020-01-06T00:00:00Z | ssRNA(+) | 348 | S protein | Sudan | Camelus dromedarius | |
| QFS19663.1 | El-Kafrawy,S.A., Corman,V.M., Tolah,A.M., Al Masaudi,S.B., Hassan,A.M., Muller,M.A., Bleicker,T., Harakeh,S.M., Alzahrani,A.A., Alsaaidi,G.A., Alagili,A.N., Hashem,A.M., Zumla,A., Drosten,C., Azhar,E.I. | 2020-01-06T00:00:00Z | ssRNA(+) | 348 | S protein | Sudan | Camelus dromedarius | |
| QFS19664.1 | El-Kafrawy,S.A., Corman,V.M., Tolah,A.M., Al Masaudi,S.B., Hassan,A.M., Muller,M.A., Bleicker,T., Harakeh,S.M., Alzahrani,A.A., Alsaaidi,G.A., Alagili,A.N., Hashem,A.M., Zumla,A., Drosten,C., Azhar,E.I. | 2020-01-06T00:00:00Z | ssRNA(+) | 348 | S protein | Sudan | Camelus dromedarius | |
| QFS19665.1 | El-Kafrawy,S.A., Corman,V.M., Tolah,A.M., Al Masaudi,S.B., Hassan,A.M., Muller,M.A., Bleicker,T., Harakeh,S.M., Alzahrani,A.A., Alsaaidi,G.A., Alagili,A.N., Hashem,A.M., Zumla,A., Drosten,C., Azhar,E.I. | 2020-01-06T00:00:00Z | ssRNA(+) | 348 | S protein | Sudan | Camelus dromedarius | |
| QFS19666.1 | El-Kafrawy,S.A., Corman,V.M., Tolah,A.M., Al Masaudi,S.B., Hassan,A.M., Muller,M.A., Bleicker,T., Harakeh,S.M., Alzahrani,A.A., Alsaaidi,G.A., Alagili,A.N., Hashem,A.M., Zumla,A., Drosten,C., Azhar,E.I. | 2020-01-06T00:00:00Z | ssRNA(+) | 348 | S protein | Sudan | Camelus dromedarius | |
| QFS19667.1 | El-Kafrawy,S.A., Corman,V.M., Tolah,A.M., Al Masaudi,S.B., Hassan,A.M., Muller,M.A., Bleicker,T., Harakeh,S.M., Alzahrani,A.A., Alsaaidi,G.A., Alagili,A.N., Hashem,A.M., Zumla,A., Drosten,C., Azhar,E.I. | 2020-01-06T00:00:00Z | ssRNA(+) | 348 | S protein | Sudan | Camelus dromedarius | |
| QFS19668.1 | El-Kafrawy,S.A., Corman,V.M., Tolah,A.M., Al Masaudi,S.B., Hassan,A.M., Muller,M.A., Bleicker,T., Harakeh,S.M., Alzahrani,A.A., Alsaaidi,G.A., Alagili,A.N., Hashem,A.M., Zumla,A., Drosten,C., Azhar,E.I. | 2020-01-06T00:00:00Z | ssRNA(+) | 348 | S protein | Sudan | Camelus dromedarius | |
| QFS19669.1 | El-Kafrawy,S.A., Corman,V.M., Tolah,A.M., Al Masaudi,S.B., Hassan,A.M., Muller,M.A., Bleicker,T., Harakeh,S.M., Alzahrani,A.A., Alsaaidi,G.A., Alagili,A.N., Hashem,A.M., Zumla,A., Drosten,C., Azhar,E.I. | 2020-01-06T00:00:00Z | ssRNA(+) | 348 | S protein | Sudan | Camelus dromedarius | |
| QFS19670.1 | El-Kafrawy,S.A., Corman,V.M., Tolah,A.M., Al Masaudi,S.B., Hassan,A.M., Muller,M.A., Bleicker,T., Harakeh,S.M., Alzahrani,A.A., Alsaaidi,G.A., Alagili,A.N., Hashem,A.M., Zumla,A., Drosten,C., Azhar,E.I. | 2020-01-06T00:00:00Z | ssRNA(+) | 348 | S protein | Sudan | Camelus dromedarius | |
| QFS19671.1 | El-Kafrawy,S.A., Corman,V.M., Tolah,A.M., Al Masaudi,S.B., Hassan,A.M., Muller,M.A., Bleicker,T., Harakeh,S.M., Alzahrani,A.A., Alsaaidi,G.A., Alagili,A.N., Hashem,A.M., Zumla,A., Drosten,C., Azhar,E.I. | 2020-01-06T00:00:00Z | ssRNA(+) | 348 | S protein | Sudan | Camelus dromedarius | |
| QFS19672.1 | El-Kafrawy,S.A., Corman,V.M., Tolah,A.M., Al Masaudi,S.B., Hassan,A.M., Muller,M.A., Bleicker,T., Harakeh,S.M., Alzahrani,A.A., Alsaaidi,G.A., Alagili,A.N., Hashem,A.M., Zumla,A., Drosten,C., Azhar,E.I. | 2020-01-06T00:00:00Z | ssRNA(+) | 348 | S protein | Sudan | Camelus dromedarius | |
| QFS19673.1 | El-Kafrawy,S.A., Corman,V.M., Tolah,A.M., Al Masaudi,S.B., Hassan,A.M., Muller,M.A., Bleicker,T., Harakeh,S.M., Alzahrani,A.A., Alsaaidi,G.A., Alagili,A.N., Hashem,A.M., Zumla,A., Drosten,C., Azhar,E.I. | 2020-01-06T00:00:00Z | ssRNA(+) | 348 | S protein | Sudan | Camelus dromedarius | |
| QFS19674.1 | El-Kafrawy,S.A., Corman,V.M., Tolah,A.M., Al Masaudi,S.B., Hassan,A.M., Muller,M.A., Bleicker,T., Harakeh,S.M., Alzahrani,A.A., Alsaaidi,G.A., Alagili,A.N., Hashem,A.M., Zumla,A., Drosten,C., Azhar,E.I. | 2020-01-06T00:00:00Z | ssRNA(+) | 348 | S protein | Sudan | Camelus dromedarius | |
| QFS19675.1 | El-Kafrawy,S.A., Corman,V.M., Tolah,A.M., Al Masaudi,S.B., Hassan,A.M., Muller,M.A., Bleicker,T., Harakeh,S.M., Alzahrani,A.A., Alsaaidi,G.A., Alagili,A.N., Hashem,A.M., Zumla,A., Drosten,C., Azhar,E.I. | 2020-01-06T00:00:00Z | ssRNA(+) | 348 | S protein | Sudan | Camelus dromedarius | |
| QFS19676.1 | El-Kafrawy,S.A., Corman,V.M., Tolah,A.M., Al Masaudi,S.B., Hassan,A.M., Muller,M.A., Bleicker,T., Harakeh,S.M., Alzahrani,A.A., Alsaaidi,G.A., Alagili,A.N., Hashem,A.M., Zumla,A., Drosten,C., Azhar,E.I. | 2020-01-06T00:00:00Z | ssRNA(+) | 348 | S protein | Sudan | Camelus dromedarius | |
| QFS19677.1 | El-Kafrawy,S.A., Corman,V.M., Tolah,A.M., Al Masaudi,S.B., Hassan,A.M., Muller,M.A., Bleicker,T., Harakeh,S.M., Alzahrani,A.A., Alsaaidi,G.A., Alagili,A.N., Hashem,A.M., Zumla,A., Drosten,C., Azhar,E.I. | 2020-01-06T00:00:00Z | ssRNA(+) | 348 | S protein | Sudan | Camelus dromedarius | |
| QFS19678.1 | El-Kafrawy,S.A., Corman,V.M., Tolah,A.M., Al Masaudi,S.B., Hassan,A.M., Muller,M.A., Bleicker,T., Harakeh,S.M., Alzahrani,A.A., Alsaaidi,G.A., Alagili,A.N., Hashem,A.M., Zumla,A., Drosten,C., Azhar,E.I. | 2020-01-06T00:00:00Z | ssRNA(+) | 348 | S protein | Sudan | Camelus dromedarius | |
| QFS19679.1 | El-Kafrawy,S.A., Corman,V.M., Tolah,A.M., Al Masaudi,S.B., Hassan,A.M., Muller,M.A., Bleicker,T., Harakeh,S.M., Alzahrani,A.A., Alsaaidi,G.A., Alagili,A.N., Hashem,A.M., Zumla,A., Drosten,C., Azhar,E.I. | 2020-01-06T00:00:00Z | ssRNA(+) | 348 | S protein | Sudan | Camelus dromedarius | |
| QFS19680.1 | El-Kafrawy,S.A., Corman,V.M., Tolah,A.M., Al Masaudi,S.B., Hassan,A.M., Muller,M.A., Bleicker,T., Harakeh,S.M., Alzahrani,A.A., Alsaaidi,G.A., Alagili,A.N., Hashem,A.M., Zumla,A., Drosten,C., Azhar,E.I. | 2020-01-06T00:00:00Z | ssRNA(+) | 348 | S protein | Sudan | Camelus dromedarius | |
| QFS19681.1 | El-Kafrawy,S.A., Corman,V.M., Tolah,A.M., Al Masaudi,S.B., Hassan,A.M., Muller,M.A., Bleicker,T., Harakeh,S.M., Alzahrani,A.A., Alsaaidi,G.A., Alagili,A.N., Hashem,A.M., Zumla,A., Drosten,C., Azhar,E.I. | 2020-01-06T00:00:00Z | ssRNA(+) | 348 | S protein | Sudan | Camelus dromedarius | |
| QFS19682.1 | El-Kafrawy,S.A., Corman,V.M., Tolah,A.M., Al Masaudi,S.B., Hassan,A.M., Muller,M.A., Bleicker,T., Harakeh,S.M., Alzahrani,A.A., Alsaaidi,G.A., Alagili,A.N., Hashem,A.M., Zumla,A., Drosten,C., Azhar,E.I. | 2020-01-06T00:00:00Z | ssRNA(+) | 348 | S protein | Sudan | Camelus dromedarius | |
| QFS19683.1 | El-Kafrawy,S.A., Corman,V.M., Tolah,A.M., Al Masaudi,S.B., Hassan,A.M., Muller,M.A., Bleicker,T., Harakeh,S.M., Alzahrani,A.A., Alsaaidi,G.A., Alagili,A.N., Hashem,A.M., Zumla,A., Drosten,C., Azhar,E.I. | 2020-01-06T00:00:00Z | ssRNA(+) | 348 | S protein | Sudan | Camelus dromedarius | |
| QFS19684.1 | El-Kafrawy,S.A., Corman,V.M., Tolah,A.M., Al Masaudi,S.B., Hassan,A.M., Muller,M.A., Bleicker,T., Harakeh,S.M., Alzahrani,A.A., Alsaaidi,G.A., Alagili,A.N., Hashem,A.M., Zumla,A., Drosten,C., Azhar,E.I. | 2020-01-06T00:00:00Z | ssRNA(+) | 348 | S protein | Sudan | Camelus dromedarius | |
| QFS19685.1 | El-Kafrawy,S.A., Corman,V.M., Tolah,A.M., Al Masaudi,S.B., Hassan,A.M., Muller,M.A., Bleicker,T., Harakeh,S.M., Alzahrani,A.A., Alsaaidi,G.A., Alagili,A.N., Hashem,A.M., Zumla,A., Drosten,C., Azhar,E.I. | 2020-01-06T00:00:00Z | ssRNA(+) | 348 | S protein | Sudan | Camelus dromedarius | |
| QFS19686.1 | El-Kafrawy,S.A., Corman,V.M., Tolah,A.M., Al Masaudi,S.B., Hassan,A.M., Muller,M.A., Bleicker,T., Harakeh,S.M., Alzahrani,A.A., Alsaaidi,G.A., Alagili,A.N., Hashem,A.M., Zumla,A., Drosten,C., Azhar,E.I. | 2020-01-06T00:00:00Z | ssRNA(+) | 348 | S protein | Sudan | Camelus dromedarius | |
| QDI73610.1 | Chu,D.K., Poon,L.L., Gomaa,M.M., Shehata,M.M., Perera,R.A., Abu Zeid,D., El Rifay,A.S., Siu,L.Y., Guan,Y., Webby,R.J., Ali,M.A., Peiris,M., Kayali,G., Chan,R.W., Hemida,M.G., Alnaeem,A., Tao,K.P., Ng,H.Y., Chan,M.C., Nicholls,J.M., Peiris,J.S., Chu,D.K.W., Poon,L.L.M., Ali,M., Peiris,M.J.S. | 2019-07-08T00:00:00Z | ssRNA(+) | 1353 | S protein | Egypt | Camelus dromedarius | |
| AXP07345.1 | Ommeh,S., Zhang,W., Zohaib,A., Chen,J., Zhang,H., Hu,B., Ge,X.Y., Yang,X.L., Masika,M., Obanda,V., Luo,Y., Li,S., Waruhiu,C., Li,B., Zhu,Y., Ouma,D., Odendo,V., Wang,L.F., Anderson,D.E., Lichoti,J., Mungube,E., Gakuya,F., Zhou,P., Ngeiywa,K.J., Yan,B., Agwanda,B., Shi,Z.L., Ge,X.-Y., Yang,X.-L., Wang,L.-F., Ngeiywa,K.-J., Shi,Z.-L. | 2019-01-03T00:00:00Z | ssRNA(+) | 1353 | S protein | Kenya | Camelus dromedarius | |
| AXP07355.1 | Ommeh,S., Zhang,W., Zohaib,A., Chen,J., Zhang,H., Hu,B., Ge,X.Y., Yang,X.L., Masika,M., Obanda,V., Luo,Y., Li,S., Waruhiu,C., Li,B., Zhu,Y., Ouma,D., Odendo,V., Wang,L.F., Anderson,D.E., Lichoti,J., Mungube,E., Gakuya,F., Zhou,P., Ngeiywa,K.J., Yan,B., Agwanda,B., Shi,Z.L., Ge,X.-Y., Yang,X.-L., Wang,L.-F., Ngeiywa,K.-J., Shi,Z.-L. | 2019-01-03T00:00:00Z | ssRNA(+) | 1353 | S protein | Kenya | Camelus dromedarius | |
| AVV62526.1 | Luo,C.M., Wang,N., Yang,X.L., Liu,H.Z., Zhang,W., Li,B., Hu,B., Peng,C., Geng,Q.B., Zhu,G.J., Li,F., Shi,Z.L., Luo,C.-M., Geng,Q.-B., Yang,X.-L., Liu,H.-Z., Ge,X.-Y., Shi,Z.-L. | 2018-04-10T00:00:00Z | ssRNA(+) | 1356 | S protein | China |  | |
| AVV62537.1 | Luo,C.M., Wang,N., Yang,X.L., Liu,H.Z., Zhang,W., Li,B., Hu,B., Peng,C., Geng,Q.B., Zhu,G.J., Li,F., Shi,Z.L., Luo,C.-M., Geng,Q.-B., Yang,X.-L., Liu,H.-Z., Ge,X.-Y., Shi,Z.-L. | 2018-04-10T00:00:00Z | ssRNA(+) | 1349 | S protein | China |  | |
| AVN89280.1 | Chu,D.K.W., Hui,K.P.Y., Perera,R.A.P.M., Miguel,E., Niemeyer,D., Zhao,J., Channappanavar,R., Dudas,G., Oladipo,J.O., Traore,A., Fassi-Fihri,O., Ali,A., Demissie,G.F., Muth,D., Chan,M.C.W., Nicholls,J.M., Meyerholz,D.K., Kuranga,S.A., Mamo,G., Zhou,Z., So,R.T.Y., Hemida,M.G., Webby,R.J., Roger,F., Rambaut,A., Poon,L.L.M., Perlman,S., Drosten,C., Chevalier,V., Peiris,M. | 2018-03-20T00:00:00Z | ssRNA(+) | 178 | S protein | Ethiopia | Camelus dromedarius | |
| AVN89291.1 | Chu,D.K.W., Hui,K.P.Y., Perera,R.A.P.M., Miguel,E., Niemeyer,D., Zhao,J., Channappanavar,R., Dudas,G., Oladipo,J.O., Traore,A., Fassi-Fihri,O., Ali,A., Demissie,G.F., Muth,D., Chan,M.C.W., Nicholls,J.M., Meyerholz,D.K., Kuranga,S.A., Mamo,G., Zhou,Z., So,R.T.Y., Hemida,M.G., Webby,R.J., Roger,F., Rambaut,A., Poon,L.L.M., Perlman,S., Drosten,C., Chevalier,V., Peiris,M. | 2018-03-20T00:00:00Z | ssRNA(+) | 1353 | S protein | Ethiopia | Camelus dromedarius | |
| AVN89302.1 | Chu,D.K.W., Hui,K.P.Y., Perera,R.A.P.M., Miguel,E., Niemeyer,D., Zhao,J., Channappanavar,R., Dudas,G., Oladipo,J.O., Traore,A., Fassi-Fihri,O., Ali,A., Demissie,G.F., Muth,D., Chan,M.C.W., Nicholls,J.M., Meyerholz,D.K., Kuranga,S.A., Mamo,G., Zhou,Z., So,R.T.Y., Hemida,M.G., Webby,R.J., Roger,F., Rambaut,A., Poon,L.L.M., Perlman,S., Drosten,C., Chevalier,V., Peiris,M. | 2018-03-20T00:00:00Z | ssRNA(+) | 1353 | S protein | Ethiopia | Camelus dromedarius | |
| AVN89313.1 | Chu,D.K.W., Hui,K.P.Y., Perera,R.A.P.M., Miguel,E., Niemeyer,D., Zhao,J., Channappanavar,R., Dudas,G., Oladipo,J.O., Traore,A., Fassi-Fihri,O., Ali,A., Demissie,G.F., Muth,D., Chan,M.C.W., Nicholls,J.M., Meyerholz,D.K., Kuranga,S.A., Mamo,G., Zhou,Z., So,R.T.Y., Hemida,M.G., Webby,R.J., Roger,F., Rambaut,A., Poon,L.L.M., Perlman,S., Drosten,C., Chevalier,V., Peiris,M. | 2018-03-20T00:00:00Z | ssRNA(+) | 1353 | S protein | Ethiopia | Camelus dromedarius | |
| AVN89324.1 | Chu,D.K.W., Hui,K.P.Y., Perera,R.A.P.M., Miguel,E., Niemeyer,D., Zhao,J., Channappanavar,R., Dudas,G., Oladipo,J.O., Traore,A., Fassi-Fihri,O., Ali,A., Demissie,G.F., Muth,D., Chan,M.C.W., Nicholls,J.M., Meyerholz,D.K., Kuranga,S.A., Mamo,G., Zhou,Z., So,R.T.Y., Hemida,M.G., Webby,R.J., Roger,F., Rambaut,A., Poon,L.L.M., Perlman,S., Drosten,C., Chevalier,V., Peiris,M. | 2018-03-20T00:00:00Z | ssRNA(+) | 1353 | S protein | Morocco | Camelus dromedarius | |
| AVN89334.1 | Chu,D.K.W., Hui,K.P.Y., Perera,R.A.P.M., Miguel,E., Niemeyer,D., Zhao,J., Channappanavar,R., Dudas,G., Oladipo,J.O., Traore,A., Fassi-Fihri,O., Ali,A., Demissie,G.F., Muth,D., Chan,M.C.W., Nicholls,J.M., Meyerholz,D.K., Kuranga,S.A., Mamo,G., Zhou,Z., So,R.T.Y., Hemida,M.G., Webby,R.J., Roger,F., Rambaut,A., Poon,L.L.M., Perlman,S., Drosten,C., Chevalier,V., Peiris,M. | 2018-03-20T00:00:00Z | ssRNA(+) | 1353 | S protein | Burkina Faso | Camelus dromedarius | |
| AVN89344.1 | Chu,D.K.W., Hui,K.P.Y., Perera,R.A.P.M., Miguel,E., Niemeyer,D., Zhao,J., Channappanavar,R., Dudas,G., Oladipo,J.O., Traore,A., Fassi-Fihri,O., Ali,A., Demissie,G.F., Muth,D., Chan,M.C.W., Nicholls,J.M., Meyerholz,D.K., Kuranga,S.A., Mamo,G., Zhou,Z., So,R.T.Y., Hemida,M.G., Webby,R.J., Roger,F., Rambaut,A., Poon,L.L.M., Perlman,S., Drosten,C., Chevalier,V., Peiris,M. | 2018-03-20T00:00:00Z | ssRNA(+) | 1353 | S protein | Burkina Faso | Camelus dromedarius | |
| AVN89354.1 | Chu,D.K.W., Hui,K.P.Y., Perera,R.A.P.M., Miguel,E., Niemeyer,D., Zhao,J., Channappanavar,R., Dudas,G., Oladipo,J.O., Traore,A., Fassi-Fihri,O., Ali,A., Demissie,G.F., Muth,D., Chan,M.C.W., Nicholls,J.M., Meyerholz,D.K., Kuranga,S.A., Mamo,G., Zhou,Z., So,R.T.Y., Hemida,M.G., Webby,R.J., Roger,F., Rambaut,A., Poon,L.L.M., Perlman,S., Drosten,C., Chevalier,V., Peiris,M. | 2018-03-20T00:00:00Z | ssRNA(+) | 1353 | S protein | Nigeria | Camelus dromedarius | |
| AVN89365.1 | Chu,D.K.W., Hui,K.P.Y., Perera,R.A.P.M., Miguel,E., Niemeyer,D., Zhao,J., Channappanavar,R., Dudas,G., Oladipo,J.O., Traore,A., Fassi-Fihri,O., Ali,A., Demissie,G.F., Muth,D., Chan,M.C.W., Nicholls,J.M., Meyerholz,D.K., Kuranga,S.A., Mamo,G., Zhou,Z., So,R.T.Y., Hemida,M.G., Webby,R.J., Roger,F., Rambaut,A., Poon,L.L.M., Perlman,S., Drosten,C., Chevalier,V., Peiris,M. | 2018-03-20T00:00:00Z | ssRNA(+) | 1353 | S protein | Burkina Faso | Camelus dromedarius | |
| AVN89376.1 | Chu,D.K.W., Hui,K.P.Y., Perera,R.A.P.M., Miguel,E., Niemeyer,D., Zhao,J., Channappanavar,R., Dudas,G., Oladipo,J.O., Traore,A., Fassi-Fihri,O., Ali,A., Demissie,G.F., Muth,D., Chan,M.C.W., Nicholls,J.M., Meyerholz,D.K., Kuranga,S.A., Mamo,G., Zhou,Z., So,R.T.Y., Hemida,M.G., Webby,R.J., Roger,F., Rambaut,A., Poon,L.L.M., Perlman,S., Drosten,C., Chevalier,V., Peiris,M. | 2018-03-20T00:00:00Z | ssRNA(+) | 1353 | S protein | Nigeria | Camelus dromedarius | |
| AVN89387.1 | Chu,D.K.W., Hui,K.P.Y., Perera,R.A.P.M., Miguel,E., Niemeyer,D., Zhao,J., Channappanavar,R., Dudas,G., Oladipo,J.O., Traore,A., Fassi-Fihri,O., Ali,A., Demissie,G.F., Muth,D., Chan,M.C.W., Nicholls,J.M., Meyerholz,D.K., Kuranga,S.A., Mamo,G., Zhou,Z., So,R.T.Y., Hemida,M.G., Webby,R.J., Roger,F., Rambaut,A., Poon,L.L.M., Perlman,S., Drosten,C., Chevalier,V., Peiris,M. | 2018-03-20T00:00:00Z | ssRNA(+) | 1353 | S protein | Nigeria | Camelus dromedarius | |
| AVN89398.1 | Chu,D.K.W., Hui,K.P.Y., Perera,R.A.P.M., Miguel,E., Niemeyer,D., Zhao,J., Channappanavar,R., Dudas,G., Oladipo,J.O., Traore,A., Fassi-Fihri,O., Ali,A., Demissie,G.F., Muth,D., Chan,M.C.W., Nicholls,J.M., Meyerholz,D.K., Kuranga,S.A., Mamo,G., Zhou,Z., So,R.T.Y., Hemida,M.G., Webby,R.J., Roger,F., Rambaut,A., Poon,L.L.M., Perlman,S., Drosten,C., Chevalier,V., Peiris,M. | 2018-03-20T00:00:00Z | ssRNA(+) | 1353 | S protein | Nigeria | Camelus dromedarius | |
| AVN89409.1 | Chu,D.K.W., Hui,K.P.Y., Perera,R.A.P.M., Miguel,E., Niemeyer,D., Zhao,J., Channappanavar,R., Dudas,G., Oladipo,J.O., Traore,A., Fassi-Fihri,O., Ali,A., Demissie,G.F., Muth,D., Chan,M.C.W., Nicholls,J.M., Meyerholz,D.K., Kuranga,S.A., Mamo,G., Zhou,Z., So,R.T.Y., Hemida,M.G., Webby,R.J., Roger,F., Rambaut,A., Poon,L.L.M., Perlman,S., Drosten,C., Chevalier,V., Peiris,M. | 2018-03-20T00:00:00Z | ssRNA(+) | 1353 | S protein | Nigeria | Camelus dromedarius | |
| AVN89420.1 | Chu,D.K.W., Hui,K.P.Y., Perera,R.A.P.M., Miguel,E., Niemeyer,D., Zhao,J., Channappanavar,R., Dudas,G., Oladipo,J.O., Traore,A., Fassi-Fihri,O., Ali,A., Demissie,G.F., Muth,D., Chan,M.C.W., Nicholls,J.M., Meyerholz,D.K., Kuranga,S.A., Mamo,G., Zhou,Z., So,R.T.Y., Hemida,M.G., Webby,R.J., Roger,F., Rambaut,A., Poon,L.L.M., Perlman,S., Drosten,C., Chevalier,V., Peiris,M. | 2018-03-20T00:00:00Z | ssRNA(+) | 1353 | S protein | Nigeria | Camelus dromedarius | |
| AVN89431.1 | Chu,D.K.W., Hui,K.P.Y., Perera,R.A.P.M., Miguel,E., Niemeyer,D., Zhao,J., Channappanavar,R., Dudas,G., Oladipo,J.O., Traore,A., Fassi-Fihri,O., Ali,A., Demissie,G.F., Muth,D., Chan,M.C.W., Nicholls,J.M., Meyerholz,D.K., Kuranga,S.A., Mamo,G., Zhou,Z., So,R.T.Y., Hemida,M.G., Webby,R.J., Roger,F., Rambaut,A., Poon,L.L.M., Perlman,S., Drosten,C., Chevalier,V., Peiris,M. | 2018-03-20T00:00:00Z | ssRNA(+) | 1353 | S protein | Nigeria | Camelus dromedarius | |
| AVN89442.1 | Chu,D.K.W., Hui,K.P.Y., Perera,R.A.P.M., Miguel,E., Niemeyer,D., Zhao,J., Channappanavar,R., Dudas,G., Oladipo,J.O., Traore,A., Fassi-Fihri,O., Ali,A., Demissie,G.F., Muth,D., Chan,M.C.W., Nicholls,J.M., Meyerholz,D.K., Kuranga,S.A., Mamo,G., Zhou,Z., So,R.T.Y., Hemida,M.G., Webby,R.J., Roger,F., Rambaut,A., Poon,L.L.M., Perlman,S., Drosten,C., Chevalier,V., Peiris,M. | 2018-03-20T00:00:00Z | ssRNA(+) | 1353 | S protein | Nigeria | Camelus dromedarius | |
| AVN89453.1 | Chu,D.K.W., Hui,K.P.Y., Perera,R.A.P.M., Miguel,E., Niemeyer,D., Zhao,J., Channappanavar,R., Dudas,G., Oladipo,J.O., Traore,A., Fassi-Fihri,O., Ali,A., Demissie,G.F., Muth,D., Chan,M.C.W., Nicholls,J.M., Meyerholz,D.K., Kuranga,S.A., Mamo,G., Zhou,Z., So,R.T.Y., Hemida,M.G., Webby,R.J., Roger,F., Rambaut,A., Poon,L.L.M., Perlman,S., Drosten,C., Chevalier,V., Peiris,M. | 2018-03-20T00:00:00Z | ssRNA(+) | 1353 | S protein | Nigeria | Camelus dromedarius | |
| ALL26396.1 | Alagaili,A.N., Mohammed,O.B., Mishra,N., Briese,T., Lipkin,W.I. | 2016-09-30T00:00:00Z | ssRNA(+) | 1353 | S protein | Saudi Arabia | Camelus dromedarius | |
| ALL26409.1 | Alagaili,A.N., Mohammed,O.B., Mishra,N., Briese,T., Lipkin,W.I. | 2016-09-30T00:00:00Z | ssRNA(+) | 1353 | S protein | Saudi Arabia | Camelus dromedarius | |
| AHY21469.1 | Frey,K.G., Redden,C.L., Bishop-Lilly,K.A., Johnson,R., Hensley,L.E., Raviprakash,K., Luke,T., Kochel,T., Mokashi,V.P., Defang,G.N., Hensley,L., Kanakette,R. | 2014-05-04T00:00:00Z | ssRNA(+) | 1353 | S protein |  |  | |
| AHY22525.1 | Briese,T., Mishra,N., Jain,K., Zalmout,I.S., Jabado,O.J., Karesh,W.B., Daszak,P., Mohammed,O.B., Alagaili,A.N., Lipkin,W.I. | 2014-05-04T00:00:00Z | ssRNA(+) | 1353 | S protein | Saudi Arabia | Camelus dromedarius | |
| AHY22535.1 | Briese,T., Mishra,N., Jain,K., Zalmout,I.S., Jabado,O.J., Karesh,W.B., Daszak,P., Mohammed,O.B., Alagaili,A.N., Lipkin,W.I. | 2014-05-04T00:00:00Z | ssRNA(+) | 1353 | S protein | Saudi Arabia | Camelus dromedarius | |
| AHY22545.1 | Briese,T., Mishra,N., Jain,K., Zalmout,I.S., Jabado,O.J., Karesh,W.B., Daszak,P., Mohammed,O.B., Alagaili,A.N., Lipkin,W.I. | 2014-05-04T00:00:00Z | ssRNA(+) | 1353 | S protein | Saudi Arabia | Camelus dromedarius | |
| AHY22555.1 | Briese,T., Mishra,N., Jain,K., Zalmout,I.S., Jabado,O.J., Karesh,W.B., Daszak,P., Mohammed,O.B., Alagaili,A.N., Lipkin,W.I. | 2014-05-04T00:00:00Z | ssRNA(+) | 1353 | S protein | Saudi Arabia | Camelus dromedarius | |
| AHY22565.1 | Briese,T., Mishra,N., Jain,K., Zalmout,I.S., Jabado,O.J., Karesh,W.B., Daszak,P., Mohammed,O.B., Alagaili,A.N., Lipkin,W.I. | 2014-05-04T00:00:00Z | ssRNA(+) | 1353 | S protein | Saudi Arabia | Camelus dromedarius | |
| AHN10824.1 | Memish,Z.A., Cotten,M., Meyer,B., Watson,S.J., Alsahafi,A.J., Al Rabeeah,A.A., Corman,V.M., Sieberg,A., Makhdoom,H.Q., Assiri,A., Al Masri,M., Aldabbagh,S., Bosch,B.J., Beer,M., Muller,M.A., Kellam,P., Drosten,C., Palser,A.L., Zumla,A. | 2014-03-24T00:00:00Z | ssRNA(+) | 163 | S protein | Saudi Arabia | Camelus dromedarius | |
| AHN10826.1 | Memish,Z.A., Cotten,M., Meyer,B., Watson,S.J., Alsahafi,A.J., Al Rabeeah,A.A., Corman,V.M., Sieberg,A., Makhdoom,H.Q., Assiri,A., Al Masri,M., Aldabbagh,S., Bosch,B.J., Beer,M., Muller,M.A., Kellam,P., Drosten,C., Palser,A.L., Zumla,A. | 2014-03-24T00:00:00Z | ssRNA(+) | 214 | S protein | Saudi Arabia | Camelus dromedarius | |
| AHL31370.1 | Alagaili,A.N., Briese,T., Mishra,N., Kapoor,V., Sameroff,S.C., de Wit,E., Munster,V.J., Hensley,L.E., Zalmout,I.S., Kapoor,A., Epstein,J.H., Karesh,W.B., Daszak,P., Mohammed,O.B., Lipkin,W.I. | 2014-03-12T00:00:00Z | ssRNA(+) | 308 | S protein | Saudi Arabia | Camelus dromedarius | |
| AHL31372.1 | Alagaili,A.N., Briese,T., Mishra,N., Kapoor,V., Sameroff,S.C., de Wit,E., Munster,V.J., Hensley,L.E., Zalmout,I.S., Kapoor,A., Epstein,J.H., Karesh,W.B., Daszak,P., Mohammed,O.B., Lipkin,W.I. | 2014-03-12T00:00:00Z | ssRNA(+) | 308 | S protein | Saudi Arabia | Camelus dromedarius | |
| AHL31374.1 | Alagaili,A.N., Briese,T., Mishra,N., Kapoor,V., Sameroff,S.C., de Wit,E., Munster,V.J., Hensley,L.E., Zalmout,I.S., Kapoor,A., Epstein,J.H., Karesh,W.B., Daszak,P., Mohammed,O.B., Lipkin,W.I. | 2014-03-12T00:00:00Z | ssRNA(+) | 308 | S protein | Saudi Arabia | Camelus dromedarius | |
| AHL31376.1 | Alagaili,A.N., Briese,T., Mishra,N., Kapoor,V., Sameroff,S.C., de Wit,E., Munster,V.J., Hensley,L.E., Zalmout,I.S., Kapoor,A., Epstein,J.H., Karesh,W.B., Daszak,P., Mohammed,O.B., Lipkin,W.I. | 2014-03-12T00:00:00Z | ssRNA(+) | 308 | S protein | Saudi Arabia | Camelus dromedarius | |
| AHL31378.1 | Alagaili,A.N., Briese,T., Mishra,N., Kapoor,V., Sameroff,S.C., de Wit,E., Munster,V.J., Hensley,L.E., Zalmout,I.S., Kapoor,A., Epstein,J.H., Karesh,W.B., Daszak,P., Mohammed,O.B., Lipkin,W.I. | 2014-03-12T00:00:00Z | ssRNA(+) | 308 | S protein | Saudi Arabia | Camelus dromedarius | |
| AHL31380.1 | Alagaili,A.N., Briese,T., Mishra,N., Kapoor,V., Sameroff,S.C., de Wit,E., Munster,V.J., Hensley,L.E., Zalmout,I.S., Kapoor,A., Epstein,J.H., Karesh,W.B., Daszak,P., Mohammed,O.B., Lipkin,W.I. | 2014-03-12T00:00:00Z | ssRNA(+) | 308 | S protein | Saudi Arabia | Camelus dromedarius | |
| AHC74083.1 | Haagmans,B.L., Al Dhahiry,S.H., Reusken,C.B., Raj,V.S., Galiano,M., Myers,R., Godeke,G.J., Jonges,M., Farag,E., Diab,A., Ghobashy,H., Alhajri,F., Al-Thani,M., Al-Marri,S.A., Al Romaihi,H.E., Al Khal,A., Bermingham,A., Osterhaus,A.D., Alhajri,M.M., Koopmans,M.P., Al Dhahiry,S.H.S., Reusken,C.B.E.M., AlHajri,M.M., Koopmans,M.P.G. | 2013-12-19T00:00:00Z | ssRNA(+) | 119 | Spike | Qatar | Camelus dromedarius | |
| K9N5Q8.1 | Bermingham,A., Chand,M.A., Brown,C.S., Aarons,E., Tong,C., Langrish,C., Hoschler,K., Brown,K., Galiano,M., Myers,R., Pebody,R.G., Green,H.K., Boddington,N.L., Gopal,R., Price,N., Newsholme,W., Drosten,C., Fouchier,R.A., Zambon,M., Raj,V.S., Mou,H., Smits,S.L., Dekkers,D.H., Muller,M.A., Dijkman,R., Muth,D., Demmers,J.A., Zaki,A., Thiel,V., Rottier,P.J., Osterhaus,A.D., Bosch,B.J., Haagmans,B.L. | 2013-05-29T00:00:00Z | ssRNA(+) | 1353 | Spike glycoprotein | | Camelus dromedarius | |
| QJX19878.1 | Al-Shomrani,B.M., Manee,M.M., Alharbi,S.N., Altammami,M.A., Alshehri,M.A., Nassar,M.S., Bakhrebah,M.A., Al-Fageeh,M.B., Alshomrani,B.M., Alfageeh,M.B. | 2021-01-25T00:00:00Z | ssRNA(+) | 1353 | spike | Saudi Arabia | Camelus dromedarius | |
| QJX19888.1 | Al-Shomrani,B.M., Manee,M.M., Alharbi,S.N., Altammami,M.A., Alshehri,M.A., Nassar,M.S., Bakhrebah,M.A., Al-Fageeh,M.B., Alshomrani,B.M., Alfageeh,M.B. | 2021-01-25T00:00:00Z | ssRNA(+) | 1353 | spike | Saudi Arabia | Camelus dromedarius | |
| QJX19898.1 | Al-Shomrani,B.M., Manee,M.M., Alharbi,S.N., Altammami,M.A., Alshehri,M.A., Nassar,M.S., Bakhrebah,M.A., Al-Fageeh,M.B., Alshomrani,B.M., Alfageeh,M.B. | 2021-01-25T00:00:00Z | ssRNA(+) | 1353 | spike | Saudi Arabia | Camelus dromedarius | |
| QJX19908.1 | Al-Shomrani,B.M., Manee,M.M., Alharbi,S.N., Altammami,M.A., Alshehri,M.A., Nassar,M.S., Bakhrebah,M.A., Al-Fageeh,M.B., Alshomrani,B.M., Alfageeh,M.B. | 2021-01-25T00:00:00Z | ssRNA(+) | 1353 | spike | Saudi Arabia | Camelus dromedarius | |
| QJX19918.1 | Al-Shomrani,B.M., Manee,M.M., Alharbi,S.N., Altammami,M.A., Alshehri,M.A., Nassar,M.S., Bakhrebah,M.A., Al-Fageeh,M.B., Alshomrani,B.M., Alfageeh,M.B. | 2021-01-25T00:00:00Z | ssRNA(+) | 1353 | spike | Saudi Arabia | Camelus dromedarius | |
| QJX19928.1 | Al-Shomrani,B.M., Manee,M.M., Alharbi,S.N., Altammami,M.A., Alshehri,M.A., Nassar,M.S., Bakhrebah,M.A., Al-Fageeh,M.B., Alshomrani,B.M., Alfageeh,M.B. | 2021-01-25T00:00:00Z | ssRNA(+) | 1353 | spike | Saudi Arabia | Camelus dromedarius | |
| QJX19938.1 | Al-Shomrani,B.M., Manee,M.M., Alharbi,S.N., Altammami,M.A., Alshehri,M.A., Nassar,M.S., Bakhrebah,M.A., Al-Fageeh,M.B., Alshomrani,B.M., Alfageeh,M.B. | 2021-01-25T00:00:00Z | ssRNA(+) | 1353 | spike | Saudi Arabia | Camelus dromedarius | |
| QJX19948.1 | Al-Shomrani,B.M., Manee,M.M., Alharbi,S.N., Altammami,M.A., Alshehri,M.A., Nassar,M.S., Bakhrebah,M.A., Al-Fageeh,M.B., Alshomrani,B.M., Alfageeh,M.B. | 2021-01-25T00:00:00Z | ssRNA(+) | 1353 | spike | Saudi Arabia | Camelus dromedarius | |
| AWF93650.1 | Kandeil,A. Sr., Gomaa,M., Shehata,M., Ali,M.A., Kayali,G. | 2019-05-01T00:00:00Z | ssRNA(+) | 234 | spike | Egypt | Equus asinus | |
| AWF93651.1 | Kandeil,A. Sr., Gomaa,M., Shehata,M., Ali,M.A., Kayali,G. | 2019-05-01T00:00:00Z | ssRNA(+) | 232 | spike | Egypt | Capra hircus | |
| AWF93652.1 | Kandeil,A. Sr., Gomaa,M., Shehata,M., Ali,M.A., Kayali,G. | 2019-05-01T00:00:00Z | ssRNA(+) | 229 | spike | Egypt | Bovidae | |
| AWF93653.1 | Kandeil,A. Sr., Gomaa,M., Shehata,M., Ali,M.A., Kayali,G. | 2019-05-01T00:00:00Z | ssRNA(+) | 225 | spike | Egypt | Camelus | |
| AWF93654.1 | Kandeil,A. Sr., Gomaa,M., Shehata,M., Ali,M.A., Kayali,G. | 2019-05-01T00:00:00Z | ssRNA(+) | 230 | spike | Egypt | Capra hircus | |
| AWF93655.1 | Kandeil,A. Sr., Gomaa,M., Shehata,M., Ali,M.A., Kayali,G. | 2019-05-01T00:00:00Z | ssRNA(+) | 240 | spike | Egypt | Capra hircus | |
| AWF93656.1 | Kandeil,A. Sr., Gomaa,M., Shehata,M., Ali,M.A., Kayali,G. | 2019-05-01T00:00:00Z | ssRNA(+) | 220 | spike | Egypt | Camelus | |
| AWF93657.1 | Kandeil,A. Sr., Gomaa,M., Shehata,M., Ali,M.A., Kayali,G. | 2019-05-01T00:00:00Z | ssRNA(+) | 234 | spike | Egypt | Camelus | |
| QBM11737.1 | Shirato,K., Melaku,S.K., Kawachi,K., Nao,N., Iwata-Yoshikawa,N., Kawase,M., Kamitani,W., Matsuyama,S., Tessema,T.S., Sentsui,H. | 2019-03-20T00:00:00Z | ssRNA(+) | 1353 | spike | Ethiopia | Camelus dromedarius | |
| QBM11748.1 | Shirato,K., Melaku,S.K., Kawachi,K., Nao,N., Iwata-Yoshikawa,N., Kawase,M., Kamitani,W., Matsuyama,S., Tessema,T.S., Sentsui,H. | 2019-03-20T00:00:00Z | ssRNA(+) | 1353 | spike | Ethiopia | Camelus dromedarius | |
| APT69959.1 | Shehata,M.M., Kandeil,A., El-Shesheny,R., Ali,M.A.A., Kayali,G. | 2017-01-15T00:00:00Z | ssRNA(+) | 183 | spike | Egypt | Camelus dromedarius | |
| APT69960.1 | Shehata,M.M., Kandeil,A., El-Shesheny,R., Ali,M.A.A., Kayali,G. | 2017-01-15T00:00:00Z | ssRNA(+) | 194 | spike | Egypt | Camelus dromedarius | |
| APT69961.1 | Shehata,M.M., Kandeil,A., El-Shesheny,R., Ali,M.A.A., Kayali,G. | 2017-01-15T00:00:00Z | ssRNA(+) | 202 | spike | Egypt | Camelus dromedarius | |
| APT69962.1 | Shehata,M.M., Kandeil,A., El-Shesheny,R., Ali,M.A.A., Kayali,G. | 2017-01-15T00:00:00Z | ssRNA(+) | 204 | spike | Egypt | Camelus dromedarius | |
| APT69963.1 | Shehata,M.M., Kandeil,A., El-Shesheny,R., Ali,M.A.A., Kayali,G. | 2017-01-15T00:00:00Z | ssRNA(+) | 203 | spike | Egypt | Camelus dromedarius | |
| APT69964.1 | Shehata,M.M., Kandeil,A., El-Shesheny,R., Ali,M.A.A., Kayali,G. | 2017-01-15T00:00:00Z | ssRNA(+) | 202 | spike | Egypt | Camelus dromedarius | |
| APT69965.1 | Shehata,M.M., Kandeil,A., El-Shesheny,R., Ali,M.A.A., Kayali,G. | 2017-01-15T00:00:00Z | ssRNA(+) | 204 | spike | Egypt | Camelus dromedarius | |
| APT69966.1 | Shehata,M.M., Kandeil,A., El-Shesheny,R., Ali,M.A.A., Kayali,G. | 2017-01-15T00:00:00Z | ssRNA(+) | 203 | spike | Egypt | Camelus dromedarius | |
| APT69967.1 | Shehata,M.M., Kandeil,A., El-Shesheny,R., Ali,M.A.A., Kayali,G. | 2017-01-15T00:00:00Z | ssRNA(+) | 205 | spike | Egypt | Camelus dromedarius | |
| APT69968.1 | Shehata,M.M., Kandeil,A., El-Shesheny,R., Ali,M.A.A., Kayali,G. | 2017-01-15T00:00:00Z | ssRNA(+) | 204 | spike | Egypt | Camelus dromedarius | |
| APT69969.1 | Shehata,M.M., Kandeil,A., El-Shesheny,R., Ali,M.A.A., Kayali,G. | 2017-01-15T00:00:00Z | ssRNA(+) | 202 | spike | Egypt | Camelus dromedarius | |
| APT69970.1 | Shehata,M.M., Kandeil,A., El-Shesheny,R., Ali,M.A.A., Kayali,G. | 2017-01-15T00:00:00Z | ssRNA(+) | 182 | spike | Egypt | Camelus dromedarius | |
| APT69971.1 | Shehata,M.M., Kandeil,A., El-Shesheny,R., Ali,M.A.A., Kayali,G. | 2017-01-15T00:00:00Z | ssRNA(+) | 192 | spike | Egypt | Camelus dromedarius | |
| APT69972.1 | Shehata,M.M., Kandeil,A., El-Shesheny,R., Ali,M.A.A., Kayali,G. | 2017-01-15T00:00:00Z | ssRNA(+) | 182 | spike | Egypt | Camelus dromedarius | |
| APT69973.1 | Shehata,M.M., Kandeil,A., El-Shesheny,R., Ali,M.A.A., Kayali,G. | 2017-01-15T00:00:00Z | ssRNA(+) | 184 | spike | Egypt | Camelus dromedarius | |
| APT69974.1 | Shehata,M.M., Kandeil,A., El-Shesheny,R., Ali,M.A.A., Kayali,G. | 2017-01-15T00:00:00Z | ssRNA(+) | 192 | spike | Egypt | Camelus dromedarius | |
| APT69975.1 | Shehata,M.M., Kandeil,A., El-Shesheny,R., Ali,M.A.A., Kayali,G. | 2017-01-15T00:00:00Z | ssRNA(+) | 181 | spike | Egypt | Camelus dromedarius | |
| APT69976.1 | Shehata,M.M., Kandeil,A., El-Shesheny,R., Ali,M.A.A., Kayali,G. | 2017-01-15T00:00:00Z | ssRNA(+) | 200 | spike | Egypt | Camelus dromedarius | |
| APT69977.1 | Shehata,M.M., Kandeil,A., El-Shesheny,R., Ali,M.A.A., Kayali,G. | 2017-01-15T00:00:00Z | ssRNA(+) | 199 | spike | Egypt | Camelus dromedarius | |
| APT69978.1 | Shehata,M.M., Kandeil,A., El-Shesheny,R., Ali,M.A.A., Kayali,G. | 2017-01-15T00:00:00Z | ssRNA(+) | 199 | spike | Egypt | Camelus dromedarius | |
| APT69979.1 | Shehata,M.M., Kandeil,A., El-Shesheny,R., Ali,M.A.A., Kayali,G. | 2017-01-15T00:00:00Z | ssRNA(+) | 199 | spike | Egypt | Camelus dromedarius | |
| ALU34109.1 | Haagmans,B.L., van den Brand,J.M., Raj,V.S., Volz,A., Wohlsein,P., Smits,S.L., Schipper,D., Bestebroer,T.M., Okba,N., Fux,R., Bensaid,A., Solanes Foz,D., Kuiken,T., Baumgartner,W., Segales,J., Sutter,G., Osterhaus,A.D., Van den Brand,J.M.A., Stalin Raj,V., Osterhaus,A.D.M.E. | 2015-12-28T00:00:00Z | ssRNA(+) | 231 | spike | Netherlands |  | |
| ALU34110.1 | Haagmans,B.L., van den Brand,J.M., Raj,V.S., Volz,A., Wohlsein,P., Smits,S.L., Schipper,D., Bestebroer,T.M., Okba,N., Fux,R., Bensaid,A., Solanes Foz,D., Kuiken,T., Baumgartner,W., Segales,J., Sutter,G., Osterhaus,A.D., Van den Brand,J.M.A., Stalin Raj,V., Osterhaus,A.D.M.E. | 2015-12-28T00:00:00Z | ssRNA(+) | 231 | spike | Spain | Camelus dromedarius | |
| AJO62171.1 | Yusof,M.F., Eltahir,Y.M., Serhan,W.S., Hashem,F.M., Elsayed,E.A., Marzoug,B.A., Abdelazim,A.S., Bensalah,O.K., Al Muhairi,S.S., Yusof,M.M., Alsayed,E.A., Marzouq,B.A., Abelazim,A.S. | 2015-02-24T00:00:00Z | ssRNA(+) | 342 | spike | United Arab Emirates | Camelus dromedarius | |
| AJO62172.1 | Yusof,M.F., Eltahir,Y.M., Serhan,W.S., Hashem,F.M., Elsayed,E.A., Marzoug,B.A., Abdelazim,A.S., Bensalah,O.K., Al Muhairi,S.S., Yusof,M.M., Alsayed,E.A., Marzouq,B.A., Abelazim,A.S. | 2015-02-24T00:00:00Z | ssRNA(+) | 341 | spike | United Arab Emirates | Camelus dromedarius | |
| AJO62173.1 | Yusof,M.F., Eltahir,Y.M., Serhan,W.S., Hashem,F.M., Elsayed,E.A., Marzoug,B.A., Abdelazim,A.S., Bensalah,O.K., Al Muhairi,S.S., Yusof,M.M., Alsayed,E.A., Marzouq,B.A., Abelazim,A.S. | 2015-02-24T00:00:00Z | ssRNA(+) | 341 | spike | United Arab Emirates | Camelus dromedarius | |
| AJO62174.1 | Yusof,M.F., Eltahir,Y.M., Serhan,W.S., Hashem,F.M., Elsayed,E.A., Marzoug,B.A., Abdelazim,A.S., Bensalah,O.K., Al Muhairi,S.S., Yusof,M.M., Alsayed,E.A., Marzouq,B.A., Abelazim,A.S. | 2015-02-24T00:00:00Z | ssRNA(+) | 273 | spike | United Arab Emirates | Camelus dromedarius | |
| AJO62175.1 | Yusof,M.F., Eltahir,Y.M., Serhan,W.S., Hashem,F.M., Elsayed,E.A., Marzoug,B.A., Abdelazim,A.S., Bensalah,O.K., Al Muhairi,S.S., Yusof,M.M., Alsayed,E.A., Marzouq,B.A., Abelazim,A.S. | 2015-02-24T00:00:00Z | ssRNA(+) | 341 | spike | United Arab Emirates | Camelus dromedarius | |
| AJO62176.1 | Yusof,M.F., Eltahir,Y.M., Serhan,W.S., Hashem,F.M., Elsayed,E.A., Marzoug,B.A., Abdelazim,A.S., Bensalah,O.K., Al Muhairi,S.S., Yusof,M.M., Alsayed,E.A., Marzouq,B.A., Abelazim,A.S. | 2015-02-24T00:00:00Z | ssRNA(+) | 341 | spike | United Arab Emirates | Camelus dromedarius | |
| AJO62177.1 | Yusof,M.F., Eltahir,Y.M., Serhan,W.S., Hashem,F.M., Elsayed,E.A., Marzoug,B.A., Abdelazim,A.S., Bensalah,O.K., Al Muhairi,S.S., Yusof,M.M., Alsayed,E.A., Marzouq,B.A., Abelazim,A.S. | 2015-02-24T00:00:00Z | ssRNA(+) | 253 | spike | United Arab Emirates | Camelus dromedarius | |
| AJO62178.1 | Yusof,M.F., Eltahir,Y.M., Serhan,W.S., Hashem,F.M., Elsayed,E.A., Marzoug,B.A., Abdelazim,A.S., Bensalah,O.K., Al Muhairi,S.S., Yusof,M.M., Alsayed,E.A., Marzouq,B.A., Abelazim,A.S. | 2015-02-24T00:00:00Z | ssRNA(+) | 341 | spike | United Arab Emirates | Camelus dromedarius | |
| AJO62179.1 | Yusof,M.F., Eltahir,Y.M., Serhan,W.S., Hashem,F.M., Elsayed,E.A., Marzoug,B.A., Abdelazim,A.S., Bensalah,O.K., Al Muhairi,S.S., Yusof,M.M., Alsayed,E.A., Marzouq,B.A., Abelazim,A.S. | 2015-02-24T00:00:00Z | ssRNA(+) | 341 | spike | United Arab Emirates | Camelus dromedarius | |
| AJO62180.1 | Yusof,M.F., Eltahir,Y.M., Serhan,W.S., Hashem,F.M., Elsayed,E.A., Marzoug,B.A., Abdelazim,A.S., Bensalah,O.K., Al Muhairi,S.S., Yusof,M.M., Alsayed,E.A., Marzouq,B.A., Abelazim,A.S. | 2015-02-24T00:00:00Z | ssRNA(+) | 341 | spike | United Arab Emirates | Camelus dromedarius | |
| AJO62181.1 | Yusof,M.F., Eltahir,Y.M., Serhan,W.S., Hashem,F.M., Elsayed,E.A., Marzoug,B.A., Abdelazim,A.S., Bensalah,O.K., Al Muhairi,S.S., Yusof,M.M., Alsayed,E.A., Marzouq,B.A., Abelazim,A.S. | 2015-02-24T00:00:00Z | ssRNA(+) | 341 | spike | United Arab Emirates | Camelus dromedarius | |
| AJO62182.1 | Yusof,M.F., Eltahir,Y.M., Serhan,W.S., Hashem,F.M., Elsayed,E.A., Marzoug,B.A., Abdelazim,A.S., Bensalah,O.K., Al Muhairi,S.S., Yusof,M.M., Alsayed,E.A., Marzouq,B.A., Abelazim,A.S. | 2015-02-24T00:00:00Z | ssRNA(+) | 341 | spike | United Arab Emirates | Camelus dromedarius | |
| AJO62183.1 | Yusof,M.F., Eltahir,Y.M., Serhan,W.S., Hashem,F.M., Elsayed,E.A., Marzoug,B.A., Abdelazim,A.S., Bensalah,O.K., Al Muhairi,S.S., Yusof,M.M., Alsayed,E.A., Marzouq,B.A., Abelazim,A.S. | 2015-02-24T00:00:00Z | ssRNA(+) | 355 | spike | United Arab Emirates | Camelus dromedarius | |
| AJO62184.1 | Yusof,M.F., Eltahir,Y.M., Serhan,W.S., Hashem,F.M., Elsayed,E.A., Marzoug,B.A., Abdelazim,A.S., Bensalah,O.K., Al Muhairi,S.S., Yusof,M.M., Alsayed,E.A., Marzouq,B.A., Abelazim,A.S. | 2015-02-24T00:00:00Z | ssRNA(+) | 200 | spike | United Arab Emirates | Camelus dromedarius | |
| AJO62185.1 | Yusof,M.F., Eltahir,Y.M., Serhan,W.S., Hashem,F.M., Elsayed,E.A., Marzoug,B.A., Abdelazim,A.S., Bensalah,O.K., Al Muhairi,S.S., Yusof,M.M., Alsayed,E.A., Marzouq,B.A., Abelazim,A.S. | 2015-02-24T00:00:00Z | ssRNA(+) | 189 | spike | United Arab Emirates | Camelus dromedarius | |
| AJO62186.1 | Yusof,M.F., Eltahir,Y.M., Serhan,W.S., Hashem,F.M., Elsayed,E.A., Marzoug,B.A., Abdelazim,A.S., Bensalah,O.K., Al Muhairi,S.S., Yusof,M.M., Alsayed,E.A., Marzouq,B.A., Abelazim,A.S. | 2015-02-24T00:00:00Z | ssRNA(+) | 202 | spike | United Arab Emirates | Camelus dromedarius | |
| AJO62187.1 | Yusof,M.F., Eltahir,Y.M., Serhan,W.S., Hashem,F.M., Elsayed,E.A., Marzoug,B.A., Abdelazim,A.S., Bensalah,O.K., Al Muhairi,S.S., Yusof,M.M., Alsayed,E.A., Marzouq,B.A., Abelazim,A.S. | 2015-02-24T00:00:00Z | ssRNA(+) | 355 | spike | United Arab Emirates | Camelus dromedarius | |
| AJO62188.1 | Yusof,M.F., Eltahir,Y.M., Serhan,W.S., Hashem,F.M., Elsayed,E.A., Marzoug,B.A., Abdelazim,A.S., Bensalah,O.K., Al Muhairi,S.S., Yusof,M.M., Alsayed,E.A., Marzouq,B.A., Abelazim,A.S. | 2015-02-24T00:00:00Z | ssRNA(+) | 355 | spike | United Arab Emirates | Camelus dromedarius | |
| AJO62189.1 | Yusof,M.F., Eltahir,Y.M., Serhan,W.S., Hashem,F.M., Elsayed,E.A., Marzoug,B.A., Abdelazim,A.S., Bensalah,O.K., Al Muhairi,S.S., Yusof,M.M., Alsayed,E.A., Marzouq,B.A., Abelazim,A.S. | 2015-02-24T00:00:00Z | ssRNA(+) | 189 | spike | United Arab Emirates | Camelus dromedarius | |
| AJO62190.1 | Yusof,M.F., Eltahir,Y.M., Serhan,W.S., Hashem,F.M., Elsayed,E.A., Marzoug,B.A., Abdelazim,A.S., Bensalah,O.K., Al Muhairi,S.S., Yusof,M.M., Alsayed,E.A., Marzouq,B.A., Abelazim,A.S. | 2015-02-24T00:00:00Z | ssRNA(+) | 355 | spike | United Arab Emirates | Camelus dromedarius | |
| AJO62191.1 | Yusof,M.F., Eltahir,Y.M., Serhan,W.S., Hashem,F.M., Elsayed,E.A., Marzoug,B.A., Abdelazim,A.S., Bensalah,O.K., Al Muhairi,S.S., Yusof,M.M., Alsayed,E.A., Marzouq,B.A., Abelazim,A.S. | 2015-02-24T00:00:00Z | ssRNA(+) | 201 | spike | United Arab Emirates | Camelus dromedarius | |
| AJO62192.1 | Yusof,M.F., Eltahir,Y.M., Serhan,W.S., Hashem,F.M., Elsayed,E.A., Marzoug,B.A., Abdelazim,A.S., Bensalah,O.K., Al Muhairi,S.S., Yusof,M.M., Alsayed,E.A., Marzouq,B.A., Abelazim,A.S. | 2015-02-24T00:00:00Z | ssRNA(+) | 202 | spike | United Arab Emirates | Camelus dromedarius | |
| AJO62193.1 | Yusof,M.F., Eltahir,Y.M., Serhan,W.S., Hashem,F.M., Elsayed,E.A., Marzoug,B.A., Abdelazim,A.S., Bensalah,O.K., Al Muhairi,S.S., Yusof,M.M., Alsayed,E.A., Marzouq,B.A., Abelazim,A.S. | 2015-02-24T00:00:00Z | ssRNA(+) | 355 | spike | United Arab Emirates | Camelus dromedarius | |
| AJO62194.1 | Yusof,M.F., Eltahir,Y.M., Serhan,W.S., Hashem,F.M., Elsayed,E.A., Marzoug,B.A., Abdelazim,A.S., Bensalah,O.K., Al Muhairi,S.S., Yusof,M.M., Alsayed,E.A., Marzouq,B.A., Abelazim,A.S. | 2015-02-24T00:00:00Z | ssRNA(+) | 202 | spike | United Arab Emirates | Camelus dromedarius | |
| AHX00711.1 | Hemida,M.G., Chu,D.K., Poon,L.L., Perera,R.A., Alhammadi,M.A., Ng,H.Y., Siu,L.Y., Guan,Y., Alnaeem,A., Peiris,M., Chu,D.K.W., Poon,L.L.M., Ng,H. | 2014-04-22T00:00:00Z | ssRNA(+) | 1353 | spike | Saudi Arabia | Camelus dromedarius | |
| AHX00721.1 | Hemida,M.G., Chu,D.K., Poon,L.L., Perera,R.A., Alhammadi,M.A., Ng,H.Y., Siu,L.Y., Guan,Y., Alnaeem,A., Peiris,M., Chu,D.K.W., Poon,L.L.M., Ng,H. | 2014-04-22T00:00:00Z | ssRNA(+) | 1353 | spike | Saudi Arabia | Camelus dromedarius | |
| AHX00731.1 | Hemida,M.G., Chu,D.K., Poon,L.L., Perera,R.A., Alhammadi,M.A., Ng,H.Y., Siu,L.Y., Guan,Y., Alnaeem,A., Peiris,M., Chu,D.K.W., Poon,L.L.M., Ng,H. | 2014-04-22T00:00:00Z | ssRNA(+) | 1353 | spike | Saudi Arabia | Camelus dromedarius | |
| QQK33709.1 | Eckstein,S., Ehmann,R., Gritli,A., Ben Yahia,H., Diehl,M., Woelfel,R., Ben Rhaiem,M., Stoecker,K., Handrick,S., Ben Moussa,M. | 2022-12-01T00:00:00Z | ssRNA(+) | 212 | spike glycoprotein | Tunisia | Camelus dromedarius | |
| QQK33710.1 | Eckstein,S., Ehmann,R., Gritli,A., Ben Yahia,H., Diehl,M., Woelfel,R., Ben Rhaiem,M., Stoecker,K., Handrick,S., Ben Moussa,M. | 2022-12-01T00:00:00Z | ssRNA(+) | 212 | spike glycoprotein | Tunisia | Camelus dromedarius | |
| QQK33711.1 | Eckstein,S., Ehmann,R., Gritli,A., Ben Yahia,H., Diehl,M., Woelfel,R., Ben Rhaiem,M., Stoecker,K., Handrick,S., Ben Moussa,M. | 2022-12-01T00:00:00Z | ssRNA(+) | 212 | spike glycoprotein | Tunisia | Camelus dromedarius | |
| QQK33712.1 | Eckstein,S., Ehmann,R., Gritli,A., Ben Yahia,H., Diehl,M., Woelfel,R., Ben Rhaiem,M., Stoecker,K., Handrick,S., Ben Moussa,M. | 2022-12-01T00:00:00Z | ssRNA(+) | 212 | spike glycoprotein | Tunisia | Camelus dromedarius | |
| QQK33713.1 | Eckstein,S., Ehmann,R., Gritli,A., Ben Yahia,H., Diehl,M., Woelfel,R., Ben Rhaiem,M., Stoecker,K., Handrick,S., Ben Moussa,M. | 2022-12-01T00:00:00Z | ssRNA(+) | 212 | spike glycoprotein | Tunisia | Camelus dromedarius | |
| QQK33714.1 | Eckstein,S., Ehmann,R., Gritli,A., Ben Yahia,H., Diehl,M., Woelfel,R., Ben Rhaiem,M., Stoecker,K., Handrick,S., Ben Moussa,M. | 2022-12-01T00:00:00Z | ssRNA(+) | 212 | spike glycoprotein | Tunisia | Camelus dromedarius | |
| QQK33715.1 | Eckstein,S., Ehmann,R., Gritli,A., Ben Yahia,H., Diehl,M., Woelfel,R., Ben Rhaiem,M., Stoecker,K., Handrick,S., Ben Moussa,M. | 2022-12-01T00:00:00Z | ssRNA(+) | 212 | spike glycoprotein | Tunisia | Camelus dromedarius | |
| QQK33716.1 | Eckstein,S., Ehmann,R., Gritli,A., Ben Yahia,H., Diehl,M., Woelfel,R., Ben Rhaiem,M., Stoecker,K., Handrick,S., Ben Moussa,M. | 2022-12-01T00:00:00Z | ssRNA(+) | 212 | spike glycoprotein | Tunisia | Camelus dromedarius | |
| QQK33717.1 | Eckstein,S., Ehmann,R., Gritli,A., Ben Yahia,H., Diehl,M., Woelfel,R., Ben Rhaiem,M., Stoecker,K., Handrick,S., Ben Moussa,M. | 2022-12-01T00:00:00Z | ssRNA(+) | 212 | spike glycoprotein | Tunisia | Camelus dromedarius | |
| QQK33718.1 | Eckstein,S., Ehmann,R., Gritli,A., Ben Yahia,H., Diehl,M., Woelfel,R., Ben Rhaiem,M., Stoecker,K., Handrick,S., Ben Moussa,M. | 2022-12-01T00:00:00Z | ssRNA(+) | 212 | spike glycoprotein | Tunisia | Camelus dromedarius | |
| QQK33719.1 | Eckstein,S., Ehmann,R., Gritli,A., Ben Yahia,H., Diehl,M., Woelfel,R., Ben Rhaiem,M., Stoecker,K., Handrick,S., Ben Moussa,M. | 2022-12-01T00:00:00Z | ssRNA(+) | 212 | spike glycoprotein | Tunisia | Camelus dromedarius | |
| QQK33720.1 | Eckstein,S., Ehmann,R., Gritli,A., Ben Yahia,H., Diehl,M., Woelfel,R., Ben Rhaiem,M., Stoecker,K., Handrick,S., Ben Moussa,M. | 2022-12-01T00:00:00Z | ssRNA(+) | 212 | spike glycoprotein | Tunisia | Camelus dromedarius | |
| QQK33721.1 | Eckstein,S., Ehmann,R., Gritli,A., Ben Yahia,H., Diehl,M., Woelfel,R., Ben Rhaiem,M., Stoecker,K., Handrick,S., Ben Moussa,M. | 2022-12-01T00:00:00Z | ssRNA(+) | 212 | spike glycoprotein | Tunisia | Camelus dromedarius | |
| QQK33722.1 | Eckstein,S., Ehmann,R., Gritli,A., Ben Yahia,H., Diehl,M., Woelfel,R., Ben Rhaiem,M., Stoecker,K., Handrick,S., Ben Moussa,M. | 2022-12-01T00:00:00Z | ssRNA(+) | 212 | spike glycoprotein | Tunisia | Camelus dromedarius | |
| UYR00344.1 | Kandeil,A., Gomaa,M.R., Shehata,M., Taweel,A.E., Kayed,A., El-Shesheny,R., Alabady,M., Bahl,J., Ali,M.A., Kayali,G. | 2022-11-01T00:00:00Z | ssRNA(+) | 1353 | spike glycoprotein | Egypt | Camelus dromedarius | |
| UYR00354.1 | Kandeil,A., Gomaa,M.R., Shehata,M., Taweel,A.E., Kayed,A., El-Shesheny,R., Alabady,M., Bahl,J., Ali,M.A., Kayali,G. | 2022-11-01T00:00:00Z | ssRNA(+) | 1353 | spike glycoprotein | Egypt | Camelus dromedarius | |
| UYR00365.1 | Kandeil,A., Gomaa,M.R., Shehata,M., Taweel,A.E., Kayed,A., El-Shesheny,R., Alabady,M., Bahl,J., Ali,M.A., Kayali,G. | 2022-11-01T00:00:00Z | ssRNA(+) | 1353 | spike glycoprotein | Egypt | Camelus dromedarius | |
| UBS90429.1 | Ngere,I., Hunsperger,E.A., Tong,S., Oyugi,J., Jaoko,W., Harcourt,J.L., Thornburg,N.J., Oyas,H., Muturi,M., Osoro,E.M., Gachohi,J., Ombok,C., Dawa,J., Tao,Y., Zhang,J., Mwasi,L., Ochieng,C., Mwatondo,A., Bodha,B., Langat,D., Herman-Roloff,A., Njenga,M.K., Widdowson,M.A., Munyua,P.M., Hunsperger,E., Lidechi,S., Onyango,C., Paden,C. | 2022-09-02T00:00:00Z | ssRNA(+) | 1353 | spike glycoprotein | Kenya |  | |
| UBS90440.1 | Ngere,I., Hunsperger,E.A., Tong,S., Oyugi,J., Jaoko,W., Harcourt,J.L., Thornburg,N.J., Oyas,H., Muturi,M., Osoro,E.M., Gachohi,J., Ombok,C., Dawa,J., Tao,Y., Zhang,J., Mwasi,L., Ochieng,C., Mwatondo,A., Bodha,B., Langat,D., Herman-Roloff,A., Njenga,M.K., Widdowson,M.A., Munyua,P.M., Hunsperger,E., Lidechi,S., Onyango,C., Paden,C. | 2022-09-02T00:00:00Z | ssRNA(+) | 1353 | spike glycoprotein | Kenya |  | |
| UBS90451.1 | Ngere,I., Hunsperger,E.A., Tong,S., Oyugi,J., Jaoko,W., Harcourt,J.L., Thornburg,N.J., Oyas,H., Muturi,M., Osoro,E.M., Gachohi,J., Ombok,C., Dawa,J., Tao,Y., Zhang,J., Mwasi,L., Ochieng,C., Mwatondo,A., Bodha,B., Langat,D., Herman-Roloff,A., Njenga,M.K., Widdowson,M.A., Munyua,P.M., Hunsperger,E., Lidechi,S., Onyango,C., Paden,C. | 2022-09-02T00:00:00Z | ssRNA(+) | 1353 | spike glycoprotein | Kenya | Camelus | |
| UBS90462.1 | Ngere,I., Hunsperger,E.A., Tong,S., Oyugi,J., Jaoko,W., Harcourt,J.L., Thornburg,N.J., Oyas,H., Muturi,M., Osoro,E.M., Gachohi,J., Ombok,C., Dawa,J., Tao,Y., Zhang,J., Mwasi,L., Ochieng,C., Mwatondo,A., Bodha,B., Langat,D., Herman-Roloff,A., Njenga,M.K., Widdowson,M.A., Munyua,P.M., Hunsperger,E., Lidechi,S., Onyango,C., Paden,C. | 2022-09-02T00:00:00Z | ssRNA(+) | 1353 | spike glycoprotein | Kenya | Camelus | |
| UBS90473.1 | Ngere,I., Hunsperger,E.A., Tong,S., Oyugi,J., Jaoko,W., Harcourt,J.L., Thornburg,N.J., Oyas,H., Muturi,M., Osoro,E.M., Gachohi,J., Ombok,C., Dawa,J., Tao,Y., Zhang,J., Mwasi,L., Ochieng,C., Mwatondo,A., Bodha,B., Langat,D., Herman-Roloff,A., Njenga,M.K., Widdowson,M.A., Munyua,P.M., Hunsperger,E., Lidechi,S., Onyango,C., Paden,C. | 2022-09-02T00:00:00Z | ssRNA(+) | 1353 | spike glycoprotein | Kenya | Camelus | |
| UBS90484.1 | Ngere,I., Hunsperger,E.A., Tong,S., Oyugi,J., Jaoko,W., Harcourt,J.L., Thornburg,N.J., Oyas,H., Muturi,M., Osoro,E.M., Gachohi,J., Ombok,C., Dawa,J., Tao,Y., Zhang,J., Mwasi,L., Ochieng,C., Mwatondo,A., Bodha,B., Langat,D., Herman-Roloff,A., Njenga,M.K., Widdowson,M.A., Munyua,P.M., Hunsperger,E., Lidechi,S., Onyango,C., Paden,C. | 2022-09-02T00:00:00Z | ssRNA(+) | 1353 | spike glycoprotein | Kenya |  | |
| UBS90495.1 | Ngere,I., Hunsperger,E.A., Tong,S., Oyugi,J., Jaoko,W., Harcourt,J.L., Thornburg,N.J., Oyas,H., Muturi,M., Osoro,E.M., Gachohi,J., Ombok,C., Dawa,J., Tao,Y., Zhang,J., Mwasi,L., Ochieng,C., Mwatondo,A., Bodha,B., Langat,D., Herman-Roloff,A., Njenga,M.K., Widdowson,M.A., Munyua,P.M., Hunsperger,E., Lidechi,S., Onyango,C., Paden,C. | 2022-09-02T00:00:00Z | ssRNA(+) | 1353 | spike glycoprotein | Kenya | Camelus | |
| UBS90506.1 | Ngere,I., Hunsperger,E.A., Tong,S., Oyugi,J., Jaoko,W., Harcourt,J.L., Thornburg,N.J., Oyas,H., Muturi,M., Osoro,E.M., Gachohi,J., Ombok,C., Dawa,J., Tao,Y., Zhang,J., Mwasi,L., Ochieng,C., Mwatondo,A., Bodha,B., Langat,D., Herman-Roloff,A., Njenga,M.K., Widdowson,M.A., Munyua,P.M., Hunsperger,E., Lidechi,S., Onyango,C., Paden,C. | 2022-09-02T00:00:00Z | ssRNA(+) | 1353 | spike glycoprotein | Kenya | Camelus | |
| UBS90517.1 | Ngere,I., Hunsperger,E.A., Tong,S., Oyugi,J., Jaoko,W., Harcourt,J.L., Thornburg,N.J., Oyas,H., Muturi,M., Osoro,E.M., Gachohi,J., Ombok,C., Dawa,J., Tao,Y., Zhang,J., Mwasi,L., Ochieng,C., Mwatondo,A., Bodha,B., Langat,D., Herman-Roloff,A., Njenga,M.K., Widdowson,M.A., Munyua,P.M., Hunsperger,E., Lidechi,S., Onyango,C., Paden,C. | 2022-09-02T00:00:00Z | ssRNA(+) | 1353 | spike glycoprotein | Kenya |  | |
| AXG21643.1 | Al Balwi,M., Khan,A., UdayaRaja,G.K., AlMasoud,A., AlAbdulkareem,I., AlAsiri,A., Mani Balavenkatesh,M., AlHarbi,W., El-Saed,A., Balkhy,H. | 2018-07-31T00:00:00Z | ssRNA(+) | 1353 | spike glycoprotein | Saudi Arabia |  | |
| ASJ26610.1 | Hemida,M.G., Alnaeem,A., Chu,D.K., Perera,R.A., Chan,S.M., Almathen,F., Yau,E., Ng,B.C., Webby,R.J., Poon,L.L., Peiris,M., Chu,D.K.W., Perera,R.A.P.M., Chan,S.M.S., Ng,B.C.Y., Poon,L.L.M. | 2018-05-31T00:00:00Z | ssRNA(+) | 1126 | spike glycoprotein | Saudi Arabia | Camelus dromedarius | |
| ASJ26618.1 | Hemida,M.G., Alnaeem,A., Chu,D.K., Perera,R.A., Chan,S.M., Almathen,F., Yau,E., Ng,B.C., Webby,R.J., Poon,L.L., Peiris,M., Chu,D.K.W., Perera,R.A.P.M., Chan,S.M.S., Ng,B.C.Y., Poon,L.L.M. | 2018-05-31T00:00:00Z | ssRNA(+) | 1126 | spike glycoprotein | Saudi Arabia | Camelus dromedarius | |
| ASJ26626.1 | Hemida,M.G., Alnaeem,A., Chu,D.K., Perera,R.A., Chan,S.M., Almathen,F., Yau,E., Ng,B.C., Webby,R.J., Poon,L.L., Peiris,M., Chu,D.K.W., Perera,R.A.P.M., Chan,S.M.S., Ng,B.C.Y., Poon,L.L.M. | 2018-05-31T00:00:00Z | ssRNA(+) | 1126 | spike glycoprotein | Saudi Arabia | Camelus dromedarius | |
| ASU45818.1 | Paden,C.R., Yusof,M.F.B.M., Al Hammadi,Z.M., Queen,K., Tao,Y., Eltahir,Y.M., Elsayed,E.A., Marzoug,B.A., Bensalah,O.K.A., Khalafalla,A.I., Al Mulla,M., Elkheir,K.A., Issa,Z.B., Pradeep,K., Elsaleh,F.N., Imambaccus,H., Sasse,J., Weber,S., Shi,M., Zhang,J., Li,Y., Pham,H., Kim,L., Hall,A.J., Gerber,S.I., Al Hosani,F.I., Tong,S., Al Muhairi,S.S.M., Al Muhairi,S.S., Al Hammadi,Z., Abou Elkheir,K., Al Bandar,Z., El Saleh,F., Hall,A., Gerber,S. | 2017-12-20T00:00:00Z | ssRNA(+) | 1353 | spike glycoprotein | United Arab Emirates | Camelus | |
| ASU45829.1 | Paden,C.R., Yusof,M.F.B.M., Al Hammadi,Z.M., Queen,K., Tao,Y., Eltahir,Y.M., Elsayed,E.A., Marzoug,B.A., Bensalah,O.K.A., Khalafalla,A.I., Al Mulla,M., Elkheir,K.A., Issa,Z.B., Pradeep,K., Elsaleh,F.N., Imambaccus,H., Sasse,J., Weber,S., Shi,M., Zhang,J., Li,Y., Pham,H., Kim,L., Hall,A.J., Gerber,S.I., Al Hosani,F.I., Tong,S., Al Muhairi,S.S.M., Al Muhairi,S.S., Al Hammadi,Z., Abou Elkheir,K., Al Bandar,Z., El Saleh,F., Hall,A., Gerber,S. | 2017-12-20T00:00:00Z | ssRNA(+) | 1353 | spike glycoprotein | United Arab Emirates | Camelus | |
| ASU45840.1 | Paden,C.R., Yusof,M.F.B.M., Al Hammadi,Z.M., Queen,K., Tao,Y., Eltahir,Y.M., Elsayed,E.A., Marzoug,B.A., Bensalah,O.K.A., Khalafalla,A.I., Al Mulla,M., Elkheir,K.A., Issa,Z.B., Pradeep,K., Elsaleh,F.N., Imambaccus,H., Sasse,J., Weber,S., Shi,M., Zhang,J., Li,Y., Pham,H., Kim,L., Hall,A.J., Gerber,S.I., Al Hosani,F.I., Tong,S., Al Muhairi,S.S.M., Al Muhairi,S.S., Al Hammadi,Z., Abou Elkheir,K., Al Bandar,Z., El Saleh,F., Hall,A., Gerber,S. | 2017-12-20T00:00:00Z | ssRNA(+) | 1353 | spike glycoprotein | United Arab Emirates | Camelus | |
| ASU45851.1 | Paden,C.R., Yusof,M.F.B.M., Al Hammadi,Z.M., Queen,K., Tao,Y., Eltahir,Y.M., Elsayed,E.A., Marzoug,B.A., Bensalah,O.K.A., Khalafalla,A.I., Al Mulla,M., Elkheir,K.A., Issa,Z.B., Pradeep,K., Elsaleh,F.N., Imambaccus,H., Sasse,J., Weber,S., Shi,M., Zhang,J., Li,Y., Pham,H., Kim,L., Hall,A.J., Gerber,S.I., Al Hosani,F.I., Tong,S., Al Muhairi,S.S.M., Al Muhairi,S.S., Al Hammadi,Z., Abou Elkheir,K., Al Bandar,Z., El Saleh,F., Hall,A., Gerber,S. | 2017-12-20T00:00:00Z | ssRNA(+) | 1353 | spike glycoprotein | United Arab Emirates | Camelus | |
| ASU45862.1 | Paden,C.R., Yusof,M.F.B.M., Al Hammadi,Z.M., Queen,K., Tao,Y., Eltahir,Y.M., Elsayed,E.A., Marzoug,B.A., Bensalah,O.K.A., Khalafalla,A.I., Al Mulla,M., Elkheir,K.A., Issa,Z.B., Pradeep,K., Elsaleh,F.N., Imambaccus,H., Sasse,J., Weber,S., Shi,M., Zhang,J., Li,Y., Pham,H., Kim,L., Hall,A.J., Gerber,S.I., Al Hosani,F.I., Tong,S., Al Muhairi,S.S.M., Al Muhairi,S.S., Al Hammadi,Z., Abou Elkheir,K., Al Bandar,Z., El Saleh,F., Hall,A., Gerber,S. | 2017-12-20T00:00:00Z | ssRNA(+) | 1353 | spike glycoprotein | United Arab Emirates | Camelus | |
| ASU45873.1 | Paden,C.R., Yusof,M.F.B.M., Al Hammadi,Z.M., Queen,K., Tao,Y., Eltahir,Y.M., Elsayed,E.A., Marzoug,B.A., Bensalah,O.K.A., Khalafalla,A.I., Al Mulla,M., Elkheir,K.A., Issa,Z.B., Pradeep,K., Elsaleh,F.N., Imambaccus,H., Sasse,J., Weber,S., Shi,M., Zhang,J., Li,Y., Pham,H., Kim,L., Hall,A.J., Gerber,S.I., Al Hosani,F.I., Tong,S., Al Muhairi,S.S.M., Al Muhairi,S.S., Al Hammadi,Z., Abou Elkheir,K., Al Bandar,Z., El Saleh,F., Hall,A., Gerber,S. | 2017-12-20T00:00:00Z | ssRNA(+) | 1353 | spike glycoprotein | United Arab Emirates | Camelus | |
| ASU89921.1 | Yusof,M.F., Queen,K., Eltahir,Y.M., Paden,C.R., Al Hammadi,Z.M.A.H., Tao,Y., Li,Y., Khalafalla,A.I., Shi,M., Zhang,J., Mohamed,M.S.A.E., Abd Elaal Ahmed,M.H., Azeez,I.A., Bensalah,O.K., Eldahab,Z.S., Al Hosani,F.I., Gerber,S.I., Hall,A.J., Tong,S., Al Muhairi,S.S., Al Hammadi,Z.M., Mohamed,M.S., Ahmed,M.H. | 2017-11-15T00:00:00Z | ssRNA(+) | 1353 | spike glycoprotein | United Arab Emirates | Camelus dromedarius | |
| ASU89933.1 | Yusof,M.F., Queen,K., Eltahir,Y.M., Paden,C.R., Al Hammadi,Z.M.A.H., Tao,Y., Li,Y., Khalafalla,A.I., Shi,M., Zhang,J., Mohamed,M.S.A.E., Abd Elaal Ahmed,M.H., Azeez,I.A., Bensalah,O.K., Eldahab,Z.S., Al Hosani,F.I., Gerber,S.I., Hall,A.J., Tong,S., Al Muhairi,S.S., Al Hammadi,Z.M., Mohamed,M.S., Ahmed,M.H. | 2017-11-15T00:00:00Z | ssRNA(+) | 1353 | spike glycoprotein | United Arab Emirates | Camelus dromedarius | |
| ASU89944.1 | Yusof,M.F., Queen,K., Eltahir,Y.M., Paden,C.R., Al Hammadi,Z.M.A.H., Tao,Y., Li,Y., Khalafalla,A.I., Shi,M., Zhang,J., Mohamed,M.S.A.E., Abd Elaal Ahmed,M.H., Azeez,I.A., Bensalah,O.K., Eldahab,Z.S., Al Hosani,F.I., Gerber,S.I., Hall,A.J., Tong,S., Al Muhairi,S.S., Al Hammadi,Z.M., Mohamed,M.S., Ahmed,M.H. | 2017-11-15T00:00:00Z | ssRNA(+) | 1353 | spike glycoprotein | United Arab Emirates | Camelus dromedarius | |
| ASU89955.1 | Yusof,M.F., Queen,K., Eltahir,Y.M., Paden,C.R., Al Hammadi,Z.M.A.H., Tao,Y., Li,Y., Khalafalla,A.I., Shi,M., Zhang,J., Mohamed,M.S.A.E., Abd Elaal Ahmed,M.H., Azeez,I.A., Bensalah,O.K., Eldahab,Z.S., Al Hosani,F.I., Gerber,S.I., Hall,A.J., Tong,S., Al Muhairi,S.S., Al Hammadi,Z.M., Mohamed,M.S., Ahmed,M.H. | 2017-11-15T00:00:00Z | ssRNA(+) | 1353 | spike glycoprotein | United Arab Emirates | Camelus dromedarius | |
| ASU89966.1 | Yusof,M.F., Queen,K., Eltahir,Y.M., Paden,C.R., Al Hammadi,Z.M.A.H., Tao,Y., Li,Y., Khalafalla,A.I., Shi,M., Zhang,J., Mohamed,M.S.A.E., Abd Elaal Ahmed,M.H., Azeez,I.A., Bensalah,O.K., Eldahab,Z.S., Al Hosani,F.I., Gerber,S.I., Hall,A.J., Tong,S., Al Muhairi,S.S., Al Hammadi,Z.M., Mohamed,M.S., Ahmed,M.H. | 2017-11-15T00:00:00Z | ssRNA(+) | 1353 | spike glycoprotein | United Arab Emirates | Camelus dromedarius | |
| ASU89977.1 | Yusof,M.F., Queen,K., Eltahir,Y.M., Paden,C.R., Al Hammadi,Z.M.A.H., Tao,Y., Li,Y., Khalafalla,A.I., Shi,M., Zhang,J., Mohamed,M.S.A.E., Abd Elaal Ahmed,M.H., Azeez,I.A., Bensalah,O.K., Eldahab,Z.S., Al Hosani,F.I., Gerber,S.I., Hall,A.J., Tong,S., Al Muhairi,S.S., Al Hammadi,Z.M., Mohamed,M.S., Ahmed,M.H. | 2017-11-15T00:00:00Z | ssRNA(+) | 1353 | spike glycoprotein | United Arab Emirates | Camelus dromedarius | |
| ASU89988.1 | Yusof,M.F., Queen,K., Eltahir,Y.M., Paden,C.R., Al Hammadi,Z.M.A.H., Tao,Y., Li,Y., Khalafalla,A.I., Shi,M., Zhang,J., Mohamed,M.S.A.E., Abd Elaal Ahmed,M.H., Azeez,I.A., Bensalah,O.K., Eldahab,Z.S., Al Hosani,F.I., Gerber,S.I., Hall,A.J., Tong,S., Al Muhairi,S.S., Al Hammadi,Z.M., Mohamed,M.S., Ahmed,M.H. | 2017-11-15T00:00:00Z | ssRNA(+) | 1353 | spike glycoprotein | United Arab Emirates | Camelus dromedarius | |
| ASU89999.1 | Yusof,M.F., Queen,K., Eltahir,Y.M., Paden,C.R., Al Hammadi,Z.M.A.H., Tao,Y., Li,Y., Khalafalla,A.I., Shi,M., Zhang,J., Mohamed,M.S.A.E., Abd Elaal Ahmed,M.H., Azeez,I.A., Bensalah,O.K., Eldahab,Z.S., Al Hosani,F.I., Gerber,S.I., Hall,A.J., Tong,S., Al Muhairi,S.S., Al Hammadi,Z.M., Mohamed,M.S., Ahmed,M.H. | 2017-11-15T00:00:00Z | ssRNA(+) | 1353 | spike glycoprotein | United Arab Emirates | Camelus dromedarius | |
| ASU90010.1 | Yusof,M.F., Queen,K., Eltahir,Y.M., Paden,C.R., Al Hammadi,Z.M.A.H., Tao,Y., Li,Y., Khalafalla,A.I., Shi,M., Zhang,J., Mohamed,M.S.A.E., Abd Elaal Ahmed,M.H., Azeez,I.A., Bensalah,O.K., Eldahab,Z.S., Al Hosani,F.I., Gerber,S.I., Hall,A.J., Tong,S., Al Muhairi,S.S., Al Hammadi,Z.M., Mohamed,M.S., Ahmed,M.H. | 2017-11-15T00:00:00Z | ssRNA(+) | 1353 | spike glycoprotein | United Arab Emirates | Camelus dromedarius | |
| ASU90021.1 | Yusof,M.F., Queen,K., Eltahir,Y.M., Paden,C.R., Al Hammadi,Z.M.A.H., Tao,Y., Li,Y., Khalafalla,A.I., Shi,M., Zhang,J., Mohamed,M.S.A.E., Abd Elaal Ahmed,M.H., Azeez,I.A., Bensalah,O.K., Eldahab,Z.S., Al Hosani,F.I., Gerber,S.I., Hall,A.J., Tong,S., Al Muhairi,S.S., Al Hammadi,Z.M., Mohamed,M.S., Ahmed,M.H. | 2017-11-15T00:00:00Z | ssRNA(+) | 1353 | spike glycoprotein | United Arab Emirates | Camelus dromedarius | |
| ASU90032.1 | Yusof,M.F., Queen,K., Eltahir,Y.M., Paden,C.R., Al Hammadi,Z.M.A.H., Tao,Y., Li,Y., Khalafalla,A.I., Shi,M., Zhang,J., Mohamed,M.S.A.E., Abd Elaal Ahmed,M.H., Azeez,I.A., Bensalah,O.K., Eldahab,Z.S., Al Hosani,F.I., Gerber,S.I., Hall,A.J., Tong,S., Al Muhairi,S.S., Al Hammadi,Z.M., Mohamed,M.S., Ahmed,M.H. | 2017-11-15T00:00:00Z | ssRNA(+) | 1353 | spike glycoprotein | United Arab Emirates | Camelus dromedarius | |
| ASU90043.1 | Yusof,M.F., Queen,K., Eltahir,Y.M., Paden,C.R., Al Hammadi,Z.M.A.H., Tao,Y., Li,Y., Khalafalla,A.I., Shi,M., Zhang,J., Mohamed,M.S.A.E., Abd Elaal Ahmed,M.H., Azeez,I.A., Bensalah,O.K., Eldahab,Z.S., Al Hosani,F.I., Gerber,S.I., Hall,A.J., Tong,S., Al Muhairi,S.S., Al Hammadi,Z.M., Mohamed,M.S., Ahmed,M.H. | 2017-11-15T00:00:00Z | ssRNA(+) | 1353 | spike glycoprotein | United Arab Emirates | Camelus dromedarius | |
| ASU90054.1 | Yusof,M.F., Queen,K., Eltahir,Y.M., Paden,C.R., Al Hammadi,Z.M.A.H., Tao,Y., Li,Y., Khalafalla,A.I., Shi,M., Zhang,J., Mohamed,M.S.A.E., Abd Elaal Ahmed,M.H., Azeez,I.A., Bensalah,O.K., Eldahab,Z.S., Al Hosani,F.I., Gerber,S.I., Hall,A.J., Tong,S., Al Muhairi,S.S., Al Hammadi,Z.M., Mohamed,M.S., Ahmed,M.H. | 2017-11-15T00:00:00Z | ssRNA(+) | 1353 | spike glycoprotein | United Arab Emirates | Camelus dromedarius | |
| ASU90065.1 | Yusof,M.F., Queen,K., Eltahir,Y.M., Paden,C.R., Al Hammadi,Z.M.A.H., Tao,Y., Li,Y., Khalafalla,A.I., Shi,M., Zhang,J., Mohamed,M.S.A.E., Abd Elaal Ahmed,M.H., Azeez,I.A., Bensalah,O.K., Eldahab,Z.S., Al Hosani,F.I., Gerber,S.I., Hall,A.J., Tong,S., Al Muhairi,S.S., Al Hammadi,Z.M., Mohamed,M.S., Ahmed,M.H. | 2017-11-15T00:00:00Z | ssRNA(+) | 1353 | spike glycoprotein | United Arab Emirates | Camelus dromedarius | |
| ASU90076.1 | Yusof,M.F., Queen,K., Eltahir,Y.M., Paden,C.R., Al Hammadi,Z.M.A.H., Tao,Y., Li,Y., Khalafalla,A.I., Shi,M., Zhang,J., Mohamed,M.S.A.E., Abd Elaal Ahmed,M.H., Azeez,I.A., Bensalah,O.K., Eldahab,Z.S., Al Hosani,F.I., Gerber,S.I., Hall,A.J., Tong,S., Al Muhairi,S.S., Al Hammadi,Z.M., Mohamed,M.S., Ahmed,M.H. | 2017-11-15T00:00:00Z | ssRNA(+) | 1353 | spike glycoprotein | United Arab Emirates | Camelus dromedarius | |
| ASU90087.1 | Yusof,M.F., Queen,K., Eltahir,Y.M., Paden,C.R., Al Hammadi,Z.M.A.H., Tao,Y., Li,Y., Khalafalla,A.I., Shi,M., Zhang,J., Mohamed,M.S.A.E., Abd Elaal Ahmed,M.H., Azeez,I.A., Bensalah,O.K., Eldahab,Z.S., Al Hosani,F.I., Gerber,S.I., Hall,A.J., Tong,S., Al Muhairi,S.S., Al Hammadi,Z.M., Mohamed,M.S., Ahmed,M.H. | 2017-11-15T00:00:00Z | ssRNA(+) | 1353 | spike glycoprotein | United Arab Emirates | Camelus dromedarius | |
| ASU90098.1 | Yusof,M.F., Queen,K., Eltahir,Y.M., Paden,C.R., Al Hammadi,Z.M.A.H., Tao,Y., Li,Y., Khalafalla,A.I., Shi,M., Zhang,J., Mohamed,M.S.A.E., Abd Elaal Ahmed,M.H., Azeez,I.A., Bensalah,O.K., Eldahab,Z.S., Al Hosani,F.I., Gerber,S.I., Hall,A.J., Tong,S., Al Muhairi,S.S., Al Hammadi,Z.M., Mohamed,M.S., Ahmed,M.H. | 2017-11-15T00:00:00Z | ssRNA(+) | 1353 | spike glycoprotein | United Arab Emirates | Camelus dromedarius | |
| ASU90109.1 | Yusof,M.F., Queen,K., Eltahir,Y.M., Paden,C.R., Al Hammadi,Z.M.A.H., Tao,Y., Li,Y., Khalafalla,A.I., Shi,M., Zhang,J., Mohamed,M.S.A.E., Abd Elaal Ahmed,M.H., Azeez,I.A., Bensalah,O.K., Eldahab,Z.S., Al Hosani,F.I., Gerber,S.I., Hall,A.J., Tong,S., Al Muhairi,S.S., Al Hammadi,Z.M., Mohamed,M.S., Ahmed,M.H. | 2017-11-15T00:00:00Z | ssRNA(+) | 1353 | spike glycoprotein | United Arab Emirates | Camelus dromedarius | |
| ASU90120.1 | Yusof,M.F., Queen,K., Eltahir,Y.M., Paden,C.R., Al Hammadi,Z.M.A.H., Tao,Y., Li,Y., Khalafalla,A.I., Shi,M., Zhang,J., Mohamed,M.S.A.E., Abd Elaal Ahmed,M.H., Azeez,I.A., Bensalah,O.K., Eldahab,Z.S., Al Hosani,F.I., Gerber,S.I., Hall,A.J., Tong,S., Al Muhairi,S.S., Al Hammadi,Z.M., Mohamed,M.S., Ahmed,M.H. | 2017-11-15T00:00:00Z | ssRNA(+) | 1353 | spike glycoprotein | United Arab Emirates | Camelus dromedarius | |
| ASU90131.1 | Yusof,M.F., Queen,K., Eltahir,Y.M., Paden,C.R., Al Hammadi,Z.M.A.H., Tao,Y., Li,Y., Khalafalla,A.I., Shi,M., Zhang,J., Mohamed,M.S.A.E., Abd Elaal Ahmed,M.H., Azeez,I.A., Bensalah,O.K., Eldahab,Z.S., Al Hosani,F.I., Gerber,S.I., Hall,A.J., Tong,S., Al Muhairi,S.S., Al Hammadi,Z.M., Mohamed,M.S., Ahmed,M.H. | 2017-11-15T00:00:00Z | ssRNA(+) | 1353 | spike glycoprotein | United Arab Emirates | Camelus dromedarius | |
| ASU90142.1 | Yusof,M.F., Queen,K., Eltahir,Y.M., Paden,C.R., Al Hammadi,Z.M.A.H., Tao,Y., Li,Y., Khalafalla,A.I., Shi,M., Zhang,J., Mohamed,M.S.A.E., Abd Elaal Ahmed,M.H., Azeez,I.A., Bensalah,O.K., Eldahab,Z.S., Al Hosani,F.I., Gerber,S.I., Hall,A.J., Tong,S., Al Muhairi,S.S., Al Hammadi,Z.M., Mohamed,M.S., Ahmed,M.H. | 2017-11-15T00:00:00Z | ssRNA(+) | 1353 | spike glycoprotein | United Arab Emirates | Camelus dromedarius | |
| ASU90153.1 | Yusof,M.F., Queen,K., Eltahir,Y.M., Paden,C.R., Al Hammadi,Z.M.A.H., Tao,Y., Li,Y., Khalafalla,A.I., Shi,M., Zhang,J., Mohamed,M.S.A.E., Abd Elaal Ahmed,M.H., Azeez,I.A., Bensalah,O.K., Eldahab,Z.S., Al Hosani,F.I., Gerber,S.I., Hall,A.J., Tong,S., Al Muhairi,S.S., Al Hammadi,Z.M., Mohamed,M.S., Ahmed,M.H. | 2017-11-15T00:00:00Z | ssRNA(+) | 1353 | spike glycoprotein | United Arab Emirates | Camelus dromedarius | |
| ASU90164.1 | Yusof,M.F., Queen,K., Eltahir,Y.M., Paden,C.R., Al Hammadi,Z.M.A.H., Tao,Y., Li,Y., Khalafalla,A.I., Shi,M., Zhang,J., Mohamed,M.S.A.E., Abd Elaal Ahmed,M.H., Azeez,I.A., Bensalah,O.K., Eldahab,Z.S., Al Hosani,F.I., Gerber,S.I., Hall,A.J., Tong,S., Al Muhairi,S.S., Al Hammadi,Z.M., Mohamed,M.S., Ahmed,M.H. | 2017-11-15T00:00:00Z | ssRNA(+) | 1353 | spike glycoprotein | United Arab Emirates | Camelus dromedarius | |
| ASU90175.1 | Yusof,M.F., Queen,K., Eltahir,Y.M., Paden,C.R., Al Hammadi,Z.M.A.H., Tao,Y., Li,Y., Khalafalla,A.I., Shi,M., Zhang,J., Mohamed,M.S.A.E., Abd Elaal Ahmed,M.H., Azeez,I.A., Bensalah,O.K., Eldahab,Z.S., Al Hosani,F.I., Gerber,S.I., Hall,A.J., Tong,S., Al Muhairi,S.S., Al Hammadi,Z.M., Mohamed,M.S., Ahmed,M.H. | 2017-11-15T00:00:00Z | ssRNA(+) | 1353 | spike glycoprotein | United Arab Emirates | Camelus dromedarius | |
| ASU90186.1 | Yusof,M.F., Queen,K., Eltahir,Y.M., Paden,C.R., Al Hammadi,Z.M.A.H., Tao,Y., Li,Y., Khalafalla,A.I., Shi,M., Zhang,J., Mohamed,M.S.A.E., Abd Elaal Ahmed,M.H., Azeez,I.A., Bensalah,O.K., Eldahab,Z.S., Al Hosani,F.I., Gerber,S.I., Hall,A.J., Tong,S., Al Muhairi,S.S., Al Hammadi,Z.M., Mohamed,M.S., Ahmed,M.H. | 2017-11-15T00:00:00Z | ssRNA(+) | 1353 | spike glycoprotein | United Arab Emirates | Camelus dromedarius | |
| ASU90197.1 | Yusof,M.F., Queen,K., Eltahir,Y.M., Paden,C.R., Al Hammadi,Z.M.A.H., Tao,Y., Li,Y., Khalafalla,A.I., Shi,M., Zhang,J., Mohamed,M.S.A.E., Abd Elaal Ahmed,M.H., Azeez,I.A., Bensalah,O.K., Eldahab,Z.S., Al Hosani,F.I., Gerber,S.I., Hall,A.J., Tong,S., Al Muhairi,S.S., Al Hammadi,Z.M., Mohamed,M.S., Ahmed,M.H. | 2017-11-15T00:00:00Z | ssRNA(+) | 1353 | spike glycoprotein | United Arab Emirates | Camelus dromedarius | |
| ASU90208.1 | Yusof,M.F., Queen,K., Eltahir,Y.M., Paden,C.R., Al Hammadi,Z.M.A.H., Tao,Y., Li,Y., Khalafalla,A.I., Shi,M., Zhang,J., Mohamed,M.S.A.E., Abd Elaal Ahmed,M.H., Azeez,I.A., Bensalah,O.K., Eldahab,Z.S., Al Hosani,F.I., Gerber,S.I., Hall,A.J., Tong,S., Al Muhairi,S.S., Al Hammadi,Z.M., Mohamed,M.S., Ahmed,M.H. | 2017-11-15T00:00:00Z | ssRNA(+) | 1353 | spike glycoprotein | United Arab Emirates | Camelus dromedarius | |
| ASU90219.1 | Yusof,M.F., Queen,K., Eltahir,Y.M., Paden,C.R., Al Hammadi,Z.M.A.H., Tao,Y., Li,Y., Khalafalla,A.I., Shi,M., Zhang,J., Mohamed,M.S.A.E., Abd Elaal Ahmed,M.H., Azeez,I.A., Bensalah,O.K., Eldahab,Z.S., Al Hosani,F.I., Gerber,S.I., Hall,A.J., Tong,S., Al Muhairi,S.S., Al Hammadi,Z.M., Mohamed,M.S., Ahmed,M.H. | 2017-11-15T00:00:00Z | ssRNA(+) | 1353 | spike glycoprotein | United Arab Emirates | Camelus dromedarius | |
| ASU90230.1 | Yusof,M.F., Queen,K., Eltahir,Y.M., Paden,C.R., Al Hammadi,Z.M.A.H., Tao,Y., Li,Y., Khalafalla,A.I., Shi,M., Zhang,J., Mohamed,M.S.A.E., Abd Elaal Ahmed,M.H., Azeez,I.A., Bensalah,O.K., Eldahab,Z.S., Al Hosani,F.I., Gerber,S.I., Hall,A.J., Tong,S., Al Muhairi,S.S., Al Hammadi,Z.M., Mohamed,M.S., Ahmed,M.H. | 2017-11-15T00:00:00Z | ssRNA(+) | 1353 | spike glycoprotein | United Arab Emirates | Camelus dromedarius | |
| ASU90241.1 | Yusof,M.F., Queen,K., Eltahir,Y.M., Paden,C.R., Al Hammadi,Z.M.A.H., Tao,Y., Li,Y., Khalafalla,A.I., Shi,M., Zhang,J., Mohamed,M.S.A.E., Abd Elaal Ahmed,M.H., Azeez,I.A., Bensalah,O.K., Eldahab,Z.S., Al Hosani,F.I., Gerber,S.I., Hall,A.J., Tong,S., Al Muhairi,S.S., Al Hammadi,Z.M., Mohamed,M.S., Ahmed,M.H. | 2017-11-15T00:00:00Z | ssRNA(+) | 1353 | spike glycoprotein | United Arab Emirates | Camelus dromedarius | |
| ASU90252.1 | Yusof,M.F., Queen,K., Eltahir,Y.M., Paden,C.R., Al Hammadi,Z.M.A.H., Tao,Y., Li,Y., Khalafalla,A.I., Shi,M., Zhang,J., Mohamed,M.S.A.E., Abd Elaal Ahmed,M.H., Azeez,I.A., Bensalah,O.K., Eldahab,Z.S., Al Hosani,F.I., Gerber,S.I., Hall,A.J., Tong,S., Al Muhairi,S.S., Al Hammadi,Z.M., Mohamed,M.S., Ahmed,M.H. | 2017-11-15T00:00:00Z | ssRNA(+) | 1353 | spike glycoprotein | United Arab Emirates | Camelus dromedarius | |
| ASU90263.1 | Yusof,M.F., Queen,K., Eltahir,Y.M., Paden,C.R., Al Hammadi,Z.M.A.H., Tao,Y., Li,Y., Khalafalla,A.I., Shi,M., Zhang,J., Mohamed,M.S.A.E., Abd Elaal Ahmed,M.H., Azeez,I.A., Bensalah,O.K., Eldahab,Z.S., Al Hosani,F.I., Gerber,S.I., Hall,A.J., Tong,S., Al Muhairi,S.S., Al Hammadi,Z.M., Mohamed,M.S., Ahmed,M.H. | 2017-11-15T00:00:00Z | ssRNA(+) | 1353 | spike glycoprotein | United Arab Emirates | Camelus dromedarius | |
| ASU90274.1 | Yusof,M.F., Queen,K., Eltahir,Y.M., Paden,C.R., Al Hammadi,Z.M.A.H., Tao,Y., Li,Y., Khalafalla,A.I., Shi,M., Zhang,J., Mohamed,M.S.A.E., Abd Elaal Ahmed,M.H., Azeez,I.A., Bensalah,O.K., Eldahab,Z.S., Al Hosani,F.I., Gerber,S.I., Hall,A.J., Tong,S., Al Muhairi,S.S., Al Hammadi,Z.M., Mohamed,M.S., Ahmed,M.H. | 2017-11-15T00:00:00Z | ssRNA(+) | 1353 | spike glycoprotein | United Arab Emirates | Camelus dromedarius | |
| ASU90285.1 | Yusof,M.F., Queen,K., Eltahir,Y.M., Paden,C.R., Al Hammadi,Z.M.A.H., Tao,Y., Li,Y., Khalafalla,A.I., Shi,M., Zhang,J., Mohamed,M.S.A.E., Abd Elaal Ahmed,M.H., Azeez,I.A., Bensalah,O.K., Eldahab,Z.S., Al Hosani,F.I., Gerber,S.I., Hall,A.J., Tong,S., Al Muhairi,S.S., Al Hammadi,Z.M., Mohamed,M.S., Ahmed,M.H. | 2017-11-15T00:00:00Z | ssRNA(+) | 1353 | spike glycoprotein | United Arab Emirates | Camelus dromedarius | |
| ASU90295.1 | Yusof,M.F., Queen,K., Eltahir,Y.M., Paden,C.R., Al Hammadi,Z.M.A.H., Tao,Y., Li,Y., Khalafalla,A.I., Shi,M., Zhang,J., Mohamed,M.S.A.E., Abd Elaal Ahmed,M.H., Azeez,I.A., Bensalah,O.K., Eldahab,Z.S., Al Hosani,F.I., Gerber,S.I., Hall,A.J., Tong,S., Al Muhairi,S.S., Al Hammadi,Z.M., Mohamed,M.S., Ahmed,M.H. | 2017-11-15T00:00:00Z | ssRNA(+) | 1353 | spike glycoprotein | United Arab Emirates | Camelus dromedarius | |
| ASU90307.1 | Yusof,M.F., Queen,K., Eltahir,Y.M., Paden,C.R., Al Hammadi,Z.M.A.H., Tao,Y., Li,Y., Khalafalla,A.I., Shi,M., Zhang,J., Mohamed,M.S.A.E., Abd Elaal Ahmed,M.H., Azeez,I.A., Bensalah,O.K., Eldahab,Z.S., Al Hosani,F.I., Gerber,S.I., Hall,A.J., Tong,S., Al Muhairi,S.S., Al Hammadi,Z.M., Mohamed,M.S., Ahmed,M.H. | 2017-11-15T00:00:00Z | ssRNA(+) | 1353 | spike glycoprotein | United Arab Emirates | Camelus dromedarius | |
| ASU90318.1 | Yusof,M.F., Queen,K., Eltahir,Y.M., Paden,C.R., Al Hammadi,Z.M.A.H., Tao,Y., Li,Y., Khalafalla,A.I., Shi,M., Zhang,J., Mohamed,M.S.A.E., Abd Elaal Ahmed,M.H., Azeez,I.A., Bensalah,O.K., Eldahab,Z.S., Al Hosani,F.I., Gerber,S.I., Hall,A.J., Tong,S., Al Muhairi,S.S., Al Hammadi,Z.M., Mohamed,M.S., Ahmed,M.H. | 2017-11-15T00:00:00Z | ssRNA(+) | 1353 | spike glycoprotein | United Arab Emirates | Camelus dromedarius | |
| ASU90329.1 | Yusof,M.F., Queen,K., Eltahir,Y.M., Paden,C.R., Al Hammadi,Z.M.A.H., Tao,Y., Li,Y., Khalafalla,A.I., Shi,M., Zhang,J., Mohamed,M.S.A.E., Abd Elaal Ahmed,M.H., Azeez,I.A., Bensalah,O.K., Eldahab,Z.S., Al Hosani,F.I., Gerber,S.I., Hall,A.J., Tong,S., Al Muhairi,S.S., Al Hammadi,Z.M., Mohamed,M.S., Ahmed,M.H. | 2017-11-15T00:00:00Z | ssRNA(+) | 1353 | spike glycoprotein | United Arab Emirates | Camelus dromedarius | |
| ASU90340.1 | Yusof,M.F., Queen,K., Eltahir,Y.M., Paden,C.R., Al Hammadi,Z.M.A.H., Tao,Y., Li,Y., Khalafalla,A.I., Shi,M., Zhang,J., Mohamed,M.S.A.E., Abd Elaal Ahmed,M.H., Azeez,I.A., Bensalah,O.K., Eldahab,Z.S., Al Hosani,F.I., Gerber,S.I., Hall,A.J., Tong,S., Al Muhairi,S.S., Al Hammadi,Z.M., Mohamed,M.S., Ahmed,M.H. | 2017-11-15T00:00:00Z | ssRNA(+) | 1353 | spike glycoprotein | United Arab Emirates | Camelus dromedarius | |
| ASU90351.1 | Yusof,M.F., Queen,K., Eltahir,Y.M., Paden,C.R., Al Hammadi,Z.M.A.H., Tao,Y., Li,Y., Khalafalla,A.I., Shi,M., Zhang,J., Mohamed,M.S.A.E., Abd Elaal Ahmed,M.H., Azeez,I.A., Bensalah,O.K., Eldahab,Z.S., Al Hosani,F.I., Gerber,S.I., Hall,A.J., Tong,S., Al Muhairi,S.S., Al Hammadi,Z.M., Mohamed,M.S., Ahmed,M.H. | 2017-11-15T00:00:00Z | ssRNA(+) | 1353 | spike glycoprotein | United Arab Emirates | Camelus dromedarius | |
| ASU90362.1 | Yusof,M.F., Queen,K., Eltahir,Y.M., Paden,C.R., Al Hammadi,Z.M.A.H., Tao,Y., Li,Y., Khalafalla,A.I., Shi,M., Zhang,J., Mohamed,M.S.A.E., Abd Elaal Ahmed,M.H., Azeez,I.A., Bensalah,O.K., Eldahab,Z.S., Al Hosani,F.I., Gerber,S.I., Hall,A.J., Tong,S., Al Muhairi,S.S., Al Hammadi,Z.M., Mohamed,M.S., Ahmed,M.H. | 2017-11-15T00:00:00Z | ssRNA(+) | 1353 | spike glycoprotein | United Arab Emirates | Camelus dromedarius | |
| ASU90373.1 | Yusof,M.F., Queen,K., Eltahir,Y.M., Paden,C.R., Al Hammadi,Z.M.A.H., Tao,Y., Li,Y., Khalafalla,A.I., Shi,M., Zhang,J., Mohamed,M.S.A.E., Abd Elaal Ahmed,M.H., Azeez,I.A., Bensalah,O.K., Eldahab,Z.S., Al Hosani,F.I., Gerber,S.I., Hall,A.J., Tong,S., Al Muhairi,S.S., Al Hammadi,Z.M., Mohamed,M.S., Ahmed,M.H. | 2017-11-15T00:00:00Z | ssRNA(+) | 1353 | spike glycoprotein | United Arab Emirates | Camelus dromedarius | |
| ASU90384.1 | Yusof,M.F., Queen,K., Eltahir,Y.M., Paden,C.R., Al Hammadi,Z.M.A.H., Tao,Y., Li,Y., Khalafalla,A.I., Shi,M., Zhang,J., Mohamed,M.S.A.E., Abd Elaal Ahmed,M.H., Azeez,I.A., Bensalah,O.K., Eldahab,Z.S., Al Hosani,F.I., Gerber,S.I., Hall,A.J., Tong,S., Al Muhairi,S.S., Al Hammadi,Z.M., Mohamed,M.S., Ahmed,M.H. | 2017-11-15T00:00:00Z | ssRNA(+) | 1353 | spike glycoprotein | United Arab Emirates | Camelus dromedarius | |
| ASU90395.1 | Yusof,M.F., Queen,K., Eltahir,Y.M., Paden,C.R., Al Hammadi,Z.M.A.H., Tao,Y., Li,Y., Khalafalla,A.I., Shi,M., Zhang,J., Mohamed,M.S.A.E., Abd Elaal Ahmed,M.H., Azeez,I.A., Bensalah,O.K., Eldahab,Z.S., Al Hosani,F.I., Gerber,S.I., Hall,A.J., Tong,S., Al Muhairi,S.S., Al Hammadi,Z.M., Mohamed,M.S., Ahmed,M.H. | 2017-11-15T00:00:00Z | ssRNA(+) | 1353 | spike glycoprotein | United Arab Emirates | Camelus dromedarius | |
| ASU90406.1 | Yusof,M.F., Queen,K., Eltahir,Y.M., Paden,C.R., Al Hammadi,Z.M.A.H., Tao,Y., Li,Y., Khalafalla,A.I., Shi,M., Zhang,J., Mohamed,M.S.A.E., Abd Elaal Ahmed,M.H., Azeez,I.A., Bensalah,O.K., Eldahab,Z.S., Al Hosani,F.I., Gerber,S.I., Hall,A.J., Tong,S., Al Muhairi,S.S., Al Hammadi,Z.M., Mohamed,M.S., Ahmed,M.H. | 2017-11-15T00:00:00Z | ssRNA(+) | 1353 | spike glycoprotein | United Arab Emirates | Camelus dromedarius | |
| ASU90417.1 | Yusof,M.F., Queen,K., Eltahir,Y.M., Paden,C.R., Al Hammadi,Z.M.A.H., Tao,Y., Li,Y., Khalafalla,A.I., Shi,M., Zhang,J., Mohamed,M.S.A.E., Abd Elaal Ahmed,M.H., Azeez,I.A., Bensalah,O.K., Eldahab,Z.S., Al Hosani,F.I., Gerber,S.I., Hall,A.J., Tong,S., Al Muhairi,S.S., Al Hammadi,Z.M., Mohamed,M.S., Ahmed,M.H. | 2017-11-15T00:00:00Z | ssRNA(+) | 1353 | spike glycoprotein | United Arab Emirates | Camelus dromedarius | |
| ASU90428.1 | Yusof,M.F., Queen,K., Eltahir,Y.M., Paden,C.R., Al Hammadi,Z.M.A.H., Tao,Y., Li,Y., Khalafalla,A.I., Shi,M., Zhang,J., Mohamed,M.S.A.E., Abd Elaal Ahmed,M.H., Azeez,I.A., Bensalah,O.K., Eldahab,Z.S., Al Hosani,F.I., Gerber,S.I., Hall,A.J., Tong,S., Al Muhairi,S.S., Al Hammadi,Z.M., Mohamed,M.S., Ahmed,M.H. | 2017-11-15T00:00:00Z | ssRNA(+) | 1353 | spike glycoprotein | United Arab Emirates | Camelus dromedarius | |
| ASU90439.1 | Yusof,M.F., Queen,K., Eltahir,Y.M., Paden,C.R., Al Hammadi,Z.M.A.H., Tao,Y., Li,Y., Khalafalla,A.I., Shi,M., Zhang,J., Mohamed,M.S.A.E., Abd Elaal Ahmed,M.H., Azeez,I.A., Bensalah,O.K., Eldahab,Z.S., Al Hosani,F.I., Gerber,S.I., Hall,A.J., Tong,S., Al Muhairi,S.S., Al Hammadi,Z.M., Mohamed,M.S., Ahmed,M.H. | 2017-11-15T00:00:00Z | ssRNA(+) | 1353 | spike glycoprotein | United Arab Emirates | Camelus dromedarius | |
| ASU90450.1 | Yusof,M.F., Queen,K., Eltahir,Y.M., Paden,C.R., Al Hammadi,Z.M.A.H., Tao,Y., Li,Y., Khalafalla,A.I., Shi,M., Zhang,J., Mohamed,M.S.A.E., Abd Elaal Ahmed,M.H., Azeez,I.A., Bensalah,O.K., Eldahab,Z.S., Al Hosani,F.I., Gerber,S.I., Hall,A.J., Tong,S., Al Muhairi,S.S., Al Hammadi,Z.M., Mohamed,M.S., Ahmed,M.H. | 2017-11-15T00:00:00Z | ssRNA(+) | 1353 | spike glycoprotein | United Arab Emirates | Camelus dromedarius | |
| ASU90461.1 | Yusof,M.F., Queen,K., Eltahir,Y.M., Paden,C.R., Al Hammadi,Z.M.A.H., Tao,Y., Li,Y., Khalafalla,A.I., Shi,M., Zhang,J., Mohamed,M.S.A.E., Abd Elaal Ahmed,M.H., Azeez,I.A., Bensalah,O.K., Eldahab,Z.S., Al Hosani,F.I., Gerber,S.I., Hall,A.J., Tong,S., Al Muhairi,S.S., Al Hammadi,Z.M., Mohamed,M.S., Ahmed,M.H. | 2017-11-15T00:00:00Z | ssRNA(+) | 1353 | spike glycoprotein | United Arab Emirates | Camelus dromedarius | |
| ASU90472.1 | Yusof,M.F., Queen,K., Eltahir,Y.M., Paden,C.R., Al Hammadi,Z.M.A.H., Tao,Y., Li,Y., Khalafalla,A.I., Shi,M., Zhang,J., Mohamed,M.S.A.E., Abd Elaal Ahmed,M.H., Azeez,I.A., Bensalah,O.K., Eldahab,Z.S., Al Hosani,F.I., Gerber,S.I., Hall,A.J., Tong,S., Al Muhairi,S.S., Al Hammadi,Z.M., Mohamed,M.S., Ahmed,M.H. | 2017-11-15T00:00:00Z | ssRNA(+) | 1353 | spike glycoprotein | United Arab Emirates | Camelus dromedarius | |
| ASU90483.1 | Yusof,M.F., Queen,K., Eltahir,Y.M., Paden,C.R., Al Hammadi,Z.M.A.H., Tao,Y., Li,Y., Khalafalla,A.I., Shi,M., Zhang,J., Mohamed,M.S.A.E., Abd Elaal Ahmed,M.H., Azeez,I.A., Bensalah,O.K., Eldahab,Z.S., Al Hosani,F.I., Gerber,S.I., Hall,A.J., Tong,S., Al Muhairi,S.S., Al Hammadi,Z.M., Mohamed,M.S., Ahmed,M.H. | 2017-11-15T00:00:00Z | ssRNA(+) | 1353 | spike glycoprotein | United Arab Emirates | Camelus dromedarius | |
| ASU90494.1 | Yusof,M.F., Queen,K., Eltahir,Y.M., Paden,C.R., Al Hammadi,Z.M.A.H., Tao,Y., Li,Y., Khalafalla,A.I., Shi,M., Zhang,J., Mohamed,M.S.A.E., Abd Elaal Ahmed,M.H., Azeez,I.A., Bensalah,O.K., Eldahab,Z.S., Al Hosani,F.I., Gerber,S.I., Hall,A.J., Tong,S., Al Muhairi,S.S., Al Hammadi,Z.M., Mohamed,M.S., Ahmed,M.H. | 2017-11-15T00:00:00Z | ssRNA(+) | 1353 | spike glycoprotein | United Arab Emirates | Camelus dromedarius | |
| ASU90505.1 | Yusof,M.F., Queen,K., Eltahir,Y.M., Paden,C.R., Al Hammadi,Z.M.A.H., Tao,Y., Li,Y., Khalafalla,A.I., Shi,M., Zhang,J., Mohamed,M.S.A.E., Abd Elaal Ahmed,M.H., Azeez,I.A., Bensalah,O.K., Eldahab,Z.S., Al Hosani,F.I., Gerber,S.I., Hall,A.J., Tong,S., Al Muhairi,S.S., Al Hammadi,Z.M., Mohamed,M.S., Ahmed,M.H. | 2017-11-15T00:00:00Z | ssRNA(+) | 1353 | spike glycoprotein | United Arab Emirates | Camelus dromedarius | |
| ASU90516.1 | Yusof,M.F., Queen,K., Eltahir,Y.M., Paden,C.R., Al Hammadi,Z.M.A.H., Tao,Y., Li,Y., Khalafalla,A.I., Shi,M., Zhang,J., Mohamed,M.S.A.E., Abd Elaal Ahmed,M.H., Azeez,I.A., Bensalah,O.K., Eldahab,Z.S., Al Hosani,F.I., Gerber,S.I., Hall,A.J., Tong,S., Al Muhairi,S.S., Al Hammadi,Z.M., Mohamed,M.S., Ahmed,M.H. | 2017-11-15T00:00:00Z | ssRNA(+) | 1353 | spike glycoprotein | United Arab Emirates | Camelus dromedarius | |
| ASU90527.1 | Yusof,M.F., Queen,K., Eltahir,Y.M., Paden,C.R., Al Hammadi,Z.M.A.H., Tao,Y., Li,Y., Khalafalla,A.I., Shi,M., Zhang,J., Mohamed,M.S.A.E., Abd Elaal Ahmed,M.H., Azeez,I.A., Bensalah,O.K., Eldahab,Z.S., Al Hosani,F.I., Gerber,S.I., Hall,A.J., Tong,S., Al Muhairi,S.S., Al Hammadi,Z.M., Mohamed,M.S., Ahmed,M.H. | 2017-11-15T00:00:00Z | ssRNA(+) | 1353 | spike glycoprotein | United Arab Emirates | Camelus dromedarius | |
| ASU90538.1 | Yusof,M.F., Queen,K., Eltahir,Y.M., Paden,C.R., Al Hammadi,Z.M.A.H., Tao,Y., Li,Y., Khalafalla,A.I., Shi,M., Zhang,J., Mohamed,M.S.A.E., Abd Elaal Ahmed,M.H., Azeez,I.A., Bensalah,O.K., Eldahab,Z.S., Al Hosani,F.I., Gerber,S.I., Hall,A.J., Tong,S., Al Muhairi,S.S., Al Hammadi,Z.M., Mohamed,M.S., Ahmed,M.H. | 2017-11-15T00:00:00Z | ssRNA(+) | 1353 | spike glycoprotein | United Arab Emirates | Camelus dromedarius | |
| ASU90549.1 | Yusof,M.F., Queen,K., Eltahir,Y.M., Paden,C.R., Al Hammadi,Z.M.A.H., Tao,Y., Li,Y., Khalafalla,A.I., Shi,M., Zhang,J., Mohamed,M.S.A.E., Abd Elaal Ahmed,M.H., Azeez,I.A., Bensalah,O.K., Eldahab,Z.S., Al Hosani,F.I., Gerber,S.I., Hall,A.J., Tong,S., Al Muhairi,S.S., Al Hammadi,Z.M., Mohamed,M.S., Ahmed,M.H. | 2017-11-15T00:00:00Z | ssRNA(+) | 1353 | spike glycoprotein | United Arab Emirates | Camelus dromedarius | |
| ASU90560.1 | Yusof,M.F., Queen,K., Eltahir,Y.M., Paden,C.R., Al Hammadi,Z.M.A.H., Tao,Y., Li,Y., Khalafalla,A.I., Shi,M., Zhang,J., Mohamed,M.S.A.E., Abd Elaal Ahmed,M.H., Azeez,I.A., Bensalah,O.K., Eldahab,Z.S., Al Hosani,F.I., Gerber,S.I., Hall,A.J., Tong,S., Al Muhairi,S.S., Al Hammadi,Z.M., Mohamed,M.S., Ahmed,M.H. | 2017-11-15T00:00:00Z | ssRNA(+) | 1353 | spike glycoprotein | United Arab Emirates | Camelus dromedarius | |
| ASU90571.1 | Yusof,M.F., Queen,K., Eltahir,Y.M., Paden,C.R., Al Hammadi,Z.M.A.H., Tao,Y., Li,Y., Khalafalla,A.I., Shi,M., Zhang,J., Mohamed,M.S.A.E., Abd Elaal Ahmed,M.H., Azeez,I.A., Bensalah,O.K., Eldahab,Z.S., Al Hosani,F.I., Gerber,S.I., Hall,A.J., Tong,S., Al Muhairi,S.S., Al Hammadi,Z.M., Mohamed,M.S., Ahmed,M.H. | 2017-11-15T00:00:00Z | ssRNA(+) | 1353 | spike glycoprotein | United Arab Emirates | Camelus dromedarius | |
| ASU90582.1 | Yusof,M.F., Queen,K., Eltahir,Y.M., Paden,C.R., Al Hammadi,Z.M.A.H., Tao,Y., Li,Y., Khalafalla,A.I., Shi,M., Zhang,J., Mohamed,M.S.A.E., Abd Elaal Ahmed,M.H., Azeez,I.A., Bensalah,O.K., Eldahab,Z.S., Al Hosani,F.I., Gerber,S.I., Hall,A.J., Tong,S., Al Muhairi,S.S., Al Hammadi,Z.M., Mohamed,M.S., Ahmed,M.H. | 2017-11-15T00:00:00Z | ssRNA(+) | 1353 | spike glycoprotein | United Arab Emirates | Camelus dromedarius | |
| ASU90593.1 | Yusof,M.F., Queen,K., Eltahir,Y.M., Paden,C.R., Al Hammadi,Z.M.A.H., Tao,Y., Li,Y., Khalafalla,A.I., Shi,M., Zhang,J., Mohamed,M.S.A.E., Abd Elaal Ahmed,M.H., Azeez,I.A., Bensalah,O.K., Eldahab,Z.S., Al Hosani,F.I., Gerber,S.I., Hall,A.J., Tong,S., Al Muhairi,S.S., Al Hammadi,Z.M., Mohamed,M.S., Ahmed,M.H. | 2017-11-15T00:00:00Z | ssRNA(+) | 1353 | spike glycoprotein | United Arab Emirates | Camelus dromedarius | |
| ASU90604.1 | Yusof,M.F., Queen,K., Eltahir,Y.M., Paden,C.R., Al Hammadi,Z.M.A.H., Tao,Y., Li,Y., Khalafalla,A.I., Shi,M., Zhang,J., Mohamed,M.S.A.E., Abd Elaal Ahmed,M.H., Azeez,I.A., Bensalah,O.K., Eldahab,Z.S., Al Hosani,F.I., Gerber,S.I., Hall,A.J., Tong,S., Al Muhairi,S.S., Al Hammadi,Z.M., Mohamed,M.S., Ahmed,M.H. | 2017-11-15T00:00:00Z | ssRNA(+) | 1353 | spike glycoprotein | United Arab Emirates | Camelus dromedarius | |
| ASU90615.1 | Yusof,M.F., Queen,K., Eltahir,Y.M., Paden,C.R., Al Hammadi,Z.M.A.H., Tao,Y., Li,Y., Khalafalla,A.I., Shi,M., Zhang,J., Mohamed,M.S.A.E., Abd Elaal Ahmed,M.H., Azeez,I.A., Bensalah,O.K., Eldahab,Z.S., Al Hosani,F.I., Gerber,S.I., Hall,A.J., Tong,S., Al Muhairi,S.S., Al Hammadi,Z.M., Mohamed,M.S., Ahmed,M.H. | 2017-11-15T00:00:00Z | ssRNA(+) | 1353 | spike glycoprotein | United Arab Emirates | Camelus dromedarius | |
| ASU90626.1 | Yusof,M.F., Queen,K., Eltahir,Y.M., Paden,C.R., Al Hammadi,Z.M.A.H., Tao,Y., Li,Y., Khalafalla,A.I., Shi,M., Zhang,J., Mohamed,M.S.A.E., Abd Elaal Ahmed,M.H., Azeez,I.A., Bensalah,O.K., Eldahab,Z.S., Al Hosani,F.I., Gerber,S.I., Hall,A.J., Tong,S., Al Muhairi,S.S., Al Hammadi,Z.M., Mohamed,M.S., Ahmed,M.H. | 2017-11-15T00:00:00Z | ssRNA(+) | 1353 | spike glycoprotein | United Arab Emirates | Camelus dromedarius | |
| ASU90637.1 | Yusof,M.F., Queen,K., Eltahir,Y.M., Paden,C.R., Al Hammadi,Z.M.A.H., Tao,Y., Li,Y., Khalafalla,A.I., Shi,M., Zhang,J., Mohamed,M.S.A.E., Abd Elaal Ahmed,M.H., Azeez,I.A., Bensalah,O.K., Eldahab,Z.S., Al Hosani,F.I., Gerber,S.I., Hall,A.J., Tong,S., Al Muhairi,S.S., Al Hammadi,Z.M., Mohamed,M.S., Ahmed,M.H. | 2017-11-15T00:00:00Z | ssRNA(+) | 1353 | spike glycoprotein | United Arab Emirates | Camelus dromedarius | |
| ASU90648.1 | Yusof,M.F., Queen,K., Eltahir,Y.M., Paden,C.R., Al Hammadi,Z.M.A.H., Tao,Y., Li,Y., Khalafalla,A.I., Shi,M., Zhang,J., Mohamed,M.S.A.E., Abd Elaal Ahmed,M.H., Azeez,I.A., Bensalah,O.K., Eldahab,Z.S., Al Hosani,F.I., Gerber,S.I., Hall,A.J., Tong,S., Al Muhairi,S.S., Al Hammadi,Z.M., Mohamed,M.S., Ahmed,M.H. | 2017-11-15T00:00:00Z | ssRNA(+) | 1353 | spike glycoprotein | United Arab Emirates | Camelus dromedarius | |
| ASU90659.1 | Yusof,M.F., Queen,K., Eltahir,Y.M., Paden,C.R., Al Hammadi,Z.M.A.H., Tao,Y., Li,Y., Khalafalla,A.I., Shi,M., Zhang,J., Mohamed,M.S.A.E., Abd Elaal Ahmed,M.H., Azeez,I.A., Bensalah,O.K., Eldahab,Z.S., Al Hosani,F.I., Gerber,S.I., Hall,A.J., Tong,S., Al Muhairi,S.S., Al Hammadi,Z.M., Mohamed,M.S., Ahmed,M.H. | 2017-11-15T00:00:00Z | ssRNA(+) | 1353 | spike glycoprotein | United Arab Emirates | Camelus dromedarius | |
| ASU90670.1 | Yusof,M.F., Queen,K., Eltahir,Y.M., Paden,C.R., Al Hammadi,Z.M.A.H., Tao,Y., Li,Y., Khalafalla,A.I., Shi,M., Zhang,J., Mohamed,M.S.A.E., Abd Elaal Ahmed,M.H., Azeez,I.A., Bensalah,O.K., Eldahab,Z.S., Al Hosani,F.I., Gerber,S.I., Hall,A.J., Tong,S., Al Muhairi,S.S., Al Hammadi,Z.M., Mohamed,M.S., Ahmed,M.H. | 2017-11-15T00:00:00Z | ssRNA(+) | 1353 | spike glycoprotein | United Arab Emirates | Camelus dromedarius | |
| ASU90681.1 | Yusof,M.F., Queen,K., Eltahir,Y.M., Paden,C.R., Al Hammadi,Z.M.A.H., Tao,Y., Li,Y., Khalafalla,A.I., Shi,M., Zhang,J., Mohamed,M.S.A.E., Abd Elaal Ahmed,M.H., Azeez,I.A., Bensalah,O.K., Eldahab,Z.S., Al Hosani,F.I., Gerber,S.I., Hall,A.J., Tong,S., Al Muhairi,S.S., Al Hammadi,Z.M., Mohamed,M.S., Ahmed,M.H. | 2017-11-15T00:00:00Z | ssRNA(+) | 1353 | spike glycoprotein | United Arab Emirates | Camelus dromedarius | |
| ASU90692.1 | Yusof,M.F., Queen,K., Eltahir,Y.M., Paden,C.R., Al Hammadi,Z.M.A.H., Tao,Y., Li,Y., Khalafalla,A.I., Shi,M., Zhang,J., Mohamed,M.S.A.E., Abd Elaal Ahmed,M.H., Azeez,I.A., Bensalah,O.K., Eldahab,Z.S., Al Hosani,F.I., Gerber,S.I., Hall,A.J., Tong,S., Al Muhairi,S.S., Al Hammadi,Z.M., Mohamed,M.S., Ahmed,M.H. | 2017-11-15T00:00:00Z | ssRNA(+) | 1353 | spike glycoprotein | United Arab Emirates | Camelus dromedarius | |
| ASU90703.1 | Yusof,M.F., Queen,K., Eltahir,Y.M., Paden,C.R., Al Hammadi,Z.M.A.H., Tao,Y., Li,Y., Khalafalla,A.I., Shi,M., Zhang,J., Mohamed,M.S.A.E., Abd Elaal Ahmed,M.H., Azeez,I.A., Bensalah,O.K., Eldahab,Z.S., Al Hosani,F.I., Gerber,S.I., Hall,A.J., Tong,S., Al Muhairi,S.S., Al Hammadi,Z.M., Mohamed,M.S., Ahmed,M.H. | 2017-11-15T00:00:00Z | ssRNA(+) | 1353 | spike glycoprotein | United Arab Emirates | Camelus dromedarius | |
| ASU90714.1 | Yusof,M.F., Queen,K., Eltahir,Y.M., Paden,C.R., Al Hammadi,Z.M.A.H., Tao,Y., Li,Y., Khalafalla,A.I., Shi,M., Zhang,J., Mohamed,M.S.A.E., Abd Elaal Ahmed,M.H., Azeez,I.A., Bensalah,O.K., Eldahab,Z.S., Al Hosani,F.I., Gerber,S.I., Hall,A.J., Tong,S., Al Muhairi,S.S., Al Hammadi,Z.M., Mohamed,M.S., Ahmed,M.H. | 2017-11-15T00:00:00Z | ssRNA(+) | 1353 | spike glycoprotein | United Arab Emirates | Camelus dromedarius | |
| ASU90725.1 | Yusof,M.F., Queen,K., Eltahir,Y.M., Paden,C.R., Al Hammadi,Z.M.A.H., Tao,Y., Li,Y., Khalafalla,A.I., Shi,M., Zhang,J., Mohamed,M.S.A.E., Abd Elaal Ahmed,M.H., Azeez,I.A., Bensalah,O.K., Eldahab,Z.S., Al Hosani,F.I., Gerber,S.I., Hall,A.J., Tong,S., Al Muhairi,S.S., Al Hammadi,Z.M., Mohamed,M.S., Ahmed,M.H. | 2017-11-15T00:00:00Z | ssRNA(+) | 1353 | spike glycoprotein | United Arab Emirates | Camelus dromedarius | |
| ASU90736.1 | Yusof,M.F., Queen,K., Eltahir,Y.M., Paden,C.R., Al Hammadi,Z.M.A.H., Tao,Y., Li,Y., Khalafalla,A.I., Shi,M., Zhang,J., Mohamed,M.S.A.E., Abd Elaal Ahmed,M.H., Azeez,I.A., Bensalah,O.K., Eldahab,Z.S., Al Hosani,F.I., Gerber,S.I., Hall,A.J., Tong,S., Al Muhairi,S.S., Al Hammadi,Z.M., Mohamed,M.S., Ahmed,M.H. | 2017-11-15T00:00:00Z | ssRNA(+) | 1353 | spike glycoprotein | United Arab Emirates | Camelus dromedarius | |
| ASU90747.1 | Yusof,M.F., Queen,K., Eltahir,Y.M., Paden,C.R., Al Hammadi,Z.M.A.H., Tao,Y., Li,Y., Khalafalla,A.I., Shi,M., Zhang,J., Mohamed,M.S.A.E., Abd Elaal Ahmed,M.H., Azeez,I.A., Bensalah,O.K., Eldahab,Z.S., Al Hosani,F.I., Gerber,S.I., Hall,A.J., Tong,S., Al Muhairi,S.S., Al Hammadi,Z.M., Mohamed,M.S., Ahmed,M.H. | 2017-11-15T00:00:00Z | ssRNA(+) | 1353 | spike glycoprotein | United Arab Emirates | Camelus dromedarius | |
| ASU90758.1 | Yusof,M.F., Queen,K., Eltahir,Y.M., Paden,C.R., Al Hammadi,Z.M.A.H., Tao,Y., Li,Y., Khalafalla,A.I., Shi,M., Zhang,J., Mohamed,M.S.A.E., Abd Elaal Ahmed,M.H., Azeez,I.A., Bensalah,O.K., Eldahab,Z.S., Al Hosani,F.I., Gerber,S.I., Hall,A.J., Tong,S., Al Muhairi,S.S., Al Hammadi,Z.M., Mohamed,M.S., Ahmed,M.H. | 2017-11-15T00:00:00Z | ssRNA(+) | 1353 | spike glycoprotein | United Arab Emirates | Camelus dromedarius | |
| ASU90769.1 | Yusof,M.F., Queen,K., Eltahir,Y.M., Paden,C.R., Al Hammadi,Z.M.A.H., Tao,Y., Li,Y., Khalafalla,A.I., Shi,M., Zhang,J., Mohamed,M.S.A.E., Abd Elaal Ahmed,M.H., Azeez,I.A., Bensalah,O.K., Eldahab,Z.S., Al Hosani,F.I., Gerber,S.I., Hall,A.J., Tong,S., Al Muhairi,S.S., Al Hammadi,Z.M., Mohamed,M.S., Ahmed,M.H. | 2017-11-15T00:00:00Z | ssRNA(+) | 1353 | spike glycoprotein | United Arab Emirates | Camelus dromedarius | |
| ASU90780.1 | Yusof,M.F., Queen,K., Eltahir,Y.M., Paden,C.R., Al Hammadi,Z.M.A.H., Tao,Y., Li,Y., Khalafalla,A.I., Shi,M., Zhang,J., Mohamed,M.S.A.E., Abd Elaal Ahmed,M.H., Azeez,I.A., Bensalah,O.K., Eldahab,Z.S., Al Hosani,F.I., Gerber,S.I., Hall,A.J., Tong,S., Al Muhairi,S.S., Al Hammadi,Z.M., Mohamed,M.S., Ahmed,M.H. | 2017-11-15T00:00:00Z | ssRNA(+) | 1353 | spike glycoprotein | United Arab Emirates | Camelus dromedarius | |
| ASU90791.1 | Yusof,M.F., Queen,K., Eltahir,Y.M., Paden,C.R., Al Hammadi,Z.M.A.H., Tao,Y., Li,Y., Khalafalla,A.I., Shi,M., Zhang,J., Mohamed,M.S.A.E., Abd Elaal Ahmed,M.H., Azeez,I.A., Bensalah,O.K., Eldahab,Z.S., Al Hosani,F.I., Gerber,S.I., Hall,A.J., Tong,S., Al Muhairi,S.S., Al Hammadi,Z.M., Mohamed,M.S., Ahmed,M.H. | 2017-11-15T00:00:00Z | ssRNA(+) | 1353 | spike glycoprotein | United Arab Emirates | Camelus dromedarius | |
| ASU90802.1 | Yusof,M.F., Queen,K., Eltahir,Y.M., Paden,C.R., Al Hammadi,Z.M.A.H., Tao,Y., Li,Y., Khalafalla,A.I., Shi,M., Zhang,J., Mohamed,M.S.A.E., Abd Elaal Ahmed,M.H., Azeez,I.A., Bensalah,O.K., Eldahab,Z.S., Al Hosani,F.I., Gerber,S.I., Hall,A.J., Tong,S., Al Muhairi,S.S., Al Hammadi,Z.M., Mohamed,M.S., Ahmed,M.H. | 2017-11-15T00:00:00Z | ssRNA(+) | 1353 | spike glycoprotein | United Arab Emirates | Camelus dromedarius | |
| ASU90813.1 | Yusof,M.F., Queen,K., Eltahir,Y.M., Paden,C.R., Al Hammadi,Z.M.A.H., Tao,Y., Li,Y., Khalafalla,A.I., Shi,M., Zhang,J., Mohamed,M.S.A.E., Abd Elaal Ahmed,M.H., Azeez,I.A., Bensalah,O.K., Eldahab,Z.S., Al Hosani,F.I., Gerber,S.I., Hall,A.J., Tong,S., Al Muhairi,S.S., Al Hammadi,Z.M., Mohamed,M.S., Ahmed,M.H. | 2017-11-15T00:00:00Z | ssRNA(+) | 1353 | spike glycoprotein | United Arab Emirates | Camelus dromedarius | |
| ASU90824.1 | Yusof,M.F., Queen,K., Eltahir,Y.M., Paden,C.R., Al Hammadi,Z.M.A.H., Tao,Y., Li,Y., Khalafalla,A.I., Shi,M., Zhang,J., Mohamed,M.S.A.E., Abd Elaal Ahmed,M.H., Azeez,I.A., Bensalah,O.K., Eldahab,Z.S., Al Hosani,F.I., Gerber,S.I., Hall,A.J., Tong,S., Al Muhairi,S.S., Al Hammadi,Z.M., Mohamed,M.S., Ahmed,M.H. | 2017-11-15T00:00:00Z | ssRNA(+) | 1353 | spike glycoprotein | United Arab Emirates | Camelus dromedarius | |
| ASU90835.1 | Yusof,M.F., Queen,K., Eltahir,Y.M., Paden,C.R., Al Hammadi,Z.M.A.H., Tao,Y., Li,Y., Khalafalla,A.I., Shi,M., Zhang,J., Mohamed,M.S.A.E., Abd Elaal Ahmed,M.H., Azeez,I.A., Bensalah,O.K., Eldahab,Z.S., Al Hosani,F.I., Gerber,S.I., Hall,A.J., Tong,S., Al Muhairi,S.S., Al Hammadi,Z.M., Mohamed,M.S., Ahmed,M.H. | 2017-11-15T00:00:00Z | ssRNA(+) | 1353 | spike glycoprotein | United Arab Emirates | Camelus dromedarius | |
| ASU90846.1 | Yusof,M.F., Queen,K., Eltahir,Y.M., Paden,C.R., Al Hammadi,Z.M.A.H., Tao,Y., Li,Y., Khalafalla,A.I., Shi,M., Zhang,J., Mohamed,M.S.A.E., Abd Elaal Ahmed,M.H., Azeez,I.A., Bensalah,O.K., Eldahab,Z.S., Al Hosani,F.I., Gerber,S.I., Hall,A.J., Tong,S., Al Muhairi,S.S., Al Hammadi,Z.M., Mohamed,M.S., Ahmed,M.H. | 2017-11-15T00:00:00Z | ssRNA(+) | 1353 | spike glycoprotein | United Arab Emirates | Camelus dromedarius | |
| ASU90857.1 | Yusof,M.F., Queen,K., Eltahir,Y.M., Paden,C.R., Al Hammadi,Z.M.A.H., Tao,Y., Li,Y., Khalafalla,A.I., Shi,M., Zhang,J., Mohamed,M.S.A.E., Abd Elaal Ahmed,M.H., Azeez,I.A., Bensalah,O.K., Eldahab,Z.S., Al Hosani,F.I., Gerber,S.I., Hall,A.J., Tong,S., Al Muhairi,S.S., Al Hammadi,Z.M., Mohamed,M.S., Ahmed,M.H. | 2017-11-15T00:00:00Z | ssRNA(+) | 1353 | spike glycoprotein | United Arab Emirates | Camelus dromedarius | |
| ASU90868.1 | Yusof,M.F., Queen,K., Eltahir,Y.M., Paden,C.R., Al Hammadi,Z.M.A.H., Tao,Y., Li,Y., Khalafalla,A.I., Shi,M., Zhang,J., Mohamed,M.S.A.E., Abd Elaal Ahmed,M.H., Azeez,I.A., Bensalah,O.K., Eldahab,Z.S., Al Hosani,F.I., Gerber,S.I., Hall,A.J., Tong,S., Al Muhairi,S.S., Al Hammadi,Z.M., Mohamed,M.S., Ahmed,M.H. | 2017-11-15T00:00:00Z | ssRNA(+) | 1353 | spike glycoprotein | United Arab Emirates | Camelus dromedarius | |
| ASU90879.1 | Yusof,M.F., Queen,K., Eltahir,Y.M., Paden,C.R., Al Hammadi,Z.M.A.H., Tao,Y., Li,Y., Khalafalla,A.I., Shi,M., Zhang,J., Mohamed,M.S.A.E., Abd Elaal Ahmed,M.H., Azeez,I.A., Bensalah,O.K., Eldahab,Z.S., Al Hosani,F.I., Gerber,S.I., Hall,A.J., Tong,S., Al Muhairi,S.S., Al Hammadi,Z.M., Mohamed,M.S., Ahmed,M.H. | 2017-11-15T00:00:00Z | ssRNA(+) | 1353 | spike glycoprotein | United Arab Emirates | Camelus dromedarius | |
| ASU90890.1 | Yusof,M.F., Queen,K., Eltahir,Y.M., Paden,C.R., Al Hammadi,Z.M.A.H., Tao,Y., Li,Y., Khalafalla,A.I., Shi,M., Zhang,J., Mohamed,M.S.A.E., Abd Elaal Ahmed,M.H., Azeez,I.A., Bensalah,O.K., Eldahab,Z.S., Al Hosani,F.I., Gerber,S.I., Hall,A.J., Tong,S., Al Muhairi,S.S., Al Hammadi,Z.M., Mohamed,M.S., Ahmed,M.H. | 2017-11-15T00:00:00Z | ssRNA(+) | 1353 | spike glycoprotein | United Arab Emirates | Camelus dromedarius | |
| ASU90901.1 | Yusof,M.F., Queen,K., Eltahir,Y.M., Paden,C.R., Al Hammadi,Z.M.A.H., Tao,Y., Li,Y., Khalafalla,A.I., Shi,M., Zhang,J., Mohamed,M.S.A.E., Abd Elaal Ahmed,M.H., Azeez,I.A., Bensalah,O.K., Eldahab,Z.S., Al Hosani,F.I., Gerber,S.I., Hall,A.J., Tong,S., Al Muhairi,S.S., Al Hammadi,Z.M., Mohamed,M.S., Ahmed,M.H. | 2017-11-15T00:00:00Z | ssRNA(+) | 1353 | spike glycoprotein | United Arab Emirates | Camelus dromedarius | |
| ASU90912.1 | Yusof,M.F., Queen,K., Eltahir,Y.M., Paden,C.R., Al Hammadi,Z.M.A.H., Tao,Y., Li,Y., Khalafalla,A.I., Shi,M., Zhang,J., Mohamed,M.S.A.E., Abd Elaal Ahmed,M.H., Azeez,I.A., Bensalah,O.K., Eldahab,Z.S., Al Hosani,F.I., Gerber,S.I., Hall,A.J., Tong,S., Al Muhairi,S.S., Al Hammadi,Z.M., Mohamed,M.S., Ahmed,M.H. | 2017-11-15T00:00:00Z | ssRNA(+) | 1353 | spike glycoprotein | United Arab Emirates | Camelus dromedarius | |
| ASU90923.1 | Yusof,M.F., Queen,K., Eltahir,Y.M., Paden,C.R., Al Hammadi,Z.M.A.H., Tao,Y., Li,Y., Khalafalla,A.I., Shi,M., Zhang,J., Mohamed,M.S.A.E., Abd Elaal Ahmed,M.H., Azeez,I.A., Bensalah,O.K., Eldahab,Z.S., Al Hosani,F.I., Gerber,S.I., Hall,A.J., Tong,S., Al Muhairi,S.S., Al Hammadi,Z.M., Mohamed,M.S., Ahmed,M.H. | 2017-11-15T00:00:00Z | ssRNA(+) | 1353 | spike glycoprotein | United Arab Emirates | Camelus dromedarius | |
| ASU90934.1 | Yusof,M.F., Queen,K., Eltahir,Y.M., Paden,C.R., Al Hammadi,Z.M.A.H., Tao,Y., Li,Y., Khalafalla,A.I., Shi,M., Zhang,J., Mohamed,M.S.A.E., Abd Elaal Ahmed,M.H., Azeez,I.A., Bensalah,O.K., Eldahab,Z.S., Al Hosani,F.I., Gerber,S.I., Hall,A.J., Tong,S., Al Muhairi,S.S., Al Hammadi,Z.M., Mohamed,M.S., Ahmed,M.H. | 2017-11-15T00:00:00Z | ssRNA(+) | 1353 | spike glycoprotein | United Arab Emirates | Camelus dromedarius | |
| ASU90945.1 | Yusof,M.F., Queen,K., Eltahir,Y.M., Paden,C.R., Al Hammadi,Z.M.A.H., Tao,Y., Li,Y., Khalafalla,A.I., Shi,M., Zhang,J., Mohamed,M.S.A.E., Abd Elaal Ahmed,M.H., Azeez,I.A., Bensalah,O.K., Eldahab,Z.S., Al Hosani,F.I., Gerber,S.I., Hall,A.J., Tong,S., Al Muhairi,S.S., Al Hammadi,Z.M., Mohamed,M.S., Ahmed,M.H. | 2017-11-15T00:00:00Z | ssRNA(+) | 1353 | spike glycoprotein | United Arab Emirates | Camelus dromedarius | |
| ASU90956.1 | Yusof,M.F., Queen,K., Eltahir,Y.M., Paden,C.R., Al Hammadi,Z.M.A.H., Tao,Y., Li,Y., Khalafalla,A.I., Shi,M., Zhang,J., Mohamed,M.S.A.E., Abd Elaal Ahmed,M.H., Azeez,I.A., Bensalah,O.K., Eldahab,Z.S., Al Hosani,F.I., Gerber,S.I., Hall,A.J., Tong,S., Al Muhairi,S.S., Al Hammadi,Z.M., Mohamed,M.S., Ahmed,M.H. | 2017-11-15T00:00:00Z | ssRNA(+) | 1353 | spike glycoprotein | United Arab Emirates | Camelus dromedarius | |
| ASU90966.1 | Yusof,M.F., Queen,K., Eltahir,Y.M., Paden,C.R., Al Hammadi,Z.M.A.H., Tao,Y., Li,Y., Khalafalla,A.I., Shi,M., Zhang,J., Mohamed,M.S.A.E., Abd Elaal Ahmed,M.H., Azeez,I.A., Bensalah,O.K., Eldahab,Z.S., Al Hosani,F.I., Gerber,S.I., Hall,A.J., Tong,S., Al Muhairi,S.S., Al Hammadi,Z.M., Mohamed,M.S., Ahmed,M.H. | 2017-11-15T00:00:00Z | ssRNA(+) | 1353 | spike glycoprotein | United Arab Emirates | Camelus dromedarius | |
| ASU90978.1 | Yusof,M.F., Queen,K., Eltahir,Y.M., Paden,C.R., Al Hammadi,Z.M.A.H., Tao,Y., Li,Y., Khalafalla,A.I., Shi,M., Zhang,J., Mohamed,M.S.A.E., Abd Elaal Ahmed,M.H., Azeez,I.A., Bensalah,O.K., Eldahab,Z.S., Al Hosani,F.I., Gerber,S.I., Hall,A.J., Tong,S., Al Muhairi,S.S., Al Hammadi,Z.M., Mohamed,M.S., Ahmed,M.H. | 2017-11-15T00:00:00Z | ssRNA(+) | 1353 | spike glycoprotein | United Arab Emirates | Camelus dromedarius | |
| ASU90988.1 | Yusof,M.F., Queen,K., Eltahir,Y.M., Paden,C.R., Al Hammadi,Z.M.A.H., Tao,Y., Li,Y., Khalafalla,A.I., Shi,M., Zhang,J., Mohamed,M.S.A.E., Abd Elaal Ahmed,M.H., Azeez,I.A., Bensalah,O.K., Eldahab,Z.S., Al Hosani,F.I., Gerber,S.I., Hall,A.J., Tong,S., Al Muhairi,S.S., Al Hammadi,Z.M., Mohamed,M.S., Ahmed,M.H. | 2017-11-15T00:00:00Z | ssRNA(+) | 1353 | spike glycoprotein | United Arab Emirates | Camelus dromedarius | |
| ASU90999.1 | Yusof,M.F., Queen,K., Eltahir,Y.M., Paden,C.R., Al Hammadi,Z.M.A.H., Tao,Y., Li,Y., Khalafalla,A.I., Shi,M., Zhang,J., Mohamed,M.S.A.E., Abd Elaal Ahmed,M.H., Azeez,I.A., Bensalah,O.K., Eldahab,Z.S., Al Hosani,F.I., Gerber,S.I., Hall,A.J., Tong,S., Al Muhairi,S.S., Al Hammadi,Z.M., Mohamed,M.S., Ahmed,M.H. | 2017-11-15T00:00:00Z | ssRNA(+) | 1353 | spike glycoprotein | United Arab Emirates | Camelus dromedarius | |
| ASU91010.1 | Yusof,M.F., Queen,K., Eltahir,Y.M., Paden,C.R., Al Hammadi,Z.M.A.H., Tao,Y., Li,Y., Khalafalla,A.I., Shi,M., Zhang,J., Mohamed,M.S.A.E., Abd Elaal Ahmed,M.H., Azeez,I.A., Bensalah,O.K., Eldahab,Z.S., Al Hosani,F.I., Gerber,S.I., Hall,A.J., Tong,S., Al Muhairi,S.S., Al Hammadi,Z.M., Mohamed,M.S., Ahmed,M.H. | 2017-11-15T00:00:00Z | ssRNA(+) | 1353 | spike glycoprotein | United Arab Emirates | Camelus dromedarius | |
| ASU91021.1 | Yusof,M.F., Queen,K., Eltahir,Y.M., Paden,C.R., Al Hammadi,Z.M.A.H., Tao,Y., Li,Y., Khalafalla,A.I., Shi,M., Zhang,J., Mohamed,M.S.A.E., Abd Elaal Ahmed,M.H., Azeez,I.A., Bensalah,O.K., Eldahab,Z.S., Al Hosani,F.I., Gerber,S.I., Hall,A.J., Tong,S., Al Muhairi,S.S., Al Hammadi,Z.M., Mohamed,M.S., Ahmed,M.H. | 2017-11-15T00:00:00Z | ssRNA(+) | 1353 | spike glycoprotein | United Arab Emirates | Camelus dromedarius | |
| ASU91032.1 | Yusof,M.F., Queen,K., Eltahir,Y.M., Paden,C.R., Al Hammadi,Z.M.A.H., Tao,Y., Li,Y., Khalafalla,A.I., Shi,M., Zhang,J., Mohamed,M.S.A.E., Abd Elaal Ahmed,M.H., Azeez,I.A., Bensalah,O.K., Eldahab,Z.S., Al Hosani,F.I., Gerber,S.I., Hall,A.J., Tong,S., Al Muhairi,S.S., Al Hammadi,Z.M., Mohamed,M.S., Ahmed,M.H. | 2017-11-15T00:00:00Z | ssRNA(+) | 1353 | spike glycoprotein | United Arab Emirates | Camelus dromedarius | |
| ASU91043.1 | Yusof,M.F., Queen,K., Eltahir,Y.M., Paden,C.R., Al Hammadi,Z.M.A.H., Tao,Y., Li,Y., Khalafalla,A.I., Shi,M., Zhang,J., Mohamed,M.S.A.E., Abd Elaal Ahmed,M.H., Azeez,I.A., Bensalah,O.K., Eldahab,Z.S., Al Hosani,F.I., Gerber,S.I., Hall,A.J., Tong,S., Al Muhairi,S.S., Al Hammadi,Z.M., Mohamed,M.S., Ahmed,M.H. | 2017-11-15T00:00:00Z | ssRNA(+) | 1353 | spike glycoprotein | United Arab Emirates | Camelus dromedarius | |
| ASU91054.1 | Yusof,M.F., Queen,K., Eltahir,Y.M., Paden,C.R., Al Hammadi,Z.M.A.H., Tao,Y., Li,Y., Khalafalla,A.I., Shi,M., Zhang,J., Mohamed,M.S.A.E., Abd Elaal Ahmed,M.H., Azeez,I.A., Bensalah,O.K., Eldahab,Z.S., Al Hosani,F.I., Gerber,S.I., Hall,A.J., Tong,S., Al Muhairi,S.S., Al Hammadi,Z.M., Mohamed,M.S., Ahmed,M.H. | 2017-11-15T00:00:00Z | ssRNA(+) | 1353 | spike glycoprotein | United Arab Emirates | Camelus dromedarius | |
| ASU91065.1 | Yusof,M.F., Queen,K., Eltahir,Y.M., Paden,C.R., Al Hammadi,Z.M.A.H., Tao,Y., Li,Y., Khalafalla,A.I., Shi,M., Zhang,J., Mohamed,M.S.A.E., Abd Elaal Ahmed,M.H., Azeez,I.A., Bensalah,O.K., Eldahab,Z.S., Al Hosani,F.I., Gerber,S.I., Hall,A.J., Tong,S., Al Muhairi,S.S., Al Hammadi,Z.M., Mohamed,M.S., Ahmed,M.H. | 2017-11-15T00:00:00Z | ssRNA(+) | 1353 | spike glycoprotein | United Arab Emirates | Camelus dromedarius | |
| ASU91076.1 | Yusof,M.F., Queen,K., Eltahir,Y.M., Paden,C.R., Al Hammadi,Z.M.A.H., Tao,Y., Li,Y., Khalafalla,A.I., Shi,M., Zhang,J., Mohamed,M.S.A.E., Abd Elaal Ahmed,M.H., Azeez,I.A., Bensalah,O.K., Eldahab,Z.S., Al Hosani,F.I., Gerber,S.I., Hall,A.J., Tong,S., Al Muhairi,S.S., Al Hammadi,Z.M., Mohamed,M.S., Ahmed,M.H. | 2017-11-15T00:00:00Z | ssRNA(+) | 1353 | spike glycoprotein | United Arab Emirates | Camelus dromedarius | |
| ASU91087.1 | Yusof,M.F., Queen,K., Eltahir,Y.M., Paden,C.R., Al Hammadi,Z.M.A.H., Tao,Y., Li,Y., Khalafalla,A.I., Shi,M., Zhang,J., Mohamed,M.S.A.E., Abd Elaal Ahmed,M.H., Azeez,I.A., Bensalah,O.K., Eldahab,Z.S., Al Hosani,F.I., Gerber,S.I., Hall,A.J., Tong,S., Al Muhairi,S.S., Al Hammadi,Z.M., Mohamed,M.S., Ahmed,M.H. | 2017-11-15T00:00:00Z | ssRNA(+) | 1353 | spike glycoprotein | United Arab Emirates | Camelus dromedarius | |
| ASU91098.1 | Yusof,M.F., Queen,K., Eltahir,Y.M., Paden,C.R., Al Hammadi,Z.M.A.H., Tao,Y., Li,Y., Khalafalla,A.I., Shi,M., Zhang,J., Mohamed,M.S.A.E., Abd Elaal Ahmed,M.H., Azeez,I.A., Bensalah,O.K., Eldahab,Z.S., Al Hosani,F.I., Gerber,S.I., Hall,A.J., Tong,S., Al Muhairi,S.S., Al Hammadi,Z.M., Mohamed,M.S., Ahmed,M.H. | 2017-11-15T00:00:00Z | ssRNA(+) | 1353 | spike glycoprotein | United Arab Emirates | Camelus dromedarius | |
| ASU91109.1 | Yusof,M.F., Queen,K., Eltahir,Y.M., Paden,C.R., Al Hammadi,Z.M.A.H., Tao,Y., Li,Y., Khalafalla,A.I., Shi,M., Zhang,J., Mohamed,M.S.A.E., Abd Elaal Ahmed,M.H., Azeez,I.A., Bensalah,O.K., Eldahab,Z.S., Al Hosani,F.I., Gerber,S.I., Hall,A.J., Tong,S., Al Muhairi,S.S., Al Hammadi,Z.M., Mohamed,M.S., Ahmed,M.H. | 2017-11-15T00:00:00Z | ssRNA(+) | 1353 | spike glycoprotein | United Arab Emirates | Camelus dromedarius | |
| ASU91120.1 | Yusof,M.F., Queen,K., Eltahir,Y.M., Paden,C.R., Al Hammadi,Z.M.A.H., Tao,Y., Li,Y., Khalafalla,A.I., Shi,M., Zhang,J., Mohamed,M.S.A.E., Abd Elaal Ahmed,M.H., Azeez,I.A., Bensalah,O.K., Eldahab,Z.S., Al Hosani,F.I., Gerber,S.I., Hall,A.J., Tong,S., Al Muhairi,S.S., Al Hammadi,Z.M., Mohamed,M.S., Ahmed,M.H. | 2017-11-15T00:00:00Z | ssRNA(+) | 1353 | spike glycoprotein | United Arab Emirates | Camelus dromedarius | |
| ASU91131.1 | Yusof,M.F., Queen,K., Eltahir,Y.M., Paden,C.R., Al Hammadi,Z.M.A.H., Tao,Y., Li,Y., Khalafalla,A.I., Shi,M., Zhang,J., Mohamed,M.S.A.E., Abd Elaal Ahmed,M.H., Azeez,I.A., Bensalah,O.K., Eldahab,Z.S., Al Hosani,F.I., Gerber,S.I., Hall,A.J., Tong,S., Al Muhairi,S.S., Al Hammadi,Z.M., Mohamed,M.S., Ahmed,M.H. | 2017-11-15T00:00:00Z | ssRNA(+) | 1353 | spike glycoprotein | United Arab Emirates | Camelus dromedarius | |
| ASU91142.1 | Yusof,M.F., Queen,K., Eltahir,Y.M., Paden,C.R., Al Hammadi,Z.M.A.H., Tao,Y., Li,Y., Khalafalla,A.I., Shi,M., Zhang,J., Mohamed,M.S.A.E., Abd Elaal Ahmed,M.H., Azeez,I.A., Bensalah,O.K., Eldahab,Z.S., Al Hosani,F.I., Gerber,S.I., Hall,A.J., Tong,S., Al Muhairi,S.S., Al Hammadi,Z.M., Mohamed,M.S., Ahmed,M.H. | 2017-11-15T00:00:00Z | ssRNA(+) | 1353 | spike glycoprotein | United Arab Emirates | Camelus dromedarius | |
| ASU91153.1 | Yusof,M.F., Queen,K., Eltahir,Y.M., Paden,C.R., Al Hammadi,Z.M.A.H., Tao,Y., Li,Y., Khalafalla,A.I., Shi,M., Zhang,J., Mohamed,M.S.A.E., Abd Elaal Ahmed,M.H., Azeez,I.A., Bensalah,O.K., Eldahab,Z.S., Al Hosani,F.I., Gerber,S.I., Hall,A.J., Tong,S., Al Muhairi,S.S., Al Hammadi,Z.M., Mohamed,M.S., Ahmed,M.H. | 2017-11-15T00:00:00Z | ssRNA(+) | 1353 | spike glycoprotein | United Arab Emirates | Camelus dromedarius | |
| ASU91164.1 | Yusof,M.F., Queen,K., Eltahir,Y.M., Paden,C.R., Al Hammadi,Z.M.A.H., Tao,Y., Li,Y., Khalafalla,A.I., Shi,M., Zhang,J., Mohamed,M.S.A.E., Abd Elaal Ahmed,M.H., Azeez,I.A., Bensalah,O.K., Eldahab,Z.S., Al Hosani,F.I., Gerber,S.I., Hall,A.J., Tong,S., Al Muhairi,S.S., Al Hammadi,Z.M., Mohamed,M.S., Ahmed,M.H. | 2017-11-15T00:00:00Z | ssRNA(+) | 1353 | spike glycoprotein | United Arab Emirates | Camelus dromedarius | |
| ASU91175.1 | Yusof,M.F., Queen,K., Eltahir,Y.M., Paden,C.R., Al Hammadi,Z.M.A.H., Tao,Y., Li,Y., Khalafalla,A.I., Shi,M., Zhang,J., Mohamed,M.S.A.E., Abd Elaal Ahmed,M.H., Azeez,I.A., Bensalah,O.K., Eldahab,Z.S., Al Hosani,F.I., Gerber,S.I., Hall,A.J., Tong,S., Al Muhairi,S.S., Al Hammadi,Z.M., Mohamed,M.S., Ahmed,M.H. | 2017-11-15T00:00:00Z | ssRNA(+) | 1353 | spike glycoprotein | United Arab Emirates | Camelus dromedarius | |
| ASU91186.1 | Yusof,M.F., Queen,K., Eltahir,Y.M., Paden,C.R., Al Hammadi,Z.M.A.H., Tao,Y., Li,Y., Khalafalla,A.I., Shi,M., Zhang,J., Mohamed,M.S.A.E., Abd Elaal Ahmed,M.H., Azeez,I.A., Bensalah,O.K., Eldahab,Z.S., Al Hosani,F.I., Gerber,S.I., Hall,A.J., Tong,S., Al Muhairi,S.S., Al Hammadi,Z.M., Mohamed,M.S., Ahmed,M.H. | 2017-11-15T00:00:00Z | ssRNA(+) | 1353 | spike glycoprotein | United Arab Emirates | Camelus dromedarius | |
| ASU91197.1 | Yusof,M.F., Queen,K., Eltahir,Y.M., Paden,C.R., Al Hammadi,Z.M.A.H., Tao,Y., Li,Y., Khalafalla,A.I., Shi,M., Zhang,J., Mohamed,M.S.A.E., Abd Elaal Ahmed,M.H., Azeez,I.A., Bensalah,O.K., Eldahab,Z.S., Al Hosani,F.I., Gerber,S.I., Hall,A.J., Tong,S., Al Muhairi,S.S., Al Hammadi,Z.M., Mohamed,M.S., Ahmed,M.H. | 2017-11-15T00:00:00Z | ssRNA(+) | 1353 | spike glycoprotein | United Arab Emirates | Camelus dromedarius | |
| ASU91208.1 | Yusof,M.F., Queen,K., Eltahir,Y.M., Paden,C.R., Al Hammadi,Z.M.A.H., Tao,Y., Li,Y., Khalafalla,A.I., Shi,M., Zhang,J., Mohamed,M.S.A.E., Abd Elaal Ahmed,M.H., Azeez,I.A., Bensalah,O.K., Eldahab,Z.S., Al Hosani,F.I., Gerber,S.I., Hall,A.J., Tong,S., Al Muhairi,S.S., Al Hammadi,Z.M., Mohamed,M.S., Ahmed,M.H. | 2017-11-15T00:00:00Z | ssRNA(+) | 1353 | spike glycoprotein | United Arab Emirates | Camelus dromedarius | |
| ASU91219.1 | Yusof,M.F., Queen,K., Eltahir,Y.M., Paden,C.R., Al Hammadi,Z.M.A.H., Tao,Y., Li,Y., Khalafalla,A.I., Shi,M., Zhang,J., Mohamed,M.S.A.E., Abd Elaal Ahmed,M.H., Azeez,I.A., Bensalah,O.K., Eldahab,Z.S., Al Hosani,F.I., Gerber,S.I., Hall,A.J., Tong,S., Al Muhairi,S.S., Al Hammadi,Z.M., Mohamed,M.S., Ahmed,M.H. | 2017-11-15T00:00:00Z | ssRNA(+) | 1353 | spike glycoprotein | United Arab Emirates | Camelus dromedarius | |
| ASU91230.1 | Yusof,M.F., Queen,K., Eltahir,Y.M., Paden,C.R., Al Hammadi,Z.M.A.H., Tao,Y., Li,Y., Khalafalla,A.I., Shi,M., Zhang,J., Mohamed,M.S.A.E., Abd Elaal Ahmed,M.H., Azeez,I.A., Bensalah,O.K., Eldahab,Z.S., Al Hosani,F.I., Gerber,S.I., Hall,A.J., Tong,S., Al Muhairi,S.S., Al Hammadi,Z.M., Mohamed,M.S., Ahmed,M.H. | 2017-11-15T00:00:00Z | ssRNA(+) | 1353 | spike glycoprotein | United Arab Emirates | Camelus dromedarius | |
| ASU91241.1 | Yusof,M.F., Queen,K., Eltahir,Y.M., Paden,C.R., Al Hammadi,Z.M.A.H., Tao,Y., Li,Y., Khalafalla,A.I., Shi,M., Zhang,J., Mohamed,M.S.A.E., Abd Elaal Ahmed,M.H., Azeez,I.A., Bensalah,O.K., Eldahab,Z.S., Al Hosani,F.I., Gerber,S.I., Hall,A.J., Tong,S., Al Muhairi,S.S., Al Hammadi,Z.M., Mohamed,M.S., Ahmed,M.H. | 2017-11-15T00:00:00Z | ssRNA(+) | 1353 | spike glycoprotein | United Arab Emirates | Camelus dromedarius | |
| ASU91252.1 | Yusof,M.F., Queen,K., Eltahir,Y.M., Paden,C.R., Al Hammadi,Z.M.A.H., Tao,Y., Li,Y., Khalafalla,A.I., Shi,M., Zhang,J., Mohamed,M.S.A.E., Abd Elaal Ahmed,M.H., Azeez,I.A., Bensalah,O.K., Eldahab,Z.S., Al Hosani,F.I., Gerber,S.I., Hall,A.J., Tong,S., Al Muhairi,S.S., Al Hammadi,Z.M., Mohamed,M.S., Ahmed,M.H. | 2017-11-15T00:00:00Z | ssRNA(+) | 1353 | spike glycoprotein | United Arab Emirates | Camelus dromedarius | |
| ASU91262.1 | Yusof,M.F., Queen,K., Eltahir,Y.M., Paden,C.R., Al Hammadi,Z.M.A.H., Tao,Y., Li,Y., Khalafalla,A.I., Shi,M., Zhang,J., Mohamed,M.S.A.E., Abd Elaal Ahmed,M.H., Azeez,I.A., Bensalah,O.K., Eldahab,Z.S., Al Hosani,F.I., Gerber,S.I., Hall,A.J., Tong,S., Al Muhairi,S.S., Al Hammadi,Z.M., Mohamed,M.S., Ahmed,M.H. | 2017-11-15T00:00:00Z | ssRNA(+) | 1353 | spike glycoprotein | United Arab Emirates | Camelus dromedarius | |
| ASU91273.1 | Yusof,M.F., Queen,K., Eltahir,Y.M., Paden,C.R., Al Hammadi,Z.M.A.H., Tao,Y., Li,Y., Khalafalla,A.I., Shi,M., Zhang,J., Mohamed,M.S.A.E., Abd Elaal Ahmed,M.H., Azeez,I.A., Bensalah,O.K., Eldahab,Z.S., Al Hosani,F.I., Gerber,S.I., Hall,A.J., Tong,S., Al Muhairi,S.S., Al Hammadi,Z.M., Mohamed,M.S., Ahmed,M.H. | 2017-11-15T00:00:00Z | ssRNA(+) | 1353 | spike glycoprotein | United Arab Emirates | Camelus dromedarius | |
| ASU91284.1 | Yusof,M.F., Queen,K., Eltahir,Y.M., Paden,C.R., Al Hammadi,Z.M.A.H., Tao,Y., Li,Y., Khalafalla,A.I., Shi,M., Zhang,J., Mohamed,M.S.A.E., Abd Elaal Ahmed,M.H., Azeez,I.A., Bensalah,O.K., Eldahab,Z.S., Al Hosani,F.I., Gerber,S.I., Hall,A.J., Tong,S., Al Muhairi,S.S., Al Hammadi,Z.M., Mohamed,M.S., Ahmed,M.H. | 2017-11-15T00:00:00Z | ssRNA(+) | 1353 | spike glycoprotein | United Arab Emirates | Camelus dromedarius | |
| ASU91295.1 | Yusof,M.F., Queen,K., Eltahir,Y.M., Paden,C.R., Al Hammadi,Z.M.A.H., Tao,Y., Li,Y., Khalafalla,A.I., Shi,M., Zhang,J., Mohamed,M.S.A.E., Abd Elaal Ahmed,M.H., Azeez,I.A., Bensalah,O.K., Eldahab,Z.S., Al Hosani,F.I., Gerber,S.I., Hall,A.J., Tong,S., Al Muhairi,S.S., Al Hammadi,Z.M., Mohamed,M.S., Ahmed,M.H. | 2017-11-15T00:00:00Z | ssRNA(+) | 1353 | spike glycoprotein | United Arab Emirates | Camelus dromedarius | |
| ASU91305.1 | Yusof,M.F., Queen,K., Eltahir,Y.M., Paden,C.R., Al Hammadi,Z.M.A.H., Tao,Y., Li,Y., Khalafalla,A.I., Shi,M., Zhang,J., Mohamed,M.S.A.E., Abd Elaal Ahmed,M.H., Azeez,I.A., Bensalah,O.K., Eldahab,Z.S., Al Hosani,F.I., Gerber,S.I., Hall,A.J., Tong,S., Al Muhairi,S.S., Al Hammadi,Z.M., Mohamed,M.S., Ahmed,M.H. | 2017-11-15T00:00:00Z | ssRNA(+) | 1353 | spike glycoprotein | United Arab Emirates | Camelus dromedarius | |
| ASU91315.1 | Yusof,M.F., Queen,K., Eltahir,Y.M., Paden,C.R., Al Hammadi,Z.M.A.H., Tao,Y., Li,Y., Khalafalla,A.I., Shi,M., Zhang,J., Mohamed,M.S.A.E., Abd Elaal Ahmed,M.H., Azeez,I.A., Bensalah,O.K., Eldahab,Z.S., Al Hosani,F.I., Gerber,S.I., Hall,A.J., Tong,S., Al Muhairi,S.S., Al Hammadi,Z.M., Mohamed,M.S., Ahmed,M.H. | 2017-11-15T00:00:00Z | ssRNA(+) | 1353 | spike glycoprotein | United Arab Emirates | Camelus dromedarius | |
| ASU91325.1 | Yusof,M.F., Queen,K., Eltahir,Y.M., Paden,C.R., Al Hammadi,Z.M.A.H., Tao,Y., Li,Y., Khalafalla,A.I., Shi,M., Zhang,J., Mohamed,M.S.A.E., Abd Elaal Ahmed,M.H., Azeez,I.A., Bensalah,O.K., Eldahab,Z.S., Al Hosani,F.I., Gerber,S.I., Hall,A.J., Tong,S., Al Muhairi,S.S., Al Hammadi,Z.M., Mohamed,M.S., Ahmed,M.H. | 2017-11-15T00:00:00Z | ssRNA(+) | 1353 | spike glycoprotein | United Arab Emirates | Camelus dromedarius | |
| AOR17479.1 | Ali,M., Kandeil,A., Gomaa,M., El Shesheny,R., Nageh,A., Shehata,M., Kayali,G. | 2016-12-31T00:00:00Z | ssRNA(+) | 195 | spike glycoprotein | Egypt | Camelus dromedarius | |
| AOR17480.1 | Ali,M., Kandeil,A., Gomaa,M., El Shesheny,R., Nageh,A., Shehata,M., Kayali,G. | 2016-12-31T00:00:00Z | ssRNA(+) | 195 | spike glycoprotein | Egypt | Camelus dromedarius | |
| AOR17481.1 | Ali,M., Kandeil,A., Gomaa,M., El Shesheny,R., Nageh,A., Shehata,M., Kayali,G. | 2016-12-31T00:00:00Z | ssRNA(+) | 195 | spike glycoprotein | Egypt | Camelus dromedarius | |
| AOR17482.1 | Ali,M., Kandeil,A., Gomaa,M., El Shesheny,R., Nageh,A., Shehata,M., Kayali,G. | 2016-12-31T00:00:00Z | ssRNA(+) | 195 | spike glycoprotein | Egypt | Camelus dromedarius | |
| AOR17483.1 | Ali,M., Kandeil,A., Gomaa,M., El Shesheny,R., Nageh,A., Shehata,M., Kayali,G. | 2016-12-31T00:00:00Z | ssRNA(+) | 195 | spike glycoprotein | Egypt | Camelus dromedarius | |
| AOR17484.1 | Ali,M., Kandeil,A., Gomaa,M., El Shesheny,R., Nageh,A., Shehata,M., Kayali,G. | 2016-12-31T00:00:00Z | ssRNA(+) | 195 | spike glycoprotein | Egypt | Camelus dromedarius | |
| AOR17485.1 | Ali,M., Kandeil,A., Gomaa,M., El Shesheny,R., Nageh,A., Shehata,M., Kayali,G. | 2016-12-31T00:00:00Z | ssRNA(+) | 147 | spike glycoprotein | Egypt | Camelus dromedarius | |
| AOR17486.1 | Ali,M., Kandeil,A., Gomaa,M., El Shesheny,R., Nageh,A., Shehata,M., Kayali,G. | 2016-12-31T00:00:00Z | ssRNA(+) | 144 | spike glycoprotein | Egypt | Camelus dromedarius | |
| AMO03401.1 | Shehata,M.M., Kandeil,A., Ali,M.A., Kayali,G. | 2016-03-07T00:00:00Z | ssRNA(+) | 1353 | spike glycoprotein | Egypt | Camelus dromedarius | |
| AHY61337.1 | Yang,L., Wu,Z., Ren,X., Yang,F., Zhang,J., He,G., Dong,J., Sun,L., Zhu,Y., Zhang,S., Jin,Q., Yang,J., Qian,Z., Du,J. | 2014-05-06T00:00:00Z | ssRNA(+) | 1322 | spike glycoprotein | China | Vespertilio sinensis | |
| AYN72346.1 | Zohaib,A., Saqib,M., Athar,M.A., Chen,J., Sial,A.U., Khan,S., Taj,Z., Sadia,H., Tahir,U., Tayyab,M.H., Qureshi,M.A., Mansoor,M.K., Naeem,M.A., Hu,B.J., Khan,B.A., Ujjan,I.D., Li,B., Zhang,W., Luo,Y., Zhu,Y., Waruhiu,C., Khan,I., Yang,X.L., Sajid,M.S., Corman,V.M., Yan,B., Shi,Z.L. | 2018-10-29T00:00:00Z | ssRNA(+) | 320 | spike glycoproteinein | Pakistan | Camelus | |
| USL83011.1 | Korneenko,E.V., Speranskaya,A.S., Samoilov,A.E., Artyushin,I.V., Dedkov,V.G., Dolgova,A.S. | 2022-06-27T00:00:00Z | ssRNA(+) | 1366 | spike protein | Russia | Pipistrellus nathusii | |
| QSI72189.1 | Lee,H.H., Teng,L.L., Woo,C.Y. | 2021-03-08T00:00:00Z | ssRNA(+) | 1353 | spike protein | United Arab Emirates | Camelus dromedarius | |
| QSI72200.1 | Lee,H.H., Teng,L.L., Woo,C.Y. | 2021-03-08T00:00:00Z | ssRNA(+) | 1353 | spike protein | United Arab Emirates | Camelus dromedarius | |
| QKF93418.1 | Kim,M., Cho,H., Lee,S.-H., Park,W.-J., Kim,J.-M., Moon,J.-S., Kim,G.-W., Lee,W., Jung,H.-G., Yang,J.-S., Choi,J.-H., Lee,J.-Y., Kim,S.S., Oh,J.-W. | 2021-01-11T00:00:00Z | ssRNA(+) | 1353 | spike protein | |  |  |
| QKX95939.1 | Gutierrez-Alvarez,J., Wang,L., Fernandez-Delgado,R., Li,K., McCray,P.B., Perlman,S., Sola,I., Zuniga,S., Enjuanes,L. | 2020-06-29T00:00:00Z | ssRNA(+) | 1353 | spike protein | |  |  |
| QCZ25046.1 | Alhafufi,A.N., Samy,K., Albaqshi,H.A., Al-Ghadeer,H., Zidan,K.H., Alyousef,A. | 2020-06-01T00:00:00Z | ssRNA(+) | 1353 | spike protein | Saudi Arabia | Camelus bactrianus | |
| QGV13484.1 | Shaheen,M.A., Hagag,N.M., Hanafy,S., Ibrahim,E., Mansour,A., Nubie,O., Lubroth,J., Von Dobschuetz,S., Makonnen,Y. | 2019-12-15T00:00:00Z | ssRNA(+) | 1353 | spike protein | Egypt | Camelus | |
| QEU56411.1 | Sohrab,S.S., Azhar,E.I. | 2019-10-01T00:00:00Z | ssRNA(+) | 1353 | spike protein | Saudi Arabia | Camelus dromedarius | |
| QCI31469.1 | Mishra,N., Alagaili,A.N., Mohammed,O.B., Briese,T., Lipkin,W.I. | 2019-05-07T00:00:00Z | ssRNA(+) | 1353 | spike protein | Saudi Arabia | Camelus dromedarius | |
| QCI31480.1 | Mishra,N., Alagaili,A.N., Mohammed,O.B., Briese,T., Lipkin,W.I. | 2019-05-07T00:00:00Z | ssRNA(+) | 1353 | spike protein | Saudi Arabia | Camelus dromedarius | |
| QAT98898.1 | Kiambi,S., Corman,V.M., Sitawa,R., Githinji,J., Ngoci,J., Ozomata,A.S., Gardner,E., von Dobschuetz,S., Morzaria,S., Kimutai,J., Schroeder,S., Njagi,O., Simpkin,P., Rugalema,G., Tadesse,Z., Lubroth,J., Makonnen,Y., Drosten,C., Muller,M.A., Fasina,F.O. | 2019-01-30T00:00:00Z | ssRNA(+) | 1353 | spike protein | Kenya | Camelus dromedarius | |
| QAT98909.1 | Kiambi,S., Corman,V.M., Sitawa,R., Githinji,J., Ngoci,J., Ozomata,A.S., Gardner,E., von Dobschuetz,S., Morzaria,S., Kimutai,J., Schroeder,S., Njagi,O., Simpkin,P., Rugalema,G., Tadesse,Z., Lubroth,J., Makonnen,Y., Drosten,C., Muller,M.A., Fasina,F.O. | 2019-01-30T00:00:00Z | ssRNA(+) | 1353 | spike protein | Kenya | Camelus dromedarius | |
| ATQ39390.1 | Geldenhuys,M., Mortlock,M., Weyer,J., Bezuidt,O., Seamark,E.C.J., Kearney,T., Gleasner,C., Erkkila,T.H., Cui,H., Markotter,W., Seamark,E., Tracy,E. | 2018-04-04T00:00:00Z | ssRNA(+) | 1344 | spike protein | South Africa | Laephotis capensis | |
| AUM60014.1 | Moreno,A., Lelli,D., de Sabato,L., Zaccaria,G., Boni,A., Sozzi,E., Prosperi,A., Lavazza,A., Cella,E., Castrucci,M.R., Cicozzi,M., Vaccari,G., De Sabato,L. | 2018-01-10T00:00:00Z | ssRNA(+) | 1345 | spike protein | Italy | Hypsugo savii | |
| AUM60024.1 | Moreno,A., Lelli,D., de Sabato,L., Zaccaria,G., Boni,A., Sozzi,E., Prosperi,A., Lavazza,A., Cella,E., Castrucci,M.R., Cicozzi,M., Vaccari,G., De Sabato,L. | 2018-01-10T00:00:00Z | ssRNA(+) | 1345 | spike protein | Italy | Pipistrellus kuhlii | |
| AQZ41296.1 | Queen,K., Al-Jardani,A., Zhang,J., Al Kindi,H., Li,Y., Tao,Y., Al Baqlani,S., Al Mahrouqi,S., Tong,S. | 2017-12-31T00:00:00Z | ssRNA(+) | 1353 | spike protein | Oman | Camelus | |
| ATW75477.1 | Yusof,M.F., Queen,K., Eltahir,Y.M., Paden,C.R., Al Hammadi,Z.M.A.H., Tao,Y., Li,Y., Khalafalla,A.I., Shi,M., Zhang,J., Mohamed,M.S.A.E., Abd Elaal Ahmed,M.H., Azeez,I.A., Bensalah,O.K., Eldahab,Z.S., Al Hosani,F.I., Gerber,S.I., Hall,A.J., Tong,S., Al Muhairi,S.S., Al Hammadi,Z.M., Mohamed,M.S., Ahmed,M.H. | 2017-11-25T00:00:00Z | ssRNA(+) | 1352 | spike protein | United Arab Emirates | Camelus dromedarius | |
| ATW75478.1 | Yusof,M.F., Queen,K., Eltahir,Y.M., Paden,C.R., Al Hammadi,Z.M.A.H., Tao,Y., Li,Y., Khalafalla,A.I., Shi,M., Zhang,J., Mohamed,M.S.A.E., Abd Elaal Ahmed,M.H., Azeez,I.A., Bensalah,O.K., Eldahab,Z.S., Al Hosani,F.I., Gerber,S.I., Hall,A.J., Tong,S., Al Muhairi,S.S., Al Hammadi,Z.M., Mohamed,M.S., Ahmed,M.H. | 2017-11-25T00:00:00Z | ssRNA(+) | 1353 | spike protein | United Arab Emirates | Camelus dromedarius | |
| ATW75479.1 | Yusof,M.F., Queen,K., Eltahir,Y.M., Paden,C.R., Al Hammadi,Z.M.A.H., Tao,Y., Li,Y., Khalafalla,A.I., Shi,M., Zhang,J., Mohamed,M.S.A.E., Abd Elaal Ahmed,M.H., Azeez,I.A., Bensalah,O.K., Eldahab,Z.S., Al Hosani,F.I., Gerber,S.I., Hall,A.J., Tong,S., Al Muhairi,S.S., Al Hammadi,Z.M., Mohamed,M.S., Ahmed,M.H. | 2017-11-25T00:00:00Z | ssRNA(+) | 1353 | spike protein | United Arab Emirates | Camelus dromedarius | |
| ATW75480.1 | Yusof,M.F., Queen,K., Eltahir,Y.M., Paden,C.R., Al Hammadi,Z.M.A.H., Tao,Y., Li,Y., Khalafalla,A.I., Shi,M., Zhang,J., Mohamed,M.S.A.E., Abd Elaal Ahmed,M.H., Azeez,I.A., Bensalah,O.K., Eldahab,Z.S., Al Hosani,F.I., Gerber,S.I., Hall,A.J., Tong,S., Al Muhairi,S.S., Al Hammadi,Z.M., Mohamed,M.S., Ahmed,M.H. | 2017-11-25T00:00:00Z | ssRNA(+) | 1353 | spike protein | United Arab Emirates | Camelus dromedarius | |
| ARA67737.1 | Ali,M.A., ElShesheny,R., Kandeil,A., Shehata,M. | 2017-03-26T00:00:00Z | ssRNA(+) | 178 | spike protein | Egypt | Camelus | |
| ARA67738.1 | Ali,M.A., ElShesheny,R., Kandeil,A., Shehata,M. | 2017-03-26T00:00:00Z | ssRNA(+) | 200 | spike protein | Egypt | Camelus | |
| ARA67739.1 | Ali,M.A., ElShesheny,R., Kandeil,A., Shehata,M. | 2017-03-26T00:00:00Z | ssRNA(+) | 129 | spike protein | Egypt | Camelus | |
| ARA67740.1 | Ali,M.A., ElShesheny,R., Kandeil,A., Shehata,M. | 2017-03-26T00:00:00Z | ssRNA(+) | 111 | spike protein | Egypt | Camelus | |
| ARA67741.1 | Ali,M.A., ElShesheny,R., Kandeil,A., Shehata,M. | 2017-03-26T00:00:00Z | ssRNA(+) | 199 | spike protein | Egypt | Camelus | |
| ARA67742.1 | Ali,M.A., ElShesheny,R., Kandeil,A., Shehata,M. | 2017-03-26T00:00:00Z | ssRNA(+) | 200 | spike protein | Egypt | Camelus | |
| ARA67743.1 | Ali,M.A., ElShesheny,R., Kandeil,A., Shehata,M. | 2017-03-26T00:00:00Z | ssRNA(+) | 200 | spike protein | Egypt | Camelus | |
| APR62694.1 | van Doremalen,N., Hijazeen,Z.S., Holloway,P., Omari,B.A., McDowell,C., Adney,D., Talafha,H.A., Guitian,J., Steel,J., Amarin,N., Tibbo,M., Abu-Basha,E., Al-Majali,A.M., Munster,V.J., Richt,J.A., Hijazeen,Z.S.K., Al Omari,B. | 2017-01-03T00:00:00Z | ssRNA(+) | 205 | spike protein | Jordan | Camelus | |
| APR62695.1 | van Doremalen,N., Hijazeen,Z.S., Holloway,P., Omari,B.A., McDowell,C., Adney,D., Talafha,H.A., Guitian,J., Steel,J., Amarin,N., Tibbo,M., Abu-Basha,E., Al-Majali,A.M., Munster,V.J., Richt,J.A., Hijazeen,Z.S.K., Al Omari,B. | 2017-01-03T00:00:00Z | ssRNA(+) | 205 | spike protein | Jordan | Camelus | |
| APR62696.1 | van Doremalen,N., Hijazeen,Z.S., Holloway,P., Omari,B.A., McDowell,C., Adney,D., Talafha,H.A., Guitian,J., Steel,J., Amarin,N., Tibbo,M., Abu-Basha,E., Al-Majali,A.M., Munster,V.J., Richt,J.A., Hijazeen,Z.S.K., Al Omari,B. | 2017-01-03T00:00:00Z | ssRNA(+) | 205 | spike protein | Jordan | Camelus | |
| APR62697.1 | van Doremalen,N., Hijazeen,Z.S., Holloway,P., Omari,B.A., McDowell,C., Adney,D., Talafha,H.A., Guitian,J., Steel,J., Amarin,N., Tibbo,M., Abu-Basha,E., Al-Majali,A.M., Munster,V.J., Richt,J.A., Hijazeen,Z.S.K., Al Omari,B. | 2017-01-03T00:00:00Z | ssRNA(+) | 205 | spike protein | Jordan | Camelus |  |
| APR62698.1 | van Doremalen,N., Hijazeen,Z.S., Holloway,P., Omari,B.A., McDowell,C., Adney,D., Talafha,H.A., Guitian,J., Steel,J., Amarin,N., Tibbo,M., Abu-Basha,E., Al-Majali,A.M., Munster,V.J., Richt,J.A., Hijazeen,Z.S.K., Al Omari,B. | 2017-01-03T00:00:00Z | ssRNA(+) | 205 | spike protein | Jordan | Camelus |  |
| APR62699.1 | van Doremalen,N., Hijazeen,Z.S., Holloway,P., Omari,B.A., McDowell,C., Adney,D., Talafha,H.A., Guitian,J., Steel,J., Amarin,N., Tibbo,M., Abu-Basha,E., Al-Majali,A.M., Munster,V.J., Richt,J.A., Hijazeen,Z.S.K., Al Omari,B. | 2017-01-03T00:00:00Z | ssRNA(+) | 205 | spike protein | Jordan | Camelus |  |
| APR62700.1 | van Doremalen,N., Hijazeen,Z.S., Holloway,P., Omari,B.A., McDowell,C., Adney,D., Talafha,H.A., Guitian,J., Steel,J., Amarin,N., Tibbo,M., Abu-Basha,E., Al-Majali,A.M., Munster,V.J., Richt,J.A., Hijazeen,Z.S.K., Al Omari,B. | 2017-01-03T00:00:00Z | ssRNA(+) | 205 | spike protein | Jordan | Camelus |  |
| APR62701.1 | van Doremalen,N., Hijazeen,Z.S., Holloway,P., Omari,B.A., McDowell,C., Adney,D., Talafha,H.A., Guitian,J., Steel,J., Amarin,N., Tibbo,M., Abu-Basha,E., Al-Majali,A.M., Munster,V.J., Richt,J.A., Hijazeen,Z.S.K., Al Omari,B. | 2017-01-03T00:00:00Z | ssRNA(+) | 205 | spike protein | Jordan | Camelus |  |
| APR62702.1 | van Doremalen,N., Hijazeen,Z.S., Holloway,P., Omari,B.A., McDowell,C., Adney,D., Talafha,H.A., Guitian,J., Steel,J., Amarin,N., Tibbo,M., Abu-Basha,E., Al-Majali,A.M., Munster,V.J., Richt,J.A., Hijazeen,Z.S.K., Al Omari,B. | 2017-01-03T00:00:00Z | ssRNA(+) | 205 | spike protein | Jordan | Camelus |  |
| APR62703.1 | van Doremalen,N., Hijazeen,Z.S., Holloway,P., Omari,B.A., McDowell,C., Adney,D., Talafha,H.A., Guitian,J., Steel,J., Amarin,N., Tibbo,M., Abu-Basha,E., Al-Majali,A.M., Munster,V.J., Richt,J.A., Hijazeen,Z.S.K., Al Omari,B. | 2017-01-03T00:00:00Z | ssRNA(+) | 205 | spike protein | Jordan | Camelus |  |
| APR62704.1 | van Doremalen,N., Hijazeen,Z.S., Holloway,P., Omari,B.A., McDowell,C., Adney,D., Talafha,H.A., Guitian,J., Steel,J., Amarin,N., Tibbo,M., Abu-Basha,E., Al-Majali,A.M., Munster,V.J., Richt,J.A., Hijazeen,Z.S.K., Al Omari,B. | 2017-01-03T00:00:00Z | ssRNA(+) | 205 | spike protein | Jordan | Camelus |  |
| APR62705.1 | van Doremalen,N., Hijazeen,Z.S., Holloway,P., Omari,B.A., McDowell,C., Adney,D., Talafha,H.A., Guitian,J., Steel,J., Amarin,N., Tibbo,M., Abu-Basha,E., Al-Majali,A.M., Munster,V.J., Richt,J.A., Hijazeen,Z.S.K., Al Omari,B. | 2017-01-03T00:00:00Z | ssRNA(+) | 205 | spike protein | Jordan | Camelus |  |
| APR62706.1 | van Doremalen,N., Hijazeen,Z.S., Holloway,P., Omari,B.A., McDowell,C., Adney,D., Talafha,H.A., Guitian,J., Steel,J., Amarin,N., Tibbo,M., Abu-Basha,E., Al-Majali,A.M., Munster,V.J., Richt,J.A., Hijazeen,Z.S.K., Al Omari,B. | 2017-01-03T00:00:00Z | ssRNA(+) | 205 | spike protein | Jordan | Camelus |  |
| APR62707.1 | van Doremalen,N., Hijazeen,Z.S., Holloway,P., Omari,B.A., McDowell,C., Adney,D., Talafha,H.A., Guitian,J., Steel,J., Amarin,N., Tibbo,M., Abu-Basha,E., Al-Majali,A.M., Munster,V.J., Richt,J.A., Hijazeen,Z.S.K., Al Omari,B. | 2017-01-03T00:00:00Z | ssRNA(+) | 205 | spike protein | Jordan | Camelus |  |
| APR62708.1 | van Doremalen,N., Hijazeen,Z.S., Holloway,P., Omari,B.A., McDowell,C., Adney,D., Talafha,H.A., Guitian,J., Steel,J., Amarin,N., Tibbo,M., Abu-Basha,E., Al-Majali,A.M., Munster,V.J., Richt,J.A., Hijazeen,Z.S.K., Al Omari,B. | 2017-01-03T00:00:00Z | ssRNA(+) | 205 | spike protein | Jordan | Camelus |  |
| APR62709.1 | van Doremalen,N., Hijazeen,Z.S., Holloway,P., Omari,B.A., McDowell,C., Adney,D., Talafha,H.A., Guitian,J., Steel,J., Amarin,N., Tibbo,M., Abu-Basha,E., Al-Majali,A.M., Munster,V.J., Richt,J.A., Hijazeen,Z.S.K., Al Omari,B. | 2017-01-03T00:00:00Z | ssRNA(+) | 205 | spike protein | Jordan | Camelus |  |
| ANI69824.1 | Lau,S.K., Wernery,R., Wong,E.Y., Joseph,S., Tsang,A.K., Patteril,N.A., Elizabeth,S.K., Chan,K.H., Muhammed,R., Kinne,J., Yuen,K.Y., Wernery,U., Woo,P.C., Lau,S.K.P., Wong,E.Y.M., Tsang,A.K.L., Woo,P.C.Y. | 2016-12-29T00:00:00Z | ssRNA(+) | 1353 | spike protein | United Arab Emirates | Camelus dromedarius | |
| ANI69835.1 | Lau,S.K., Wernery,R., Wong,E.Y., Joseph,S., Tsang,A.K., Patteril,N.A., Elizabeth,S.K., Chan,K.H., Muhammed,R., Kinne,J., Yuen,K.Y., Wernery,U., Woo,P.C., Lau,S.K.P., Wong,E.Y.M., Tsang,A.K.L., Woo,P.C.Y. | 2016-12-29T00:00:00Z | ssRNA(+) | 1353 | spike protein | United Arab Emirates | Camelus dromedarius | |
| ANI69846.1 | Lau,S.K., Wernery,R., Wong,E.Y., Joseph,S., Tsang,A.K., Patteril,N.A., Elizabeth,S.K., Chan,K.H., Muhammed,R., Kinne,J., Yuen,K.Y., Wernery,U., Woo,P.C., Lau,S.K.P., Wong,E.Y.M., Tsang,A.K.L., Woo,P.C.Y. | 2016-12-29T00:00:00Z | ssRNA(+) | 1353 | spike protein | United Arab Emirates | Camelus dromedarius | |
| ANI69857.1 | Lau,S.K., Wernery,R., Wong,E.Y., Joseph,S., Tsang,A.K., Patteril,N.A., Elizabeth,S.K., Chan,K.H., Muhammed,R., Kinne,J., Yuen,K.Y., Wernery,U., Woo,P.C., Lau,S.K.P., Wong,E.Y.M., Tsang,A.K.L., Woo,P.C.Y. | 2016-12-29T00:00:00Z | ssRNA(+) | 1353 | spike protein | United Arab Emirates | Camelus dromedarius | |
| ANI69868.1 | Lau,S.K., Wernery,R., Wong,E.Y., Joseph,S., Tsang,A.K., Patteril,N.A., Elizabeth,S.K., Chan,K.H., Muhammed,R., Kinne,J., Yuen,K.Y., Wernery,U., Woo,P.C., Lau,S.K.P., Wong,E.Y.M., Tsang,A.K.L., Woo,P.C.Y. | 2016-12-29T00:00:00Z | ssRNA(+) | 1353 | spike protein | United Arab Emirates | Camelus dromedarius | |
| ANI69878.1 | Lau,S.K., Wernery,R., Wong,E.Y., Joseph,S., Tsang,A.K., Patteril,N.A., Elizabeth,S.K., Chan,K.H., Muhammed,R., Kinne,J., Yuen,K.Y., Wernery,U., Woo,P.C., Lau,S.K.P., Wong,E.Y.M., Tsang,A.K.L., Woo,P.C.Y. | 2016-12-29T00:00:00Z | ssRNA(+) | 1353 | spike protein | United Arab Emirates | Camelus dromedarius | |
| ANI69889.1 | Lau,S.K., Wernery,R., Wong,E.Y., Joseph,S., Tsang,A.K., Patteril,N.A., Elizabeth,S.K., Chan,K.H., Muhammed,R., Kinne,J., Yuen,K.Y., Wernery,U., Woo,P.C., Lau,S.K.P., Wong,E.Y.M., Tsang,A.K.L., Woo,P.C.Y. | 2016-12-29T00:00:00Z | ssRNA(+) | 1353 | spike protein | United Arab Emirates | Camelus dromedarius | |
| ANI69900.1 | Lau,S.K., Wernery,R., Wong,E.Y., Joseph,S., Tsang,A.K., Patteril,N.A., Elizabeth,S.K., Chan,K.H., Muhammed,R., Kinne,J., Yuen,K.Y., Wernery,U., Woo,P.C., Lau,S.K.P., Wong,E.Y.M., Tsang,A.K.L., Woo,P.C.Y. | 2016-12-29T00:00:00Z | ssRNA(+) | 1353 | spike protein | United Arab Emirates | Camelus dromedarius | |
| ANI69911.1 | Lau,S.K., Wernery,R., Wong,E.Y., Joseph,S., Tsang,A.K., Patteril,N.A., Elizabeth,S.K., Chan,K.H., Muhammed,R., Kinne,J., Yuen,K.Y., Wernery,U., Woo,P.C., Lau,S.K.P., Wong,E.Y.M., Tsang,A.K.L., Woo,P.C.Y. | 2016-12-29T00:00:00Z | ssRNA(+) | 1353 | spike protein | United Arab Emirates | Camelus dromedarius | |
| ANI69922.1 | Lau,S.K., Wernery,R., Wong,E.Y., Joseph,S., Tsang,A.K., Patteril,N.A., Elizabeth,S.K., Chan,K.H., Muhammed,R., Kinne,J., Yuen,K.Y., Wernery,U., Woo,P.C., Lau,S.K.P., Wong,E.Y.M., Tsang,A.K.L., Woo,P.C.Y. | 2016-12-29T00:00:00Z | ssRNA(+) | 1353 | spike protein | United Arab Emirates | Camelus dromedarius | |
| ALT66870.1 | Wernery,U., Corman,V.M., Wong,E.Y., Tsang,A.K., Muth,D., Lau,S.K., Khazanehdari,K., Zirkel,F., Ali,M., Nagy,P., Juhasz,J., Wernery,R., Joseph,S., Syriac,G., Elizabeth,S.K., Patteril,N.A., Woo,P.C., Drosten,C., Wong,E.Y.M., Tsang,A.K.L., Lau,S.K.P., Patteril,N.A.G., Woo,P.C.Y. | 2015-12-27T00:00:00Z | ssRNA(+) | 1353 | spike protein | United Arab Emirates | Camelus dromedarius | |
| ALT66880.1 | Wernery,U., Corman,V.M., Wong,E.Y., Tsang,A.K., Muth,D., Lau,S.K., Khazanehdari,K., Zirkel,F., Ali,M., Nagy,P., Juhasz,J., Wernery,R., Joseph,S., Syriac,G., Elizabeth,S.K., Patteril,N.A., Woo,P.C., Drosten,C., Wong,E.Y.M., Tsang,A.K.L., Lau,S.K.P., Patteril,N.A.G., Woo,P.C.Y. | 2015-12-27T00:00:00Z | ssRNA(+) | 1353 | spike protein | United Arab Emirates | Camelus dromedarius | |
| AKQ21055.1 | Al Hammadi,Z.M., Chu,D.K., Eltahir,Y.M., Al Hosani,F., Al Mulla,M., Tarnini,W., Hall,A.J., Perera,R.A., Abdelkhalek,M.M., Peiris,J.S., Al Muhairi,S.S., Poon,L.L., Chu,D.K.W., Peiris,M.J.S., Poon,L.L.M. | 2015-12-17T00:00:00Z | ssRNA(+) | 1353 | spike protein | United Arab Emirates | Camelus dromedarius | |
| AKQ21064.1 | Al Hammadi,Z.M., Chu,D.K., Eltahir,Y.M., Al Hosani,F., Al Mulla,M., Tarnini,W., Hall,A.J., Perera,R.A., Abdelkhalek,M.M., Peiris,J.S., Al Muhairi,S.S., Poon,L.L., Chu,D.K.W., Peiris,M.J.S., Poon,L.L.M. | 2015-12-17T00:00:00Z | ssRNA(+) | 1353 | spike protein | United Arab Emirates | Camelus dromedarius | |
| AKQ21073.1 | Al Hammadi,Z.M., Chu,D.K., Eltahir,Y.M., Al Hosani,F., Al Mulla,M., Tarnini,W., Hall,A.J., Perera,R.A., Abdelkhalek,M.M., Peiris,J.S., Al Muhairi,S.S., Poon,L.L., Chu,D.K.W., Peiris,M.J.S., Poon,L.L.M. | 2015-12-17T00:00:00Z | ssRNA(+) | 1353 | spike protein | United Arab Emirates | Camelus dromedarius | |
| ALA49341.1 | Sabir,J.S.M., Lam,T.Y., Ahmed,M.M., Li,L., Shen,Y., AboAba,S., Qureshi,M.I., Abu-Zaid,M., Zhang,Y., Khiyami,M.A., Alharbi,N.S., Hajrah,N.H., Sabir,M.J., Mutwakil,M.H.Z., Kabli,S.A., Alsulaimany,F.A.S., Obaid,A.Y., Zhou,B., Smith,D.K., Holmes,E.C., Zhu,H., Guan,Y. | 2015-12-17T00:00:00Z | ssRNA(+) | 1353 | spike protein | Saudi Arabia | Camelus |  |
| ALA49352.1 | Sabir,J.S.M., Lam,T.Y., Ahmed,M.M., Li,L., Shen,Y., AboAba,S., Qureshi,M.I., Abu-Zaid,M., Zhang,Y., Khiyami,M.A., Alharbi,N.S., Hajrah,N.H., Sabir,M.J., Mutwakil,M.H.Z., Kabli,S.A., Alsulaimany,F.A.S., Obaid,A.Y., Zhou,B., Smith,D.K., Holmes,E.C., Zhu,H., Guan,Y. | 2015-12-17T00:00:00Z | ssRNA(+) | 1353 | spike protein | Saudi Arabia | Camelus |  |
| ALA49363.1 | Sabir,J.S.M., Lam,T.Y., Ahmed,M.M., Li,L., Shen,Y., AboAba,S., Qureshi,M.I., Abu-Zaid,M., Zhang,Y., Khiyami,M.A., Alharbi,N.S., Hajrah,N.H., Sabir,M.J., Mutwakil,M.H.Z., Kabli,S.A., Alsulaimany,F.A.S., Obaid,A.Y., Zhou,B., Smith,D.K., Holmes,E.C., Zhu,H., Guan,Y. | 2015-12-17T00:00:00Z | ssRNA(+) | 1353 | spike protein | Saudi Arabia | Camelus |  |
| ALA49374.1 | Sabir,J.S.M., Lam,T.Y., Ahmed,M.M., Li,L., Shen,Y., AboAba,S., Qureshi,M.I., Abu-Zaid,M., Zhang,Y., Khiyami,M.A., Alharbi,N.S., Hajrah,N.H., Sabir,M.J., Mutwakil,M.H.Z., Kabli,S.A., Alsulaimany,F.A.S., Obaid,A.Y., Zhou,B., Smith,D.K., Holmes,E.C., Zhu,H., Guan,Y. | 2015-12-17T00:00:00Z | ssRNA(+) | 1353 | spike protein | Saudi Arabia | Camelus |  |
| ALA49385.1 | Sabir,J.S.M., Lam,T.Y., Ahmed,M.M., Li,L., Shen,Y., AboAba,S., Qureshi,M.I., Abu-Zaid,M., Zhang,Y., Khiyami,M.A., Alharbi,N.S., Hajrah,N.H., Sabir,M.J., Mutwakil,M.H.Z., Kabli,S.A., Alsulaimany,F.A.S., Obaid,A.Y., Zhou,B., Smith,D.K., Holmes,E.C., Zhu,H., Guan,Y. | 2015-12-17T00:00:00Z | ssRNA(+) | 1353 | spike protein | Saudi Arabia | Camelus |  |
| ALA49396.1 | Sabir,J.S.M., Lam,T.Y., Ahmed,M.M., Li,L., Shen,Y., AboAba,S., Qureshi,M.I., Abu-Zaid,M., Zhang,Y., Khiyami,M.A., Alharbi,N.S., Hajrah,N.H., Sabir,M.J., Mutwakil,M.H.Z., Kabli,S.A., Alsulaimany,F.A.S., Obaid,A.Y., Zhou,B., Smith,D.K., Holmes,E.C., Zhu,H., Guan,Y. | 2015-12-17T00:00:00Z | ssRNA(+) | 1353 | spike protein | Saudi Arabia | Camelus |  |
| ALA49407.1 | Sabir,J.S.M., Lam,T.Y., Ahmed,M.M., Li,L., Shen,Y., AboAba,S., Qureshi,M.I., Abu-Zaid,M., Zhang,Y., Khiyami,M.A., Alharbi,N.S., Hajrah,N.H., Sabir,M.J., Mutwakil,M.H.Z., Kabli,S.A., Alsulaimany,F.A.S., Obaid,A.Y., Zhou,B., Smith,D.K., Holmes,E.C., Zhu,H., Guan,Y. | 2015-12-17T00:00:00Z | ssRNA(+) | 1353 | spike protein | Saudi Arabia | Camelus |  |
| ALA49418.1 | Sabir,J.S.M., Lam,T.Y., Ahmed,M.M., Li,L., Shen,Y., AboAba,S., Qureshi,M.I., Abu-Zaid,M., Zhang,Y., Khiyami,M.A., Alharbi,N.S., Hajrah,N.H., Sabir,M.J., Mutwakil,M.H.Z., Kabli,S.A., Alsulaimany,F.A.S., Obaid,A.Y., Zhou,B., Smith,D.K., Holmes,E.C., Zhu,H., Guan,Y. | 2015-12-17T00:00:00Z | ssRNA(+) | 1353 | spike protein | Saudi Arabia | Camelus |  |
| ALA49429.1 | Sabir,J.S.M., Lam,T.Y., Ahmed,M.M., Li,L., Shen,Y., AboAba,S., Qureshi,M.I., Abu-Zaid,M., Zhang,Y., Khiyami,M.A., Alharbi,N.S., Hajrah,N.H., Sabir,M.J., Mutwakil,M.H.Z., Kabli,S.A., Alsulaimany,F.A.S., Obaid,A.Y., Zhou,B., Smith,D.K., Holmes,E.C., Zhu,H., Guan,Y. | 2015-12-17T00:00:00Z | ssRNA(+) | 1353 | spike protein | Saudi Arabia | Camelus |  |
| ALA49440.1 | Sabir,J.S.M., Lam,T.Y., Ahmed,M.M., Li,L., Shen,Y., AboAba,S., Qureshi,M.I., Abu-Zaid,M., Zhang,Y., Khiyami,M.A., Alharbi,N.S., Hajrah,N.H., Sabir,M.J., Mutwakil,M.H.Z., Kabli,S.A., Alsulaimany,F.A.S., Obaid,A.Y., Zhou,B., Smith,D.K., Holmes,E.C., Zhu,H., Guan,Y. | 2015-12-17T00:00:00Z | ssRNA(+) | 1353 | spike protein | Saudi Arabia | Camelus |  |
| ALA49451.1 | Sabir,J.S.M., Lam,T.Y., Ahmed,M.M., Li,L., Shen,Y., AboAba,S., Qureshi,M.I., Abu-Zaid,M., Zhang,Y., Khiyami,M.A., Alharbi,N.S., Hajrah,N.H., Sabir,M.J., Mutwakil,M.H.Z., Kabli,S.A., Alsulaimany,F.A.S., Obaid,A.Y., Zhou,B., Smith,D.K., Holmes,E.C., Zhu,H., Guan,Y. | 2015-12-17T00:00:00Z | ssRNA(+) | 1353 | spike protein | Saudi Arabia | Camelus |  |
| ALA49462.1 | Sabir,J.S.M., Lam,T.Y., Ahmed,M.M., Li,L., Shen,Y., AboAba,S., Qureshi,M.I., Abu-Zaid,M., Zhang,Y., Khiyami,M.A., Alharbi,N.S., Hajrah,N.H., Sabir,M.J., Mutwakil,M.H.Z., Kabli,S.A., Alsulaimany,F.A.S., Obaid,A.Y., Zhou,B., Smith,D.K., Holmes,E.C., Zhu,H., Guan,Y. | 2015-12-17T00:00:00Z | ssRNA(+) | 1353 | spike protein | Saudi Arabia | Camelus |  |
| ALA49473.1 | Sabir,J.S.M., Lam,T.Y., Ahmed,M.M., Li,L., Shen,Y., AboAba,S., Qureshi,M.I., Abu-Zaid,M., Zhang,Y., Khiyami,M.A., Alharbi,N.S., Hajrah,N.H., Sabir,M.J., Mutwakil,M.H.Z., Kabli,S.A., Alsulaimany,F.A.S., Obaid,A.Y., Zhou,B., Smith,D.K., Holmes,E.C., Zhu,H., Guan,Y. | 2015-12-17T00:00:00Z | ssRNA(+) | 1353 | spike protein | Saudi Arabia | Camelus |  |
| ALA49484.1 | Sabir,J.S.M., Lam,T.Y., Ahmed,M.M., Li,L., Shen,Y., AboAba,S., Qureshi,M.I., Abu-Zaid,M., Zhang,Y., Khiyami,M.A., Alharbi,N.S., Hajrah,N.H., Sabir,M.J., Mutwakil,M.H.Z., Kabli,S.A., Alsulaimany,F.A.S., Obaid,A.Y., Zhou,B., Smith,D.K., Holmes,E.C., Zhu,H., Guan,Y. | 2015-12-17T00:00:00Z | ssRNA(+) | 1353 | spike protein | Saudi Arabia | Camelus |  |
| ALA49495.1 | Sabir,J.S.M., Lam,T.Y., Ahmed,M.M., Li,L., Shen,Y., AboAba,S., Qureshi,M.I., Abu-Zaid,M., Zhang,Y., Khiyami,M.A., Alharbi,N.S., Hajrah,N.H., Sabir,M.J., Mutwakil,M.H.Z., Kabli,S.A., Alsulaimany,F.A.S., Obaid,A.Y., Zhou,B., Smith,D.K., Holmes,E.C., Zhu,H., Guan,Y. | 2015-12-17T00:00:00Z | ssRNA(+) | 1353 | spike protein | Saudi Arabia | Camelus |  |
| ALA49506.1 | Sabir,J.S.M., Lam,T.Y., Ahmed,M.M., Li,L., Shen,Y., AboAba,S., Qureshi,M.I., Abu-Zaid,M., Zhang,Y., Khiyami,M.A., Alharbi,N.S., Hajrah,N.H., Sabir,M.J., Mutwakil,M.H.Z., Kabli,S.A., Alsulaimany,F.A.S., Obaid,A.Y., Zhou,B., Smith,D.K., Holmes,E.C., Zhu,H., Guan,Y. | 2015-12-17T00:00:00Z | ssRNA(+) | 1353 | spike protein | Saudi Arabia | Camelus |  |
| ALA49517.1 | Sabir,J.S.M., Lam,T.Y., Ahmed,M.M., Li,L., Shen,Y., AboAba,S., Qureshi,M.I., Abu-Zaid,M., Zhang,Y., Khiyami,M.A., Alharbi,N.S., Hajrah,N.H., Sabir,M.J., Mutwakil,M.H.Z., Kabli,S.A., Alsulaimany,F.A.S., Obaid,A.Y., Zhou,B., Smith,D.K., Holmes,E.C., Zhu,H., Guan,Y. | 2015-12-17T00:00:00Z | ssRNA(+) | 1353 | spike protein | Saudi Arabia | Camelus |  |
| ALA49528.1 | Sabir,J.S.M., Lam,T.Y., Ahmed,M.M., Li,L., Shen,Y., AboAba,S., Qureshi,M.I., Abu-Zaid,M., Zhang,Y., Khiyami,M.A., Alharbi,N.S., Hajrah,N.H., Sabir,M.J., Mutwakil,M.H.Z., Kabli,S.A., Alsulaimany,F.A.S., Obaid,A.Y., Zhou,B., Smith,D.K., Holmes,E.C., Zhu,H., Guan,Y. | 2015-12-17T00:00:00Z | ssRNA(+) | 1353 | spike protein | Saudi Arabia | Camelus |  |
| ALA49539.1 | Sabir,J.S.M., Lam,T.Y., Ahmed,M.M., Li,L., Shen,Y., AboAba,S., Qureshi,M.I., Abu-Zaid,M., Zhang,Y., Khiyami,M.A., Alharbi,N.S., Hajrah,N.H., Sabir,M.J., Mutwakil,M.H.Z., Kabli,S.A., Alsulaimany,F.A.S., Obaid,A.Y., Zhou,B., Smith,D.K., Holmes,E.C., Zhu,H., Guan,Y. | 2015-12-17T00:00:00Z | ssRNA(+) | 1353 | spike protein | Saudi Arabia | Camelus |  |
| ALA49550.1 | Sabir,J.S.M., Lam,T.Y., Ahmed,M.M., Li,L., Shen,Y., AboAba,S., Qureshi,M.I., Abu-Zaid,M., Zhang,Y., Khiyami,M.A., Alharbi,N.S., Hajrah,N.H., Sabir,M.J., Mutwakil,M.H.Z., Kabli,S.A., Alsulaimany,F.A.S., Obaid,A.Y., Zhou,B., Smith,D.K., Holmes,E.C., Zhu,H., Guan,Y. | 2015-12-17T00:00:00Z | ssRNA(+) | 1353 | spike protein | Saudi Arabia | Camelus |  |
| ALA49561.1 | Sabir,J.S.M., Lam,T.Y., Ahmed,M.M., Li,L., Shen,Y., AboAba,S., Qureshi,M.I., Abu-Zaid,M., Zhang,Y., Khiyami,M.A., Alharbi,N.S., Hajrah,N.H., Sabir,M.J., Mutwakil,M.H.Z., Kabli,S.A., Alsulaimany,F.A.S., Obaid,A.Y., Zhou,B., Smith,D.K., Holmes,E.C., Zhu,H., Guan,Y. | 2015-12-17T00:00:00Z | ssRNA(+) | 1353 | spike protein | Saudi Arabia | Camelus |  |
| ALA49572.1 | Sabir,J.S.M., Lam,T.Y., Ahmed,M.M., Li,L., Shen,Y., AboAba,S., Qureshi,M.I., Abu-Zaid,M., Zhang,Y., Khiyami,M.A., Alharbi,N.S., Hajrah,N.H., Sabir,M.J., Mutwakil,M.H.Z., Kabli,S.A., Alsulaimany,F.A.S., Obaid,A.Y., Zhou,B., Smith,D.K., Holmes,E.C., Zhu,H., Guan,Y. | 2015-12-17T00:00:00Z | ssRNA(+) | 1353 | spike protein | Saudi Arabia | Camelus |  |
| ALA49583.1 | Sabir,J.S.M., Lam,T.Y., Ahmed,M.M., Li,L., Shen,Y., AboAba,S., Qureshi,M.I., Abu-Zaid,M., Zhang,Y., Khiyami,M.A., Alharbi,N.S., Hajrah,N.H., Sabir,M.J., Mutwakil,M.H.Z., Kabli,S.A., Alsulaimany,F.A.S., Obaid,A.Y., Zhou,B., Smith,D.K., Holmes,E.C., Zhu,H., Guan,Y. | 2015-12-17T00:00:00Z | ssRNA(+) | 1353 | spike protein | Saudi Arabia | Camelus |  |
| ALA49594.1 | Sabir,J.S.M., Lam,T.Y., Ahmed,M.M., Li,L., Shen,Y., AboAba,S., Qureshi,M.I., Abu-Zaid,M., Zhang,Y., Khiyami,M.A., Alharbi,N.S., Hajrah,N.H., Sabir,M.J., Mutwakil,M.H.Z., Kabli,S.A., Alsulaimany,F.A.S., Obaid,A.Y., Zhou,B., Smith,D.K., Holmes,E.C., Zhu,H., Guan,Y. | 2015-12-17T00:00:00Z | ssRNA(+) | 1353 | spike protein | Saudi Arabia | Camelus |  |
| ALA49605.1 | Sabir,J.S.M., Lam,T.Y., Ahmed,M.M., Li,L., Shen,Y., AboAba,S., Qureshi,M.I., Abu-Zaid,M., Zhang,Y., Khiyami,M.A., Alharbi,N.S., Hajrah,N.H., Sabir,M.J., Mutwakil,M.H.Z., Kabli,S.A., Alsulaimany,F.A.S., Obaid,A.Y., Zhou,B., Smith,D.K., Holmes,E.C., Zhu,H., Guan,Y. | 2015-12-17T00:00:00Z | ssRNA(+) | 1353 | spike protein | Saudi Arabia | Camelus |  |
| ALA49616.1 | Sabir,J.S.M., Lam,T.Y., Ahmed,M.M., Li,L., Shen,Y., AboAba,S., Qureshi,M.I., Abu-Zaid,M., Zhang,Y., Khiyami,M.A., Alharbi,N.S., Hajrah,N.H., Sabir,M.J., Mutwakil,M.H.Z., Kabli,S.A., Alsulaimany,F.A.S., Obaid,A.Y., Zhou,B., Smith,D.K., Holmes,E.C., Zhu,H., Guan,Y. | 2015-12-17T00:00:00Z | ssRNA(+) | 1353 | spike protein | Saudi Arabia | Camelus |  |
| ALA49627.1 | Sabir,J.S.M., Lam,T.Y., Ahmed,M.M., Li,L., Shen,Y., AboAba,S., Qureshi,M.I., Abu-Zaid,M., Zhang,Y., Khiyami,M.A., Alharbi,N.S., Hajrah,N.H., Sabir,M.J., Mutwakil,M.H.Z., Kabli,S.A., Alsulaimany,F.A.S., Obaid,A.Y., Zhou,B., Smith,D.K., Holmes,E.C., Zhu,H., Guan,Y. | 2015-12-17T00:00:00Z | ssRNA(+) | 1353 | spike protein | Saudi Arabia | Camelus |  |
| ALA49638.1 | Sabir,J.S.M., Lam,T.Y., Ahmed,M.M., Li,L., Shen,Y., AboAba,S., Qureshi,M.I., Abu-Zaid,M., Zhang,Y., Khiyami,M.A., Alharbi,N.S., Hajrah,N.H., Sabir,M.J., Mutwakil,M.H.Z., Kabli,S.A., Alsulaimany,F.A.S., Obaid,A.Y., Zhou,B., Smith,D.K., Holmes,E.C., Zhu,H., Guan,Y. | 2015-12-17T00:00:00Z | ssRNA(+) | 1353 | spike protein | Saudi Arabia | Camelus |  |
| ALA49649.1 | Sabir,J.S.M., Lam,T.Y., Ahmed,M.M., Li,L., Shen,Y., AboAba,S., Qureshi,M.I., Abu-Zaid,M., Zhang,Y., Khiyami,M.A., Alharbi,N.S., Hajrah,N.H., Sabir,M.J., Mutwakil,M.H.Z., Kabli,S.A., Alsulaimany,F.A.S., Obaid,A.Y., Zhou,B., Smith,D.K., Holmes,E.C., Zhu,H., Guan,Y. | 2015-12-17T00:00:00Z | ssRNA(+) | 1353 | spike protein | Saudi Arabia | Camelus |  |
| ALA49660.1 | Sabir,J.S.M., Lam,T.Y., Ahmed,M.M., Li,L., Shen,Y., AboAba,S., Qureshi,M.I., Abu-Zaid,M., Zhang,Y., Khiyami,M.A., Alharbi,N.S., Hajrah,N.H., Sabir,M.J., Mutwakil,M.H.Z., Kabli,S.A., Alsulaimany,F.A.S., Obaid,A.Y., Zhou,B., Smith,D.K., Holmes,E.C., Zhu,H., Guan,Y. | 2015-12-17T00:00:00Z | ssRNA(+) | 1353 | spike protein | Saudi Arabia | Camelus |  |
| ALA49671.1 | Sabir,J.S.M., Lam,T.Y., Ahmed,M.M., Li,L., Shen,Y., AboAba,S., Qureshi,M.I., Abu-Zaid,M., Zhang,Y., Khiyami,M.A., Alharbi,N.S., Hajrah,N.H., Sabir,M.J., Mutwakil,M.H.Z., Kabli,S.A., Alsulaimany,F.A.S., Obaid,A.Y., Zhou,B., Smith,D.K., Holmes,E.C., Zhu,H., Guan,Y. | 2015-12-17T00:00:00Z | ssRNA(+) | 1353 | spike protein | Saudi Arabia | Camelus |  |
| ALA49682.1 | Sabir,J.S.M., Lam,T.Y., Ahmed,M.M., Li,L., Shen,Y., AboAba,S., Qureshi,M.I., Abu-Zaid,M., Zhang,Y., Khiyami,M.A., Alharbi,N.S., Hajrah,N.H., Sabir,M.J., Mutwakil,M.H.Z., Kabli,S.A., Alsulaimany,F.A.S., Obaid,A.Y., Zhou,B., Smith,D.K., Holmes,E.C., Zhu,H., Guan,Y. | 2015-12-17T00:00:00Z | ssRNA(+) | 1353 | spike protein | Saudi Arabia | Camelus |  |
| ALA49693.1 | Sabir,J.S.M., Lam,T.Y., Ahmed,M.M., Li,L., Shen,Y., AboAba,S., Qureshi,M.I., Abu-Zaid,M., Zhang,Y., Khiyami,M.A., Alharbi,N.S., Hajrah,N.H., Sabir,M.J., Mutwakil,M.H.Z., Kabli,S.A., Alsulaimany,F.A.S., Obaid,A.Y., Zhou,B., Smith,D.K., Holmes,E.C., Zhu,H., Guan,Y. | 2015-12-17T00:00:00Z | ssRNA(+) | 1353 | spike protein | Saudi Arabia | Camelus |  |
| ALA49704.1 | Sabir,J.S.M., Lam,T.Y., Ahmed,M.M., Li,L., Shen,Y., AboAba,S., Qureshi,M.I., Abu-Zaid,M., Zhang,Y., Khiyami,M.A., Alharbi,N.S., Hajrah,N.H., Sabir,M.J., Mutwakil,M.H.Z., Kabli,S.A., Alsulaimany,F.A.S., Obaid,A.Y., Zhou,B., Smith,D.K., Holmes,E.C., Zhu,H., Guan,Y. | 2015-12-17T00:00:00Z | ssRNA(+) | 1353 | spike protein | Saudi Arabia | Camelus |  |
| ALA49715.1 | Sabir,J.S.M., Lam,T.Y., Ahmed,M.M., Li,L., Shen,Y., AboAba,S., Qureshi,M.I., Abu-Zaid,M., Zhang,Y., Khiyami,M.A., Alharbi,N.S., Hajrah,N.H., Sabir,M.J., Mutwakil,M.H.Z., Kabli,S.A., Alsulaimany,F.A.S., Obaid,A.Y., Zhou,B., Smith,D.K., Holmes,E.C., Zhu,H., Guan,Y. | 2015-12-17T00:00:00Z | ssRNA(+) | 1353 | spike protein | Saudi Arabia | Camelus |  |
| ALA49726.1 | Sabir,J.S.M., Lam,T.Y., Ahmed,M.M., Li,L., Shen,Y., AboAba,S., Qureshi,M.I., Abu-Zaid,M., Zhang,Y., Khiyami,M.A., Alharbi,N.S., Hajrah,N.H., Sabir,M.J., Mutwakil,M.H.Z., Kabli,S.A., Alsulaimany,F.A.S., Obaid,A.Y., Zhou,B., Smith,D.K., Holmes,E.C., Zhu,H., Guan,Y. | 2015-12-17T00:00:00Z | ssRNA(+) | 1353 | spike protein | Saudi Arabia | Camelus |  |
| ALA49737.1 | Sabir,J.S.M., Lam,T.Y., Ahmed,M.M., Li,L., Shen,Y., AboAba,S., Qureshi,M.I., Abu-Zaid,M., Zhang,Y., Khiyami,M.A., Alharbi,N.S., Hajrah,N.H., Sabir,M.J., Mutwakil,M.H.Z., Kabli,S.A., Alsulaimany,F.A.S., Obaid,A.Y., Zhou,B., Smith,D.K., Holmes,E.C., Zhu,H., Guan,Y. | 2015-12-17T00:00:00Z | ssRNA(+) | 1353 | spike protein | Saudi Arabia | Camelus |  |
| ALA49748.1 | Sabir,J.S.M., Lam,T.Y., Ahmed,M.M., Li,L., Shen,Y., AboAba,S., Qureshi,M.I., Abu-Zaid,M., Zhang,Y., Khiyami,M.A., Alharbi,N.S., Hajrah,N.H., Sabir,M.J., Mutwakil,M.H.Z., Kabli,S.A., Alsulaimany,F.A.S., Obaid,A.Y., Zhou,B., Smith,D.K., Holmes,E.C., Zhu,H., Guan,Y. | 2015-12-17T00:00:00Z | ssRNA(+) | 1353 | spike protein | Saudi Arabia | Camelus |  |
| ALA49759.1 | Sabir,J.S.M., Lam,T.Y., Ahmed,M.M., Li,L., Shen,Y., AboAba,S., Qureshi,M.I., Abu-Zaid,M., Zhang,Y., Khiyami,M.A., Alharbi,N.S., Hajrah,N.H., Sabir,M.J., Mutwakil,M.H.Z., Kabli,S.A., Alsulaimany,F.A.S., Obaid,A.Y., Zhou,B., Smith,D.K., Holmes,E.C., Zhu,H., Guan,Y. | 2015-12-17T00:00:00Z | ssRNA(+) | 1353 | spike protein | Saudi Arabia | Camelus |  |
| ALA49770.1 | Sabir,J.S.M., Lam,T.Y., Ahmed,M.M., Li,L., Shen,Y., AboAba,S., Qureshi,M.I., Abu-Zaid,M., Zhang,Y., Khiyami,M.A., Alharbi,N.S., Hajrah,N.H., Sabir,M.J., Mutwakil,M.H.Z., Kabli,S.A., Alsulaimany,F.A.S., Obaid,A.Y., Zhou,B., Smith,D.K., Holmes,E.C., Zhu,H., Guan,Y. | 2015-12-17T00:00:00Z | ssRNA(+) | 1353 | spike protein | Saudi Arabia | Camelus |  |
| ALA49781.1 | Sabir,J.S.M., Lam,T.Y., Ahmed,M.M., Li,L., Shen,Y., AboAba,S., Qureshi,M.I., Abu-Zaid,M., Zhang,Y., Khiyami,M.A., Alharbi,N.S., Hajrah,N.H., Sabir,M.J., Mutwakil,M.H.Z., Kabli,S.A., Alsulaimany,F.A.S., Obaid,A.Y., Zhou,B., Smith,D.K., Holmes,E.C., Zhu,H., Guan,Y. | 2015-12-17T00:00:00Z | ssRNA(+) | 1353 | spike protein | Saudi Arabia | Camelus |  |
| ALA49792.1 | Sabir,J.S.M., Lam,T.Y., Ahmed,M.M., Li,L., Shen,Y., AboAba,S., Qureshi,M.I., Abu-Zaid,M., Zhang,Y., Khiyami,M.A., Alharbi,N.S., Hajrah,N.H., Sabir,M.J., Mutwakil,M.H.Z., Kabli,S.A., Alsulaimany,F.A.S., Obaid,A.Y., Zhou,B., Smith,D.K., Holmes,E.C., Zhu,H., Guan,Y. | 2015-12-17T00:00:00Z | ssRNA(+) | 1353 | spike protein | Saudi Arabia | Camelus |  |
| ALA49803.1 | Sabir,J.S.M., Lam,T.Y., Ahmed,M.M., Li,L., Shen,Y., AboAba,S., Qureshi,M.I., Abu-Zaid,M., Zhang,Y., Khiyami,M.A., Alharbi,N.S., Hajrah,N.H., Sabir,M.J., Mutwakil,M.H.Z., Kabli,S.A., Alsulaimany,F.A.S., Obaid,A.Y., Zhou,B., Smith,D.K., Holmes,E.C., Zhu,H., Guan,Y. | 2015-12-17T00:00:00Z | ssRNA(+) | 1353 | spike protein | Saudi Arabia | Camelus |  |
| ALA49814.1 | Sabir,J.S.M., Lam,T.Y., Ahmed,M.M., Li,L., Shen,Y., AboAba,S., Qureshi,M.I., Abu-Zaid,M., Zhang,Y., Khiyami,M.A., Alharbi,N.S., Hajrah,N.H., Sabir,M.J., Mutwakil,M.H.Z., Kabli,S.A., Alsulaimany,F.A.S., Obaid,A.Y., Zhou,B., Smith,D.K., Holmes,E.C., Zhu,H., Guan,Y. | 2015-12-17T00:00:00Z | ssRNA(+) | 1353 | spike protein | Saudi Arabia | Camelus |  |
| ALA49825.1 | Sabir,J.S.M., Lam,T.Y., Ahmed,M.M., Li,L., Shen,Y., AboAba,S., Qureshi,M.I., Abu-Zaid,M., Zhang,Y., Khiyami,M.A., Alharbi,N.S., Hajrah,N.H., Sabir,M.J., Mutwakil,M.H.Z., Kabli,S.A., Alsulaimany,F.A.S., Obaid,A.Y., Zhou,B., Smith,D.K., Holmes,E.C., Zhu,H., Guan,Y. | 2015-12-17T00:00:00Z | ssRNA(+) | 1353 | spike protein | Saudi Arabia | Camelus |  |
| ALA49836.1 | Sabir,J.S.M., Lam,T.Y., Ahmed,M.M., Li,L., Shen,Y., AboAba,S., Qureshi,M.I., Abu-Zaid,M., Zhang,Y., Khiyami,M.A., Alharbi,N.S., Hajrah,N.H., Sabir,M.J., Mutwakil,M.H.Z., Kabli,S.A., Alsulaimany,F.A.S., Obaid,A.Y., Zhou,B., Smith,D.K., Holmes,E.C., Zhu,H., Guan,Y. | 2015-12-17T00:00:00Z | ssRNA(+) | 1353 | spike protein | Saudi Arabia | Camelus |  |
| ALA49847.1 | Sabir,J.S.M., Lam,T.Y., Ahmed,M.M., Li,L., Shen,Y., AboAba,S., Qureshi,M.I., Abu-Zaid,M., Zhang,Y., Khiyami,M.A., Alharbi,N.S., Hajrah,N.H., Sabir,M.J., Mutwakil,M.H.Z., Kabli,S.A., Alsulaimany,F.A.S., Obaid,A.Y., Zhou,B., Smith,D.K., Holmes,E.C., Zhu,H., Guan,Y. | 2015-12-17T00:00:00Z | ssRNA(+) | 1353 | spike protein | Saudi Arabia | Camelus |  |
| ALA49858.1 | Sabir,J.S.M., Lam,T.Y., Ahmed,M.M., Li,L., Shen,Y., AboAba,S., Qureshi,M.I., Abu-Zaid,M., Zhang,Y., Khiyami,M.A., Alharbi,N.S., Hajrah,N.H., Sabir,M.J., Mutwakil,M.H.Z., Kabli,S.A., Alsulaimany,F.A.S., Obaid,A.Y., Zhou,B., Smith,D.K., Holmes,E.C., Zhu,H., Guan,Y. | 2015-12-17T00:00:00Z | ssRNA(+) | 1353 | spike protein | Saudi Arabia | Camelus |  |
| ALA49869.1 | Sabir,J.S.M., Lam,T.Y., Ahmed,M.M., Li,L., Shen,Y., AboAba,S., Qureshi,M.I., Abu-Zaid,M., Zhang,Y., Khiyami,M.A., Alharbi,N.S., Hajrah,N.H., Sabir,M.J., Mutwakil,M.H.Z., Kabli,S.A., Alsulaimany,F.A.S., Obaid,A.Y., Zhou,B., Smith,D.K., Holmes,E.C., Zhu,H., Guan,Y. | 2015-12-17T00:00:00Z | ssRNA(+) | 1353 | spike protein | Saudi Arabia | Camelus |  |
| ALA49880.1 | Sabir,J.S.M., Lam,T.Y., Ahmed,M.M., Li,L., Shen,Y., AboAba,S., Qureshi,M.I., Abu-Zaid,M., Zhang,Y., Khiyami,M.A., Alharbi,N.S., Hajrah,N.H., Sabir,M.J., Mutwakil,M.H.Z., Kabli,S.A., Alsulaimany,F.A.S., Obaid,A.Y., Zhou,B., Smith,D.K., Holmes,E.C., Zhu,H., Guan,Y. | 2015-12-17T00:00:00Z | ssRNA(+) | 1353 | spike protein | Saudi Arabia | Camelus |  |
| ALA49891.1 | Sabir,J.S.M., Lam,T.Y., Ahmed,M.M., Li,L., Shen,Y., AboAba,S., Qureshi,M.I., Abu-Zaid,M., Zhang,Y., Khiyami,M.A., Alharbi,N.S., Hajrah,N.H., Sabir,M.J., Mutwakil,M.H.Z., Kabli,S.A., Alsulaimany,F.A.S., Obaid,A.Y., Zhou,B., Smith,D.K., Holmes,E.C., Zhu,H., Guan,Y. | 2015-12-17T00:00:00Z | ssRNA(+) | 1353 | spike protein | Saudi Arabia | Camelus |  |
| ALA49902.1 | Sabir,J.S.M., Lam,T.Y., Ahmed,M.M., Li,L., Shen,Y., AboAba,S., Qureshi,M.I., Abu-Zaid,M., Zhang,Y., Khiyami,M.A., Alharbi,N.S., Hajrah,N.H., Sabir,M.J., Mutwakil,M.H.Z., Kabli,S.A., Alsulaimany,F.A.S., Obaid,A.Y., Zhou,B., Smith,D.K., Holmes,E.C., Zhu,H., Guan,Y. | 2015-12-17T00:00:00Z | ssRNA(+) | 1353 | spike protein | Saudi Arabia | Camelus |  |
| ALA49913.1 | Sabir,J.S.M., Lam,T.Y., Ahmed,M.M., Li,L., Shen,Y., AboAba,S., Qureshi,M.I., Abu-Zaid,M., Zhang,Y., Khiyami,M.A., Alharbi,N.S., Hajrah,N.H., Sabir,M.J., Mutwakil,M.H.Z., Kabli,S.A., Alsulaimany,F.A.S., Obaid,A.Y., Zhou,B., Smith,D.K., Holmes,E.C., Zhu,H., Guan,Y. | 2015-12-17T00:00:00Z | ssRNA(+) | 1353 | spike protein | Saudi Arabia | Camelus |  |
| ALA49924.1 | Sabir,J.S.M., Lam,T.Y., Ahmed,M.M., Li,L., Shen,Y., AboAba,S., Qureshi,M.I., Abu-Zaid,M., Zhang,Y., Khiyami,M.A., Alharbi,N.S., Hajrah,N.H., Sabir,M.J., Mutwakil,M.H.Z., Kabli,S.A., Alsulaimany,F.A.S., Obaid,A.Y., Zhou,B., Smith,D.K., Holmes,E.C., Zhu,H., Guan,Y. | 2015-12-17T00:00:00Z | ssRNA(+) | 1353 | spike protein | Saudi Arabia | Camelus |  |
| ALA49935.1 | Sabir,J.S.M., Lam,T.Y., Ahmed,M.M., Li,L., Shen,Y., AboAba,S., Qureshi,M.I., Abu-Zaid,M., Zhang,Y., Khiyami,M.A., Alharbi,N.S., Hajrah,N.H., Sabir,M.J., Mutwakil,M.H.Z., Kabli,S.A., Alsulaimany,F.A.S., Obaid,A.Y., Zhou,B., Smith,D.K., Holmes,E.C., Zhu,H., Guan,Y. | 2015-12-17T00:00:00Z | ssRNA(+) | 1353 | spike protein | Saudi Arabia | Camelus |  |
| ALA49946.1 | Sabir,J.S.M., Lam,T.Y., Ahmed,M.M., Li,L., Shen,Y., AboAba,S., Qureshi,M.I., Abu-Zaid,M., Zhang,Y., Khiyami,M.A., Alharbi,N.S., Hajrah,N.H., Sabir,M.J., Mutwakil,M.H.Z., Kabli,S.A., Alsulaimany,F.A.S., Obaid,A.Y., Zhou,B., Smith,D.K., Holmes,E.C., Zhu,H., Guan,Y. | 2015-12-17T00:00:00Z | ssRNA(+) | 1353 | spike protein | Saudi Arabia | Camelus |  |
| ALA49957.1 | Sabir,J.S.M., Lam,T.Y., Ahmed,M.M., Li,L., Shen,Y., AboAba,S., Qureshi,M.I., Abu-Zaid,M., Zhang,Y., Khiyami,M.A., Alharbi,N.S., Hajrah,N.H., Sabir,M.J., Mutwakil,M.H.Z., Kabli,S.A., Alsulaimany,F.A.S., Obaid,A.Y., Zhou,B., Smith,D.K., Holmes,E.C., Zhu,H., Guan,Y. | 2015-12-17T00:00:00Z | ssRNA(+) | 1353 | spike protein | Saudi Arabia | Camelus |  |
| ALA49968.1 | Sabir,J.S.M., Lam,T.Y., Ahmed,M.M., Li,L., Shen,Y., AboAba,S., Qureshi,M.I., Abu-Zaid,M., Zhang,Y., Khiyami,M.A., Alharbi,N.S., Hajrah,N.H., Sabir,M.J., Mutwakil,M.H.Z., Kabli,S.A., Alsulaimany,F.A.S., Obaid,A.Y., Zhou,B., Smith,D.K., Holmes,E.C., Zhu,H., Guan,Y. | 2015-12-17T00:00:00Z | ssRNA(+) | 1353 | spike protein | Saudi Arabia | Camelus |  |
| ALA49979.1 | Sabir,J.S.M., Lam,T.Y., Ahmed,M.M., Li,L., Shen,Y., AboAba,S., Qureshi,M.I., Abu-Zaid,M., Zhang,Y., Khiyami,M.A., Alharbi,N.S., Hajrah,N.H., Sabir,M.J., Mutwakil,M.H.Z., Kabli,S.A., Alsulaimany,F.A.S., Obaid,A.Y., Zhou,B., Smith,D.K., Holmes,E.C., Zhu,H., Guan,Y. | 2015-12-17T00:00:00Z | ssRNA(+) | 1353 | spike protein | Saudi Arabia | Camelus |  |
| ALA49990.1 | Sabir,J.S.M., Lam,T.Y., Ahmed,M.M., Li,L., Shen,Y., AboAba,S., Qureshi,M.I., Abu-Zaid,M., Zhang,Y., Khiyami,M.A., Alharbi,N.S., Hajrah,N.H., Sabir,M.J., Mutwakil,M.H.Z., Kabli,S.A., Alsulaimany,F.A.S., Obaid,A.Y., Zhou,B., Smith,D.K., Holmes,E.C., Zhu,H., Guan,Y. | 2015-12-17T00:00:00Z | ssRNA(+) | 1353 | spike protein | Saudi Arabia | Camelus |  |
| ALA50001.1 | Sabir,J.S.M., Lam,T.Y., Ahmed,M.M., Li,L., Shen,Y., AboAba,S., Qureshi,M.I., Abu-Zaid,M., Zhang,Y., Khiyami,M.A., Alharbi,N.S., Hajrah,N.H., Sabir,M.J., Mutwakil,M.H.Z., Kabli,S.A., Alsulaimany,F.A.S., Obaid,A.Y., Zhou,B., Smith,D.K., Holmes,E.C., Zhu,H., Guan,Y. | 2015-12-17T00:00:00Z | ssRNA(+) | 1353 | spike protein | Saudi Arabia | Camelus |  |
| ALA50012.1 | Sabir,J.S.M., Lam,T.Y., Ahmed,M.M., Li,L., Shen,Y., AboAba,S., Qureshi,M.I., Abu-Zaid,M., Zhang,Y., Khiyami,M.A., Alharbi,N.S., Hajrah,N.H., Sabir,M.J., Mutwakil,M.H.Z., Kabli,S.A., Alsulaimany,F.A.S., Obaid,A.Y., Zhou,B., Smith,D.K., Holmes,E.C., Zhu,H., Guan,Y. | 2015-12-17T00:00:00Z | ssRNA(+) | 1353 | spike protein | Saudi Arabia | Camelus |  |
| ALA50023.1 | Sabir,J.S.M., Lam,T.Y., Ahmed,M.M., Li,L., Shen,Y., AboAba,S., Qureshi,M.I., Abu-Zaid,M., Zhang,Y., Khiyami,M.A., Alharbi,N.S., Hajrah,N.H., Sabir,M.J., Mutwakil,M.H.Z., Kabli,S.A., Alsulaimany,F.A.S., Obaid,A.Y., Zhou,B., Smith,D.K., Holmes,E.C., Zhu,H., Guan,Y. | 2015-12-17T00:00:00Z | ssRNA(+) | 1353 | spike protein | Saudi Arabia | Camelus |  |
| ALA50034.1 | Sabir,J.S.M., Lam,T.Y., Ahmed,M.M., Li,L., Shen,Y., AboAba,S., Qureshi,M.I., Abu-Zaid,M., Zhang,Y., Khiyami,M.A., Alharbi,N.S., Hajrah,N.H., Sabir,M.J., Mutwakil,M.H.Z., Kabli,S.A., Alsulaimany,F.A.S., Obaid,A.Y., Zhou,B., Smith,D.K., Holmes,E.C., Zhu,H., Guan,Y. | 2015-12-17T00:00:00Z | ssRNA(+) | 1353 | spike protein | Saudi Arabia | Camelus |  |
| ALA50045.1 | Sabir,J.S.M., Lam,T.Y., Ahmed,M.M., Li,L., Shen,Y., AboAba,S., Qureshi,M.I., Abu-Zaid,M., Zhang,Y., Khiyami,M.A., Alharbi,N.S., Hajrah,N.H., Sabir,M.J., Mutwakil,M.H.Z., Kabli,S.A., Alsulaimany,F.A.S., Obaid,A.Y., Zhou,B., Smith,D.K., Holmes,E.C., Zhu,H., Guan,Y. | 2015-12-17T00:00:00Z | ssRNA(+) | 1353 | spike protein | Saudi Arabia | Camelus |  |
| ALA50056.1 | Sabir,J.S.M., Lam,T.Y., Ahmed,M.M., Li,L., Shen,Y., AboAba,S., Qureshi,M.I., Abu-Zaid,M., Zhang,Y., Khiyami,M.A., Alharbi,N.S., Hajrah,N.H., Sabir,M.J., Mutwakil,M.H.Z., Kabli,S.A., Alsulaimany,F.A.S., Obaid,A.Y., Zhou,B., Smith,D.K., Holmes,E.C., Zhu,H., Guan,Y. | 2015-12-17T00:00:00Z | ssRNA(+) | 1353 | spike protein | Saudi Arabia | Camelus |  |
| ALA50067.1 | Sabir,J.S.M., Lam,T.Y., Ahmed,M.M., Li,L., Shen,Y., AboAba,S., Qureshi,M.I., Abu-Zaid,M., Zhang,Y., Khiyami,M.A., Alharbi,N.S., Hajrah,N.H., Sabir,M.J., Mutwakil,M.H.Z., Kabli,S.A., Alsulaimany,F.A.S., Obaid,A.Y., Zhou,B., Smith,D.K., Holmes,E.C., Zhu,H., Guan,Y. | 2015-12-17T00:00:00Z | ssRNA(+) | 1353 | spike protein | Saudi Arabia | Camelus |  |
| ALS20350.1 | Chu,D.K., Oladipo,J.O., Perera,R.A., Kuranga,S.A., Chan,S.M., Poon,L.L., Peiris,M., Chu,D.K.W., Perera,R.A.P.M., Chan,S.M.S., Poon,L.L.M. | 2015-12-16T00:00:00Z | ssRNA(+) | 1353 | spike protein | Nigeria | Camelus dromedarius | |
| ALS20351.1 | Chu,D.K., Oladipo,J.O., Perera,R.A., Kuranga,S.A., Chan,S.M., Poon,L.L., Peiris,M., Chu,D.K.W., Perera,R.A.P.M., Chan,S.M.S., Poon,L.L.M. | 2015-12-16T00:00:00Z | ssRNA(+) | 198 | spike protein | Nigeria | Camelus dromedarius | |
| ALS20352.1 | Chu,D.K., Oladipo,J.O., Perera,R.A., Kuranga,S.A., Chan,S.M., Poon,L.L., Peiris,M., Chu,D.K.W., Perera,R.A.P.M., Chan,S.M.S., Poon,L.L.M. | 2015-12-16T00:00:00Z | ssRNA(+) | 198 | spike protein | Nigeria | Camelus dromedarius | |
| ALS20353.1 | Chu,D.K., Oladipo,J.O., Perera,R.A., Kuranga,S.A., Chan,S.M., Poon,L.L., Peiris,M., Chu,D.K.W., Perera,R.A.P.M., Chan,S.M.S., Poon,L.L.M. | 2015-12-16T00:00:00Z | ssRNA(+) | 198 | spike protein | Nigeria | Camelus dromedarius | |
| ALS20354.1 | Chu,D.K., Oladipo,J.O., Perera,R.A., Kuranga,S.A., Chan,S.M., Poon,L.L., Peiris,M., Chu,D.K.W., Perera,R.A.P.M., Chan,S.M.S., Poon,L.L.M. | 2015-12-16T00:00:00Z | ssRNA(+) | 198 | spike protein | Nigeria | Camelus dromedarius | |
| ALS20355.1 | Chu,D.K., Oladipo,J.O., Perera,R.A., Kuranga,S.A., Chan,S.M., Poon,L.L., Peiris,M., Chu,D.K.W., Perera,R.A.P.M., Chan,S.M.S., Poon,L.L.M. | 2015-12-16T00:00:00Z | ssRNA(+) | 198 | spike protein | Nigeria | Camelus dromedarius | |
| ALR69641.1 | Wernery,U., Rasoul,I.E., Wong,E.Y., Joseph,M., Chen,Y., Jose,S., Tsang,A.K., Patteril,N.A., Chen,H., Elizabeth,S.K., Yuen,K.Y., Joseph,S., Xia,N., Wernery,R., Lau,S.K., Woo,P.C., El Rasoul,H., Wong,E.Y.M., Tsang,A.K.L., Patteril,N.A.G., Yuen,K.-Y., Lau,S.K.P., Woo,P.C.Y. | 2015-12-09T00:00:00Z | ssRNA(+) | 1353 | spike protein | United Arab Emirates | Camelus bactrianus | |
| AKO69634.1 | Khalafalla,A.I., Lu,X., Al-Mubarak,A.I., Dalab,A.H., Al-Busadah,K.A., Erdman,D.D., Al-Mubarak,A.I.A., Dalab,A.H.S., Al-Busada,K.A.S. | 2015-07-06T00:00:00Z | ssRNA(+) | 1353 | spike protein | Saudi Arabia | Camelus bactrianus | |
| AKO69635.1 | Khalafalla,A.I., Lu,X., Al-Mubarak,A.I., Dalab,A.H., Al-Busadah,K.A., Erdman,D.D., Al-Mubarak,A.I.A., Dalab,A.H.S., Al-Busada,K.A.S. | 2015-07-06T00:00:00Z | ssRNA(+) | 1353 | spike protein | Saudi Arabia | Camelus bactrianus | |
| AKO69636.1 | Khalafalla,A.I., Lu,X., Al-Mubarak,A.I., Dalab,A.H., Al-Busadah,K.A., Erdman,D.D., Al-Mubarak,A.I.A., Dalab,A.H.S., Al-Busada,K.A.S. | 2015-07-06T00:00:00Z | ssRNA(+) | 1353 | spike protein | Saudi Arabia | Camelus bactrianus | |
| AKA63509.1 | Khalafalla,A.I., Lu,X., Al-Mubarak,A.I., Dalab,A.H., Al-Busadah,K.A., Erdman,D.D., Al-Mubarak,A.I.A., Dalab,A.H.S., Al-Busada,K.A.S. | 2015-07-02T00:00:00Z | ssRNA(+) | 1353 | spike protein | Saudi Arabia | Camelus bactrianus | |
| AHX71946.1 | Raj,V.S., Farag,E.A., Reusken,C.B., Lamers,M.M., Pas,S.D., Voermans,J., Smits,S.L., Osterhaus,A.D., Al-Mawlawi,N., Al-Romaihi,H.E., AlHajri,M.M., El-Sayed,A.M., Mohran,K.A., Ghobashy,H., Alhajri,F., Al-Thani,M., Al-Marri,S.A., El-Maghraby,M.M., Koopmans,M.P., Haagmans,B.L., Stalin Raj,V., Farag,E.A.B.A., Reusken,C.B.E.M., Koopmans,M.P.G. | 2014-05-05T00:00:00Z | ssRNA(+) | 1353 | spike protein | Qatar | Camelus dromedarius | |
| AHX71941.1 | Nowotny,N., Kolodziejek,J., Stalin Raj,V., Farag,E.A.B.A., Lamers,M.M., Reusken,C.B.E.M., AlHajri,M.M., Koopmans,M.P.G., Haagmans,B.L., Al Dhahiry,S.H.S. | 2014-05-01T00:00:00Z | ssRNA(+) | 234 | spike protein | Qatar | Camelus dromedarius | |
| AHX71943.1 | Nowotny,N., Kolodziejek,J., Stalin Raj,V., Farag,E.A.B.A., Lamers,M.M., Reusken,C.B.E.M., AlHajri,M.M., Koopmans,M.P.G., Haagmans,B.L., Al Dhahiry,S.H.S. | 2014-05-01T00:00:00Z | ssRNA(+) | 291 | spike protein | Qatar | Camelus dromedarius | |
| AHL31359.1 | Alagaili,A.N., Briese,T., Mishra,N., Kapoor,V., Sameroff,S.C., de Wit,E., Munster,V.J., Hensley,L.E., Zalmout,I.S., Kapoor,A., Epstein,J.H., Karesh,W.B., Daszak,P., Mohammed,O.B., Lipkin,W.I. | 2014-03-12T00:00:00Z | ssRNA(+) | 347 | spike protein | Saudi Arabia | Camelus dromedarius | |
| AHL31360.1 | Alagaili,A.N., Briese,T., Mishra,N., Kapoor,V., Sameroff,S.C., de Wit,E., Munster,V.J., Hensley,L.E., Zalmout,I.S., Kapoor,A., Epstein,J.H., Karesh,W.B., Daszak,P., Mohammed,O.B., Lipkin,W.I. | 2014-03-12T00:00:00Z | ssRNA(+) | 347 | spike protein | Saudi Arabia | Camelus dromedarius | |
| AHL31361.1 | Alagaili,A.N., Briese,T., Mishra,N., Kapoor,V., Sameroff,S.C., de Wit,E., Munster,V.J., Hensley,L.E., Zalmout,I.S., Kapoor,A., Epstein,J.H., Karesh,W.B., Daszak,P., Mohammed,O.B., Lipkin,W.I. | 2014-03-12T00:00:00Z | ssRNA(+) | 347 | spike protein | Saudi Arabia | Camelus dromedarius | |
| AHL31362.1 | Alagaili,A.N., Briese,T., Mishra,N., Kapoor,V., Sameroff,S.C., de Wit,E., Munster,V.J., Hensley,L.E., Zalmout,I.S., Kapoor,A., Epstein,J.H., Karesh,W.B., Daszak,P., Mohammed,O.B., Lipkin,W.I. | 2014-03-12T00:00:00Z | ssRNA(+) | 347 | spike protein | Saudi Arabia | Camelus dromedarius | |
| AHL31363.1 | Alagaili,A.N., Briese,T., Mishra,N., Kapoor,V., Sameroff,S.C., de Wit,E., Munster,V.J., Hensley,L.E., Zalmout,I.S., Kapoor,A., Epstein,J.H., Karesh,W.B., Daszak,P., Mohammed,O.B., Lipkin,W.I. | 2014-03-12T00:00:00Z | ssRNA(+) | 347 | spike protein | Saudi Arabia | Camelus dromedarius | |
| AHL31364.1 | Alagaili,A.N., Briese,T., Mishra,N., Kapoor,V., Sameroff,S.C., de Wit,E., Munster,V.J., Hensley,L.E., Zalmout,I.S., Kapoor,A., Epstein,J.H., Karesh,W.B., Daszak,P., Mohammed,O.B., Lipkin,W.I. | 2014-03-12T00:00:00Z | ssRNA(+) | 347 | spike protein | Saudi Arabia | Camelus dromedarius | |
| AHL31365.1 | Alagaili,A.N., Briese,T., Mishra,N., Kapoor,V., Sameroff,S.C., de Wit,E., Munster,V.J., Hensley,L.E., Zalmout,I.S., Kapoor,A., Epstein,J.H., Karesh,W.B., Daszak,P., Mohammed,O.B., Lipkin,W.I. | 2014-03-12T00:00:00Z | ssRNA(+) | 347 | spike protein | Saudi Arabia | Camelus dromedarius | |
| AHL31366.1 | Alagaili,A.N., Briese,T., Mishra,N., Kapoor,V., Sameroff,S.C., de Wit,E., Munster,V.J., Hensley,L.E., Zalmout,I.S., Kapoor,A., Epstein,J.H., Karesh,W.B., Daszak,P., Mohammed,O.B., Lipkin,W.I. | 2014-03-12T00:00:00Z | ssRNA(+) | 347 | spike protein | Saudi Arabia | Camelus dromedarius | |
| AHL31367.1 | Alagaili,A.N., Briese,T., Mishra,N., Kapoor,V., Sameroff,S.C., de Wit,E., Munster,V.J., Hensley,L.E., Zalmout,I.S., Kapoor,A., Epstein,J.H., Karesh,W.B., Daszak,P., Mohammed,O.B., Lipkin,W.I. | 2014-03-12T00:00:00Z | ssRNA(+) | 347 | spike protein | Saudi Arabia | Camelus dromedarius | |
| AHL31368.1 | Alagaili,A.N., Briese,T., Mishra,N., Kapoor,V., Sameroff,S.C., de Wit,E., Munster,V.J., Hensley,L.E., Zalmout,I.S., Kapoor,A., Epstein,J.H., Karesh,W.B., Daszak,P., Mohammed,O.B., Lipkin,W.I. | 2014-03-12T00:00:00Z | ssRNA(+) | 347 | spike protein | Saudi Arabia | Camelus dromedarius | |
| AGY29650.2 | Ithete,N.L., Stoffberg,S., Corman,V.M., Cottontail,V.M., Richards,L.R., Schoeman,M.C., Drosten,C., Drexler,J.F., Preiser,W., Stoffberg Hockey,S., Richards,L. | 2013-10-23T00:00:00Z | ssRNA(+) | 1344 | spike protein | South Africa | Laephotis capensis | |
| AHY04435.1 | Nowotny,N., Kolodziejek,J. | 2014-05-03T00:00:00Z | ssRNA(+) | 291 | spike1 | Oman | Camelus dromedarius | |
| AHY04436.1 | Nowotny,N., Kolodziejek,J. | 2014-05-03T00:00:00Z | ssRNA(+) | 234 | spike2 | Oman | Camelus dromedarius | |
| UYV90078.1 | Kandeil,A., Gomaa,M.R., Shehata,M.M., El Taweel,A.N., Kayed,A., El-Shesheny,R., Alabady,M., Bahl,J., Ali,M.A., Kayali,G. | 2022-10-31T00:00:00Z | ssRNA(+) | 1353 | surface glycoprotein | Egypt | Camelus dromedarius | |
| UYV90088.1 | Kandeil,A., Gomaa,M.R., Shehata,M.M., El Taweel,A.N., Kayed,A., El-Shesheny,R., Alabady,M., Bahl,J., Ali,M.A., Kayali,G. | 2022-10-31T00:00:00Z | ssRNA(+) | 1358 | surface glycoprotein | Egypt | Camelus dromedarius | |
| 8DGV_A | Zhou,P., Song,G., Liu,H., Yuan,M., He,W.-T., Beutler,N., Zhu,X., Tse,L.V., Martinez,D.R., Schafer,A., Anzanello,F., Yong,P., Peng,L., Dueker,K., Musharrafieh,R., Callaghan,S., Capozzola,T., Limbo,O., Parren,M., Garcia,E., Rawlings,S.A., Smith,D.M., Nemazee,D., Jardine,J.G., Safonova,Y., Rogers,T.F., Wilson,I.A., Baric,R.S., Gralinski,L.E., Burton,D.R., Andrabi,R. | 2023-01-25T00:00:00Z | ssRNA(+) | 27 |  |  |  |  |
| 7V5J_A | Wang,X., Zhao,J., Wang,Z., Zeng,J., Zhang,S., Wang,Y. | 2022-09-21T00:00:00Z | ssRNA(+) | 1189 |  |  |  |  |
| 7V5J_B | Wang,X., Zhao,J., Wang,Z., Zeng,J., Zhang,S., Wang,Y. | 2022-09-21T00:00:00Z | ssRNA(+) | 1189 |  |  |  |  |
| 7V5J_C | Wang,X., Zhao,J., Wang,Z., Zeng,J., Zhang,S., Wang,Y. | 2022-09-21T00:00:00Z | ssRNA(+) | 1189 |  |  |  |  |
| 7V6N_A | Wang,X., Zhao,J., Wang,Z., Zeng,J., Zhang,S., Wang,Y. | 2022-09-14T00:00:00Z | ssRNA(+) | 1189 |  |  |  |  |
| 7V6N_B | Wang,X., Zhao,J., Wang,Z., Zeng,J., Zhang,S., Wang,Y. | 2022-09-14T00:00:00Z | ssRNA(+) | 1189 |  |  |  |  |
| 7V6N_C | Wang,X., Zhao,J., Wang,Z., Zeng,J., Zhang,S., Wang,Y. | 2022-09-14T00:00:00Z | ssRNA(+) | 1189 |  |  |  |  |
| 7V3L_A | Wang,X., Zhao,J., Wang,Z., Wang,Y., Zeng,J. | 2022-08-17T00:00:00Z | ssRNA(+) | 1290 |  |  |  |  |
| 7V3L_B | Wang,X., Zhao,J., Wang,Z., Wang,Y., Zeng,J. | 2022-08-17T00:00:00Z | ssRNA(+) | 1290 |  |  |  |  |
| 7V3L_C | Wang,X., Zhao,J., Wang,Z., Wang,Y., Zeng,J. | 2022-08-17T00:00:00Z | ssRNA(+) | 1290 |  |  |  |  |
| 8DGY_A | Dampalla,C.S., Nguyen,H.N., Rathnayake,A.D., Kim,Y., Perera,K.D., Madden,T.K., Thurman,H.A., Machen,A.J., Kashipathy,M.M., Liu,L., Battaile,K.P., Lovell,S., Chang,K.O., Groutas,W.C., Chamandi,S.D., Picard,H.R., Thruman,H.A. | 2022-07-06T00:00:00Z | ssRNA(+) | 313 |  |  |  |  |
| 8CZT_A | Dampalla,C.S., Nguyen,H.N., Rathnayake,A.D., Kim,Y., Perera,K.D., Madden,T.K., Thurman,H.A., Machen,A.J., Kashipathy,M.M., Liu,L., Battaile,K.P., Lovell,S., Chang,K.O., Groutas,W.C., Chamandi,S.D., Picard,H.R., Thruman,H.A. | 2022-06-22T00:00:00Z | ssRNA(+) | 313 |  |  |  |  |
| 8CZU_A | Dampalla,C.S., Nguyen,H.N., Rathnayake,A.D., Kim,Y., Perera,K.D., Madden,T.K., Thurman,H.A., Machen,A.J., Kashipathy,M.M., Liu,L., Battaile,K.P., Lovell,S., Chang,K.O., Groutas,W.C., Chamandi,S.D., Picard,H.R., Thruman,H.A. | 2022-06-22T00:00:00Z | ssRNA(+) | 313 |  |  |  |  |
| 8CZV_A | Dampalla,C.S., Nguyen,H.N., Rathnayake,A.D., Kim,Y., Perera,K.D., Madden,T.K., Thurman,H.A., Machen,A.J., Kashipathy,M.M., Liu,L., Battaile,K.P., Lovell,S., Chang,K.O., Groutas,W.C., Chamandi,S.D., Picard,H.R., Thruman,H.A. | 2022-06-22T00:00:00Z | ssRNA(+) | 313 |  |  |  |  |
| 7VTC_A | Lin,C., Zhou,X.L., Zhong,F.L., Zhang,J., Li,J. | 2022-03-30T00:00:00Z | ssRNA(+) | 322 |  |  |  |  |
| 7VTC_B | Lin,C., Zhou,X.L., Zhong,F.L., Zhang,J., Li,J. | 2022-03-30T00:00:00Z | ssRNA(+) | 322 |  |  |  |  |
| 7TQ8_A | Dampalla,C.S., Nguyen,H.N., Rathnayake,A.D., Kim,Y., Perera,K.D., Madden,T.K., Thurman,H.A., Machen,A.J., Kashipathy,M.M., Liu,L., Battaile,K.P., Lovell,S., Chang,K.O., Groutas,W.C., Chamandi,S.D., Picard,H.R., Thruman,H.A. | 2022-03-02T00:00:00Z | ssRNA(+) | 313 |  |  |  |  |
| 7TQ7_A | Dampalla,C.S., Nguyen,H.N., Rathnayake,A.D., Kim,Y., Perera,K.D., Madden,T.K., Thurman,H.A., Machen,A.J., Kashipathy,M.M., Liu,L., Battaile,K.P., Lovell,S., Chang,K.O., Groutas,W.C., Chamandi,S.D., Picard,H.R., Thruman,H.A. | 2022-02-09T00:00:00Z | ssRNA(+) | 313 |  |  |  |  |
| 7T3Y_A | Dampalla,C.S., Rathnayake,A.D., Galasiti Kankanamalage,A.C., Kim,Y., Perera,K.D., Nguyen,H.N., Miller,M.J., Madden,T.K., Picard,H.R., Thurman,H.A., Kashipathy,M.M., Liu,L., Battaile,K.P., Lovell,S., Chang,K.O., Groutas,W.C., Chamandi,S.D. | 2021-12-22T00:00:00Z | ssRNA(+) | 313 |  |  |  |  |
| 7T3Y_B | Dampalla,C.S., Rathnayake,A.D., Galasiti Kankanamalage,A.C., Kim,Y., Perera,K.D., Nguyen,H.N., Miller,M.J., Madden,T.K., Picard,H.R., Thurman,H.A., Kashipathy,M.M., Liu,L., Battaile,K.P., Lovell,S., Chang,K.O., Groutas,W.C., Chamandi,S.D. | 2021-12-22T00:00:00Z | ssRNA(+) | 313 |  |  |  |  |
| 7T3Z_A | Dampalla,C.S., Rathnayake,A.D., Galasiti Kankanamalage,A.C., Kim,Y., Perera,K.D., Nguyen,H.N., Miller,M.J., Madden,T.K., Picard,H.R., Thurman,H.A., Kashipathy,M.M., Liu,L., Battaile,K.P., Lovell,S., Chang,K.O., Groutas,W.C., Chamandi,S.D. | 2021-12-22T00:00:00Z | ssRNA(+) | 313 |  |  |  |  |
| 7T3Z_B | Dampalla,C.S., Rathnayake,A.D., Galasiti Kankanamalage,A.C., Kim,Y., Perera,K.D., Nguyen,H.N., Miller,M.J., Madden,T.K., Picard,H.R., Thurman,H.A., Kashipathy,M.M., Liu,L., Battaile,K.P., Lovell,S., Chang,K.O., Groutas,W.C., Chamandi,S.D. | 2021-12-22T00:00:00Z | ssRNA(+) | 313 |  |  |  |  |
| 7T40_A | Dampalla,C.S., Rathnayake,A.D., Galasiti Kankanamalage,A.C., Kim,Y., Perera,K.D., Nguyen,H.N., Miller,M.J., Madden,T.K., Picard,H.R., Thurman,H.A., Kashipathy,M.M., Liu,L., Battaile,K.P., Lovell,S., Chang,K.O., Groutas,W.C., Chamandi,S.D. | 2021-12-22T00:00:00Z | ssRNA(+) | 313 |  |  |  |  |
| 7T41_A | Dampalla,C.S., Rathnayake,A.D., Galasiti Kankanamalage,A.C., Kim,Y., Perera,K.D., Nguyen,H.N., Miller,M.J., Madden,T.K., Picard,H.R., Thurman,H.A., Kashipathy,M.M., Liu,L., Battaile,K.P., Lovell,S., Chang,K.O., Groutas,W.C., Chamandi,S.D. | 2021-12-22T00:00:00Z | ssRNA(+) | 313 |  |  |  |  |
| 7S3M_A | Hsieh,C.L., Werner,A.P., Leist,S.R., Stevens,L.J., Falconer,E., Goldsmith,J.A., Chou,C.W., Abiona,O.M., West,A., Westendorf,K., Muthuraman,K., Fritch,E.J., Dinnon KH,3r., Schafer,A., Denison,M.R., Chappell,J.D., Baric,R.S., Graham,B.S., Corbett,K.S., McLellan,J.S. | 2021-10-27T00:00:00Z | ssRNA(+) | 15 |  |  |  |  |
| 7M55_A | Sauer,M.M., Tortorici,M.A., Park,Y.J., Walls,A.C., Homad,L., Acton,O.J., Bowen,J.E., Wang,C., Xiong,X., de van,de., Quispe,J., Hoffstrom,B.G., Bosch,B.J., McGuire,A.T., Veesler,D., Center for Structural Genomics of Infectious Diseases (CSGID), Seattle Structural Genomics Center for Infectious Disease (SSGCID) | 2021-05-28T00:00:00Z | ssRNA(+) | 15 |  |  |  |  |
| 7M5E_A | Sauer,M.M., Tortorici,M.A., Park,Y.J., Walls,A.C., Homad,L., Acton,O.J., Bowen,J.E., Wang,C., Xiong,X., de van,de., Quispe,J., Hoffstrom,B.G., Bosch,B.J., McGuire,A.T., Veesler,D., Seattle Structural Genomics Center for Infectious Disease (SSGCID) | 2021-05-28T00:00:00Z | ssRNA(+) | 1359 |  |  |  |  |
| 7M5E_C | Sauer,M.M., Tortorici,M.A., Park,Y.J., Walls,A.C., Homad,L., Acton,O.J., Bowen,J.E., Wang,C., Xiong,X., de van,de., Quispe,J., Hoffstrom,B.G., Bosch,B.J., McGuire,A.T., Veesler,D., Seattle Structural Genomics Center for Infectious Disease (SSGCID) | 2021-05-28T00:00:00Z | ssRNA(+) | 1359 |  |  |  |  |
| 7M5E_E | Sauer,M.M., Tortorici,M.A., Park,Y.J., Walls,A.C., Homad,L., Acton,O.J., Bowen,J.E., Wang,C., Xiong,X., de van,de., Quispe,J., Hoffstrom,B.G., Bosch,B.J., McGuire,A.T., Veesler,D., Seattle Structural Genomics Center for Infectious Disease (SSGCID) | 2021-05-28T00:00:00Z | ssRNA(+) | 1359 |  |  |  |  |
| 6LZ6_A | Hou,M.H., Lin,S.M., Hsu,J.N. | 2021-02-26T00:00:00Z | ssRNA(+) | 130 |  |  |  |  |
| 6LZ6_B | Hou,M.H., Lin,S.M., Hsu,J.N. | 2021-02-26T00:00:00Z | ssRNA(+) | 130 |  |  |  |  |
| 6LZ6_C | Hou,M.H., Lin,S.M., Hsu,J.N. | 2021-02-26T00:00:00Z | ssRNA(+) | 130 |  |  |  |  |
| 6LZ6_D | Hou,M.H., Lin,S.M., Hsu,J.N. | 2021-02-26T00:00:00Z | ssRNA(+) | 130 |  |  |  |  |
| 6LZ8_A | Hou,M.H., Lin,S.M., Hsu,J.N. | 2021-02-26T00:00:00Z | ssRNA(+) | 130 |  |  |  |  |
| 6LZ8_B | Hou,M.H., Lin,S.M., Hsu,J.N. | 2021-02-26T00:00:00Z | ssRNA(+) | 130 |  |  |  |  |
| 6LZ8_C | Hou,M.H., Lin,S.M., Hsu,J.N. | 2021-02-26T00:00:00Z | ssRNA(+) | 130 |  |  |  |  |
| 6LZ8_D | Hou,M.H., Lin,S.M., Hsu,J.N. | 2021-02-26T00:00:00Z | ssRNA(+) | 130 |  |  |  |  |
| 6LNN_A | Hou,M.H., Lin,S.M., Hsu,J.N. | 2021-01-14T00:00:00Z | ssRNA(+) | 130 |  |  |  |  |
| 6LNN_B | Hou,M.H., Lin,S.M., Hsu,J.N. | 2021-01-14T00:00:00Z | ssRNA(+) | 130 |  |  |  |  |
| 6LNN_C | Hou,M.H., Lin,S.M., Hsu,J.N. | 2021-01-14T00:00:00Z | ssRNA(+) | 130 |  |  |  |  |
| 6LNN_D | Hou,M.H., Lin,S.M., Hsu,J.N. | 2021-01-14T00:00:00Z | ssRNA(+) | 130 |  |  |  |  |
| 5W9H_p | Pallesen,J., Wang,N., Corbett,K.S., Wrapp,D., Kirchdoerfer,R.N., Turner,H.L., Cottrell,C.A., Becker,M.M., Wang,L., Shi,W., Kong,W.P., Andres,E.L., Kettenbach,A.N., Denison,M.R., Chappell,J.D., Graham,B.S., Ward,A.B., McLellan,J.S. | 2020-12-01T00:00:00Z | ssRNA(+) | 1329 |  |  |  |  |
| 5W9H_q | Pallesen,J., Wang,N., Corbett,K.S., Wrapp,D., Kirchdoerfer,R.N., Turner,H.L., Cottrell,C.A., Becker,M.M., Wang,L., Shi,W., Kong,W.P., Andres,E.L., Kettenbach,A.N., Denison,M.R., Chappell,J.D., Graham,B.S., Ward,A.B., McLellan,J.S. | 2020-12-01T00:00:00Z | ssRNA(+) | 1329 |  |  |  |  |
| 5W9H_r | Pallesen,J., Wang,N., Corbett,K.S., Wrapp,D., Kirchdoerfer,R.N., Turner,H.L., Cottrell,C.A., Becker,M.M., Wang,L., Shi,W., Kong,W.P., Andres,E.L., Kettenbach,A.N., Denison,M.R., Chappell,J.D., Graham,B.S., Ward,A.B., McLellan,J.S. | 2020-12-01T00:00:00Z | ssRNA(+) | 1329 |  |  |  |  |
| 6VGY_A | Rathnayake,A.D., Zheng,J., Kim,Y., Perera,K.D., Mackin,S., Meyerholz,D.K., Kashipathy,M.M., Battaile,K.P., Lovell,S., Perlman,S., Groutas,W.C., Chang,K.O., Nguyen,H.N. | 2020-08-14T00:00:00Z | ssRNA(+) | 313 |  |  |  |  |
| 6VGY_B | Rathnayake,A.D., Zheng,J., Kim,Y., Perera,K.D., Mackin,S., Meyerholz,D.K., Kashipathy,M.M., Battaile,K.P., Lovell,S., Perlman,S., Groutas,W.C., Chang,K.O., Nguyen,H.N. | 2020-08-14T00:00:00Z | ssRNA(+) | 313 |  |  |  |  |
| 6VGZ_A | Rathnayake,A.D., Zheng,J., Kim,Y., Perera,K.D., Mackin,S., Meyerholz,D.K., Kashipathy,M.M., Battaile,K.P., Lovell,S., Perlman,S., Groutas,W.C., Chang,K.O., Nguyen,H.N. | 2020-08-14T00:00:00Z | ssRNA(+) | 313 |  |  |  |  |
| 6VGZ_B | Rathnayake,A.D., Zheng,J., Kim,Y., Perera,K.D., Mackin,S., Meyerholz,D.K., Kashipathy,M.M., Battaile,K.P., Lovell,S., Perlman,S., Groutas,W.C., Chang,K.O., Nguyen,H.N. | 2020-08-14T00:00:00Z | ssRNA(+) | 313 |  |  |  |  |
| 6VH0_A | Rathnayake,A.D., Zheng,J., Kim,Y., Perera,K.D., Mackin,S., Meyerholz,D.K., Kashipathy,M.M., Battaile,K.P., Lovell,S., Perlman,S., Groutas,W.C., Chang,K.O., Nguyen,H.N. | 2020-08-14T00:00:00Z | ssRNA(+) | 313 |  |  |  |  |
| 6VH0_B | Rathnayake,A.D., Zheng,J., Kim,Y., Perera,K.D., Mackin,S., Meyerholz,D.K., Kashipathy,M.M., Battaile,K.P., Lovell,S., Perlman,S., Groutas,W.C., Chang,K.O., Nguyen,H.N. | 2020-08-14T00:00:00Z | ssRNA(+) | 313 |  |  |  |  |
| 6VH1_A | Rathnayake,A.D., Zheng,J., Kim,Y., Perera,K.D., Mackin,S., Meyerholz,D.K., Kashipathy,M.M., Battaile,K.P., Lovell,S., Perlman,S., Groutas,W.C., Chang,K.O., Nguyen,H.N. | 2020-08-14T00:00:00Z | ssRNA(+) | 313 |  |  |  |  |
| 6VH1_B | Rathnayake,A.D., Zheng,J., Kim,Y., Perera,K.D., Mackin,S., Meyerholz,D.K., Kashipathy,M.M., Battaile,K.P., Lovell,S., Perlman,S., Groutas,W.C., Chang,K.O., Nguyen,H.N. | 2020-08-14T00:00:00Z | ssRNA(+) | 313 |  |  |  |  |
| 6VH2_A | Rathnayake,A.D., Zheng,J., Kim,Y., Perera,K.D., Mackin,S., Meyerholz,D.K., Kashipathy,M.M., Battaile,K.P., Lovell,S., Perlman,S., Groutas,W.C., Chang,K.O., Nguyen,H.N. | 2020-08-14T00:00:00Z | ssRNA(+) | 313 |  |  |  |  |
| 6VH2_B | Rathnayake,A.D., Zheng,J., Kim,Y., Perera,K.D., Mackin,S., Meyerholz,D.K., Kashipathy,M.M., Battaile,K.P., Lovell,S., Perlman,S., Groutas,W.C., Chang,K.O., Nguyen,H.N. | 2020-08-14T00:00:00Z | ssRNA(+) | 313 |  |  |  |  |
| 6VH3_A | Rathnayake,A.D., Zheng,J., Kim,Y., Perera,K.D., Mackin,S., Meyerholz,D.K., Kashipathy,M.M., Battaile,K.P., Lovell,S., Perlman,S., Groutas,W.C., Chang,K.O., Nguyen,H.N. | 2020-08-14T00:00:00Z | ssRNA(+) | 313 |  |  |  |  |
| 6VH3_B | Rathnayake,A.D., Zheng,J., Kim,Y., Perera,K.D., Mackin,S., Meyerholz,D.K., Kashipathy,M.M., Battaile,K.P., Lovell,S., Perlman,S., Groutas,W.C., Chang,K.O., Nguyen,H.N. | 2020-08-14T00:00:00Z | ssRNA(+) | 313 |  |  |  |  |
| 7C02_A | Dai,L., Zheng,T., Xu,K., Han,Y., Xu,L., Huang,E., An,Y., Cheng,Y., Li,S., Liu,M., Yang,M., Li,Y., Cheng,H., Yuan,Y., Zhang,W., Ke,C., Wong,G., Qi,J., Qin,C., Yan,J., Gao,G.F. | 2020-07-29T00:00:00Z | ssRNA(+) | 246 |  |  |  |  |
| 7C02_B | Dai,L., Zheng,T., Xu,K., Han,Y., Xu,L., Huang,E., An,Y., Cheng,Y., Li,S., Liu,M., Yang,M., Li,Y., Cheng,H., Yuan,Y., Zhang,W., Ke,C., Wong,G., Qi,J., Qin,C., Yan,J., Gao,G.F. | 2020-07-29T00:00:00Z | ssRNA(+) | 246 |  |  |  |  |
| 6WAR_A | Wrapp,D., De Vlieger,D., Corbett,K.S., Torres,G.M., Wang,N., Van Breedam,W., Roose,K., van Schie,L., Hoffmann,M., Pohlmann,S., Graham,B.S., Callewaert,N., Schepens,B., Saelens,X., McLellan,J.S. | 2020-04-08T00:00:00Z | ssRNA(+) | 231 |  |  |  |  |
| 6WAR_C | Wrapp,D., De Vlieger,D., Corbett,K.S., Torres,G.M., Wang,N., Van Breedam,W., Roose,K., van Schie,L., Hoffmann,M., Pohlmann,S., Graham,B.S., Callewaert,N., Schepens,B., Saelens,X., McLellan,J.S. | 2020-04-08T00:00:00Z | ssRNA(+) | 231 |  |  |  |  |
| 6WAR_E | Wrapp,D., De Vlieger,D., Corbett,K.S., Torres,G.M., Wang,N., Van Breedam,W., Roose,K., van Schie,L., Hoffmann,M., Pohlmann,S., Graham,B.S., Callewaert,N., Schepens,B., Saelens,X., McLellan,J.S. | 2020-04-08T00:00:00Z | ssRNA(+) | 231 |  |  |  |  |
| 6WAR_G | Wrapp,D., De Vlieger,D., Corbett,K.S., Torres,G.M., Wang,N., Van Breedam,W., Roose,K., van Schie,L., Hoffmann,M., Pohlmann,S., Graham,B.S., Callewaert,N., Schepens,B., Saelens,X., McLellan,J.S. | 2020-04-08T00:00:00Z | ssRNA(+) | 231 |  |  |  |  |
| 6WAR_I | Wrapp,D., De Vlieger,D., Corbett,K.S., Torres,G.M., Wang,N., Van Breedam,W., Roose,K., van Schie,L., Hoffmann,M., Pohlmann,S., Graham,B.S., Callewaert,N., Schepens,B., Saelens,X., McLellan,J.S. | 2020-04-08T00:00:00Z | ssRNA(+) | 231 |  |  |  |  |
| 6WAR_K | Wrapp,D., De Vlieger,D., Corbett,K.S., Torres,G.M., Wang,N., Van Breedam,W., Roose,K., van Schie,L., Hoffmann,M., Pohlmann,S., Graham,B.S., Callewaert,N., Schepens,B., Saelens,X., McLellan,J.S. | 2020-04-08T00:00:00Z | ssRNA(+) | 231 |  |  |  |  |
| 6WAR_M | Wrapp,D., De Vlieger,D., Corbett,K.S., Torres,G.M., Wang,N., Van Breedam,W., Roose,K., van Schie,L., Hoffmann,M., Pohlmann,S., Graham,B.S., Callewaert,N., Schepens,B., Saelens,X., McLellan,J.S. | 2020-04-08T00:00:00Z | ssRNA(+) | 231 |  |  |  |  |
| 6WAR_O | Wrapp,D., De Vlieger,D., Corbett,K.S., Torres,G.M., Wang,N., Van Breedam,W., Roose,K., van Schie,L., Hoffmann,M., Pohlmann,S., Graham,B.S., Callewaert,N., Schepens,B., Saelens,X., McLellan,J.S. | 2020-04-08T00:00:00Z | ssRNA(+) | 231 |  |  |  |  |
| 6KL2_A | Lin,S.M., Lin,S.C., Hsu,J.N., Chang,C.K., Chien,C.M., Wang,Y.S., Wu,H.Y., Jeng,U.S., Kehn-Hall,K., Hou,M.H. | 2020-03-26T00:00:00Z | ssRNA(+) | 144 |  |  |  |  |
| 6KL2_B | Lin,S.M., Lin,S.C., Hsu,J.N., Chang,C.K., Chien,C.M., Wang,Y.S., Wu,H.Y., Jeng,U.S., Kehn-Hall,K., Hou,M.H. | 2020-03-26T00:00:00Z | ssRNA(+) | 144 |  |  |  |  |
| 6KL2_C | Lin,S.M., Lin,S.C., Hsu,J.N., Chang,C.K., Chien,C.M., Wang,Y.S., Wu,H.Y., Jeng,U.S., Kehn-Hall,K., Hou,M.H. | 2020-03-26T00:00:00Z | ssRNA(+) | 144 |  |  |  |  |
| 6KL2_D | Lin,S.M., Lin,S.C., Hsu,J.N., Chang,C.K., Chien,C.M., Wang,Y.S., Wu,H.Y., Jeng,U.S., Kehn-Hall,K., Hou,M.H. | 2020-03-26T00:00:00Z | ssRNA(+) | 144 |  |  |  |  |
| 6KL5_A | Lin,S.M., Lin,S.C., Hsu,J.N., Chang,C.K., Chien,C.M., Wang,Y.S., Wu,H.Y., Jeng,U.S., Kehn-Hall,K., Hou,M.H. | 2020-03-26T00:00:00Z | ssRNA(+) | 144 |  |  |  |  |
| 6KL5_B | Lin,S.M., Lin,S.C., Hsu,J.N., Chang,C.K., Chien,C.M., Wang,Y.S., Wu,H.Y., Jeng,U.S., Kehn-Hall,K., Hou,M.H. | 2020-03-26T00:00:00Z | ssRNA(+) | 144 |  |  |  |  |
| 6KL5_C | Lin,S.M., Lin,S.C., Hsu,J.N., Chang,C.K., Chien,C.M., Wang,Y.S., Wu,H.Y., Jeng,U.S., Kehn-Hall,K., Hou,M.H. | 2020-03-26T00:00:00Z | ssRNA(+) | 144 |  |  |  |  |
| 6KL5_D | Lin,S.M., Lin,S.C., Hsu,J.N., Chang,C.K., Chien,C.M., Wang,Y.S., Wu,H.Y., Jeng,U.S., Kehn-Hall,K., Hou,M.H. | 2020-03-26T00:00:00Z | ssRNA(+) | 144 |  |  |  |  |
| 6KL6_A | Lin,S.M., Lin,S.C., Hsu,J.N., Chang,C.K., Chien,C.M., Wang,Y.S., Wu,H.Y., Jeng,U.S., Kehn-Hall,K., Hou,M.H. | 2020-03-26T00:00:00Z | ssRNA(+) | 144 |  |  |  |  |
| 6KL6_B | Lin,S.M., Lin,S.C., Hsu,J.N., Chang,C.K., Chien,C.M., Wang,Y.S., Wu,H.Y., Jeng,U.S., Kehn-Hall,K., Hou,M.H. | 2020-03-26T00:00:00Z | ssRNA(+) | 144 |  |  |  |  |
| 6KL6_C | Lin,S.M., Lin,S.C., Hsu,J.N., Chang,C.K., Chien,C.M., Wang,Y.S., Wu,H.Y., Jeng,U.S., Kehn-Hall,K., Hou,M.H. | 2020-03-26T00:00:00Z | ssRNA(+) | 144 |  |  |  |  |
| 6KL6_D | Lin,S.M., Lin,S.C., Hsu,J.N., Chang,C.K., Chien,C.M., Wang,Y.S., Wu,H.Y., Jeng,U.S., Kehn-Hall,K., Hou,M.H. | 2020-03-26T00:00:00Z | ssRNA(+) | 144 |  |  |  |  |
| 6Q04_A | Park,Y.J., Walls,A.C., Wang,Z., Sauer,M.M., Li,W., Tortorici,M.A., Bosch,B.J., DiMaio,F., Veesler,D., Sauer,M., DiMaio,F.D., Seattle Structural Genomics Center for Infectious Disease (SSGCID) | 2019-12-16T00:00:00Z | ssRNA(+) | 1359 |  |  |  |  |
| 6Q04_B | Park,Y.J., Walls,A.C., Wang,Z., Sauer,M.M., Li,W., Tortorici,M.A., Bosch,B.J., DiMaio,F., Veesler,D., Sauer,M., DiMaio,F.D., Seattle Structural Genomics Center for Infectious Disease (SSGCID) | 2019-12-16T00:00:00Z | ssRNA(+) | 1359 |  |  |  |  |
| 6Q04_C | Park,Y.J., Walls,A.C., Wang,Z., Sauer,M.M., Li,W., Tortorici,M.A., Bosch,B.J., DiMaio,F., Veesler,D., Sauer,M., DiMaio,F.D., Seattle Structural Genomics Center for Infectious Disease (SSGCID) | 2019-12-16T00:00:00Z | ssRNA(+) | 1359 |  |  |  |  |
| 6Q05_A | Park,Y.J., Walls,A.C., Wang,Z., Sauer,M.M., Li,W., Tortorici,M.A., Bosch,B.J., DiMaio,F., Veesler,D., Sauer,M., DiMaio,F.D., Seattle Structural Genomics Center for Infectious Disease (SSGCID) | 2019-12-16T00:00:00Z | ssRNA(+) | 1359 |  |  |  |  |
| 6Q05_B | Park,Y.J., Walls,A.C., Wang,Z., Sauer,M.M., Li,W., Tortorici,M.A., Bosch,B.J., DiMaio,F., Veesler,D., Sauer,M., DiMaio,F.D., Seattle Structural Genomics Center for Infectious Disease (SSGCID) | 2019-12-16T00:00:00Z | ssRNA(+) | 1359 |  |  |  |  |
| 6Q05_C | Park,Y.J., Walls,A.C., Wang,Z., Sauer,M.M., Li,W., Tortorici,M.A., Bosch,B.J., DiMaio,F., Veesler,D., Sauer,M., DiMaio,F.D., Seattle Structural Genomics Center for Infectious Disease (SSGCID) | 2019-12-16T00:00:00Z | ssRNA(+) | 1359 |  |  |  |  |
| 6Q06_A | Park,Y.J., Walls,A.C., Wang,Z., Sauer,M.M., Li,W., Tortorici,M.A., Bosch,B.J., DiMaio,F., Veesler,D., Sauer,M., DiMaio,F.D., Seattle Structural Genomics Center for Infectious Disease (SSGCID) | 2019-12-16T00:00:00Z | ssRNA(+) | 1359 |  |  |  |  |
| 6Q06_B | Park,Y.J., Walls,A.C., Wang,Z., Sauer,M.M., Li,W., Tortorici,M.A., Bosch,B.J., DiMaio,F., Veesler,D., Sauer,M., DiMaio,F.D., Seattle Structural Genomics Center for Infectious Disease (SSGCID) | 2019-12-16T00:00:00Z | ssRNA(+) | 1359 |  |  |  |  |
| 6Q06_C | Park,Y.J., Walls,A.C., Wang,Z., Sauer,M.M., Li,W., Tortorici,M.A., Bosch,B.J., DiMaio,F., Veesler,D., Sauer,M., DiMaio,F.D., Seattle Structural Genomics Center for Infectious Disease (SSGCID) | 2019-12-16T00:00:00Z | ssRNA(+) | 1359 |  |  |  |  |
| 6Q07_A | Park,Y.J., Walls,A.C., Wang,Z., Sauer,M.M., Li,W., Tortorici,M.A., Bosch,B.J., DiMaio,F., Veesler,D., Sauer,M., DiMaio,F.D., Seattle Structural Genomics Center for Infectious Disease (SSGCID) | 2019-12-16T00:00:00Z | ssRNA(+) | 1359 |  |  |  |  |
| 6Q07_B | Park,Y.J., Walls,A.C., Wang,Z., Sauer,M.M., Li,W., Tortorici,M.A., Bosch,B.J., DiMaio,F., Veesler,D., Sauer,M., DiMaio,F.D., Seattle Structural Genomics Center for Infectious Disease (SSGCID) | 2019-12-16T00:00:00Z | ssRNA(+) | 1359 |  |  |  |  |
| 6Q07_C | Park,Y.J., Walls,A.C., Wang,Z., Sauer,M.M., Li,W., Tortorici,M.A., Bosch,B.J., DiMaio,F., Veesler,D., Sauer,M., DiMaio,F.D., Seattle Structural Genomics Center for Infectious Disease (SSGCID) | 2019-12-16T00:00:00Z | ssRNA(+) | 1359 |  |  |  |  |
| 6PZ8_A | Wang,N., Rosen,O., Wang,L., Turner,H.L., Stevens,L.J., Corbett,K.S., Bowman,C.A., Pallesen,J., Shi,W., Zhang,Y., Leung,K., Kirchdoerfer,R.N., Becker,M.M., Denison,M.R., Chappell,J.D., Ward,A.B., Graham,B.S., McLellan,J.S. | 2019-10-10T00:00:00Z | ssRNA(+) | 472 |  |  |  |  |
| 6PZ8_B | Wang,N., Rosen,O., Wang,L., Turner,H.L., Stevens,L.J., Corbett,K.S., Bowman,C.A., Pallesen,J., Shi,W., Zhang,Y., Leung,K., Kirchdoerfer,R.N., Becker,M.M., Denison,M.R., Chappell,J.D., Ward,A.B., Graham,B.S., McLellan,J.S. | 2019-10-10T00:00:00Z | ssRNA(+) | 726 |  |  |  |  |
| 6PZ8_E | Wang,N., Rosen,O., Wang,L., Turner,H.L., Stevens,L.J., Corbett,K.S., Bowman,C.A., Pallesen,J., Shi,W., Zhang,Y., Leung,K., Kirchdoerfer,R.N., Becker,M.M., Denison,M.R., Chappell,J.D., Ward,A.B., Graham,B.S., McLellan,J.S. | 2019-10-10T00:00:00Z | ssRNA(+) | 472 |  |  |  |  |
| 6PZ8_F | Wang,N., Rosen,O., Wang,L., Turner,H.L., Stevens,L.J., Corbett,K.S., Bowman,C.A., Pallesen,J., Shi,W., Zhang,Y., Leung,K., Kirchdoerfer,R.N., Becker,M.M., Denison,M.R., Chappell,J.D., Ward,A.B., Graham,B.S., McLellan,J.S. | 2019-10-10T00:00:00Z | ssRNA(+) | 726 |  |  |  |  |
| 6PZ8_I | Wang,N., Rosen,O., Wang,L., Turner,H.L., Stevens,L.J., Corbett,K.S., Bowman,C.A., Pallesen,J., Shi,W., Zhang,Y., Leung,K., Kirchdoerfer,R.N., Becker,M.M., Denison,M.R., Chappell,J.D., Ward,A.B., Graham,B.S., McLellan,J.S. | 2019-10-10T00:00:00Z | ssRNA(+) | 472 |  |  |  |  |
| 6PZ8_J | Wang,N., Rosen,O., Wang,L., Turner,H.L., Stevens,L.J., Corbett,K.S., Bowman,C.A., Pallesen,J., Shi,W., Zhang,Y., Leung,K., Kirchdoerfer,R.N., Becker,M.M., Denison,M.R., Chappell,J.D., Ward,A.B., Graham,B.S., McLellan,J.S. | 2019-10-10T00:00:00Z | ssRNA(+) | 726 |  |  |  |  |
| 6PXH_A | Wang,N., Rosen,O., Wang,L., Turner,H.L., Stevens,L.J., Corbett,K.S., Bowman,C.A., Pallesen,J., Shi,W., Zhang,Y., Leung,K., Kirchdoerfer,R.N., Becker,M.M., Denison,M.R., Chappell,J.D., Ward,A.B., Graham,B.S., McLellan,J.S. | 2019-09-27T00:00:00Z | ssRNA(+) | 342 |  |  |  |  |
| 6PXH_B | Wang,N., Rosen,O., Wang,L., Turner,H.L., Stevens,L.J., Corbett,K.S., Bowman,C.A., Pallesen,J., Shi,W., Zhang,Y., Leung,K., Kirchdoerfer,R.N., Becker,M.M., Denison,M.R., Chappell,J.D., Ward,A.B., Graham,B.S., McLellan,J.S. | 2019-09-27T00:00:00Z | ssRNA(+) | 342 |  |  |  |  |
| 6J11_A | Zhou,H., Chen,Y., Zhang,S., Niu,P., Qin,K., Jia,W., Huang,B., Lan,J., Zhang,L., Tan,W., Wang,X., Tang,W. | 2019-07-26T00:00:00Z | ssRNA(+) | 336 |  |  |  |  |
| 6J11_B | Zhou,H., Chen,Y., Zhang,S., Niu,P., Qin,K., Jia,W., Huang,B., Lan,J., Zhang,L., Tan,W., Wang,X., Tang,W. | 2019-07-26T00:00:00Z | ssRNA(+) | 336 |  |  |  |  |
| 6J11_C | Zhou,H., Chen,Y., Zhang,S., Niu,P., Qin,K., Jia,W., Huang,B., Lan,J., Zhang,L., Tan,W., Wang,X., Tang,W. | 2019-07-26T00:00:00Z | ssRNA(+) | 336 |  |  |  |  |
| 5ZU7_A | Lin,M.-H., Cho,C.-C., Chien,C.-Y., Hsu,C.-H. | 2019-06-17T00:00:00Z | ssRNA(+) | 168 |  |  |  |  |
| 5ZU9_A | Lin,M.-H., Cho,C.-C., Chien,C.-Y., Hsu,C.-H. | 2019-06-17T00:00:00Z | ssRNA(+) | 168 |  |  |  |  |
| 5ZUA_A | Lin,M.-H., Cho,C.-C., Chien,C.-Y., Hsu,C.-H. | 2019-06-17T00:00:00Z | ssRNA(+) | 168 |  |  |  |  |
| 5ZUB_A | Lin,M.-H., Cho,C.-C., Chien,C.-Y., Hsu,C.-H. | 2019-06-17T00:00:00Z | ssRNA(+) | 168 |  |  |  |  |
| 5ZVK_A | Xia,S., Yan,L., Xu,W., Agrawal,A.S., Algaissi,A., Tseng,C.K., Wang,Q., Du,L., Tan,W., Wilson,I.A., Jiang,S., Yang,B., Lu,L. | 2019-04-11T00:00:00Z | ssRNA(+) | 80 |  |  |  |  |
| 5ZVK_B | Xia,S., Yan,L., Xu,W., Agrawal,A.S., Algaissi,A., Tseng,C.K., Wang,Q., Du,L., Tan,W., Wilson,I.A., Jiang,S., Yang,B., Lu,L. | 2019-04-11T00:00:00Z | ssRNA(+) | 80 |  |  |  |  |
| 5ZVK_C | Xia,S., Yan,L., Xu,W., Agrawal,A.S., Algaissi,A., Tseng,C.K., Wang,Q., Du,L., Tan,W., Wilson,I.A., Jiang,S., Yang,B., Lu,L. | 2019-04-11T00:00:00Z | ssRNA(+) | 80 |  |  |  |  |
| 6G13_A | Nguyen,T.H., Lichiere,J., Canard,B., Papageorgiou,N., Attoumani,S., Ferron,F., Coutard,B., Nguyen,T.H.V., Ferron,F.P. | 2019-03-04T00:00:00Z | ssRNA(+) | 126 |  |  |  |  |
| 6G13_B | Nguyen,T.H., Lichiere,J., Canard,B., Papageorgiou,N., Attoumani,S., Ferron,F., Coutard,B., Nguyen,T.H.V., Ferron,F.P. | 2019-03-04T00:00:00Z | ssRNA(+) | 126 |  |  |  |  |
| 6G13_C | Nguyen,T.H., Lichiere,J., Canard,B., Papageorgiou,N., Attoumani,S., Ferron,F., Coutard,B., Nguyen,T.H.V., Ferron,F.P. | 2019-03-04T00:00:00Z | ssRNA(+) | 126 |  |  |  |  |
| 6G13_D | Nguyen,T.H., Lichiere,J., Canard,B., Papageorgiou,N., Attoumani,S., Ferron,F., Coutard,B., Nguyen,T.H.V., Ferron,F.P. | 2019-03-04T00:00:00Z | ssRNA(+) | 126 |  |  |  |  |
| 6NB3_A | Walls,A.C., Xiong,X., Park,Y.J., Tortorici,M.A., Snijder,J., Quispe,J., Cameroni,E., Gopal,R., Dai,M., Lanzavecchia,A., Zambon,M., Rey,F.A., Corti,D., Veesler,D., Snijder,S., Mian,D., Seattle Structural Genomics Center for Infectious Disease (SSGCID) | 2019-02-07T00:00:00Z | ssRNA(+) | 1359 |  |  |  |  |
| 6NB3_B | Walls,A.C., Xiong,X., Park,Y.J., Tortorici,M.A., Snijder,J., Quispe,J., Cameroni,E., Gopal,R., Dai,M., Lanzavecchia,A., Zambon,M., Rey,F.A., Corti,D., Veesler,D., Snijder,S., Mian,D., Seattle Structural Genomics Center for Infectious Disease (SSGCID) | 2019-02-07T00:00:00Z | ssRNA(+) | 1359 |  |  |  |  |
| 6NB3_C | Walls,A.C., Xiong,X., Park,Y.J., Tortorici,M.A., Snijder,J., Quispe,J., Cameroni,E., Gopal,R., Dai,M., Lanzavecchia,A., Zambon,M., Rey,F.A., Corti,D., Veesler,D., Snijder,S., Mian,D., Seattle Structural Genomics Center for Infectious Disease (SSGCID) | 2019-02-07T00:00:00Z | ssRNA(+) | 1359 |  |  |  |  |
| 6NB4_A | Walls,A.C., Xiong,X., Park,Y.J., Tortorici,M.A., Snijder,J., Quispe,J., Cameroni,E., Gopal,R., Dai,M., Lanzavecchia,A., Zambon,M., Rey,F.A., Corti,D., Veesler,D., Snijder,S., Mian,D., Seattle Structural Genomics Center for Infectious Disease (SSGCID) | 2019-02-07T00:00:00Z | ssRNA(+) | 1359 |  |  |  |  |
| 6NB4_B | Walls,A.C., Xiong,X., Park,Y.J., Tortorici,M.A., Snijder,J., Quispe,J., Cameroni,E., Gopal,R., Dai,M., Lanzavecchia,A., Zambon,M., Rey,F.A., Corti,D., Veesler,D., Snijder,S., Mian,D., Seattle Structural Genomics Center for Infectious Disease (SSGCID) | 2019-02-07T00:00:00Z | ssRNA(+) | 1359 |  |  |  |  |
| 6NB4_C | Walls,A.C., Xiong,X., Park,Y.J., Tortorici,M.A., Snijder,J., Quispe,J., Cameroni,E., Gopal,R., Dai,M., Lanzavecchia,A., Zambon,M., Rey,F.A., Corti,D., Veesler,D., Snijder,S., Mian,D., Seattle Structural Genomics Center for Infectious Disease (SSGCID) | 2019-02-07T00:00:00Z | ssRNA(+) | 1359 |  |  |  |  |
| 5YN5_A | Wei,S.M., Yang,L., Ke,Z.H., Liu,Q.Y., Yang,Z.Z., Chen,Y., Guo,D.Y., Fan,C.P. | 2018-12-06T00:00:00Z | ssRNA(+) | 303 |  |  |  |  |
| 5YN5_B | Wei,S.M., Yang,L., Ke,Z.H., Liu,Q.Y., Yang,Z.Z., Chen,Y., Guo,D.Y., Fan,C.P. | 2018-12-06T00:00:00Z | ssRNA(+) | 140 |  |  |  |  |
| 5YN6_A | Wei,S.M., Yang,L., Ke,Z.H., Liu,Q.Y., Che,Y., Yang,Z.Z., Guo,D.Y., Fan,C.P. | 2018-12-06T00:00:00Z | ssRNA(+) | 303 |  |  |  |  |
| 5YN6_B | Wei,S.M., Yang,L., Ke,Z.H., Liu,Q.Y., Che,Y., Yang,Z.Z., Guo,D.Y., Fan,C.P. | 2018-12-06T00:00:00Z | ssRNA(+) | 140 |  |  |  |  |
| 5YN8_A | Wei,S.M., Yang,L., Ke,Z.H., Liu,Q.Y., Yang,Z.Z., Chen,Y., Guo,D.Y., Fan,C.P. | 2018-12-06T00:00:00Z | ssRNA(+) | 303 |  |  |  |  |
| 5YN8_B | Wei,S.M., Yang,L., Ke,Z.H., Liu,Q.Y., Yang,Z.Z., Chen,Y., Guo,D.Y., Fan,C.P. | 2018-12-06T00:00:00Z | ssRNA(+) | 140 |  |  |  |  |
| 5YNB_A | Wei,S.M., Yang,L., Ke,Z.H., Liu,Q.Y., Chen,Y., Yang,Z.Z., Guo,D.Y., Fan,C.P. | 2018-12-06T00:00:00Z | ssRNA(+) | 303 |  |  |  |  |
| 5YNB_B | Wei,S.M., Yang,L., Ke,Z.H., Liu,Q.Y., Chen,Y., Yang,Z.Z., Guo,D.Y., Fan,C.P. | 2018-12-06T00:00:00Z | ssRNA(+) | 140 |  |  |  |  |
| 5YNF_A | Wei,S.M., Yang,L., Ke,Z.H., Yang,Z.Z., Liu,Q.Y., Chen,Y., Guo,D.Y., Fan,C.P. | 2018-12-06T00:00:00Z | ssRNA(+) | 303 |  |  |  |  |
| 5YNF_B | Wei,S.M., Yang,L., Ke,Z.H., Yang,Z.Z., Liu,Q.Y., Chen,Y., Guo,D.Y., Fan,C.P. | 2018-12-06T00:00:00Z | ssRNA(+) | 140 |  |  |  |  |
| 5YNI_A | Wei,S.M., Yang,L., Ke,Z.H., Liu,Q.Y., Yang,Z.Z., Chen,Y., Guo,D.Y., Fan,C.P. | 2018-12-06T00:00:00Z | ssRNA(+) | 303 |  |  |  |  |
| 5YNI_B | Wei,S.M., Yang,L., Ke,Z.H., Liu,Q.Y., Yang,Z.Z., Chen,Y., Guo,D.Y., Fan,C.P. | 2018-12-06T00:00:00Z | ssRNA(+) | 140 |  |  |  |  |
| 5YNJ_A | Wei,S.M., Yang,L., Ke,Z.H., Liu,Q.Y., Yang,Z.Z., Chen,Y., Guo,D.Y., Fa,C.P., Fan,C.P. | 2018-12-06T00:00:00Z | ssRNA(+) | 303 |  |  |  |  |
| 5YNJ_B | Wei,S.M., Yang,L., Ke,Z.H., Liu,Q.Y., Yang,Z.Z., Chen,Y., Guo,D.Y., Fa,C.P., Fan,C.P. | 2018-12-06T00:00:00Z | ssRNA(+) | 140 |  |  |  |  |
| 5YNM_A | Wei,S.M., Yang,L., Ke,Z.H., Liu,Q.Y., Yang,Z.Z., Chen,Y., Guo,D.Y., Fan,C.P. | 2018-12-06T00:00:00Z | ssRNA(+) | 303 |  |  |  |  |
| 5YNM_B | Wei,S.M., Yang,L., Ke,Z.H., Liu,Q.Y., Yang,Z.Z., Chen,Y., Guo,D.Y., Fan,C.P. | 2018-12-06T00:00:00Z | ssRNA(+) | 140 |  |  |  |  |
| 5YNN_A | Wei,S.M., Yang,L., Ke,Z.H., Liu,Q.Y., Yang,Z.Z., Chen,Y., Guo,D.Y., Fan,C.P. | 2018-12-06T00:00:00Z | ssRNA(+) | 303 |  |  |  |  |
| 5YNN_B | Wei,S.M., Yang,L., Ke,Z.H., Liu,Q.Y., Yang,Z.Z., Chen,Y., Guo,D.Y., Fan,C.P. | 2018-12-06T00:00:00Z | ssRNA(+) | 140 |  |  |  |  |
| 5YNO_A | Wei,S.M., Yang,L., Ke,Z.H., Liu,Q.Y., Chen,Y., Guo,D.Y., Fan,C.P. | 2018-12-06T00:00:00Z | ssRNA(+) | 303 |  |  |  |  |
| 5YNO_B | Wei,S.M., Yang,L., Ke,Z.H., Liu,Q.Y., Chen,Y., Guo,D.Y., Fan,C.P. | 2018-12-06T00:00:00Z | ssRNA(+) | 140 |  |  |  |  |
| 5YNP_A | Wei,S.M., Yang,L., Ke,Z.H., Liu,Q.Y., Yang,Z.Z., Chen,Y., Guo,D.Y., Fan,C.P. | 2018-12-06T00:00:00Z | ssRNA(+) | 303 |  |  |  |  |
| 5YNP_B | Wei,S.M., Yang,L., Ke,Z.H., Liu,Q.Y., Yang,Z.Z., Chen,Y., Guo,D.Y., Fan,C.P. | 2018-12-06T00:00:00Z | ssRNA(+) | 140 |  |  |  |  |
| 5YNQ_A | Wei,S.M., Yang,L., Ke,Z.H., Liu,Q.Y., Yang,Z.Z., Chen,Y., Guo,D.Y., Fan,C.P. | 2018-12-06T00:00:00Z | ssRNA(+) | 303 |  |  |  |  |
| 5YNQ_B | Wei,S.M., Yang,L., Ke,Z.H., Liu,Q.Y., Yang,Z.Z., Chen,Y., Guo,D.Y., Fan,C.P. | 2018-12-06T00:00:00Z | ssRNA(+) | 140 |  |  |  |  |
| 6BI8_A | Clasman,J.R., Everett,R.K., Srinivasan,K., Mesecar,A.D., Clasman,J.C. | 2018-11-11T00:00:00Z | ssRNA(+) | 259 |  |  |  |  |
| 6BI8_B | Clasman,J.R., Everett,R.K., Srinivasan,K., Mesecar,A.D., Clasman,J.C. | 2018-11-11T00:00:00Z | ssRNA(+) | 259 |  |  |  |  |
| 5YVD_A | Zhang,L., Li,L., Yan,L., Ming,Z., Jia,Z., Lou,Z., Rao,Z. | 2018-09-11T00:00:00Z | ssRNA(+) | 343 |  |  |  |  |
| 5YVD_B | Zhang,L., Li,L., Yan,L., Ming,Z., Jia,Z., Lou,Z., Rao,Z. | 2018-09-11T00:00:00Z | ssRNA(+) | 343 |  |  |  |  |
[truncated: 28,807 more chars]
